# Genesis and influencing factors of the colour of chrysoprase

**DOI:** 10.1038/s41598-021-89406-x

**Published:** 2021-05-11

**Authors:** Yuansheng Jiang, Ying Guo

**Affiliations:** School of Gemmology, China University of Geosciences (Beijing), No.29 Xueyuan Road, Beijing, 100083 China

**Keywords:** Mineralogy, Optical spectroscopy

## Abstract

The genesis and influencing factors of the colour of chrysoprase were studied based on the results of transmission electron microscopy and X-ray fluorescence, ultraviolet–visible and Raman spectroscopies. The results show that under a 6504-K fluorescent lamp, chrysoprase colour is divided into the grades of fancy, fancy intense and fancy deep. The lightness of chrysoprase is affected mainly by its chromium content, the chroma is affected by its nickel content and the hue angle is affected by the sum of its chromium and ferrum contents. The colour of chrysoprase is related significantly to the transmission window that occurs between the two main bands centred at 380 and 660 nm and the absorption peaks at 380 and 660 nm in the ultraviolet–visible spectrum. Chrysoprase with low crystallinity has more nickel and a higher chroma. The inclusions that cause the chrysoprase colour have been identified as pimelite.

## Introduction

With a beautiful apple-green colour, chrysoprase is a fine-crystalline variety of polycrystalline quartz. It is found in quartz veins in Ni-bearing rocks all over the world, such as in Australia^1^, Tanzania^2,3^, Poland^4^, Kazakhstan^5^, South Africa and America. Although early ideas were that the colour of chrysoprase was caused by Fe, Co and Cu, it is now known to be related directly to the Ni content, which ranges from a few tenths of a percent to several percent by weight^6^. Chrysoprase colour arises from admixed fine-grained Ni compounds in the silica matrix rather than from substitutional Ni in the silica itself^7^.

According to earlier studies on the mineralogical and colour properties of chrysoprase, the origin of its colour can be summarized into two models. In one, the colour is due to finely disseminated hydrous Ni silicates such as willemseite^1^, kerolite^5,8^ and pimelite^4^ minerals that are usually associated with chrysoprase deposits. In the other model, the colour is due to finely divided bunsenite (NiO)^9,10^, the evidence for which is from cubic forms observed in transmission electron microscopy (TEM) micrographs of replicas of fractured chrysoprase and a weak reflection at 2.39 Å in the X-ray pattern of chrysoprase from Kazakhstan^10^.

When discussing chrysoprase colour, as well as exploring its cause, one cannot neglect some internal factors that affect the colour of chrysoprase. The greenness of chrysoprase is in the form of a series of graded colours, which is of great value in the study of colourimetry. In recent years, colourimetry has played an important role in gemmology, involving mainly colour-change garnet^11–14^, tourmaline^15,16^, sapphire^17^, alexandrite^18^, peridot^19,20^, cubic zirconia^21^, blue amber^22^, jadeite-jade^23–27^, citrine^28^ and amethyst^29^, among others. However, chrysoprase colour has been subjected to little evaluation or analysis of its influencing factors to date. Chrysoprase colour was quantified for the first time by Sachanbiński et al. based on the CIE 1931 colour-space system^5^, but in CIE 1931 colour-space system the geometrical distance between two given colours is inconsistent with human visual perception. To solve that problem, the CIE 1976 L*a*b* uniform colour-space system was established. As a basis for representing colours quantitatively, the CIE 1976 L*a*b* uniform colour space (1) has good colour uniformity, making the visual distance between colours proportional to the Euclidean distance between colour coordinates, and (2) accords fully with the subjective law that the visual colour difference in the red–green direction is smaller than that in the yellow–blue direction^30^. The system comprises colourimetric coordinates a* and b* and lightness L*, and the chroma C* and hue angle *h*° can be calculated from a* and b* as:

1 $$C^{*} = \sqrt {a^{{{*}2}} + b^{{{*}2}} } ,$$

2 $$h^{ \circ } = \arctan \frac{{b^{*} }}{{a^{*} }}.$$

**Figure 1:**
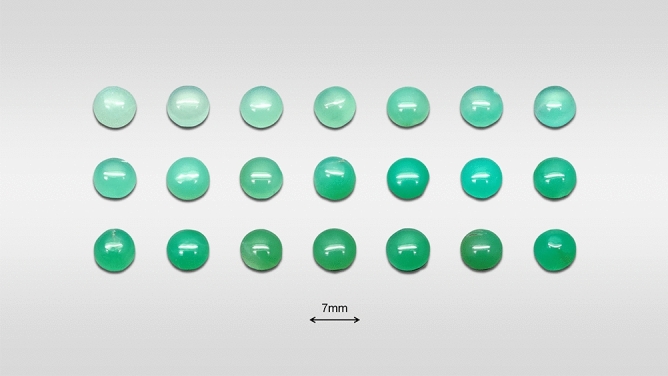
Photograph of some of the samples used in the present study. The round flat samples used for colour-parameter testing had an average mass of 3.96 g.

We used 41 samples of natural chrysoprase from Australia, the colours of which displayed continuously from pale green to vivid green. To obtain the colour parameters effectively, each sample was cut into a polished cabochon with a diameter of 7 mm, with no inclusion in the inner part of the sample as seen by the naked eye. Some of the samples are shown in Fig. 1.

In the present study, chrysoprase colour is characterized quantitatively based on the CIE 1976 L*a*b* uniform colour space, and the influences of transition elements, UV–Vis spectrum and crystallinity on the colour are discussed. The cause of chrysoprase colour is analysed using TEM.

## Results and discussion

### Colour quantification and classification

**Figure 2:**
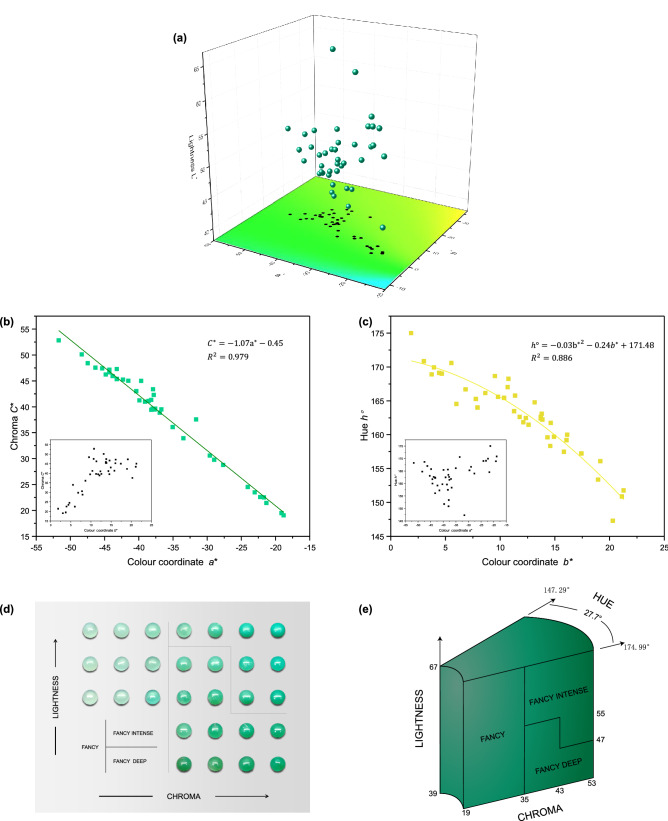
The colour analysis of chrysoprase. (**a**) 41 chrysoprase green plots in CIE 1976 *L*^∗^*a*^∗^*b*^∗^ uniform colour space. (**b**) A high negative correlation between the colourimetric coordinate a^*^ and its chroma C^*^. (**c**) A high negative correlation between the colourimetric coordinate b^*^ and its hue angle *h*°. (**d**) Chrysoprase colour is graded into the levels of fancy, fancy intense and fancy deep. (**e**) Chrysoprase colour grading system.

Under a 6504-K fluorescent lamp, the colours of the 41 chrysoprase samples were measured to obtain their values of lightness L* (39.39–66.10), colourimetric coordinates a* (− 51.71 to − 18.81) and b* (1.87–21.29), chroma C* (19.05–52.82) and hue angle *h*° (147.29–174.99). These values are consistent with the colour appearance of chrysoprase. The colour parameters of the 41 samples are projected in the CIE 1976 L*a*b* uniform colour space (Fig. 2a), where the chroma C* is indicated by the distance from the projection point to the origin, and the hue angle *h*° is indicated by the angle between the line from the projection point to the origin and the + a* axis. The results show that the 41 chrysoprase samples were all green but in different degrees.

Since chroma C* and hue angle *h*° are calculated by a* and b*, through the analysis of the colour data of 41 chrysoprases, it is found that there is a high negative correlation between the colourimetric coordinate a* and its chroma C*(Pearson’s *r* =  − 0.990, R^2^ = 0.979). As shown in Fig. 2b, compared with a*, the relationship between b* and chroma C* is more discrete, so the chroma of chrysoprase is mainly controlled by a*. Besides, it is found that there is a high negative correlation between the colourimetric coordinate b* and its hue angle *h*°(Pearson’s *r* =  − 0.928, R^2^ = 0.886). As shown in Fig. 2c, compared with b*, the relationship between a* and hue angle *h*° is more discrete, so the hue of chrysoprase is mainly controlled by b*.

Based on the CIE 1976 L^*^a^*^b^*^ uniform colour-space system, *K*-means cluster analysis and Fisher discriminant analysis were used to classify the chrysoprase colours. These two methods have been verified for classifying the colours of gemstones such as peridot^19^, tourmaline^16^, jadeite-jade^23–27^ and amethyst^29^, among others. *K*-means cluster analysis is a statistical analysis technique that divides the research object into relatively homogeneous groups. As one of the most important methods in multivariate statistical analysis, Fisher discriminant analysis summarizes the regularity of all types of samples and establishes discriminant formulae and criteria to identify the types of new sample points according to the information provided by known sample categories^19^.

The colour parameters *L*^∗^, *a*^∗^ and *b*^∗^ were analysed by *K*-means cluster analysis, and the clustering results were significant when the cluster number was 3 (Table 1). Fisher discriminant analysis was used to analyse these three types of chrysoprase colour, and the discriminant formulae are as follows:

3 $$F1 = 3.547L^{*} - 2.414a^{*} + 2.119b^{*} - 132.011,$$

4 $$F2 = 3.609L^{*} - 3.265a^{*} + 3.190b^{*} - 173.217,$$

5 $$F3 = 3.973L^{*} - 3.727a^{*} + 2.901b^{*} - 207.002.$$

**Table 1:** ANOVA results, Cluster centre and Fisher discriminant accuracy.

ANOVA results for L*, a* and b*						
	Clustering		Error		F	Sig
	Mean square	df	Mean square	df		
L*	285.447	2	21.447	38	13.309	0.000
a*	1183.557	2	16.293	38	72.643	0.000
b*	364.989	2	8.390	38	43.501	0.000

Fisher discriminant accuracy						
	1	2	3	Total (%)		
1	**100%**	0.0	0.0	100		
2	0.0	**95.5%**	4.5%	100		
3	0.0	12.5%	**87.5%**	100		

Cluster centre	1	2	3			
L*	54.09	46.18	52.99			
a*	− 24.54	− 39.69	− 44.94			
b*	5.07	15.05	11.65			
Simulated colour						

Bold words indicate the accuracy of the prediction.

The colour parameters *L*^∗^, *a*^∗^ and *b*^∗^ were entered into the Fisher discriminant formulae, and Table 1 shows the accuracy of the corresponding classification results. The numbers on the diagonal indicate the prediction accuracy, while the others indicate the prediction error. Among the 41 groups of data, two are misjudged. The accuracy of colour data updating is 95.12%, which shows that the model is effective for classifying chrysoprase colour.

From the results of the *K*-means cluster analysis and Fisher discriminant analysis and by imitating the coloured-diamond grading system of the Gemological Institute of America^31^, the chrysoprase colour is divided into the grades of (1) fancy, (2) fancy intense and (3) fancy deep, with a hue angle ranging from 147.29° to 174.99° (Fig. 2d). The chrysoprase colour grading system so established is shown in Fig. 2e.

### Chemical composition analysis

Energy-dispersive X-ray fluorescence (EDXRF) spectrometry is a quick and non-destructive means of detecting the presence of most elements in chrysoprase, and the test results are given in the Supplementary Table S1 online. The results show that the range of weight percentage [wt%] of the main oxide in the 41 chrysoprase samples is w(SiO_2_) = 92.416–99.099, and those of the other oxides are w(NiO) = 0.380–5.631, w(SO_3_) = 0.217–0.470, w(K_2_O) = 0–0.077, w(Cr_2_O_3_) = 0.008–0.035, w(CaO) = 0–1.427, w(Fe_2_O_3_) = 0–0.025 and w(ZnO) = 0–0.018. Of these, Ni, Cr and Fe are transition-metal elements, which are often related to the colour of gemstones. Bivariate correlation analysis was used to analyse the correlation between the contents of transition-metal elements and the colour parameters of the 41 chrysoprase samples (Table 2). The test was two-tailed, and the linear relationship between the two groups of data can be judged by the Pearson correlation coefficient *r*: when |*r*|< 0.3, there is weak or no correlation; when 0.3 ≤|*r*|< 0.5, there is low correlation; when 0.5 ≤|*r*|< 0.8, there is moderate correlation; when |*r*|≥ 0.8, there is high correlation.

**Table 2:** Results of bivariate correlation analysis.

Oxide		L*	C*	*h*°
NiO	*r*	− 0.479**	0.832**	− 0.308
	Sig	0.002	0.000	0.050
Cr_2_O_3_	*r*	− 0.685**	0.502**	− 0.695**
	Sig	0.000	0.001	0.000
Fe_2_O_3_	*r*	0.022	− 0.111	− 0.167
	Sig	0.890	0.491	0.297
Cr_2_O_3_ + Fe_2_O_3_	*r*	− 0.587**	0.377*	− 0.704**
	Sig	0.000	0.015	0.000

*At level 0.01 (double tail), the correlation was significant. *At level 0.05 (double tail), the correlation was significant.

**Figure 3:**
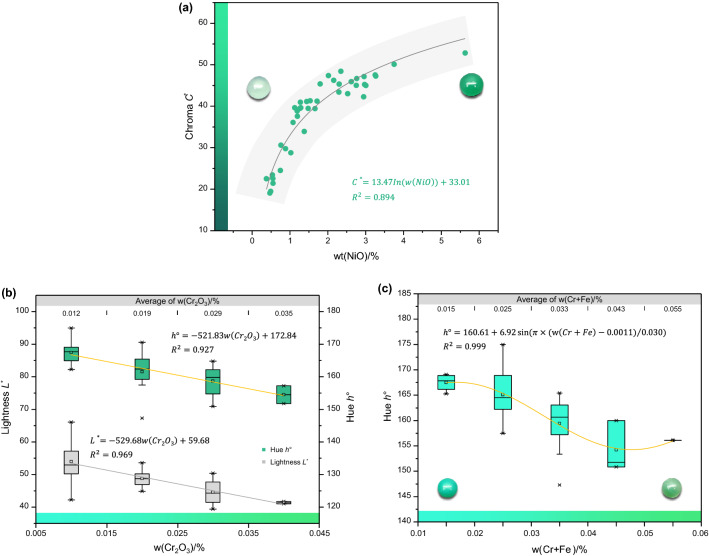
Relationship between colour parameters and transition-metal elements. (**a**) There is a highly positive correlation between Ni content and chroma. (**b**) With increasing Cr content, the lightness and hue angle exhibit downward trends. (**c**) Both Cr and Fe affect the hue angle; the higher the content of Cr and Fe, the smaller the hue angle.

The results show high positive correlation between w(NiO) and chroma C* in chrysoprase (*r*_C*_ = 0.832), as shown in Fig. 3a, and low negative correlations with lightness L* and hue angle *h*° (*r*_L*_ =  − 0.479, *r*_*h*°_ =  − 0.308). There are moderate negative correlations between w(Cr_2_O_3_) and lightness L* and hue angle *h*°(*r*_L*_ =  − 0.685, *r*_*h*°_ =  − 0.695), as shown in Fig. 3b, and moderate positive correlation (*r*_C*_ = 0.502) with chroma C*. Sachanbiński et al.^5^ reasoned that the yellow hue of chrysoprase is due to the existence of Fe^3+^. Although the present bivariate correlation analysis shows no significant correlation between w(Fe_2_O_3_) and the colour parameters, the sum of w(Fe_2_O_3_) and w(Cr_2_O_3_) has significant negative correlation with the hue angle *h*°; the Pearson correlation coefficient *r* is − 0.704, whose absolute value is greater than that of the Pearson correlation coefficient of w(Cr_2_O_3_) and *h*°. Therefore, the hue angle of chrysoprase is affected by the content of Fe and Cr; i.e. the higher the sum of the Fe and Cr contents, the lower the hue angle, and the hue tends to yellowish green (Fig. 3c).

### UV–Vis spectral analysis

**Figure 4:**
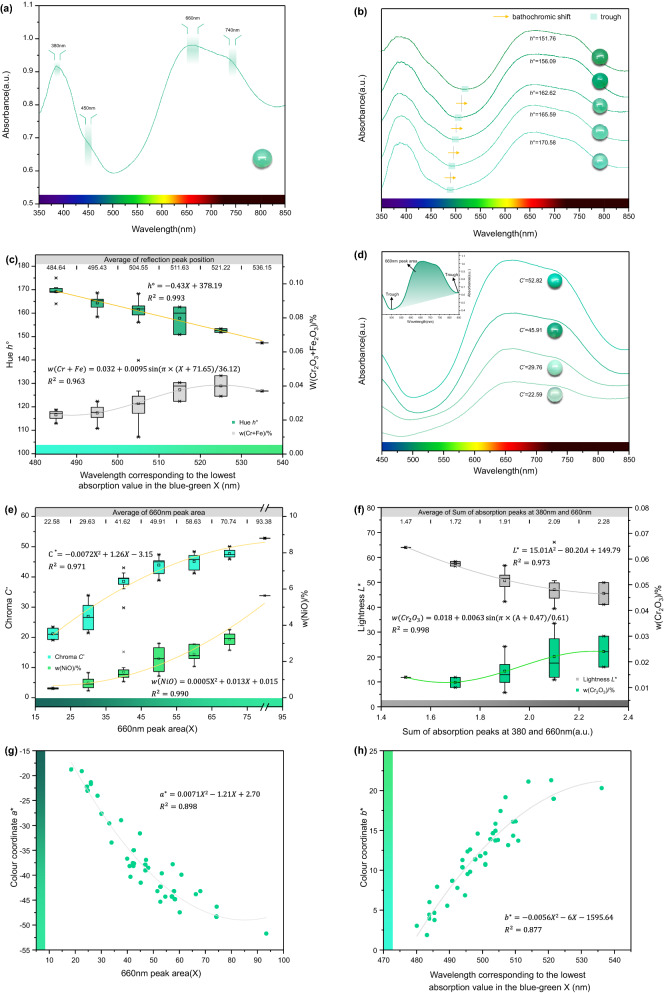
UV–Vis spectra for chrysoprase samples. (**a**) The UV–Vis spectra of chrysoprase are characterized by Ni^2+^ absorption features. (**b**) The transmission window between the two main bands of chrysoprase centred at 380 and 660 nm is responsible for the hue of the samples. (**c**) Relationship between the wavelength corresponding to the lowest absorption value in the blue–green region and hue and w(Cr_2_O_3_ + Fe_2_O_3_). (**d**) The larger the area of the 660-nm absorption peak, the greater the chroma of chrysoprase. (**e**) Relationship between the 660-nm peak area and chroma and w(NiO)%. The chroma and Ni content of chrysoprase increase with increase of the 660-nm peak area. (**f**) Relationship between the sum of absorption peaks at 380, 660 nm and lightness and w(Cr_2_O_3_). (**g**) The larger the area of the 660-nm absorption peak, the smaller the colourimetric coordinates a* value. (**h**) With the increase of the wavelength corresponding to the lowest absorption value in the blue–green region of the spectrum, the colourimetric coordinates b* value increases.

The UV–Vis spectra of chrysoprase are shown in Fig. 4a. All the spectra are consistent with the presence of octahedrally coordinated Ni^2+^ ions and agree with the spectra of other Ni^2+^-containing minerals in the literature^2,32–34^. The bands near 380 and 660 nm correspond to spin-allowed d-d transitions ^3^A_2g_(F) → ^3^T_1g_(P) and ^3^A_2g_(F) → ^3^T_1g_(F). The shoulders near 450 and 740 nm correspond to spin-forbidden d-d transitions ^3^A_2g_(F) → ^1^T_2g_(D) and ^3^A_2g_(F) → ^1^E_g_(D) ^34,35^.

The transmission window that occurs between the two main bands of chrysoprase centred at 380 and 660 nm is responsible for the hue of the samples (Fig. 4b). Upon changing the wavelength corresponding to the lowest absorption value in the blue–green region of the spectrum, the hue angle of chrysoprase also changes. When the wavelength decreases, the hue angle increases and the hue shifts to bluish green; when the wavelength increases, the hue angle decreases and the hue shifts to yellowish green.

The presence of Cr^3+^ and Fe^3+^ in chrysoprase results in its yellowish hue, this being because the absorptions related to the spin-allowed d-d transitions ^4^A_2g_(F) → ^4^T_1g_(F), ^4^T_1g_(P) caused by Cr^3+^ and the charge transfer O^2−^ → Fe^3+^ overlap in the short-wave part of the spectrum. Consequently, the wavelength corresponding to the lowest absorption value in the blue–green region of the spectrum shifts to the short-wave direction with an increasing sum of Cr and Fe contents. As shown by Fig. 4c, the colour change exhibits decreasing hue angle, and the hue tends to yellowish green, which is coincident with the former conclusion that the sum of Cr and Fe contents is related negatively to the hue angle.

Sachanbiński et al.^5^ reasoned that λ-dependent light scattering on micro defects in the chalcedony matrix (e.g. silica globules, nano- and micrometre-size mineral inclusions and gas–liquid inclusions) would lead to a decrease in the absorption within the short-wave part of the spectrum so that the wavelength corresponding to the lowest absorption value in the blue–green region of the spectrum shifted to the short-wave direction. The colour change was characterized by increasing hue angle, and the hue tended to bluish green.

There is a highly positive correlation between the area of the 660-nm absorption peak and the colour of chrysoprase, and the Pearson correlation coefficient *r* is 0.889. Figure 4d shows directly that the larger the area of 660-nm absorption peak, the greater the chroma of chrysoprase. The absorption at 660 nm is caused by the d-d electron transition of Ni^2+^. As shown in Fig. 4e, increasing Ni content leads to increased area of the absorption peak at 660 nm, so the chrysoprase colour will also change, as shown by the increasing chroma. This is consistent with the conclusion that there is high positive correlation between w(NiO) and the chroma C* of chrysoprase as obtained by EDXRF analysis.

The lightness of chrysoprase is negatively correlated with the sum of absorption peaks at 380 and 660 nm. As shown in Fig. 4f, the greater the sum of absorption peaks of 380 and 660 nm is, the darker the colour of chrysoprase is. This is because in the visible light band of 400 nm-700 nm, the more energy absorbed by 380 and 660 nm, the less energy left, which leads to the lower lightness of chrysoprase. Besides, as shown in Fig. 4f, Cr^3+^ will enhance the sum of absorption peaks at 380 and 660 nm, thus reducing the lightness of chrysoprase. This is consistent with the conclusion that there is a moderate negative correlation between w(Cr_2_O_3_) and lightness L^*^ of chrysoprase as obtained by EDXRF analysis.

Since there is a high negative correlation between the colourimetric coordinate a* and its chroma C*, the larger the area of 660-nm absorption peak, the smaller the colourimetric coordinates a* value (Fig. 4g). There is also a high negative correlation between the colourimetric coordinate b* and its hue angle *h*°, so with the increase of the wavelength corresponding to the lowest absorption value in the blue–green region of the spectrum, the colourimetric coordinates b* value increases (Fig. 4h).

### Crystallinity analysis

Crystallinity can refer to the relative ratio of amorphous body to crystal in a substance, as well as the degree of regular arrangement of internal particles and the number of structural defects^36^. The crystallinity referred to herein is the latter type. Gawel et al.^6^ reasoned that if the crystallinity of silica is lower, then there will be more microstructure defects, which can hold more Ni-containing compounds and affect the colour of chrysoprase. In the present study, to analyse how chrysoprase crystallinity affects colour, the crystallinity of the chrysoprase samples is described semi-quantitatively by means of non-destructive Raman spectroscopy.

**Figure 5:**
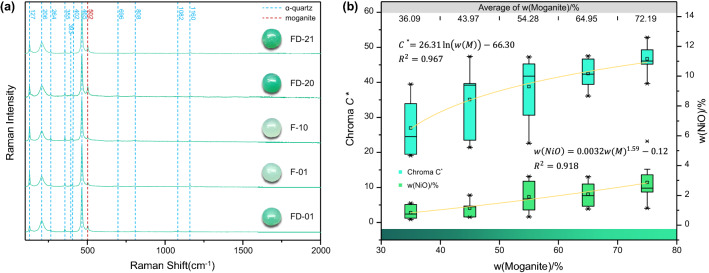
Raman spectra of chrysoprase. (**a**) All samples show standard Raman peaks of α-quartz and the characteristic peak of moganite of 502 cm^−1^. (**b**) Relationship between w(Moganite) and chroma and w(NiO). The chroma and Ni content of chrysoprase both increase with increasing moganite content.

The Raman spectra of the chrysoprase samples are shown in Fig. 5a, and all the samples show standard Raman peaks of α-quartz^37^. As well as α-quartz, most of the samples have the characteristic 502-cm^−1^ peak of moganite, which is related mainly to the symmetrical stretching bending vibration of Si–O–Si in the tetrahedral ring of silica tetrahedron^37^. As a homomorphic variant of quartz, moganite is often symbiotic with polycrystalline quartz, and according to previous studies, its relative content is related negatively to the crystallinity of polycrystalline quartz^36,38^, which can be explained from the perspective of crystal structure. The crystal structure of moganite has been described as monoclinic with alternate stacking of layers of $\left( {10\overline{1}1} \right)$ slices of left- and right-handed α-quartz corresponding to a periodic Brazil-law twinning on the unit-cell scale^39^. However, most of the quartz without double crystals in nature is composed of left- or right-handed α-quartz. It is inferred that the appearance of moganite microcrystals in α-quartz can be regarded as a structural defect, which causes the crystallinity of polycrystalline quartz mainly composed of α-quartz reduced^36^.

Götze et al.^40^ established a model for calculating the content of moganite in a sample using α-quartz as the internal standard. The Raman peaks of different ratios of α-quartz and moganite were measured under different conditions, and the Raman band integral ratio of moganite at 502 cm^−1^ and α-quartz at 465 cm^−1^ were calculated. After description and calibration, the function fitting curve of moganite content and Raman band integral ratio I_(502)_/I_(465)_ is obtained, so the moganite content of the samples can be calculated by substituting the Raman band integral ratio I_(502)_/I_(465)_ into the curve. Herein, the Raman peaks located at 502 and 465 cm^−1^ are fitted by a Gaussian Lorentzian function using the LabSpec software (version 6; Horiba Ltd., Japan), and as a result, the integral ratio of the Raman band is obtained. The calculation results are substituted into the function fitting curve of moganite content and Raman band integral ratio I_(502)_/I_(465)_ to calculate the moganite content of the samples. The content of moganite is correlated negatively with the crystallinity of polycrystalline quartz, thus the relative degree of the crystallinity of the samples is known.

The moganite content of the 41 chrysoprase samples used in this experiment varies from 20 to 80 wt%. As shown in Fig. 5b, the chroma and Ni content of chrysoprase both increase with increasing moganite content. The higher the content of moganite, the lower the crystallinity of silica, and the lower the crystallinity of silica, the more nickel. Increasing Ni content leads to increasing chroma of chrysoprase.

### Transmission electron microscopy analysis

According to earlier studies on the mineralogical and colour properties of chrysoprase, Rossman^7^ concluded that the colour of chrysoprase arises from Ni-bearing inclusions in the silica matrix rather than from substitutional Ni in the silica itself. Zbigniew Sojka et al.^41^ proved that the predominant nickel ions are located in dispersed phyllosilicates similar to that of talc, whereas only less abundant nickel ions are grafted on the surface of the chalcedony matrix by the analysis of the TPR, EPR and UV–Vis results. To easily observe the Ni-bearing inclusions in chrysoprase under the transmission electron microscope, the sample FI-01 with the highest Ni content and the highest chroma was selected for transmission electron microscope observation and energy-dispersive spectrometer analysis.

**Figure 6:**
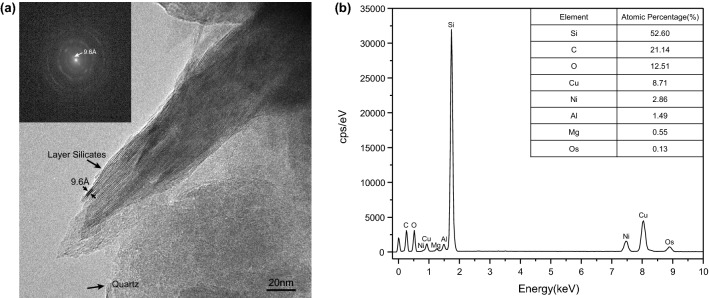
Results of TEM observations. (**a**) In chrysoprase, nanometre-size layer silicates with interlayer distances of 9.6 Å are found frequently in the quartz crystals and at their boundaries. The diffuse and weak ring-shaped selected area electron diffraction (SAED) patterns of layer silicates, in which strong reflections 9.6 Å are observed, are shown in the upper left corner of the figure. (**b**) The result of energy-dispersive spectrometer analysis.

Under TEM, layer silicates as shown in Fig. 6a are observed frequently in the quartz crystals and at their boundaries. They commonly range in thickness from 5 to 50 nm, but they are much longer than that and continue from one grain boundary to the next. The diffuse and weak ring-shaped selected area electron diffraction (SAED) patterns of layer silicates, in which strong reflections 9.6 Å are observed(calculated with DigitalMicrograph3 software), are shown in the upper left corner of Fig. 6a. The ring-shaped selected-area electron diffraction (SAED) patterns and the lattice fringe of 9.6 Å spacing shown in Fig. 6a indicate that the layer silicate is of the kerolite–pimelite series^35,42^. Pelletier^43^ distinguished the electron diffraction pattern of the talc-willemseite, with a regular structure, from the kerolite–pimelite series, showing concentric circles. Furthermore, Cristina Villanova-de-Benavent et al.^44^ also reported that kerolite–pimelite series produced ring-shaped selected-area electron diffraction (SAED) patterns. The observations in this study are also identical to the kerolite–pimelite series observed by other investigators under TEM^5,8,45^.

Kerolite-pimelite is solid solution series with Mg and Ni as two end-members, i.e. Mg-rich kerolite to Ni-rich pimelite, which can be described as phases with talc affinity and extra water in their structure, within the ideal structural formula (Mg,Ni)_3_Si_4_O_10_(OH)_2_·n(H_2_O)^44,46^. Interference from the surrounding silica grains meant that it was impossible to obtain accurate chemical analyses of the tiny kerolite–pimelite series in the present experiment, but an energy-dispersive spectrometer was used to record some chemical-element information about the kerolite–pimelite series at the boundaries of the quartz grains. The kerolite–pimelite series in the area of Fig. 6a was analyzed by the energy-dispersive spectrometer, and EDS spectra were shown in Fig. 6b. Cu, C are elements of the Cu grid, which can be ignored. In addition to Si and O, Ni and a small amount of Mg were detected, indicating that the Ni content in this kerolite–pimelite series is higher than that of Mg, so the inclusions observed under TEM that cause the chrysoprase colour are pimelite.

Since the predominant nickel ions are located in dispersed phyllosilicates^41^, the relationship between the content of pimelite and the colour and crystallinity of chrysoprase is the same as that between Ni content and these factors.

## Conclusion

Under a 6504-K fluorescent lamp, the colour of chrysoprase was measured and divided into the grades of fancy, fancy intense and fancy deep. There was moderate negative correlation between the lightness L* and the Cr content, high positive correlation between the chroma C* and the Ni content, and moderate negative correlation between the hue angle *h*° and the sum of the Fe and Cr contents.

The wavelength corresponding to the lowest absorption value in the blue–green region of the spectrum shifted to the short-wave direction with increasing sum of the Cr and Fe contents. The colour change exhibited decreasing hue angle, and the hue tended to yellowish green. The absorption peak at 660 nm was caused by the d-d transition of Ni^2+^, and increasing Ni content led to increasing area of the absorption peak at 660 nm in the UV–Vis spectrum, which was manifested as increasing chroma of chrysoprase. The lightness of chrysoprase is negatively correlated with the sum of absorption peaks at 380 and 660 nm. Cr^3+^ will enhance the sum of absorption peaks at 380 and 660 nm, thus reducing the lightness of chrysoprase. The larger the area of 660-nm absorption peak, the smaller the colourimetric coordinates a* value. With the increase of the wavelength corresponding to the lowest absorption value in the blue–green region of the spectrum, the colourimetric coordinates b* value increases.

With decreasing crystallinity of chrysoprase, its Ni content and chroma increase. Chrysoprase with low crystallinity contains more nickel. The higher the Ni content, the stronger the chrysoprase colour, which shows the increase of chroma.

The inclusions that cause the chrysoprase colour have been identified as pimelite from the present results of TEM and EDS investigations.

## Methods

### Transmission electron microscopy

The chrysoprase sample was ground into a thin slice with a thickness of 30 μm with sandpaper. The resulting slice was prepared into serial of ultrathin Sects. (70 nm) using a Power-Town XL Ultramicrotome. Ultrathin sections were placed on a holey carbon thin-film supported by a Cu grid. A JEOL JEM F200 type transmission electron microscope (TEM) equipped with Oxford 65 T energy dispersive spectrometer (EDS) was used for this study. All TEM observations were carried out at 200 kV operating voltage.

### Colourimetric analysis

Chrysoprase colour was quantified by MDIS-f8 multifunction dual integrating sphere spectrometer, which collects reflective signals on the chrysoprase surface. The test conditions were described as follows: the range of wavelength, 380–760 nm; Spectral resolution: 1 nm; CIE standard illumination, D65; Thermal stable temperature, 30 ℃; voltage of 220v and frequency of 50–60 Hz.

### EDXRF

Micro-area chemical components were measured by an EDX-7000 Energy Dispersive X-ray fluorescence spectrometer with the test conditions as follows: atmosphere, oxide; voltage of 50 kV;108 μA; 30% DT; collimator, 5 mm.

### UV–vis spectra

Absorption spectra and transmission spectra in the ultraviolet to visible (UV–Vis) range were recorded with a UV-3600 UV–VIS spectrophotometer. The test conditions were described as follows: the range of wavelength, 200–1000 nm; scanning speed, high; sampling interval, 1.0 s; scanning mode, single.

### Raman spectra

The Raman spectra were measured with Horiba LabRAM HR Evolution Raman spectrometer equipped with a Peltier-cooled charged-coupled device(CCD) detector, edge filters, and a Nd-YAG laser. The test conditions were described as follows: the range of wavelength, 200–2000 cm^−1^; acq.time, 3.0 s; accumulations, 1; autofocus, off; autoexposure, off; spike flitter, multiple accum; Delay time, 0.0 s; binning, 1; readout mode, signal; denoise, lite; ICS correction, On; dark correction, off; inst. Process,off; objective, x50_VIS_LWD; grating 1800(500 nm); ND filter, 100%; laser, 532nm_Edge.

## Supplementary Information

Supplementary Information 1.

## Data Availability

The dataset for this study is available from the corresponding author upon reasonable request.
