# Supplementary material for: Genesis and influencing factors of the colour of chrysoprase
Source: Sci Rep. 2021 May 11;11:9939. doi: 10.1038/s41598-021-89406-x (PMC8113324; doi:10.1038/s41598-021-89406-x)
Supplement: Supplementary file 1 — Supplementary Information 1. [file 41598_2021_89406_MOESM1_ESM.docx]

**Genesis and influencing factors of the colour of chrysoprase**

Yuansheng Jiang^1^ & Ying Guo^2&(Corresponding Author)^

^1^ Department of Gemmology, China University of Geosciences (Beijing), 100083, China. E-mail:jiangyuansheng1@126.com

^2^ Department of Gemmology, China University of Geosciences (Beijing), 100083, China. E-mail:guoying@cugb.edu.cn

|  |  | 1 | 2 | 3 | total |
| --- | --- | --- | --- | --- | --- |
| count | 1 | 11 | 0 | 0 | 11 |
|  | 2 | 0 | 21 | 1 | 22 |
|  | 3 | 0 | 1 | 7 | 8 |
| % | 1 | 100 | 0 | 0 | 100 |
|  | 2 | 0 | 95.5 | 4.5 | 100 |
|  | 3 | 0 | 12.5 | 87.5 | 100 |
| 95.12% of the original group observations were classified correctly. | | | | | |

Fisher discriminant accuracy(table 1)

| number | *L** | *a** | *b** | *C** | *h*° | classification |
| --- | --- | --- | --- | --- | --- | --- |
| F-01 | 58.38 | -23.04 | 4.43 | 23.46202 | 169.12 | 1 |
| F-02 | 54.26 | -22.24 | 3.95 | 22.58805 | 169.93 | 1 |
| F-03 | 52.87 | -29.59 | 7.78 | 30.59569 | 165.27 | 1 |
| F-04 | 50.68 | -27.65 | 7.93 | 28.76469 | 164.00 | 1 |
| F-05 | 48.73 | -33.46 | 5.55 | 33.91717 | 170.58 | 1 |
| F-06 | 56.79 | -24.05 | 4.67 | 24.49921 | 169.01 | 1 |
| F-07 | 57.54 | -21.33 | 1.87 | 21.41181 | 174.99 | 1 |
| F-08 | 63.99 | -28.96 | 6.85 | 29.7591 | 166.69 | 1 |
| F-09 | 53.02 | -19.11 | 3.75 | 19.47446 | 168.90 | 1 |
| F-10 | 56.51 | -21.69 | 6 | 22.50458 | 164.54 | 1 |
| F-11 | 42.21 | -18.81 | 3.03 | 19.05248 | 170.85 | 1 |
| FD-01 | 51.58 | -36.93 | 12.12 | 38.86797 | 161.83 | 2 |
| FD-02 | 43.65 | -40.34 | 14.92 | 43.01072 | 159.70 | 2 |
| FD-03 | 47.81 | -36.69 | 14.58 | 39.48079 | 158.33 | 2 |
| FD-04 | 41.83 | -37.9 | 21.13 | 43.39224 | 150.86 | 2 |
| FD-05 | 49.75 | -35.02 | 8.65 | 36.07247 | 166.13 | 2 |
| FD-06 | 44.82 | -42.29 | 16.07 | 45.24035 | 159.19 | 2 |
| FD-07 | 50.22 | -39.07 | 12.35 | 40.97545 | 162.46 | 2 |
| FD-08 | 47.25 | -38.17 | 9.81 | 39.41047 | 165.59 | 2 |
| FD-09 | 44.94 | -44.28 | 16.13 | 47.12638 | 159.98 | 2 |
| FD-10 | 45.76 | -43.21 | 13.71 | 45.33286 | 162.40 | 2 |
| FD-11 | 41.07 | -43.21 | 19.16 | 47.26743 | 156.09 | 2 |
| FD-12 | 45.4 | -43.81 | 13.71 | 45.90512 | 162.62 | 2 |
| FD-13 | 41.02 | -41.51 | 17.43 | 45.02094 | 157.22 | 2 |
| FD-14 | 41.73 | -39.64 | 21.29 | 44.99549 | 151.76 | 2 |
| FD-15 | 49.94 | -31.61 | 20.3 | 37.56703 | 147.29 | 2 |
| FD-16 | 53.6 | -37.58 | 12.6 | 39.63605 | 161.46 | 2 |
| FD-17 | 50.43 | -37.67 | 11.79 | 39.47193 | 162.62 | 2 |
| FD-18 | 49.89 | -39.89 | 10.37 | 41.21588 | 165.43 | 2 |
| FD-19 | 39.39 | -37.8 | 18.95 | 42.28407 | 153.37 | 2 |
| FD-20 | 46.73 | -43.2 | 13.86 | 45.36893 | 162.21 | 2 |
| FD-21 | 47.53 | -38.54 | 14.33 | 41.11789 | 159.60 | 2 |
| FD-22 | 46.92 | -38.16 | 15.83 | 41.31313 | 157.47 | 2 |
| FI-01 | 52.57 | -51.71 | 10.76 | 52.81763 | 168.25 | 3 |
| FI-02 | 49.77 | -47.46 | 9.52 | 48.40539 | 168.66 | 3 |
| FI-03 | 47.14 | -48.33 | 13.15 | 50.08704 | 164.78 | 3 |
| FI-04 | 52.22 | -46.32 | 10.68 | 47.5353 | 167.02 | 3 |
| FI-05 | 50.13 | -44.81 | 11.37 | 46.23 | 165.76 | 3 |
| FI-06 | 48.28 | -44.31 | 14.62 | 46.65962 | 161.74 | 3 |
| FI-07 | 52.37 | -45.34 | 13.8 | 47.39362 | 163.07 | 3 |
| FI-08 | 66.1 | -37.98 | 11.28 | 39.61968 | 163.46 | 3 |

Colour grading of chrysoprase(date of figure 2)

| Number | NiO | SiO2 | SO3 | CaO | Fe2O3 | K2O | Cr2O3 | ZnO | WO3 | Sc2O3 | Ag2O | Co2O3 | Ta2O5 | PtO2 | Cs2O | CuO | BaO | Ho2O3 | Ir2O3 | Tb4O7 | Gd2O3 | Sm2O3 | MnO | GeO2 | SeO2 |
| --- | --- | --- | --- | --- | --- | --- | --- | --- | --- | --- | --- | --- | --- | --- | --- | --- | --- | --- | --- | --- | --- | --- | --- | --- | --- |
| FD-01 | 1.192 | 98.402 | 0.333 | 0.009 | 0.007 | 0.024 | 0.019 | 0.004 | 0.01 | 0 | 0 | 0 | 0 | 0 | 0 | 0 | 0 | 0 | 0 | 0 | 0 | 0 | 0 | 0 | 0 |
| FD-02 | 2.525 | 96.946 | 0.426 | 0.021 | 0.002 | 0.039 | 0.027 | 0.007 | 0 | 0 | 0.007 | 0 | 0 | 0 | 0 | 0 | 0 | 0 | 0 | 0 | 0 | 0 | 0 | 0 | 0 |
| FI-01 | 5.631 | 92.416 | 0.423 | 1.427 | 0.012 | 0.068 | 0.01 | 0.004 | 0 | 0 | 0 | 0.009 | 0 | 0 | 0 | 0 | 0 | 0 | 0 | 0 | 0 | 0 | 0 | 0 | 0 |
| FD-03 | 1.267 | 98.23 | 0.424 | 0 | 0.007 | 0.025 | 0.017 | 0.003 | 0.007 | 0 | 0.008 | 0 | 0.012 | 0 | 0 | 0 | 0 | 0 | 0 | 0 | 0 | 0 | 0 | 0 | 0 |
| FI-02 | 2.345 | 97.203 | 0.373 | 0.017 | 0.002 | 0.029 | 0.013 | 0.006 | 0 | 0.007 | 0 | 0 | 0 | 0.005 | 0 | 0 | 0 | 0 | 0 | 0 | 0 | 0 | 0 | 0 | 0 |
| FD-04 | 2.292 | 97.215 | 0.373 | 0.017 | 0.009 | 0.041 | 0.033 | 0.002 | 0 | 0 | 0.004 | 0 | 0 | 0 | 0 | 0 | 0 | 0 | 0 | 0.014 | 0 | 0 | 0 | 0 | 0 |
| FI-03 | 3.752 | 95.702 | 0.401 | 0.036 | 0.01 | 0.053 | 0.027 | 0.004 | 0 | 0.016 | 0 | 0 | 0 | 0 | 0 | 0 | 0 | 0 | 0 | 0 | 0 | 0 | 0 | 0 | 0 |
| FD-05 | 1.082 | 98.461 | 0.39 | 0.011 | 0.008 | 0.023 | 0.013 | 0.003 | 0.009 | 0 | 0 | 0 | 0 | 0 | 0 | 0 | 0 | 0 | 0 | 0 | 0 | 0 | 0 | 0 | 0 |
| FI-04 | 3.25 | 96.262 | 0.355 | 0.06 | 0.002 | 0.037 | 0.008 | 0.003 | 0 | 0 | 0.007 | 0.005 | 0 | 0 | 0 | 0 | 0 | 0 | 0 | 0 | 0 | 0 | 0.012 | 0 | 0 |
| FD-06 | 2.972 | 96.602 | 0.3 | 0.031 | 0.01 | 0.059 | 0.023 | 0.003 | 0 | 0 | 0 | 0 | 0 | 0 | 0 | 0 | 0 | 0 | 0 | 0 | 0 | 0 | 0 | 0 | 0 |
| F-01 | 0.534 | 99.099 | 0.299 | 0.008 | 0.008 | 0.026 | 0.01 | 0.003 | 0 | 0 | 0 | 0 | 0 | 0.005 | 0 | 0.007 | 0 | 0 | 0 | 0 | 0 | 0 | 0 | 0.002 | 0 |
| F-02 | 0.55 | 98.968 | 0.38 | 0.012 | 0.009 | 0.049 | 0.011 | 0.003 | 0.011 | 0.005 | 0 | 0 | 0 | 0 | 0 | 0 | 0 | 0 | 0 | 0 | 0 | 0 | 0 | 0 | 0.002 |
| F-03 | 0.759 | 98.693 | 0.47 | 0.012 | 0.008 | 0.036 | 0.01 | 0 | 0.012 | 0 | 0 | 0 | 0 | 0 | 0 | 0 | 0 | 0 | 0 | 0 | 0 | 0 | 0 | 0 | 0 |
| FD-07 | 1.275 | 98.135 | 0.352 | 0.162 | 0.008 | 0.045 | 0.015 | 0.003 | 0 | 0 | 0.006 | 0 | 0 | 0 | 0 | 0 | 0 | 0 | 0 | 0 | 0 | 0 | 0 | 0 | 0 |
| FD-08 | 1.66 | 97.814 | 0.424 | 0.014 | 0.011 | 0.059 | 0.016 | 0.002 | 0 | 0 | 0 | 0 | 0 | 0 | 0 | 0 | 0 | 0 | 0 | 0 | 0 | 0 | 0 | 0 | 0 |
| FD-09 | 2.958 | 95.793 | 0.401 | 0.742 | 0.011 | 0.061 | 0.029 | 0.006 | 0 | 0 | 0 | 0 | 0 | 0 | 0 | 0 | 0 | 0 | 0 | 0 | 0 | 0 | 0 | 0 | 0 |
| FD-10 | 2.299 | 97.244 | 0.36 | 0.017 | 0.001 | 0.037 | 0.022 | 0.005 | 0.008 | 0.007 | 0 | 0 | 0 | 0 | 0 | 0 | 0 | 0 | 0 | 0 | 0 | 0 | 0 | 0 | 0 |
| FD-11 | 3.27 | 96.191 | 0.377 | 0.027 | 0.025 | 0.058 | 0.03 | 0.014 | 0 | 0 | 0 | 0.007 | 0 | 0 | 0 | 0 | 0 | 0 | 0 | 0 | 0 | 0 | 0 | 0 | 0 |
| FD-12 | 2.619 | 96.829 | 0.398 | 0.025 | 0.01 | 0.077 | 0.021 | 0.006 | 0 | 0 | 0.011 | 0 | 0 | 0.005 | 0 | 0 | 0 | 0 | 0 | 0 | 0 | 0 | 0 | 0 | 0 |
| FD-13 | 2.752 | 96.715 | 0.412 | 0.027 | 0.002 | 0.039 | 0.035 | 0.005 | 0 | 0.007 | 0.006 | 0 | 0 | 0 | 0 | 0 | 0 | 0 | 0 | 0 | 0 | 0 | 0 | 0 | 0 |
| FD-14 | 3.007 | 96.38 | 0.435 | 0.026 | 0.011 | 0.052 | 0.035 | 0.006 | 0.012 | 0 | 0 | 0 | 0 | 0 | 0 | 0 | 0.036 | 0 | 0 | 0 | 0 | 0 | 0 | 0 | 0 |
| FI-05 | 2.156 | 97.329 | 0.415 | 0.019 | 0.01 | 0.024 | 0.018 | 0.003 | 0.012 | 0 | 0.014 | 0 | 0 | 0 | 0 | 0 | 0 | 0 | 0 | 0 | 0 | 0 | 0 | 0 | 0 |
| F-04 | 1.023 | 98.484 | 0.368 | 0.031 | 0.012 | 0.038 | 0.014 | 0.006 | 0.011 | 0.012 | 0 | 0 | 0 | 0 | 0 | 0 | 0 | 0 | 0 | 0 | 0 | 0 | 0 | 0 | 0 |
| F-05 | 1.377 | 98.122 | 0.379 | 0.021 | 0.01 | 0.025 | 0.015 | 0.006 | 0 | 0 | 0 | 0 | 0 | 0 | 0.045 | 0 | 0 | 0 | 0 | 0 | 0 | 0 | 0 | 0 | 0 |
| FD-15 | 1.197 | 98.306 | 0.371 | 0.028 | 0.022 | 0.048 | 0.015 | 0.005 | 0.009 | 0 | 0 | 0 | 0 | 0 | 0 | 0 | 0 | 0 | 0 | 0 | 0 | 0 | 0 | 0 | 0 |
| F-06 | 0.748 | 98.685 | 0.391 | 0.021 | 0.01 | 0.049 | 0.013 | 0.004 | 0.009 | 0.013 | 0 | 0 | 0.014 | 0 | 0.042 | 0 | 0 | 0 | 0 | 0 | 0 | 0 | 0 | 0 | 0 |
| FI-06 | 2.762 | 96.687 | 0.419 | 0.048 | 0.005 | 0.038 | 0.025 | 0.004 | 0.008 | 0 | 0.005 | 0 | 0 | 0 | 0 | 0 | 0 | 0 | 0 | 0 | 0 | 0 | 0 | 0 | 0 |
| FD-16 | 1.286 | 98.408 | 0.233 | 0.026 | 0.01 | 0 | 0.019 | 0 | 0.011 | 0.008 | 0 | 0 | 0 | 0 | 0 | 0 | 0 | 0 | 0 | 0 | 0 | 0 | 0 | 0 | 0 |
| FD-17 | 1.482 | 98.005 | 0.397 | 0.032 | 0 | 0.021 | 0.026 | 0 | 0.012 | 0.01 | 0 | 0 | 0.014 | 0 | 0 | 0 | 0 | 0 | 0 | 0 | 0 | 0 | 0 | 0 | 0 |
| F-07 | 0.551 | 98.937 | 0.395 | 0.052 | 0.009 | 0.035 | 0.012 | 0.003 | 0 | 0 | 0 | 0 | 0 | 0.006 | 0 | 0 | 0 | 0 | 0 | 0 | 0 | 0 | 0 | 0 | 0 |
| FD-18 | 1.716 | 97.794 | 0.39 | 0.014 | 0.013 | 0.051 | 0.018 | 0.005 | 0 | 0 | 0 | 0 | 0 | 0 | 0 | 0 | 0 | 0 | 0 | 0 | 0 | 0 | 0 | 0 | 0 |
| F-08 | 0.884 | 98.601 | 0.398 | 0.021 | 0.008 | 0.029 | 0.014 | 0.011 | 0.013 | 0 | 0 | 0.003 | 0 | 0 | 0 | 0 | 0 | 0 | 0.005 | 0.013 | 0 | 0 | 0 | 0 | 0 |
| F-09 | 0.49 | 99.066 | 0.336 | 0.013 | 0.01 | 0.051 | 0.014 | 0.002 | 0 | 0 | 0 | 0 | 0 | 0 | 0 | 0.009 | 0 | 0 | 0 | 0 | 0 | 0.009 | 0 | 0 | 0 |
| FI-07 | 2.016 | 97.406 | 0.46 | 0.027 | 0.011 | 0.026 | 0.019 | 0.018 | 0.012 | 0 | 0 | 0.003 | 0 | 0 | 0 | 0 | 0 | 0 | 0 | 0 | 0 | 0 | 0 | 0 | 0 |
| FI-08 | 1.127 | 98.356 | 0.411 | 0.025 | 0.011 | 0.03 | 0.014 | 0.006 | 0.012 | 0.008 | 0 | 0 | 0 | 0 | 0 | 0 | 0 | 0 | 0 | 0 | 0 | 0 | 0 | 0 | 0 |
| FD-19 | 2.949 | 96.732 | 0.217 | 0.023 | 0.002 | 0.026 | 0.032 | 0.008 | 0.011 | 0 | 0 | 0 | 0 | 0 | 0 | 0 | 0 | 0 | 0 | 0 | 0 | 0 | 0 | 0 | 0 |
| F-10 | 0.38 | 99.08 | 0.412 | 0.022 | 0.011 | 0.063 | 0.014 | 0.003 | 0 | 0.009 | 0 | 0 | 0 | 0.006 | 0 | 0 | 0 | 0 | 0 | 0 | 0 | 0 | 0 | 0 | 0 |
| FD-20 | 1.799 | 97.671 | 0.456 | 0.008 | 0.01 | 0.037 | 0.014 | 0 | 0.006 | 0 | 0 | 0 | 0 | 0 | 0 | 0 | 0 | 0 | 0 | 0 | 0 | 0 | 0 | 0 | 0 |
| FD-21 | 1.443 | 98.014 | 0.442 | 0.016 | 0.012 | 0.026 | 0.022 | 0 | 0.015 | 0.01 | 0 | 0 | 0 | 0 | 0 | 0 | 0 | 0 | 0 | 0 | 0 | 0 | 0 | 0 | 0 |
| FD-22 | 1.536 | 98.035 | 0.327 | 0.01 | 0.008 | 0.05 | 0.018 | 0.004 | 0.01 | 0 | 0 | 0 | 0 | 0 | 0 | 0 | 0 | 0 | 0 | 0 | 0 | 0 | 0 | 0 | 0 |
| F-11 | 0.47 | 98.965 | 0.411 | 0.035 | 0.013 | 0.069 | 0.012 | 0 | 0.01 | 0 | 0 | 0 | 0 | 0 | 0 | 0.012 | 0 | 0 | 0 | 0 | 0 | 0 | 0 | 0 | 0 |

EDXRF data of chrysoprase samples(table S1)

| Number | NiO | Cr2O3 | Fe2O3 | Cr+Fe | *L** | *C** | *h*° |
| --- | --- | --- | --- | --- | --- | --- | --- |
| FD-01 | 1.192 | 0.019 | 0.007 | 0.026 | 51.58 | 38.868 | 161.83 |
| FD-02 | 2.525 | 0.027 | 0.002 | 0.029 | 43.65 | 43.0107 | 159.70 |
| FI-01 | 5.631 | 0.01 | 0.012 | 0.022 | 52.57 | 52.8176 | 168.25 |
| FD-03 | 1.267 | 0.017 | 0.007 | 0.024 | 47.81 | 39.4808 | 158.33 |
| FI-02 | 2.345 | 0.013 | 0.002 | 0.015 | 49.77 | 48.4054 | 168.66 |
| FD-04 | 2.292 | 0.033 | 0.009 | 0.042 | 41.83 | 43.3922 | 150.86 |
| FI-03 | 3.752 | 0.027 | 0.01 | 0.037 | 47.14 | 50.087 | 164.78 |
| FD-05 | 1.082 | 0.013 | 0.008 | 0.021 | 49.75 | 36.0725 | 166.13 |
| FI-04 | 3.25 | 0.008 | 0.002 | 0.01 | 52.22 | 47.5353 | 167.02 |
| FD-06 | 2.972 | 0.023 | 0.01 | 0.033 | 44.82 | 45.2403 | 159.19 |
| F-01 | 0.534 | 0.01 | 0.008 | 0.018 | 58.38 | 23.462 | 169.12 |
| F-02 | 0.55 | 0.011 | 0.009 | 0.02 | 54.26 | 22.5881 | 169.93 |
| F-03 | 0.759 | 0.01 | 0.008 | 0.018 | 52.87 | 30.5957 | 165.27 |
| FD-07 | 1.275 | 0.015 | 0.008 | 0.023 | 50.22 | 40.9754 | 162.46 |
| FD-08 | 1.66 | 0.016 | 0.011 | 0.027 | 47.25 | 39.4105 | 165.59 |
| FD-09 | 2.958 | 0.029 | 0.011 | 0.04 | 44.94 | 47.1264 | 159.98 |
| FD-10 | 2.299 | 0.022 | 0.001 | 0.023 | 45.76 | 45.3329 | 162.40 |
| FD-11 | 3.27 | 0.03 | 0.025 | 0.055 | 41.07 | 47.2674 | 156.09 |
| FD-12 | 2.619 | 0.021 | 0.01 | 0.031 | 45.4 | 45.9051 | 162.62 |
| FD-13 | 2.752 | 0.035 | 0.002 | 0.037 | 41.02 | 45.0209 | 157.22 |
| FD-14 | 3.007 | 0.035 | 0.011 | 0.046 | 41.73 | 44.9955 | 151.76 |
| FI-05 | 2.156 | 0.018 | 0.01 | 0.028 | 50.13 | 46.23 | 165.76 |
| F-04 | 1.023 | 0.014 | 0.012 | 0.026 | 50.68 | 28.7647 | 164.00 |
| F-05 | 1.377 | 0.015 | 0.01 | 0.025 | 48.73 | 33.9172 | 170.58 |
| FD-15 | 1.197 | 0.015 | 0.022 | 0.037 | 49.94 | 37.567 | 147.29 |
| F-06 | 0.748 | 0.013 | 0.01 | 0.023 | 56.79 | 24.4992 | 169.01 |
| FI-06 | 2.762 | 0.025 | 0.005 | 0.03 | 48.28 | 46.6596 | 161.74 |
| FD-16 | 1.286 | 0.019 | 0.01 | 0.029 | 53.6 | 39.636 | 161.46 |
| FD-17 | 1.482 | 0.026 | 0 | 0.026 | 50.43 | 39.4719 | 162.62 |
| F-07 | 0.551 | 0.012 | 0.009 | 0.021 | 57.54 | 21.4118 | 174.99 |
| FD-18 | 1.716 | 0.018 | 0.013 | 0.031 | 49.89 | 41.2159 | 165.43 |
| F-08 | 0.884 | 0.014 | 0.008 | 0.022 | 63.99 | 29.7591 | 166.69 |
| F-09 | 0.49 | 0.014 | 0.01 | 0.024 | 53.02 | 19.4745 | 168.90 |
| FI-07 | 2.016 | 0.019 | 0.011 | 0.03 | 52.37 | 47.3936 | 163.07 |
| FI-08 | 1.127 | 0.014 | 0.011 | 0.025 | 66.1 | 39.6197 | 163.46 |
| FD-19 | 2.949 | 0.032 | 0.002 | 0.034 | 39.39 | 42.2841 | 153.37 |
| F-10 | 0.38 | 0.014 | 0.011 | 0.025 | 56.51 | 22.5046 | 164.54 |
| FD-20 | 1.799 | 0.014 | 0.01 | 0.024 | 46.73 | 45.3689 | 162.21 |
| FD-21 | 1.443 | 0.022 | 0.012 | 0.034 | 47.53 | 41.1179 | 159.60 |
| FD-22 | 1.536 | 0.018 | 0.008 | 0.026 | 46.92 | 41.3131 | 157.47 |
| F-11 | 0.47 | 0.012 | 0.013 | 0.025 | 42.21 | 19.0525 | 170.85 |

Date of table 2, figure 3(a)

| categories | w(Cr2O3)% | *L** | *h*° |
| --- | --- | --- | --- |
| 0.005-0.015 | 0.008 | 52.22 | 167.02 |
|  | 0.01 | 52.57 | 168.25 |
|  | 0.01 | 58.38 | 169.12 |
|  | 0.01 | 52.87 | 165.27 |
|  | 0.011 | 54.26 | 169.93 |
|  | 0.012 | 57.54 | 174.99 |
|  | 0.012 | 42.21 | 170.85 |
|  | 0.013 | 49.77 | 168.66 |
|  | 0.013 | 49.75 | 166.13 |
|  | 0.013 | 56.79 | 169.01 |
|  | 0.014 | 50.68 | 164.00 |
|  | 0.014 | 63.99 | 166.69 |
|  | 0.014 | 53.02 | 168.90 |
|  | 0.014 | 66.1 | 163.46 |
|  | 0.014 | 56.51 | 164.54 |
|  | 0.014 | 46.73 | 162.21 |
| Mean value | 0.01225 | 53.96 | 167.44 |
| 0.015-0.025 | 0.015 | 50.22 | 162.46 |
|  | 0.015 | 48.73 | 170.58 |
|  | 0.015 | 49.94 | 147.29 |
|  | 0.016 | 47.25 | 165.59 |
|  | 0.017 | 47.81 | 158.33 |
|  | 0.018 | 50.13 | 165.76 |
|  | 0.018 | 49.89 | 165.43 |
|  | 0.018 | 46.92 | 157.47 |
|  | 0.019 | 51.58 | 161.83 |
|  | 0.019 | 53.6 | 161.46 |
|  | 0.019 | 52.37 | 163.07 |
|  | 0.021 | 45.4 | 162.62 |
|  | 0.022 | 45.76 | 162.40 |
|  | 0.022 | 47.53 | 159.60 |
|  | 0.023 | 44.82 | 159.19 |
| Mean value | 0.018466667 | 48.80 | 161.54 |
| 0.025-0.035 | 0.025 | 48.28 | 161.74 |
|  | 0.026 | 50.43 | 162.62 |
|  | 0.027 | 43.65 | 159.70 |
|  | 0.027 | 47.14 | 164.78 |
|  | 0.029 | 44.94 | 159.98 |
|  | 0.03 | 41.07 | 156.09 |
|  | 0.032 | 39.39 | 153.37 |
|  | 0.033 | 41.83 | 150.86 |
| Mean value | 0.028625 | 44.59 | 158.64 |
| 0.035-0.045 | 0.035 | 41.02 | 157.22 |
|  | 0.035 | 41.73 | 151.76 |
| Mean value | 0.035 | 41.38 | 154.49 |

With increasing Cr content, the lightness and hue angle exhibit downward trends.(figure 3b)

| categories | w(Cr+Fe)% | *h*° |
| --- | --- | --- |
| 0.01-0.02 | 0.01 | 167.02 |
|  | 0.015 | 168.66 |
|  | 0.018 | 169.12 |
|  | 0.018 | 165.27 |
| Mean value | 0.015 | 167.52 |
| 0.02-0.03 | 0.02 | 169.93 |
|  | 0.021 | 174.99 |
|  | 0.021 | 166.13 |
|  | 0.022 | 168.25 |
|  | 0.022 | 166.69 |
|  | 0.023 | 169.01 |
|  | 0.023 | 162.46 |
|  | 0.023 | 162.40 |
|  | 0.024 | 168.90 |
|  | 0.024 | 162.21 |
|  | 0.024 | 158.33 |
|  | 0.025 | 170.85 |
|  | 0.025 | 163.46 |
|  | 0.025 | 164.54 |
|  | 0.025 | 170.58 |
|  | 0.026 | 157.47 |
|  | 0.026 | 161.83 |
|  | 0.026 | 162.62 |
|  | 0.026 | 164.00 |
|  | 0.027 | 165.59 |
|  | 0.028 | 165.76 |
|  | 0.029 | 161.46 |
|  | 0.029 | 159.70 |
| Mean value | 0.025 | 165.09 |
| 0.03-0.04 | 0.03 | 163.07 |
|  | 0.03 | 161.74 |
|  | 0.031 | 165.43 |
|  | 0.031 | 162.62 |
|  | 0.033 | 159.19 |
|  | 0.034 | 159.60 |
|  | 0.034 | 153.37 |
|  | 0.037 | 147.29 |
|  | 0.037 | 164.78 |
|  | 0.037 | 157.22 |
| Mean value | 0.033 | 159.43 |
| 0.04-0.05 | 0.04 | 159.98 |
|  | 0.042 | 150.86 |
|  | 0.046 | 151.76 |
| Mean value | 0.043 | 154.20 |
| 0.05-0.06 | 0.055 | 156.09 |
| Mean value | 0.055 | 156.09 |

Both Cr and Fe affect the hue angle; the higher the content of Cr and Fe, the smaller the hue angle. (figure 3c)

| wavelength | absorbance |
| --- | --- |
| 223.933 | 0.909400842 |
| 224.724 | 0.915959896 |
| 225.516 | 0.925393074 |
| 226.307 | 0.922488015 |
| 227.098 | 0.92960526 |
| 227.889 | 0.927031207 |
| 228.68 | 0.925751436 |
| 229.471 | 0.926739008 |
| 230.262 | 0.934776828 |
| 231.053 | 0.931921932 |
| 231.844 | 0.936400367 |
| 232.635 | 0.942866009 |
| 233.425 | 0.943969716 |
| 234.216 | 0.940208516 |
| 235.007 | 0.941328409 |
| 235.797 | 0.940414825 |
| 236.588 | 0.938696389 |
| 237.378 | 0.938500196 |
| 238.168 | 0.937241429 |
| 238.959 | 0.936989765 |
| 239.749 | 0.935414086 |
| 240.539 | 0.932320914 |
| 241.329 | 0.927441638 |
| 242.119 | 0.924306411 |
| 242.909 | 0.917823021 |
| 243.699 | 0.914256208 |
| 244.489 | 0.907160033 |
| 245.279 | 0.901481053 |
| 246.068 | 0.899344616 |
| 246.858 | 0.896707058 |
| 247.648 | 0.891465138 |
| 248.437 | 0.889023171 |
| 249.227 | 0.884400311 |
| 250.016 | 0.876809161 |
| 250.805 | 0.868731719 |
| 251.595 | 0.86217321 |
| 252.384 | 0.856689471 |
| 253.173 | 0.853962195 |
| 253.962 | 0.851371843 |
| 254.751 | 0.85069893 |
| 255.54 | 0.848329075 |
| 256.329 | 0.844392173 |
| 257.118 | 0.840806429 |
| 257.907 | 0.838696352 |
| 258.695 | 0.835078526 |
| 259.484 | 0.833069233 |
| 260.273 | 0.831351795 |
| 261.061 | 0.830500639 |
| 261.85 | 0.828707427 |
| 262.638 | 0.826897304 |
| 263.426 | 0.824705141 |
| 264.215 | 0.824506632 |
| 265.003 | 0.821675161 |
| 265.791 | 0.818942155 |
| 266.579 | 0.818254577 |
| 267.367 | 0.817280487 |
| 268.155 | 0.815327851 |
| 268.943 | 0.814357373 |
| 269.731 | 0.812796175 |
| 270.519 | 0.811582212 |
| 271.306 | 0.810029799 |
| 272.094 | 0.808248555 |
| 272.882 | 0.805933779 |
| 273.669 | 0.804600095 |
| 274.457 | 0.803094707 |
| 275.244 | 0.802820932 |
| 276.031 | 0.802998992 |
| 276.819 | 0.799479269 |
| 277.606 | 0.798437572 |
| 278.393 | 0.796773536 |
| 279.18 | 0.796333795 |
| 279.967 | 0.795169318 |
| 280.754 | 0.796749512 |
| 281.541 | 0.797944129 |
| 282.328 | 0.798343931 |
| 283.115 | 0.795823383 |
| 283.901 | 0.793790567 |
| 284.688 | 0.794301816 |
| 285.475 | 0.791034358 |
| 286.261 | 0.79153067 |
| 287.048 | 0.791063937 |
| 287.834 | 0.789039342 |
| 288.62 | 0.78633493 |
| 289.407 | 0.785398478 |
| 290.193 | 0.781344828 |
| 290.979 | 0.7802119 |
| 291.765 | 0.779451207 |
| 292.551 | 0.778371386 |
| 293.337 | 0.777372576 |
| 294.123 | 0.776130606 |
| 294.909 | 0.774154756 |
| 295.695 | 0.773999486 |
| 296.48 | 0.773770821 |
| 297.266 | 0.774089335 |
| 298.052 | 0.775102385 |
| 298.837 | 0.776120038 |
| 299.623 | 0.775703057 |
| 300.408 | 0.776466454 |
| 301.193 | 0.777565699 |
| 301.979 | 0.776804943 |
| 302.764 | 0.774158353 |
| 303.549 | 0.773219333 |
| 304.334 | 0.773878853 |
| 305.119 | 0.772857729 |
| 305.904 | 0.773188272 |
| 306.689 | 0.775115944 |
| 307.474 | 0.77793928 |
| 308.259 | 0.777072852 |
| 309.043 | 0.776861496 |
| 309.828 | 0.776396387 |
| 310.612 | 0.775573443 |
| 311.397 | 0.772738844 |
| 312.181 | 0.774101218 |
| 312.966 | 0.773590786 |
| 313.75 | 0.774630172 |
| 314.534 | 0.777108927 |
| 315.319 | 0.779368063 |
| 316.103 | 0.777014468 |
| 316.887 | 0.779005596 |
| 317.671 | 0.778452208 |
| 318.455 | 0.778287376 |
| 319.239 | 0.779102783 |
| 320.022 | 0.781323643 |
| 320.806 | 0.780400685 |
| 321.59 | 0.781895926 |
| 322.373 | 0.782186412 |
| 323.157 | 0.781718271 |
| 323.941 | 0.781482325 |
| 324.724 | 0.783205269 |
| 325.507 | 0.782618723 |
| 326.291 | 0.783156252 |
| 327.074 | 0.782727025 |
| 327.857 | 0.783381826 |
| 328.64 | 0.782093879 |
| 329.423 | 0.783300953 |
| 330.206 | 0.782447988 |
| 330.989 | 0.781870618 |
| 331.772 | 0.781296129 |
| 332.555 | 0.780615639 |
| 333.338 | 0.779438342 |
| 334.12 | 0.779040953 |
| 334.903 | 0.778928652 |
| 335.685 | 0.777816018 |
| 336.468 | 0.778894915 |
| 337.25 | 0.779861578 |
| 338.033 | 0.78045857 |
| 338.815 | 0.780009296 |
| 339.597 | 0.780043443 |
| 340.379 | 0.780390287 |
| 341.161 | 0.779764492 |
| 341.943 | 0.777868301 |
| 342.725 | 0.777921507 |
| 343.507 | 0.777980854 |
| 344.289 | 0.777655508 |
| 345.071 | 0.777132975 |
| 345.852 | 0.777121633 |
| 346.634 | 0.777523463 |
| 347.416 | 0.777113069 |
| 348.197 | 0.775591131 |
| 348.979 | 0.774287159 |
| 349.76 | 0.775560783 |
| 350.541 | 0.775685456 |
| 351.323 | 0.775735562 |
| 352.104 | 0.776240655 |
| 352.885 | 0.777743309 |
| 353.666 | 0.77940457 |
| 354.447 | 0.781974799 |
| 355.228 | 0.786346448 |
| 356.009 | 0.789669873 |
| 356.79 | 0.792375066 |
| 357.57 | 0.794483578 |
| 358.351 | 0.796808076 |
| 359.132 | 0.798635076 |
| 359.912 | 0.80359753 |
| 360.693 | 0.809084015 |
| 361.473 | 0.813384031 |
| 362.253 | 0.816267268 |
| 363.034 | 0.819136065 |
| 363.814 | 0.820653472 |
| 364.594 | 0.823740409 |
| 365.374 | 0.827557603 |
| 366.154 | 0.832644625 |
| 366.934 | 0.838018526 |
| 367.714 | 0.843959094 |
| 368.494 | 0.847436529 |
| 369.274 | 0.852385971 |
| 370.053 | 0.857704583 |
| 370.833 | 0.863684791 |
| 371.612 | 0.864607371 |
| 372.392 | 0.872394723 |
| 373.171 | 0.876379241 |
| 373.951 | 0.881316735 |
| 374.73 | 0.881977011 |
| 375.509 | 0.888086632 |
| 376.289 | 0.889390366 |
| 377.068 | 0.893604011 |
| 377.847 | 0.894778564 |
| 378.626 | 0.899886178 |
| 379.405 | 0.901911995 |
| 380.183 | 0.903802536 |
| 380.962 | 0.906497742 |
| 381.741 | 0.909345182 |
| 382.52 | 0.910803238 |
| 383.298 | 0.91401819 |
| 384.077 | 0.915390479 |
| 384.855 | 0.916236852 |
| 385.634 | 0.918496025 |
| 386.412 | 0.917725474 |
| 387.19 | 0.916115992 |
| 387.968 | 0.915588235 |
| 388.747 | 0.914677301 |
| 389.525 | 0.912288944 |
| 390.303 | 0.912760216 |
| 391.081 | 0.912632387 |
| 391.858 | 0.911808751 |
| 392.636 | 0.910194216 |
| 393.414 | 0.909925104 |
| 394.192 | 0.908341873 |
| 394.969 | 0.907227095 |
| 395.747 | 0.904914642 |
| 396.524 | 0.905296729 |
| 397.302 | 0.901560637 |
| 398.079 | 0.899143809 |
| 398.856 | 0.896278316 |
| 399.634 | 0.895426797 |
| 400.411 | 0.891024906 |
| 401.188 | 0.88928491 |
| 401.965 | 0.88818203 |
| 402.742 | 0.887214389 |
| 403.519 | 0.885034899 |
| 404.296 | 0.883553736 |
| 405.072 | 0.882000771 |
| 405.849 | 0.878678069 |
| 406.626 | 0.874922377 |
| 407.402 | 0.872044024 |
| 408.179 | 0.868247706 |
| 408.955 | 0.864515677 |
| 409.732 | 0.861194384 |
| 410.508 | 0.858784164 |
| 411.284 | 0.855237118 |
| 412.06 | 0.851851424 |
| 412.836 | 0.848804452 |
| 413.612 | 0.846043507 |
| 414.388 | 0.842156854 |
| 415.164 | 0.837819762 |
| 415.94 | 0.834032129 |
| 416.716 | 0.829911306 |
| 417.492 | 0.824805181 |
| 418.267 | 0.819584541 |
| 419.043 | 0.814774943 |
| 419.818 | 0.809857382 |
| 420.594 | 0.803957951 |
| 421.369 | 0.798823968 |
| 422.144 | 0.79371943 |
| 422.92 | 0.788500792 |
| 423.695 | 0.783286662 |
| 424.47 | 0.779075777 |
| 425.245 | 0.774610884 |
| 426.02 | 0.769705912 |
| 426.795 | 0.764852764 |
| 427.57 | 0.760318092 |
| 428.345 | 0.755026882 |
| 429.119 | 0.7507299 |
| 429.894 | 0.746562165 |
| 430.669 | 0.742508697 |
| 431.443 | 0.738810272 |
| 432.217 | 0.734898567 |
| 432.992 | 0.730511949 |
| 433.766 | 0.727757681 |
| 434.54 | 0.724142397 |
| 435.315 | 0.721317731 |
| 436.089 | 0.71878104 |
| 436.863 | 0.715948466 |
| 437.637 | 0.712504714 |
| 438.411 | 0.710547609 |
| 439.185 | 0.708081871 |
| 439.958 | 0.705698656 |
| 440.732 | 0.704149858 |
| 441.506 | 0.702164636 |
| 442.279 | 0.700538453 |
| 443.053 | 0.698567878 |
| 443.826 | 0.696907079 |
| 444.6 | 0.695224546 |
| 445.373 | 0.693605501 |
| 446.146 | 0.691971146 |
| 446.919 | 0.689976551 |
| 447.693 | 0.688551372 |
| 448.466 | 0.687108568 |
| 449.239 | 0.685596565 |
| 450.012 | 0.683837611 |
| 450.784 | 0.682788525 |
| 451.557 | 0.681159622 |
| 452.33 | 0.679430057 |
| 453.103 | 0.67837366 |
| 453.875 | 0.677110806 |
| 454.648 | 0.675337156 |
| 455.42 | 0.673591012 |
| 456.192 | 0.671629569 |
| 456.965 | 0.669348592 |
| 457.737 | 0.667100619 |
| 458.509 | 0.665109278 |
| 459.281 | 0.663429921 |
| 460.053 | 0.661894413 |
| 460.825 | 0.660095417 |
| 461.597 | 0.658479491 |
| 462.369 | 0.656346508 |
| 463.141 | 0.654704116 |
| 463.913 | 0.652428645 |
| 464.684 | 0.650249078 |
| 465.456 | 0.648298834 |
| 466.227 | 0.647231649 |
| 466.999 | 0.645188049 |
| 467.77 | 0.643784937 |
| 468.541 | 0.642015259 |
| 469.313 | 0.640019195 |
| 470.084 | 0.637556122 |
| 470.855 | 0.635415212 |
| 471.626 | 0.633317149 |
| 472.397 | 0.631479787 |
| 473.168 | 0.629323114 |
| 473.939 | 0.627734352 |
| 474.71 | 0.626049583 |
| 475.48 | 0.624318753 |
| 476.251 | 0.622897634 |
| 477.021 | 0.621433552 |
| 477.792 | 0.619390665 |
| 478.562 | 0.617575683 |
| 479.333 | 0.615789043 |
| 480.103 | 0.614106628 |
| 480.873 | 0.612739924 |
| 481.643 | 0.611528696 |
| 482.414 | 0.610302356 |
| 483.184 | 0.60897475 |
| 483.954 | 0.607776355 |
| 484.723 | 0.606780491 |
| 485.493 | 0.605818727 |
| 486.263 | 0.604656389 |
| 487.033 | 0.603751937 |
| 487.802 | 0.602847665 |
| 488.572 | 0.601718921 |
| 489.341 | 0.600787677 |
| 490.111 | 0.600348514 |
| 490.88 | 0.599601133 |
| 491.649 | 0.598620677 |
| 492.419 | 0.59807041 |
| 493.188 | 0.597877012 |
| 493.957 | 0.597250638 |
| 494.726 | 0.596703117 |
| 495.495 | 0.596652141 |
| 496.264 | 0.596377032 |
| 497.032 | 0.595913302 |
| 497.801 | 0.595374048 |
| 498.57 | 0.595376506 |
| 499.339 | 0.595025983 |
| 500.107 | 0.594826034 |
| 500.876 | 0.594527123 |
| 501.644 | 0.594857715 |
| 502.412 | 0.594847652 |
| 503.181 | 0.595065047 |
| 503.949 | 0.595116237 |
| 504.717 | 0.595044553 |
| 505.485 | 0.595056012 |
| 506.253 | 0.595450608 |
| 507.021 | 0.59581838 |
| 507.789 | 0.596055983 |
| 508.556 | 0.596840921 |
| 509.324 | 0.596995376 |
| 510.092 | 0.596924817 |
| 510.859 | 0.597184994 |
| 511.627 | 0.597746029 |
| 512.394 | 0.597828437 |
| 513.162 | 0.598476616 |
| 513.929 | 0.599258396 |
| 514.696 | 0.59964193 |
| 515.463 | 0.599859223 |
| 516.231 | 0.600561801 |
| 516.998 | 0.601082223 |
| 517.765 | 0.601667016 |
| 518.531 | 0.602589184 |
| 519.298 | 0.603501025 |
| 520.065 | 0.603871622 |
| 520.832 | 0.604616654 |
| 521.598 | 0.605498105 |
| 522.365 | 0.606098758 |
| 523.131 | 0.606669165 |
| 523.898 | 0.607726435 |
| 524.664 | 0.608281709 |
| 525.43 | 0.608931496 |
| 526.197 | 0.609690201 |
| 526.963 | 0.610480063 |
| 527.729 | 0.611135586 |
| 528.495 | 0.612240673 |
| 529.261 | 0.613031424 |
| 530.027 | 0.613656885 |
| 530.792 | 0.614445137 |
| 531.558 | 0.615354917 |
| 532.324 | 0.616582225 |
| 533.089 | 0.617518853 |
| 533.855 | 0.618633556 |
| 534.62 | 0.619942081 |
| 535.386 | 0.621046135 |
| 536.151 | 0.621724275 |
| 536.916 | 0.622633762 |
| 537.682 | 0.623770236 |
| 538.447 | 0.624866254 |
| 539.212 | 0.625585877 |
| 539.977 | 0.626755077 |
| 540.742 | 0.627960402 |
| 541.506 | 0.629098663 |
| 542.271 | 0.629997514 |
| 543.036 | 0.631594814 |
| 543.801 | 0.632979239 |
| 544.565 | 0.634291965 |
| 545.33 | 0.635543395 |
| 546.094 | 0.637221259 |
| 546.858 | 0.638666784 |
| 547.623 | 0.639868291 |
| 548.387 | 0.64128867 |
| 549.151 | 0.6428996 |
| 549.915 | 0.644214762 |
| 550.679 | 0.645821093 |
| 551.443 | 0.647439369 |
| 552.207 | 0.649247822 |
| 552.971 | 0.650843806 |
| 553.734 | 0.65264442 |
| 554.498 | 0.654317748 |
| 555.262 | 0.656176818 |
| 556.025 | 0.657840614 |
| 556.789 | 0.659478539 |
| 557.552 | 0.661286326 |
| 558.315 | 0.663156863 |
| 559.078 | 0.665240716 |
| 559.842 | 0.667234583 |
| 560.605 | 0.669437537 |
| 561.368 | 0.671354574 |
| 562.131 | 0.673291022 |
| 562.894 | 0.67544035 |
| 563.656 | 0.677665728 |
| 564.419 | 0.679879696 |
| 565.182 | 0.682390086 |
| 565.945 | 0.684737553 |
| 566.707 | 0.687034464 |
| 567.47 | 0.689249356 |
| 568.232 | 0.691655031 |
| 568.994 | 0.694059304 |
| 569.757 | 0.696985146 |
| 570.519 | 0.699264372 |
| 571.281 | 0.702235276 |
| 572.043 | 0.704857114 |
| 572.805 | 0.707572341 |
| 573.567 | 0.709926985 |
| 574.329 | 0.713147993 |
| 575.09 | 0.715965938 |
| 575.852 | 0.7189129 |
| 576.614 | 0.721740618 |
| 577.375 | 0.724816969 |
| 578.137 | 0.72758214 |
| 578.898 | 0.730419866 |
| 579.66 | 0.733473279 |
| 580.421 | 0.736684988 |
| 581.182 | 0.740132647 |
| 581.943 | 0.743374795 |
| 582.704 | 0.746835653 |
| 583.465 | 0.750308441 |
| 584.226 | 0.753445158 |
| 584.987 | 0.756029159 |
| 585.748 | 0.759167725 |
| 586.509 | 0.762230701 |
| 587.269 | 0.765351341 |
| 588.03 | 0.768949676 |
| 588.79 | 0.772980689 |
| 589.551 | 0.776628228 |
| 590.311 | 0.779755479 |
| 591.072 | 0.783396964 |
| 591.832 | 0.786842023 |
| 592.592 | 0.790176924 |
| 593.352 | 0.79366374 |
| 594.112 | 0.797449678 |
| 594.872 | 0.800885301 |
| 595.632 | 0.804516672 |
| 596.392 | 0.808077976 |
| 597.151 | 0.811679844 |
| 597.911 | 0.815544944 |
| 598.671 | 0.819337115 |
| 599.43 | 0.822715839 |
| 600.19 | 0.826220006 |
| 600.949 | 0.829819892 |
| 601.708 | 0.833212498 |
| 602.468 | 0.836442773 |
| 603.227 | 0.839945554 |
| 603.986 | 0.843455878 |
| 604.745 | 0.846174918 |
| 605.504 | 0.849536497 |
| 606.263 | 0.852649874 |
| 607.022 | 0.856090689 |
| 607.78 | 0.859668242 |
| 608.539 | 0.86343726 |
| 609.298 | 0.866778166 |
| 610.056 | 0.870388445 |
| 610.815 | 0.873600941 |
| 611.573 | 0.876578026 |
| 612.332 | 0.879899667 |
| 613.09 | 0.8837101 |
| 613.848 | 0.886496417 |
| 614.606 | 0.889840247 |
| 615.364 | 0.892604236 |
| 616.122 | 0.89605309 |
| 616.88 | 0.898706982 |
| 617.638 | 0.902122082 |
| 618.396 | 0.905020281 |
| 619.153 | 0.908288742 |
| 619.911 | 0.910669054 |
| 620.668 | 0.912903274 |
| 621.426 | 0.91502937 |
| 622.183 | 0.91744239 |
| 622.941 | 0.919800392 |
| 623.698 | 0.921927515 |
| 624.455 | 0.92444459 |
| 625.212 | 0.927440326 |
| 625.969 | 0.929765098 |
| 626.726 | 0.932258381 |
| 627.483 | 0.934932105 |
| 628.24 | 0.937018873 |
| 628.997 | 0.939016707 |
| 629.753 | 0.941211103 |
| 630.51 | 0.943071791 |
| 631.267 | 0.945260587 |
| 632.023 | 0.94739928 |
| 632.78 | 0.949697646 |
| 633.536 | 0.951562049 |
| 634.292 | 0.953293018 |
| 635.048 | 0.955076052 |
| 635.804 | 0.956997351 |
| 636.561 | 0.958387563 |
| 637.316 | 0.960097648 |
| 638.072 | 0.961844196 |
| 638.828 | 0.962874244 |
| 639.584 | 0.963896683 |
| 640.34 | 0.964937753 |
| 641.095 | 0.966088169 |
| 641.851 | 0.967670229 |
| 642.606 | 0.969104082 |
| 643.362 | 0.97043127 |
| 644.117 | 0.971540504 |
| 644.872 | 0.972418827 |
| 645.628 | 0.97290451 |
| 646.383 | 0.97324116 |
| 647.138 | 0.973858189 |
| 647.893 | 0.974794423 |
| 648.648 | 0.975320698 |
| 649.402 | 0.975532226 |
| 650.157 | 0.976349401 |
| 650.912 | 0.977084026 |
| 651.667 | 0.97741156 |
| 652.421 | 0.977685288 |
| 653.176 | 0.9783116 |
| 653.93 | 0.978496992 |
| 654.684 | 0.978196506 |
| 655.439 | 0.978605893 |
| 656.193 | 0.978587903 |
| 656.947 | 0.978756161 |
| 657.701 | 0.97931261 |
| 658.455 | 0.979589924 |
| 659.209 | 0.979674129 |
| 659.963 | 0.980240216 |
| 660.716 | 0.979919621 |
| 661.47 | 0.979643611 |
| 662.224 | 0.979599069 |
| 662.977 | 0.97909939 |
| 663.731 | 0.979022901 |
| 664.484 | 0.978929832 |
| 665.237 | 0.978895233 |
| 665.991 | 0.979650367 |
| 666.744 | 0.979857566 |
| 667.497 | 0.979221852 |
| 668.25 | 0.979640792 |
| 669.003 | 0.979147009 |
| 669.756 | 0.978145832 |
| 670.509 | 0.977534518 |
| 671.261 | 0.976941055 |
| 672.014 | 0.976064902 |
| 672.767 | 0.975991239 |
| 673.519 | 0.975093238 |
| 674.272 | 0.974880236 |
| 675.024 | 0.975233504 |
| 675.776 | 0.974821735 |
| 676.529 | 0.974329553 |
| 677.281 | 0.974262731 |
| 678.033 | 0.973523638 |
| 678.785 | 0.973220216 |
| 679.537 | 0.973199087 |
| 680.289 | 0.972893086 |
| 681.041 | 0.972610766 |
| 681.792 | 0.972387908 |
| 682.544 | 0.971775554 |
| 683.295 | 0.97089483 |
| 684.047 | 0.970409494 |
| 684.798 | 0.969661706 |
| 685.55 | 0.969281891 |
| 686.301 | 0.968471222 |
| 687.052 | 0.968217244 |
| 687.803 | 0.967517151 |
| 688.555 | 0.967265292 |
| 689.306 | 0.966849174 |
| 690.057 | 0.966630808 |
| 690.807 | 0.966588114 |
| 691.558 | 0.966168528 |
| 692.309 | 0.965732738 |
| 693.06 | 0.965279882 |
| 693.81 | 0.965017384 |
| 694.561 | 0.964092446 |
| 695.311 | 0.963847179 |
| 696.061 | 0.96330982 |
| 696.812 | 0.962947941 |
| 697.562 | 0.961926615 |
| 698.312 | 0.961818138 |
| 699.062 | 0.961328833 |
| 699.812 | 0.96127872 |
| 700.562 | 0.96096553 |
| 701.312 | 0.961159435 |
| 702.062 | 0.960310481 |
| 702.811 | 0.959829278 |
| 703.561 | 0.959203158 |
| 704.31 | 0.958489403 |
| 705.06 | 0.957946191 |
| 705.809 | 0.957822171 |
| 706.559 | 0.957235352 |
| 707.308 | 0.956944096 |
| 708.057 | 0.956377012 |
| 708.806 | 0.955994905 |
| 709.555 | 0.955102814 |
| 710.304 | 0.954321379 |
| 711.053 | 0.953652383 |
| 711.802 | 0.952842723 |
| 712.551 | 0.952413534 |
| 713.299 | 0.952423008 |
| 714.048 | 0.952442092 |
| 714.796 | 0.952158828 |
| 715.545 | 0.952413526 |
| 716.293 | 0.951563728 |
| 717.041 | 0.950806213 |
| 717.79 | 0.950683388 |
| 718.538 | 0.950470327 |
| 719.286 | 0.950174759 |
| 720.034 | 0.950143763 |
| 720.782 | 0.950355273 |
| 721.53 | 0.949667789 |
| 722.278 | 0.949454785 |
| 723.025 | 0.948682084 |
| 723.773 | 0.948380583 |
| 724.52 | 0.947613889 |
| 725.268 | 0.947418654 |
| 726.015 | 0.94661824 |
| 726.763 | 0.946353104 |
| 727.51 | 0.945754348 |
| 728.257 | 0.945862674 |
| 729.004 | 0.945387922 |
| 729.751 | 0.94531166 |
| 730.498 | 0.945139102 |
| 731.245 | 0.944310024 |
| 731.992 | 0.943438358 |
| 732.739 | 0.942658317 |
| 733.485 | 0.941457039 |
| 734.232 | 0.940575223 |
| 734.979 | 0.940491756 |
| 735.725 | 0.939712281 |
| 736.471 | 0.938912058 |
| 737.218 | 0.938320612 |
| 737.964 | 0.937171955 |
| 738.71 | 0.936072213 |
| 739.456 | 0.934750768 |
| 740.202 | 0.933980845 |
| 740.948 | 0.932736143 |
| 741.694 | 0.932596531 |
| 742.44 | 0.931289096 |
| 743.186 | 0.930286589 |
| 743.931 | 0.928919783 |
| 744.677 | 0.927584802 |
| 745.422 | 0.925408205 |
| 746.168 | 0.924077474 |
| 746.913 | 0.923145664 |
| 747.658 | 0.921958184 |
| 748.404 | 0.920728602 |
| 749.149 | 0.91956783 |
| 749.894 | 0.91787534 |
| 750.639 | 0.915822657 |
| 751.384 | 0.913979302 |
| 752.128 | 0.912234914 |
| 752.873 | 0.910566624 |
| 753.618 | 0.909025792 |
| 754.362 | 0.907430179 |
| 755.107 | 0.905836163 |
| 755.851 | 0.904277018 |
| 756.596 | 0.902518054 |
| 757.34 | 0.900554693 |
| 758.084 | 0.899148588 |
| 758.829 | 0.897237115 |
| 759.573 | 0.895374833 |
| 760.317 | 0.893753634 |
| 761.061 | 0.892460988 |
| 761.804 | 0.890611433 |
| 762.548 | 0.888763538 |
| 763.292 | 0.887158082 |
| 764.036 | 0.885159338 |
| 764.779 | 0.883259813 |
| 765.523 | 0.881265357 |
| 766.266 | 0.879830839 |
| 767.009 | 0.878036625 |
| 767.753 | 0.876339707 |
| 768.496 | 0.874754132 |
| 769.239 | 0.87320308 |
| 769.982 | 0.871178184 |
| 770.725 | 0.869384082 |
| 771.468 | 0.867329124 |
| 772.211 | 0.865521086 |
| 772.953 | 0.86347668 |
| 773.696 | 0.861809301 |
| 774.439 | 0.859858395 |
| 775.181 | 0.85808241 |
| 775.924 | 0.856516574 |
| 776.666 | 0.85501567 |
| 777.408 | 0.853623433 |
| 778.15 | 0.852133155 |
| 778.893 | 0.85037889 |
| 779.635 | 0.848589638 |
| 780.377 | 0.846959275 |
| 781.118 | 0.844955356 |
| 781.86 | 0.843589372 |
| 782.602 | 0.842596404 |
| 783.344 | 0.840990563 |
| 784.085 | 0.839683247 |
| 784.827 | 0.838270766 |
| 785.568 | 0.836843496 |
| 786.31 | 0.835165204 |
| 787.051 | 0.833651516 |
| 787.792 | 0.832221391 |
| 788.533 | 0.83103783 |
| 789.275 | 0.829802607 |
| 790.016 | 0.828573422 |
| 790.757 | 0.827810389 |
| 791.497 | 0.826485372 |
| 792.238 | 0.825330389 |
| 792.979 | 0.824043954 |
| 793.719 | 0.822907056 |
| 794.46 | 0.821402383 |
| 795.201 | 0.820291112 |
| 795.941 | 0.819408624 |
| 796.681 | 0.818373665 |
| 797.422 | 0.817560699 |
| 798.162 | 0.81636057 |
| 798.902 | 0.815599502 |
| 799.642 | 0.814774738 |
| 800.382 | 0.813858634 |
| 801.122 | 0.813134809 |
| 801.861 | 0.812866002 |
| 802.601 | 0.81179377 |
| 803.341 | 0.810747365 |
| 804.08 | 0.810152828 |
| 804.82 | 0.809196608 |
| 805.559 | 0.808059137 |
| 806.299 | 0.807520687 |
| 807.038 | 0.806579384 |
| 807.777 | 0.805812241 |
| 808.516 | 0.805396714 |
| 809.255 | 0.804739845 |
| 809.994 | 0.804268656 |
| 810.733 | 0.803884258 |
| 811.472 | 0.803163504 |
| 812.211 | 0.802532125 |
| 812.949 | 0.801993707 |
| 813.688 | 0.801281804 |
| 814.427 | 0.800965081 |
| 815.165 | 0.800633439 |
| 815.903 | 0.799930463 |
| 816.642 | 0.799605245 |
| 817.38 | 0.799135999 |
| 818.118 | 0.798740816 |
| 818.856 | 0.798227246 |
| 819.594 | 0.798385564 |
| 820.332 | 0.79813153 |
| 821.07 | 0.797874243 |
| 821.808 | 0.797239653 |
| 822.545 | 0.79698961 |
| 823.283 | 0.796319953 |
| 824.02 | 0.796155848 |
| 824.758 | 0.79593961 |
| 825.495 | 0.795944316 |
| 826.232 | 0.795782307 |
| 826.97 | 0.795220631 |
| 827.707 | 0.794873256 |
| 828.444 | 0.794680251 |
| 829.181 | 0.794289849 |
| 829.918 | 0.794550813 |
| 830.655 | 0.794726493 |
| 831.391 | 0.794713362 |
| 832.128 | 0.794894722 |
| 832.865 | 0.794839274 |
| 833.601 | 0.79428967 |
| 834.338 | 0.794297743 |
| 835.074 | 0.794175278 |
| 835.81 | 0.794044689 |
| 836.547 | 0.794474077 |
| 837.283 | 0.794674403 |
| 838.019 | 0.794529556 |
| 838.755 | 0.794856957 |
| 839.491 | 0.795016719 |
| 840.227 | 0.794797755 |
| 840.962 | 0.79492506 |
| 841.698 | 0.795659544 |
| 842.434 | 0.795720973 |
| 843.169 | 0.795740434 |
| 843.905 | 0.796212091 |
| 844.64 | 0.79631215 |
| 845.375 | 0.796360747 |
| 846.111 | 0.796607295 |
| 846.846 | 0.796931072 |
| 847.581 | 0.797420075 |
| 848.316 | 0.79734036 |
| 849.051 | 0.797470847 |
| 849.786 | 0.797757602 |
| 850.52 | 0.798095191 |
| 851.255 | 0.797892714 |
| 851.99 | 0.798776342 |
| 852.724 | 0.799178777 |
| 853.459 | 0.799461359 |
| 854.193 | 0.799928083 |
| 854.927 | 0.800263155 |
| 855.662 | 0.80048819 |
| 856.396 | 0.800571559 |
| 857.13 | 0.800712622 |
| 857.864 | 0.800619601 |
| 858.598 | 0.801392696 |
| 859.332 | 0.801664814 |
| 860.065 | 0.802139991 |
| 860.799 | 0.802628684 |
| 861.533 | 0.803264597 |
| 862.266 | 0.803379217 |
| 863 | 0.803717392 |
| 863.733 | 0.804101951 |
| 864.466 | 0.804785013 |
| 865.2 | 0.805187791 |
| 865.933 | 0.805693587 |
| 866.666 | 0.806522705 |
| 867.399 | 0.807048186 |
| 868.132 | 0.807703364 |
| 868.865 | 0.808344836 |
| 869.597 | 0.808586271 |
| 870.33 | 0.809041076 |
| 871.063 | 0.809752334 |
| 871.795 | 0.809908601 |
| 872.528 | 0.810281982 |
| 873.26 | 0.811161604 |
| 873.992 | 0.811822902 |
| 874.725 | 0.812504955 |
| 875.457 | 0.813358128 |
| 876.189 | 0.813935873 |
| 876.921 | 0.814478926 |
| 877.653 | 0.81514642 |
| 878.385 | 0.815751953 |
| 879.116 | 0.816484161 |
| 879.848 | 0.817179677 |
| 880.58 | 0.818102489 |
| 881.311 | 0.818798912 |
| 882.043 | 0.819441246 |
| 882.774 | 0.820315658 |
| 883.505 | 0.821156883 |
| 884.236 | 0.821510006 |
| 884.968 | 0.822136053 |
| 885.699 | 0.822877148 |
| 886.43 | 0.823265425 |
| 887.161 | 0.823920317 |
| 887.891 | 0.824743727 |
| 888.622 | 0.825271928 |
| 889.353 | 0.825927292 |
| 890.083 | 0.826447362 |
| 890.814 | 0.827145188 |
| 891.544 | 0.828237688 |
| 892.275 | 0.829264149 |
| 893.005 | 0.830192633 |
| 893.735 | 0.831177207 |
| 894.465 | 0.831956963 |
| 895.195 | 0.832314997 |
| 895.925 | 0.83296315 |
| 896.655 | 0.833394975 |
| 897.385 | 0.83455098 |
| 898.115 | 0.835584843 |
| 898.844 | 0.836714971 |
| 899.574 | 0.837797141 |
| 900.304 | 0.838752936 |
| 901.033 | 0.839203174 |
| 901.762 | 0.840253919 |
| 902.492 | 0.841343942 |
| 903.221 | 0.842210299 |
| 903.95 | 0.843339288 |
| 904.679 | 0.844552747 |
| 905.408 | 0.845216583 |
| 906.137 | 0.845754845 |
| 906.866 | 0.846572297 |
| 907.594 | 0.847585465 |
| 908.323 | 0.84848486 |
| 909.051 | 0.84953025 |
| 909.78 | 0.850723273 |
| 910.508 | 0.851761397 |
| 911.237 | 0.852575534 |
| 911.965 | 0.853449216 |
| 912.693 | 0.854522152 |
| 913.421 | 0.85506012 |
| 914.149 | 0.855954545 |
| 914.877 | 0.857161223 |
| 915.605 | 0.858385329 |
| 916.333 | 0.859404039 |
| 917.06 | 0.860823015 |
| 917.788 | 0.861793531 |
| 918.516 | 0.862528284 |
| 919.243 | 0.863677698 |
| 919.97 | 0.864703189 |
| 920.698 | 0.865585938 |
| 921.425 | 0.866693474 |
| 922.152 | 0.86780535 |
| 922.879 | 0.868736149 |
| 923.606 | 0.869820404 |
| 924.333 | 0.87091387 |
| 925.06 | 0.872023384 |
| 925.787 | 0.873182073 |
| 926.513 | 0.874029268 |
| 927.24 | 0.874699893 |
| 927.966 | 0.87634174 |
| 928.693 | 0.877488934 |
| 929.419 | 0.878626427 |
| 930.146 | 0.879566943 |
| 930.872 | 0.880999734 |
| 931.598 | 0.881891239 |
| 932.324 | 0.882895965 |
| 933.05 | 0.883782421 |
| 933.776 | 0.884824473 |
| 934.502 | 0.886008188 |
| 935.227 | 0.886745737 |
| 935.953 | 0.887743882 |
| 936.679 | 0.888891837 |
| 937.404 | 0.890665745 |
| 938.129 | 0.891702124 |
| 938.855 | 0.892639428 |
| 939.58 | 0.894143072 |
| 940.305 | 0.895591345 |
| 941.03 | 0.896270254 |
| 941.755 | 0.897672351 |
| 942.48 | 0.89921044 |
| 943.205 | 0.900211646 |
| 943.93 | 0.901489475 |
| 944.655 | 0.902848991 |
| 945.379 | 0.903921612 |
| 946.104 | 0.905149131 |
| 946.828 | 0.905874633 |
| 947.553 | 0.907015558 |
| 948.277 | 0.908039326 |
| 949.001 | 0.909133398 |
| 949.725 | 0.91048331 |
| 950.449 | 0.912340343 |
| 951.173 | 0.913916199 |
| 951.897 | 0.915564499 |
| 952.621 | 0.916898412 |
| 953.345 | 0.918157432 |
| 954.068 | 0.919351329 |
| 954.792 | 0.920362883 |
| 955.515 | 0.92128242 |
| 956.239 | 0.922836679 |
| 956.962 | 0.924192021 |
| 957.686 | 0.925463901 |
| 958.409 | 0.926497203 |
| 959.132 | 0.927968132 |
| 959.855 | 0.92903931 |
| 960.578 | 0.930531926 |
| 961.301 | 0.931299164 |
| 962.023 | 0.932581327 |
| 962.746 | 0.934006413 |
| 963.469 | 0.934980409 |
| 964.191 | 0.935769658 |
| 964.914 | 0.937647773 |
| 965.636 | 0.938862888 |
| 966.359 | 0.939528148 |
| 967.081 | 0.940424591 |
| 967.803 | 0.941419091 |
| 968.525 | 0.941810621 |
| 969.247 | 0.942429043 |
| 969.969 | 0.943457941 |
| 970.691 | 0.944388585 |
| 971.412 | 0.945491482 |
| 972.134 | 0.946687121 |
| 972.856 | 0.947961608 |
| 973.577 | 0.949268723 |
| 974.299 | 0.950284686 |
| 975.02 | 0.950973961 |
| 975.741 | 0.952001976 |
| 976.462 | 0.952753417 |
| 977.184 | 0.95310376 |
| 977.905 | 0.954725519 |
| 978.626 | 0.956033414 |
| 979.346 | 0.957181493 |
| 980.067 | 0.958516548 |
| 980.788 | 0.960161019 |
| 981.509 | 0.961151391 |
| 982.229 | 0.962223335 |
| 982.95 | 0.963127965 |
| 983.67 | 0.963898684 |
| 984.39 | 0.964876298 |
| 985.111 | 0.965917622 |
| 985.831 | 0.966975929 |
| 986.551 | 0.967852641 |
| 987.271 | 0.969187588 |
| 987.991 | 0.970191694 |
| 988.71 | 0.971032767 |
| 989.43 | 0.972405492 |
| 990.15 | 0.973736992 |
| 990.869 | 0.974852372 |
| 991.589 | 0.976112238 |
| 992.308 | 0.977287429 |
| 993.028 | 0.977478305 |
| 993.747 | 0.978534951 |
| 994.466 | 0.979778587 |
| 995.185 | 0.981087682 |
| 995.904 | 0.981818497 |
| 996.623 | 0.982863044 |
| 997.342 | 0.983378798 |
| 998.061 | 0.983602323 |
| 998.78 | 0.982720171 |
| 999.498 | 0.982448486 |
| 1000.217 | 0.964555809 |
| 1000.935 | 0.928446415 |
| 1001.654 | 0.930463422 |
| 1002.372 | 0.929412398 |
| 1003.09 | 0.92358559 |
| 1003.808 | 0.910740151 |
| 1004.526 | 0.984121597 |
| 1005.244 | 0.985465367 |
| 1005.962 | 0.986439211 |
| 1006.68 | 0.987359807 |
| 1007.398 | 0.987659455 |
| 1008.115 | 0.987732177 |
| 1008.833 | 0.988069915 |
| 1009.55 | 0.988133716 |
| 1010.268 | 0.987606351 |
| 1010.985 | 0.987766008 |
| 1011.702 | 1.060207055 |
| 1012.419 | 1.065188953 |

Date of figure 4(a)

| *h*° | 151.76 | 156.09 | 162.62 | 165.59 | 170.58 |
| --- | --- | --- | --- | --- | --- |
| wavelength | Absorbance(normalize) | | | | |
| 223.933 | 0.52482 | 0.5809 | 0.56032 | 0.50912 | 0.50687 |
| 224.724 | 0.4734 | 0.61749 | 0.62791 | 0.47178 | 0.51611 |
| 225.516 | 0.4927 | 0.65195 | 0.60495 | 0.51457 | 0.50506 |
| 226.307 | 0.49747 | 0.67463 | 0.6229 | 0.52529 | 0.52171 |
| 227.098 | 0.50601 | 0.69095 | 0.61928 | 0.54136 | 0.52431 |
| 227.889 | 0.51597 | 0.70388 | 0.61247 | 0.56929 | 0.52894 |
| 228.68 | 0.51406 | 0.73463 | 0.6203 | 0.5925 | 0.54245 |
| 229.471 | 0.52518 | 0.72224 | 0.62625 | 0.58634 | 0.54266 |
| 230.262 | 0.53896 | 0.71625 | 0.62189 | 0.57343 | 0.5592 |
| 231.053 | 0.54713 | 0.72034 | 0.62066 | 0.58716 | 0.5704 |
| 231.844 | 0.54711 | 0.71863 | 0.62753 | 0.5857 | 0.58461 |
| 232.635 | 0.55764 | 0.70301 | 0.64152 | 0.57608 | 0.5841 |
| 233.425 | 0.55755 | 0.71398 | 0.65321 | 0.59504 | 0.59448 |
| 234.216 | 0.55678 | 0.71557 | 0.6683 | 0.60415 | 0.57729 |
| 235.007 | 0.55707 | 0.71003 | 0.67714 | 0.59906 | 0.57951 |
| 235.797 | 0.55917 | 0.71087 | 0.68411 | 0.59242 | 0.57688 |
| 236.588 | 0.56182 | 0.70902 | 0.66765 | 0.58327 | 0.56872 |
| 237.378 | 0.5738 | 0.71191 | 0.65527 | 0.58732 | 0.57566 |
| 238.168 | 0.56751 | 0.7206 | 0.639 | 0.59364 | 0.58042 |
| 238.959 | 0.56104 | 0.7327 | 0.63149 | 0.59212 | 0.55464 |
| 239.749 | 0.56626 | 0.73195 | 0.61427 | 0.59148 | 0.53195 |
| 240.539 | 0.56014 | 0.73612 | 0.60698 | 0.58331 | 0.52563 |
| 241.329 | 0.54846 | 0.72219 | 0.59822 | 0.55506 | 0.50965 |
| 242.119 | 0.55221 | 0.7158 | 0.58495 | 0.54383 | 0.49151 |
| 242.909 | 0.55713 | 0.6954 | 0.57445 | 0.52731 | 0.50116 |
| 243.699 | 0.55425 | 0.69054 | 0.55829 | 0.5091 | 0.49959 |
| 244.489 | 0.55386 | 0.67769 | 0.54413 | 0.49983 | 0.49694 |
| 245.279 | 0.54817 | 0.67117 | 0.53725 | 0.4856 | 0.4797 |
| 246.068 | 0.541 | 0.66567 | 0.52869 | 0.46044 | 0.47222 |
| 246.858 | 0.53307 | 0.6609 | 0.51748 | 0.42684 | 0.43958 |
| 247.648 | 0.51944 | 0.643 | 0.50749 | 0.39751 | 0.40515 |
| 248.437 | 0.50751 | 0.63047 | 0.49775 | 0.36954 | 0.38434 |
| 249.227 | 0.50265 | 0.6188 | 0.47687 | 0.34048 | 0.36224 |
| 250.016 | 0.49272 | 0.6008 | 0.46591 | 0.30612 | 0.33851 |
| 250.805 | 0.48123 | 0.58863 | 0.45131 | 0.2861 | 0.31396 |
| 251.595 | 0.47672 | 0.58455 | 0.44249 | 0.27078 | 0.29847 |
| 252.384 | 0.47256 | 0.57881 | 0.42632 | 0.24774 | 0.27644 |
| 253.173 | 0.46903 | 0.57138 | 0.42149 | 0.2372 | 0.26255 |
| 253.962 | 0.45677 | 0.56503 | 0.40545 | 0.22668 | 0.24524 |
| 254.751 | 0.45053 | 0.56049 | 0.39102 | 0.21634 | 0.23177 |
| 255.54 | 0.44284 | 0.55402 | 0.37942 | 0.20232 | 0.22283 |
| 256.329 | 0.43054 | 0.54467 | 0.37187 | 0.19871 | 0.20702 |
| 257.118 | 0.42547 | 0.53415 | 0.36271 | 0.17608 | 0.1926 |
| 257.907 | 0.43022 | 0.5209 | 0.35842 | 0.16298 | 0.18226 |
| 258.695 | 0.42823 | 0.50918 | 0.35009 | 0.14373 | 0.17869 |
| 259.484 | 0.43014 | 0.49731 | 0.34471 | 0.12735 | 0.17335 |
| 260.273 | 0.4349 | 0.48699 | 0.33741 | 0.10796 | 0.16592 |
| 261.061 | 0.43307 | 0.48064 | 0.32817 | 0.09933 | 0.15667 |
| 261.85 | 0.43505 | 0.47861 | 0.31774 | 0.09241 | 0.14935 |
| 262.638 | 0.43929 | 0.47159 | 0.31623 | 0.08973 | 0.14617 |
| 263.426 | 0.43307 | 0.46962 | 0.31007 | 0.08335 | 0.13347 |
| 264.215 | 0.43321 | 0.46396 | 0.30806 | 0.07423 | 0.12456 |
| 265.003 | 0.43569 | 0.45867 | 0.30154 | 0.07362 | 0.11838 |
| 265.791 | 0.429 | 0.45045 | 0.30051 | 0.06829 | 0.11255 |
| 266.579 | 0.42977 | 0.44588 | 0.293 | 0.06331 | 0.10595 |
| 267.367 | 0.43356 | 0.43487 | 0.28788 | 0.06186 | 0.10524 |
| 268.155 | 0.4316 | 0.43241 | 0.2834 | 0.06616 | 0.10806 |
| 268.943 | 0.42567 | 0.4281 | 0.2844 | 0.05886 | 0.10997 |
| 269.731 | 0.42597 | 0.42604 | 0.27968 | 0.05809 | 0.11013 |
| 270.519 | 0.42325 | 0.42208 | 0.28017 | 0.06008 | 0.10439 |
| 271.306 | 0.41543 | 0.42123 | 0.27831 | 0.06118 | 0.10117 |
| 272.094 | 0.42001 | 0.41575 | 0.27037 | 0.0616 | 0.10357 |
| 272.882 | 0.41705 | 0.41354 | 0.26774 | 0.06666 | 0.09613 |
| 273.669 | 0.41483 | 0.41344 | 0.26506 | 0.07431 | 0.09293 |
| 274.457 | 0.41182 | 0.41435 | 0.26298 | 0.0739 | 0.09094 |
| 275.244 | 0.42181 | 0.41421 | 0.26172 | 0.0769 | 0.09746 |
| 276.031 | 0.41772 | 0.41383 | 0.26167 | 0.07402 | 0.09866 |
| 276.819 | 0.43244 | 0.41661 | 0.26229 | 0.07811 | 0.10891 |
| 277.606 | 0.44153 | 0.41243 | 0.26206 | 0.07413 | 0.11374 |
| 278.393 | 0.451 | 0.40467 | 0.26058 | 0.07346 | 0.11876 |
| 279.18 | 0.44714 | 0.39937 | 0.2604 | 0.06773 | 0.12326 |
| 279.967 | 0.45147 | 0.40294 | 0.26577 | 0.07168 | 0.11924 |
| 280.754 | 0.44802 | 0.3959 | 0.26693 | 0.06779 | 0.1203 |
| 281.541 | 0.44379 | 0.39924 | 0.2685 | 0.0673 | 0.11613 |
| 282.328 | 0.44332 | 0.40679 | 0.26728 | 0.06738 | 0.11548 |
| 283.115 | 0.44197 | 0.4079 | 0.26307 | 0.06703 | 0.10013 |
| 283.901 | 0.43698 | 0.41004 | 0.25852 | 0.06033 | 0.09507 |
| 284.688 | 0.43539 | 0.41434 | 0.25066 | 0.05583 | 0.08932 |
| 285.475 | 0.43482 | 0.40615 | 0.24738 | 0.05179 | 0.08435 |
| 286.261 | 0.43445 | 0.39797 | 0.24518 | 0.04661 | 0.08313 |
| 287.048 | 0.43179 | 0.40129 | 0.24264 | 0.04685 | 0.08566 |
| 287.834 | 0.44358 | 0.39393 | 0.24129 | 0.04648 | 0.08503 |
| 288.62 | 0.44627 | 0.38737 | 0.24252 | 0.0484 | 0.09126 |
| 289.407 | 0.44693 | 0.38633 | 0.23515 | 0.04508 | 0.08727 |
| 290.193 | 0.44529 | 0.38529 | 0.23443 | 0.04213 | 0.08474 |
| 290.979 | 0.45581 | 0.38257 | 0.23427 | 0.0383 | 0.0809 |
| 291.765 | 0.45054 | 0.37979 | 0.23227 | 0.03618 | 0.07451 |
| 292.551 | 0.44779 | 0.38212 | 0.22791 | 0.0336 | 0.05902 |
| 293.337 | 0.44645 | 0.37897 | 0.23039 | 0.02666 | 0.0592 |
| 294.123 | 0.44022 | 0.37637 | 0.22884 | 0.02494 | 0.04988 |
| 294.909 | 0.43173 | 0.36646 | 0.22387 | 0.02106 | 0.04198 |
| 295.695 | 0.43171 | 0.35881 | 0.22339 | 0.01744 | 0.03707 |
| 296.48 | 0.42255 | 0.34699 | 0.22389 | 0.00859 | 0.03108 |
| 297.266 | 0.4236 | 0.34251 | 0.22327 | 0.00443 | 0.01904 |
| 298.052 | 0.42781 | 0.34458 | 0.22137 | 3.90E-04 | 0.0184 |
| 298.837 | 0.42863 | 0.34809 | 0.2235 | 0 | 0.01581 |
| 299.623 | 0.42803 | 0.35083 | 0.22402 | 0.00318 | 0.01607 |
| 300.408 | 0.43722 | 0.35815 | 0.22592 | 0.00814 | 0.01811 |
| 301.193 | 0.43739 | 0.36151 | 0.22439 | 0.01003 | 0.02084 |
| 301.979 | 0.43668 | 0.35459 | 0.22546 | 0.00833 | 0.01889 |
| 302.764 | 0.44086 | 0.35283 | 0.22465 | 0.00765 | 0.02209 |
| 303.549 | 0.4422 | 0.35151 | 0.22271 | 0.00642 | 0.01821 |
| 304.334 | 0.44393 | 0.35172 | 0.22039 | 0.0055 | 0.01899 |
| 305.119 | 0.44499 | 0.35364 | 0.22412 | 0.01016 | 0.02213 |
| 305.904 | 0.45003 | 0.3552 | 0.22759 | 0.01811 | 0.02608 |
| 306.689 | 0.45697 | 0.35601 | 0.2301 | 0.01947 | 0.02376 |
| 307.474 | 0.46149 | 0.35804 | 0.22897 | 0.0177 | 0.02552 |
| 308.259 | 0.46212 | 0.35428 | 0.23077 | 0.01396 | 0.01921 |
| 309.043 | 0.46569 | 0.35344 | 0.23149 | 0.01137 | 0.01443 |
| 309.828 | 0.46772 | 0.35477 | 0.23093 | 0.00439 | 0.00367 |
| 310.612 | 0.47029 | 0.35477 | 0.23375 | 0.00634 | 0.00245 |
| 311.397 | 0.4688 | 0.35348 | 0.23681 | 0.00893 | 0 |
| 312.181 | 0.47552 | 0.35616 | 0.23586 | 0.01697 | 5.27E-04 |
| 312.966 | 0.48101 | 0.35438 | 0.23601 | 0.01767 | 0.00334 |
| 313.75 | 0.48696 | 0.35476 | 0.23089 | 0.02703 | 0.00816 |
| 314.534 | 0.48559 | 0.35528 | 0.22927 | 0.02898 | 0.00795 |
| 315.319 | 0.48997 | 0.35783 | 0.23366 | 0.03446 | 0.01355 |
| 316.103 | 0.48877 | 0.35851 | 0.23605 | 0.0357 | 0.01126 |
| 316.887 | 0.49087 | 0.35794 | 0.23583 | 0.04039 | 0.01138 |
| 317.671 | 0.49382 | 0.36343 | 0.23885 | 0.03887 | 0.01619 |
| 318.455 | 0.49836 | 0.36443 | 0.23999 | 0.03799 | 0.0166 |
| 319.239 | 0.50081 | 0.36168 | 0.2405 | 0.03216 | 0.01702 |
| 320.022 | 0.50803 | 0.36039 | 0.24413 | 0.03523 | 0.01852 |
| 320.806 | 0.51246 | 0.35815 | 0.24395 | 0.03412 | 0.01602 |
| 321.59 | 0.51066 | 0.35456 | 0.24716 | 0.03456 | 0.01478 |
| 322.373 | 0.51287 | 0.35651 | 0.2507 | 0.03896 | 0.01211 |
| 323.157 | 0.52364 | 0.3616 | 0.24975 | 0.04452 | 0.00459 |
| 323.941 | 0.52354 | 0.36466 | 0.25033 | 0.0459 | 0.00233 |
| 324.724 | 0.52345 | 0.37374 | 0.25398 | 0.05133 | 4.31E-04 |
| 325.507 | 0.5307 | 0.3742 | 0.25459 | 0.05658 | 5.61E-04 |
| 326.291 | 0.53543 | 0.37128 | 0.2554 | 0.05545 | 0.00388 |
| 327.074 | 0.53483 | 0.36864 | 0.25565 | 0.05621 | 0.00613 |
| 327.857 | 0.54046 | 0.36583 | 0.25064 | 0.05615 | 0.0118 |
| 328.64 | 0.54279 | 0.36135 | 0.25001 | 0.0538 | 0.01557 |
| 329.423 | 0.5431 | 0.35789 | 0.25021 | 0.05718 | 0.01622 |
| 330.206 | 0.5432 | 0.35671 | 0.25173 | 0.05725 | 0.01713 |
| 330.989 | 0.54543 | 0.35594 | 0.25278 | 0.05972 | 0.01906 |
| 331.772 | 0.54659 | 0.35531 | 0.2571 | 0.05707 | 0.02088 |
| 332.555 | 0.54976 | 0.35364 | 0.25716 | 0.06355 | 0.02234 |
| 333.338 | 0.55412 | 0.35642 | 0.2584 | 0.05924 | 0.02276 |
| 334.12 | 0.55517 | 0.35659 | 0.25481 | 0.06552 | 0.01789 |
| 334.903 | 0.55027 | 0.35607 | 0.25719 | 0.0645 | 0.01695 |
| 335.685 | 0.5541 | 0.36102 | 0.25737 | 0.06816 | 0.01474 |
| 336.468 | 0.55322 | 0.35819 | 0.25951 | 0.06893 | 0.01749 |
| 337.25 | 0.55559 | 0.36183 | 0.25924 | 0.07917 | 0.01377 |
| 338.033 | 0.56336 | 0.36287 | 0.25929 | 0.08271 | 0.01683 |
| 338.815 | 0.57253 | 0.36365 | 0.25636 | 0.08984 | 0.0191 |
| 339.597 | 0.57704 | 0.36327 | 0.25755 | 0.09687 | 0.02642 |
| 340.379 | 0.58264 | 0.37166 | 0.2579 | 0.10324 | 0.02373 |
| 341.161 | 0.58274 | 0.36905 | 0.26074 | 0.10421 | 0.03224 |
| 341.943 | 0.5821 | 0.37122 | 0.26735 | 0.10821 | 0.03423 |
| 342.725 | 0.58253 | 0.37039 | 0.27132 | 0.11102 | 0.04385 |
| 343.507 | 0.57745 | 0.3716 | 0.27196 | 0.11873 | 0.03712 |
| 344.289 | 0.57839 | 0.3715 | 0.27483 | 0.12237 | 0.04336 |
| 345.071 | 0.57566 | 0.37452 | 0.27626 | 0.12318 | 0.04389 |
| 345.852 | 0.57707 | 0.37634 | 0.27324 | 0.1275 | 0.05875 |
| 346.634 | 0.57577 | 0.38412 | 0.27422 | 0.13653 | 0.05766 |
| 347.416 | 0.5769 | 0.38666 | 0.27375 | 0.1411 | 0.07211 |
| 348.197 | 0.57707 | 0.39146 | 0.27344 | 0.15584 | 0.07816 |
| 348.979 | 0.58157 | 0.39751 | 0.27357 | 0.16998 | 0.09019 |
| 349.76 | 0.58379 | 0.40449 | 0.27822 | 0.18159 | 0.10028 |
| 350.541 | 0.58925 | 0.41015 | 0.28375 | 0.19248 | 0.11918 |
| 351.323 | 0.59651 | 0.41875 | 0.29119 | 0.20339 | 0.13144 |
| 352.104 | 0.60275 | 0.42269 | 0.2996 | 0.20577 | 0.15558 |
| 352.885 | 0.60191 | 0.43018 | 0.31166 | 0.21831 | 0.16843 |
| 353.666 | 0.60178 | 0.4414 | 0.32058 | 0.23218 | 0.1811 |
| 354.447 | 0.603 | 0.44983 | 0.32982 | 0.24281 | 0.19784 |
| 355.228 | 0.59997 | 0.45943 | 0.34003 | 0.2544 | 0.22063 |
| 356.009 | 0.60605 | 0.47371 | 0.34979 | 0.27081 | 0.23165 |
| 356.79 | 0.61448 | 0.48902 | 0.35564 | 0.29184 | 0.25214 |
| 357.57 | 0.62635 | 0.50162 | 0.36698 | 0.30297 | 0.27542 |
| 358.351 | 0.63066 | 0.51416 | 0.37517 | 0.32739 | 0.29162 |
| 359.132 | 0.64041 | 0.52396 | 0.38471 | 0.34883 | 0.30828 |
| 359.912 | 0.64863 | 0.53905 | 0.39727 | 0.36693 | 0.3352 |
| 360.693 | 0.65119 | 0.55072 | 0.41006 | 0.37971 | 0.36183 |
| 361.473 | 0.6542 | 0.55994 | 0.41806 | 0.40606 | 0.38444 |
| 362.253 | 0.6639 | 0.57534 | 0.4326 | 0.42498 | 0.41746 |
| 363.034 | 0.67498 | 0.59511 | 0.44963 | 0.44979 | 0.45048 |
| 363.814 | 0.67904 | 0.60873 | 0.46015 | 0.47639 | 0.47983 |
| 364.594 | 0.69167 | 0.62402 | 0.47297 | 0.49954 | 0.50621 |
| 365.374 | 0.70027 | 0.63406 | 0.48913 | 0.52409 | 0.52881 |
| 366.154 | 0.7113 | 0.65051 | 0.49868 | 0.54752 | 0.54564 |
| 366.934 | 0.7141 | 0.6651 | 0.51203 | 0.56598 | 0.56104 |
| 367.714 | 0.72353 | 0.68105 | 0.52504 | 0.58672 | 0.57431 |
| 368.494 | 0.73724 | 0.69751 | 0.54098 | 0.60776 | 0.59175 |
| 369.274 | 0.74469 | 0.7159 | 0.55573 | 0.61509 | 0.60227 |
| 370.053 | 0.748 | 0.7288 | 0.56948 | 0.62728 | 0.6209 |
| 370.833 | 0.76022 | 0.73818 | 0.57673 | 0.64935 | 0.64152 |
| 371.612 | 0.76652 | 0.74265 | 0.5915 | 0.66154 | 0.66055 |
| 372.392 | 0.76348 | 0.74737 | 0.60162 | 0.67858 | 0.67741 |
| 373.171 | 0.764 | 0.75746 | 0.61336 | 0.70002 | 0.69681 |
| 373.951 | 0.77352 | 0.76626 | 0.62695 | 0.71539 | 0.71784 |
| 374.73 | 0.77202 | 0.77081 | 0.64482 | 0.72413 | 0.73803 |
| 375.509 | 0.7738 | 0.78386 | 0.65141 | 0.73937 | 0.75289 |
| 376.289 | 0.78157 | 0.79784 | 0.66231 | 0.75628 | 0.77766 |
| 377.068 | 0.79163 | 0.80609 | 0.66966 | 0.77228 | 0.78681 |
| 377.847 | 0.79791 | 0.80957 | 0.68124 | 0.78679 | 0.78817 |
| 378.626 | 0.80848 | 0.81794 | 0.67857 | 0.79313 | 0.79254 |
| 379.405 | 0.80818 | 0.82468 | 0.68187 | 0.79642 | 0.7997 |
| 380.183 | 0.81551 | 0.82634 | 0.6924 | 0.7966 | 0.79222 |
| 380.962 | 0.81359 | 0.83962 | 0.69426 | 0.81063 | 0.79914 |
| 381.741 | 0.81085 | 0.8487 | 0.69656 | 0.80699 | 0.81397 |
| 382.52 | 0.81009 | 0.85906 | 0.70655 | 0.81033 | 0.80489 |
| 383.298 | 0.81458 | 0.86106 | 0.71521 | 0.82068 | 0.7959 |
| 384.077 | 0.81455 | 0.86086 | 0.70934 | 0.82742 | 0.80644 |
| 384.855 | 0.81124 | 0.85823 | 0.7067 | 0.81972 | 0.81349 |
| 385.634 | 0.81084 | 0.85629 | 0.70695 | 0.82167 | 0.81188 |
| 386.412 | 0.80944 | 0.85174 | 0.7067 | 0.82625 | 0.82616 |
| 387.19 | 0.81017 | 0.84877 | 0.708 | 0.82968 | 0.83981 |
| 387.968 | 0.80816 | 0.85212 | 0.70874 | 0.83155 | 0.83658 |
| 388.747 | 0.81259 | 0.85584 | 0.71483 | 0.83022 | 0.83169 |
| 389.525 | 0.81227 | 0.86043 | 0.71451 | 0.83836 | 0.83308 |
| 390.303 | 0.81657 | 0.86462 | 0.71252 | 0.84382 | 0.82533 |
| 391.081 | 0.81583 | 0.86612 | 0.70978 | 0.8373 | 0.82758 |
| 391.858 | 0.81127 | 0.86254 | 0.70763 | 0.83288 | 0.82906 |
| 392.636 | 0.80983 | 0.85568 | 0.6984 | 0.83508 | 0.82694 |
| 393.414 | 0.8031 | 0.85726 | 0.69276 | 0.82949 | 0.81781 |
| 394.192 | 0.79822 | 0.85427 | 0.68949 | 0.82705 | 0.82146 |
| 394.969 | 0.7921 | 0.85525 | 0.68803 | 0.82687 | 0.80683 |
| 395.747 | 0.79871 | 0.85334 | 0.6856 | 0.82941 | 0.80899 |
| 396.524 | 0.79368 | 0.85184 | 0.68826 | 0.82816 | 0.80665 |
| 397.302 | 0.80314 | 0.83966 | 0.68499 | 0.82233 | 0.80553 |
| 398.079 | 0.79621 | 0.83128 | 0.68481 | 0.81675 | 0.79821 |
| 398.856 | 0.79477 | 0.82515 | 0.68012 | 0.81312 | 0.80306 |
| 399.634 | 0.79021 | 0.8236 | 0.67648 | 0.80343 | 0.8025 |
| 400.411 | 0.7875 | 0.81947 | 0.67208 | 0.79286 | 0.80374 |
| 401.188 | 0.78371 | 0.81956 | 0.6693 | 0.79256 | 0.80368 |
| 401.965 | 0.78666 | 0.81975 | 0.66109 | 0.79226 | 0.80102 |
| 402.742 | 0.78445 | 0.81888 | 0.65699 | 0.78351 | 0.79522 |
| 403.519 | 0.77959 | 0.81635 | 0.64839 | 0.78436 | 0.78334 |
| 404.296 | 0.78162 | 0.81373 | 0.64107 | 0.78591 | 0.77858 |
| 405.072 | 0.77672 | 0.81146 | 0.63625 | 0.78168 | 0.77493 |
| 405.849 | 0.77306 | 0.80663 | 0.63386 | 0.77361 | 0.76877 |
| 406.626 | 0.76857 | 0.7977 | 0.62718 | 0.76872 | 0.76179 |
| 407.402 | 0.7637 | 0.78976 | 0.62281 | 0.75886 | 0.75977 |
| 408.179 | 0.75892 | 0.78304 | 0.61791 | 0.74918 | 0.75607 |
| 408.955 | 0.75218 | 0.78094 | 0.61193 | 0.74158 | 0.74084 |
| 409.732 | 0.74834 | 0.77711 | 0.60695 | 0.73286 | 0.7384 |
| 410.508 | 0.74385 | 0.77502 | 0.60507 | 0.72587 | 0.73118 |
| 411.284 | 0.73798 | 0.77086 | 0.5989 | 0.71835 | 0.71896 |
| 412.06 | 0.73102 | 0.76637 | 0.59518 | 0.71032 | 0.70962 |
| 412.836 | 0.72916 | 0.75489 | 0.58723 | 0.69713 | 0.703 |
| 413.612 | 0.72209 | 0.74992 | 0.57939 | 0.69008 | 0.69377 |
| 414.388 | 0.71611 | 0.74211 | 0.56679 | 0.68383 | 0.68368 |
| 415.164 | 0.70945 | 0.73507 | 0.55834 | 0.67483 | 0.67017 |
| 415.94 | 0.7062 | 0.72735 | 0.5489 | 0.66762 | 0.65582 |
| 416.716 | 0.69578 | 0.71847 | 0.53929 | 0.66026 | 0.64205 |
| 417.492 | 0.68724 | 0.70834 | 0.52656 | 0.64852 | 0.62215 |
| 418.267 | 0.67638 | 0.70229 | 0.51837 | 0.63673 | 0.60562 |
| 419.043 | 0.67304 | 0.69089 | 0.50752 | 0.62493 | 0.59325 |
| 419.818 | 0.66227 | 0.67602 | 0.49509 | 0.6085 | 0.58223 |
| 420.594 | 0.65304 | 0.66603 | 0.48463 | 0.59419 | 0.57209 |
| 421.369 | 0.64252 | 0.65568 | 0.47821 | 0.58039 | 0.56048 |
| 422.144 | 0.63983 | 0.64272 | 0.46883 | 0.56475 | 0.55458 |
| 422.92 | 0.62772 | 0.63083 | 0.4602 | 0.54919 | 0.54314 |
| 423.695 | 0.61922 | 0.62341 | 0.45068 | 0.53694 | 0.5282 |
| 424.47 | 0.61057 | 0.6129 | 0.44082 | 0.52765 | 0.51308 |
| 425.245 | 0.60356 | 0.60009 | 0.42645 | 0.51095 | 0.49929 |
| 426.02 | 0.59305 | 0.58771 | 0.41198 | 0.49551 | 0.48068 |
| 426.795 | 0.58447 | 0.57744 | 0.40049 | 0.47773 | 0.4651 |
| 427.57 | 0.57233 | 0.56587 | 0.38757 | 0.46469 | 0.44589 |
| 428.345 | 0.56309 | 0.55634 | 0.37658 | 0.44694 | 0.42992 |
| 429.119 | 0.55335 | 0.54509 | 0.36716 | 0.43571 | 0.41782 |
| 429.894 | 0.54174 | 0.53487 | 0.35891 | 0.42446 | 0.40404 |
| 430.669 | 0.52961 | 0.52417 | 0.3494 | 0.41701 | 0.3917 |
| 431.443 | 0.52239 | 0.51353 | 0.34101 | 0.4045 | 0.38221 |
| 432.217 | 0.51272 | 0.50184 | 0.33368 | 0.3986 | 0.37166 |
| 432.992 | 0.50587 | 0.49137 | 0.32585 | 0.38817 | 0.35871 |
| 433.766 | 0.50008 | 0.47889 | 0.319 | 0.37804 | 0.35238 |
| 434.54 | 0.49399 | 0.47028 | 0.31224 | 0.36785 | 0.34299 |
| 435.315 | 0.48602 | 0.46122 | 0.30654 | 0.35993 | 0.3346 |
| 436.089 | 0.47951 | 0.45367 | 0.29841 | 0.34795 | 0.32821 |
| 436.863 | 0.47286 | 0.44654 | 0.29197 | 0.3413 | 0.32034 |
| 437.637 | 0.46492 | 0.44077 | 0.28563 | 0.33326 | 0.31149 |
| 438.411 | 0.45987 | 0.43131 | 0.28035 | 0.3248 | 0.306 |
| 439.185 | 0.45416 | 0.42565 | 0.2761 | 0.31874 | 0.299 |
| 439.958 | 0.44775 | 0.41716 | 0.27062 | 0.31243 | 0.29282 |
| 440.732 | 0.44314 | 0.40941 | 0.26637 | 0.30495 | 0.29161 |
| 441.506 | 0.43596 | 0.40379 | 0.25992 | 0.30284 | 0.28573 |
| 442.279 | 0.42819 | 0.39979 | 0.25429 | 0.29722 | 0.28003 |
| 443.053 | 0.42161 | 0.39271 | 0.2479 | 0.29399 | 0.27661 |
| 443.826 | 0.41639 | 0.3874 | 0.2434 | 0.28968 | 0.26985 |
| 444.6 | 0.40865 | 0.38285 | 0.23855 | 0.28831 | 0.26527 |
| 445.373 | 0.40394 | 0.37651 | 0.23643 | 0.28098 | 0.26088 |
| 446.146 | 0.4002 | 0.36973 | 0.23399 | 0.27705 | 0.25945 |
| 446.919 | 0.39772 | 0.36709 | 0.23128 | 0.27227 | 0.25826 |
| 447.693 | 0.3946 | 0.36125 | 0.22747 | 0.26918 | 0.25965 |
| 448.466 | 0.38959 | 0.35625 | 0.2249 | 0.26336 | 0.25686 |
| 449.239 | 0.38293 | 0.351 | 0.22078 | 0.25965 | 0.25472 |
| 450.012 | 0.37626 | 0.34757 | 0.21494 | 0.26007 | 0.25075 |
| 450.784 | 0.3689 | 0.34179 | 0.21001 | 0.25515 | 0.2451 |
| 451.557 | 0.36235 | 0.33838 | 0.20785 | 0.24978 | 0.23993 |
| 452.33 | 0.35789 | 0.33303 | 0.20155 | 0.24761 | 0.23568 |
| 453.103 | 0.35544 | 0.32731 | 0.19606 | 0.24424 | 0.23262 |
| 453.875 | 0.35227 | 0.32117 | 0.19143 | 0.2379 | 0.22929 |
| 454.648 | 0.348 | 0.31506 | 0.1885 | 0.23289 | 0.22912 |
| 455.42 | 0.34052 | 0.30778 | 0.18382 | 0.22811 | 0.22501 |
| 456.192 | 0.33219 | 0.30117 | 0.18137 | 0.22259 | 0.2191 |
| 456.965 | 0.32636 | 0.29546 | 0.17812 | 0.21828 | 0.21576 |
| 457.737 | 0.31921 | 0.28759 | 0.17719 | 0.21138 | 0.21213 |
| 458.509 | 0.31429 | 0.28196 | 0.17192 | 0.20882 | 0.20767 |
| 459.281 | 0.31015 | 0.27514 | 0.16689 | 0.20487 | 0.20274 |
| 460.053 | 0.30702 | 0.26912 | 0.16086 | 0.20014 | 0.20119 |
| 460.825 | 0.30153 | 0.26218 | 0.15608 | 0.19688 | 0.19896 |
| 461.597 | 0.2961 | 0.25664 | 0.14954 | 0.19383 | 0.19662 |
| 462.369 | 0.28942 | 0.24837 | 0.14554 | 0.1878 | 0.19407 |
| 463.141 | 0.28264 | 0.24124 | 0.14068 | 0.18258 | 0.19264 |
| 463.913 | 0.27412 | 0.23253 | 0.13668 | 0.178 | 0.19025 |
| 464.684 | 0.26761 | 0.22531 | 0.13234 | 0.1701 | 0.18469 |
| 465.456 | 0.26132 | 0.21853 | 0.1276 | 0.16436 | 0.17922 |
| 466.227 | 0.25459 | 0.20992 | 0.12087 | 0.15895 | 0.17484 |
| 466.999 | 0.24795 | 0.20372 | 0.11518 | 0.15412 | 0.1707 |
| 467.77 | 0.24328 | 0.19801 | 0.11036 | 0.1483 | 0.16559 |
| 468.541 | 0.23462 | 0.19142 | 0.10493 | 0.14475 | 0.16011 |
| 469.313 | 0.2274 | 0.18402 | 0.10017 | 0.14052 | 0.15698 |
| 470.084 | 0.21939 | 0.17645 | 0.09679 | 0.13398 | 0.15135 |
| 470.855 | 0.21378 | 0.16969 | 0.09273 | 0.12965 | 0.1475 |
| 471.626 | 0.20646 | 0.16246 | 0.08708 | 0.12564 | 0.14348 |
| 472.397 | 0.202 | 0.15521 | 0.08361 | 0.12093 | 0.14224 |
| 473.168 | 0.19762 | 0.14789 | 0.07854 | 0.11602 | 0.13991 |
| 473.939 | 0.19207 | 0.14263 | 0.07439 | 0.11376 | 0.13649 |
| 474.71 | 0.18497 | 0.13557 | 0.07032 | 0.10943 | 0.13421 |
| 475.48 | 0.17768 | 0.12785 | 0.06678 | 0.10501 | 0.13065 |
| 476.251 | 0.17181 | 0.11985 | 0.06172 | 0.1006 | 0.12841 |
| 477.021 | 0.16348 | 0.11406 | 0.05888 | 0.09708 | 0.12233 |
| 477.792 | 0.1575 | 0.1075 | 0.05404 | 0.09329 | 0.12114 |
| 478.562 | 0.15157 | 0.09966 | 0.05043 | 0.08916 | 0.11809 |
| 479.333 | 0.14573 | 0.09346 | 0.04787 | 0.08515 | 0.11656 |
| 480.103 | 0.13836 | 0.08879 | 0.04452 | 0.08307 | 0.11454 |
| 480.873 | 0.13218 | 0.08128 | 0.04033 | 0.08008 | 0.11386 |
| 481.643 | 0.12609 | 0.07478 | 0.03711 | 0.07686 | 0.11062 |
| 482.414 | 0.11913 | 0.06952 | 0.03376 | 0.07413 | 0.10808 |
| 483.184 | 0.1128 | 0.06279 | 0.02946 | 0.07086 | 0.10873 |
| 483.954 | 0.10571 | 0.05704 | 0.02646 | 0.06772 | 0.10668 |
| 484.723 | 0.10047 | 0.05173 | 0.02294 | 0.06713 | 0.10601 |
| 485.493 | 0.09466 | 0.04701 | 0.02076 | 0.06576 | 0.10568 |
| 486.263 | 0.09008 | 0.04271 | 0.01868 | 0.06512 | 0.10503 |
| 487.033 | 0.0853 | 0.03958 | 0.01675 | 0.06554 | 0.10183 |
| 487.802 | 0.08256 | 0.03547 | 0.01488 | 0.06377 | 0.09962 |
| 488.572 | 0.07821 | 0.03283 | 0.01348 | 0.05873 | 0.09998 |
| 489.341 | 0.07457 | 0.02987 | 0.01193 | 0.05823 | 0.09914 |
| 490.111 | 0.07047 | 0.02735 | 0.0105 | 0.05681 | 0.09972 |
| 490.88 | 0.06666 | 0.0244 | 0.00841 | 0.05529 | 0.10076 |
| 491.649 | 0.06288 | 0.02233 | 0.00639 | 0.0559 | 0.10177 |
| 492.419 | 0.0583 | 0.01915 | 0.00511 | 0.05909 | 0.09994 |
| 493.188 | 0.05425 | 0.01648 | 0.00388 | 0.05793 | 0.10284 |
| 493.957 | 0.05103 | 0.01476 | 0.00253 | 0.05839 | 0.1051 |
| 494.726 | 0.04782 | 0.01371 | 0.00224 | 0.05728 | 0.10525 |
| 495.495 | 0.04318 | 0.01074 | 0.00206 | 0.05521 | 0.10658 |
| 496.264 | 0.04098 | 0.00987 | 0.0018 | 0.05464 | 0.10903 |
| 497.032 | 0.03738 | 0.00923 | 0.00127 | 0.05685 | 0.1093 |
| 497.801 | 0.03456 | 0.00724 | 3.20E-04 | 0.05634 | 0.10969 |
| 498.57 | 0.03114 | 0.00567 | 3.16E-04 | 0.0567 | 0.11044 |
| 499.339 | 0.0292 | 0.00561 | 0 | 0.05991 | 0.1117 |
| 500.107 | 0.02673 | 0.00432 | 8.81E-04 | 0.05951 | 0.1121 |
| 500.876 | 0.02564 | 0.00379 | 1.29E-04 | 0.05861 | 0.11201 |
| 501.644 | 0.0237 | 0.00332 | 9.00E-04 | 0.06136 | 0.11265 |
| 502.412 | 0.02325 | 0.00349 | 0.00108 | 0.06353 | 0.11409 |
| 503.181 | 0.02092 | 0.0036 | 0.00183 | 0.06429 | 0.11539 |
| 503.949 | 0.01995 | 0.00292 | 9.06E-04 | 0.06637 | 0.11783 |
| 504.717 | 0.01718 | 0.00226 | 0.00162 | 0.06801 | 0.1196 |
| 505.485 | 0.01542 | 0.00119 | 0.00177 | 0.06758 | 0.12145 |
| 506.253 | 0.01347 | 1.40E-04 | 0.00222 | 0.06867 | 0.12457 |
| 507.021 | 0.01275 | 0 | 0.00261 | 0.07043 | 0.12647 |
| 507.789 | 0.0107 | 7.97E-04 | 0.00332 | 0.07166 | 0.12736 |
| 508.556 | 0.01069 | 0.00124 | 0.00435 | 0.07346 | 0.13161 |
| 509.324 | 0.00895 | 0.00238 | 0.00539 | 0.07577 | 0.13268 |
| 510.092 | 0.0077 | 0.0037 | 0.00595 | 0.07738 | 0.13299 |
| 510.859 | 0.00693 | 0.00315 | 0.0073 | 0.07753 | 0.13799 |
| 511.627 | 0.0047 | 0.00346 | 0.00911 | 0.08079 | 0.14088 |
| 512.394 | 0.00278 | 0.00407 | 0.01001 | 0.082 | 0.14244 |
| 513.162 | 0.00256 | 0.00509 | 0.01085 | 0.0845 | 0.1442 |
| 513.929 | 0.00224 | 0.00598 | 0.01219 | 0.08702 | 0.14744 |
| 514.696 | 0.00107 | 0.00799 | 0.01335 | 0.08878 | 0.14644 |
| 515.463 | 0.00235 | 0.00902 | 0.01405 | 0.09024 | 0.14849 |
| 516.231 | 0.00262 | 0.00958 | 0.01564 | 0.09202 | 0.14949 |
| 516.998 | 0.00212 | 0.01032 | 0.01749 | 0.09351 | 0.15149 |
| 517.765 | 9.90E-04 | 0.01055 | 0.01907 | 0.09495 | 0.15279 |
| 518.531 | 0.00167 | 0.01155 | 0.01956 | 0.09812 | 0.15705 |
| 519.298 | 8.51E-04 | 0.01331 | 0.0216 | 0.09902 | 0.15959 |
| 520.065 | 5.85E-04 | 0.01378 | 0.02323 | 0.10148 | 0.16075 |
| 520.832 | 0 | 0.0151 | 0.02442 | 0.10338 | 0.16487 |
| 521.598 | 0.00159 | 0.01742 | 0.02611 | 0.10631 | 0.16784 |
| 522.365 | 0.002 | 0.01907 | 0.02776 | 0.10904 | 0.16923 |
| 523.131 | 0.00299 | 0.01966 | 0.02897 | 0.11095 | 0.1718 |
| 523.898 | 0.00361 | 0.02198 | 0.03081 | 0.11267 | 0.17389 |
| 524.664 | 0.00581 | 0.02433 | 0.03286 | 0.11509 | 0.17482 |
| 525.43 | 0.00609 | 0.02636 | 0.03454 | 0.11775 | 0.17695 |
| 526.197 | 0.00611 | 0.0282 | 0.03768 | 0.12016 | 0.17874 |
| 526.963 | 0.00643 | 0.03017 | 0.03959 | 0.12242 | 0.17897 |
| 527.729 | 0.00711 | 0.03226 | 0.04156 | 0.12557 | 0.1808 |
| 528.495 | 0.00712 | 0.03347 | 0.04353 | 0.12757 | 0.18258 |
| 529.261 | 0.0074 | 0.03433 | 0.0451 | 0.12822 | 0.18569 |
| 530.027 | 0.00805 | 0.03656 | 0.04649 | 0.1314 | 0.18722 |
| 530.792 | 0.00936 | 0.03866 | 0.04844 | 0.13427 | 0.19001 |
| 531.558 | 0.01112 | 0.04124 | 0.05082 | 0.13554 | 0.19448 |
| 532.324 | 0.0118 | 0.0435 | 0.0532 | 0.13811 | 0.19587 |
| 533.089 | 0.01385 | 0.04547 | 0.05656 | 0.14115 | 0.19694 |
| 533.855 | 0.01592 | 0.04752 | 0.05903 | 0.14338 | 0.19896 |
| 534.62 | 0.01698 | 0.05051 | 0.06153 | 0.14661 | 0.20036 |
| 535.386 | 0.01732 | 0.05242 | 0.06259 | 0.14986 | 0.20115 |
| 536.151 | 0.01914 | 0.05576 | 0.06474 | 0.1528 | 0.20365 |
| 536.916 | 0.02018 | 0.05878 | 0.06653 | 0.15585 | 0.20609 |
| 537.682 | 0.02166 | 0.06151 | 0.06827 | 0.15807 | 0.20796 |
| 538.447 | 0.02325 | 0.06398 | 0.0713 | 0.15968 | 0.20976 |
| 539.212 | 0.02587 | 0.06688 | 0.07502 | 0.16322 | 0.21371 |
| 539.977 | 0.02901 | 0.06927 | 0.07764 | 0.1667 | 0.21837 |
| 540.742 | 0.03172 | 0.07237 | 0.08089 | 0.16939 | 0.21992 |
| 541.506 | 0.03352 | 0.07591 | 0.08443 | 0.17212 | 0.22218 |
| 542.271 | 0.03583 | 0.08029 | 0.08698 | 0.17545 | 0.22645 |
| 543.036 | 0.03781 | 0.08427 | 0.08911 | 0.17934 | 0.22856 |
| 543.801 | 0.03954 | 0.08783 | 0.09154 | 0.18253 | 0.22969 |
| 544.565 | 0.04162 | 0.09243 | 0.09461 | 0.18658 | 0.23203 |
| 545.33 | 0.04568 | 0.09672 | 0.09809 | 0.18896 | 0.2358 |
| 546.094 | 0.04873 | 0.10073 | 0.10127 | 0.19366 | 0.23902 |
| 546.858 | 0.05236 | 0.10445 | 0.10491 | 0.19706 | 0.23915 |
| 547.623 | 0.05618 | 0.1083 | 0.109 | 0.19979 | 0.24244 |
| 548.387 | 0.06096 | 0.11276 | 0.11221 | 0.20324 | 0.24607 |
| 549.151 | 0.06388 | 0.11676 | 0.11568 | 0.20762 | 0.24795 |
| 549.915 | 0.06804 | 0.11985 | 0.11877 | 0.20923 | 0.25178 |
| 550.679 | 0.07157 | 0.12538 | 0.12227 | 0.21097 | 0.25648 |
| 551.443 | 0.07503 | 0.13177 | 0.12571 | 0.21402 | 0.25981 |
| 552.207 | 0.07837 | 0.13626 | 0.12991 | 0.21763 | 0.26426 |
| 552.971 | 0.08307 | 0.14106 | 0.13313 | 0.22173 | 0.26987 |
| 553.734 | 0.0869 | 0.14781 | 0.13784 | 0.22725 | 0.27193 |
| 554.498 | 0.09138 | 0.15231 | 0.14155 | 0.23222 | 0.27632 |
| 555.262 | 0.09562 | 0.15825 | 0.14567 | 0.23732 | 0.27908 |
| 556.025 | 0.09961 | 0.1653 | 0.14879 | 0.24107 | 0.28268 |
| 556.789 | 0.10393 | 0.17173 | 0.15311 | 0.24506 | 0.28541 |
| 557.552 | 0.10914 | 0.17788 | 0.15717 | 0.24819 | 0.2885 |
| 558.315 | 0.11462 | 0.18501 | 0.1614 | 0.25168 | 0.2918 |
| 559.078 | 0.11989 | 0.19078 | 0.16578 | 0.25637 | 0.29752 |
| 559.842 | 0.12627 | 0.19668 | 0.17125 | 0.2617 | 0.30089 |
| 560.605 | 0.13229 | 0.20341 | 0.1759 | 0.26584 | 0.30509 |
| 561.368 | 0.13848 | 0.20932 | 0.18095 | 0.27178 | 0.3108 |
| 562.131 | 0.14375 | 0.21648 | 0.18615 | 0.27788 | 0.31336 |
| 562.894 | 0.14997 | 0.22362 | 0.19173 | 0.28315 | 0.31745 |
| 563.656 | 0.15582 | 0.23054 | 0.19616 | 0.2877 | 0.32249 |
| 564.419 | 0.16147 | 0.23798 | 0.20123 | 0.29295 | 0.32673 |
| 565.182 | 0.16731 | 0.24611 | 0.20586 | 0.29856 | 0.33207 |
| 565.945 | 0.17451 | 0.25375 | 0.21147 | 0.30341 | 0.33968 |
| 566.707 | 0.18092 | 0.26199 | 0.21614 | 0.30848 | 0.34431 |
| 567.47 | 0.18778 | 0.27105 | 0.22182 | 0.3141 | 0.34997 |
| 568.232 | 0.19457 | 0.27926 | 0.22728 | 0.3213 | 0.35576 |
| 568.994 | 0.20232 | 0.28791 | 0.23282 | 0.32789 | 0.3585 |
| 569.757 | 0.21001 | 0.2965 | 0.23927 | 0.33456 | 0.36332 |
| 570.519 | 0.21796 | 0.30598 | 0.24498 | 0.34243 | 0.36946 |
| 571.281 | 0.22515 | 0.31419 | 0.2508 | 0.34882 | 0.37405 |
| 572.043 | 0.23323 | 0.32238 | 0.25714 | 0.35397 | 0.37894 |
| 572.805 | 0.24041 | 0.33123 | 0.26342 | 0.3599 | 0.38622 |
| 573.567 | 0.24754 | 0.34021 | 0.26906 | 0.36711 | 0.39242 |
| 574.329 | 0.25544 | 0.34712 | 0.27541 | 0.37149 | 0.39764 |
| 575.09 | 0.26345 | 0.35598 | 0.28226 | 0.3791 | 0.40382 |
| 575.852 | 0.27225 | 0.36613 | 0.28793 | 0.38726 | 0.41083 |
| 576.614 | 0.2806 | 0.37531 | 0.29481 | 0.39526 | 0.4165 |
| 577.375 | 0.28892 | 0.38472 | 0.30141 | 0.40141 | 0.42241 |
| 578.137 | 0.29746 | 0.39495 | 0.30865 | 0.40879 | 0.42931 |
| 578.898 | 0.30668 | 0.40434 | 0.31478 | 0.41547 | 0.43573 |
| 579.66 | 0.31483 | 0.41424 | 0.32286 | 0.42243 | 0.44246 |
| 580.421 | 0.32384 | 0.425 | 0.32976 | 0.42989 | 0.44977 |
| 581.182 | 0.33313 | 0.43535 | 0.3366 | 0.43937 | 0.45702 |
| 581.943 | 0.34299 | 0.44551 | 0.34421 | 0.44707 | 0.46549 |
| 582.704 | 0.35257 | 0.45659 | 0.35206 | 0.45605 | 0.47364 |
| 583.465 | 0.36197 | 0.46658 | 0.35843 | 0.46457 | 0.48253 |
| 584.226 | 0.37199 | 0.47674 | 0.36518 | 0.47187 | 0.49135 |
| 584.987 | 0.38231 | 0.48675 | 0.37334 | 0.4787 | 0.50022 |
| 585.748 | 0.39133 | 0.49711 | 0.38056 | 0.48753 | 0.50658 |
| 586.509 | 0.40053 | 0.5076 | 0.38806 | 0.49547 | 0.51486 |
| 587.269 | 0.41029 | 0.51686 | 0.39687 | 0.5031 | 0.52189 |
| 588.03 | 0.41873 | 0.52614 | 0.40601 | 0.51032 | 0.528 |
| 588.79 | 0.42652 | 0.53541 | 0.41304 | 0.5167 | 0.53389 |
| 589.551 | 0.43592 | 0.54532 | 0.42113 | 0.52384 | 0.54122 |
| 590.311 | 0.44579 | 0.55388 | 0.42928 | 0.53296 | 0.54856 |
| 591.072 | 0.45537 | 0.56517 | 0.43635 | 0.54293 | 0.55746 |
| 591.832 | 0.4659 | 0.57556 | 0.44374 | 0.55298 | 0.56697 |
| 592.592 | 0.47592 | 0.58386 | 0.45321 | 0.56319 | 0.57356 |
| 593.352 | 0.48531 | 0.59445 | 0.46044 | 0.57255 | 0.58228 |
| 594.112 | 0.49468 | 0.60539 | 0.46814 | 0.5809 | 0.59121 |
| 594.872 | 0.50536 | 0.61454 | 0.47633 | 0.5897 | 0.59881 |
| 595.632 | 0.5147 | 0.62407 | 0.48395 | 0.59958 | 0.6072 |
| 596.392 | 0.52593 | 0.63523 | 0.49118 | 0.60856 | 0.61818 |
| 597.151 | 0.53633 | 0.64304 | 0.49956 | 0.61806 | 0.62711 |
| 597.911 | 0.54485 | 0.65055 | 0.50721 | 0.62645 | 0.63487 |
| 598.671 | 0.55312 | 0.65944 | 0.51355 | 0.63436 | 0.64327 |
| 599.43 | 0.56246 | 0.66816 | 0.52197 | 0.64096 | 0.65137 |
| 600.19 | 0.57123 | 0.67809 | 0.52771 | 0.6501 | 0.65869 |
| 600.949 | 0.58 | 0.68759 | 0.53458 | 0.65925 | 0.66736 |
| 601.708 | 0.58951 | 0.69756 | 0.54194 | 0.66777 | 0.67529 |
| 602.468 | 0.59779 | 0.70634 | 0.55142 | 0.67621 | 0.68053 |
| 603.227 | 0.60756 | 0.715 | 0.55841 | 0.68632 | 0.69003 |
| 603.986 | 0.61423 | 0.72384 | 0.56665 | 0.69433 | 0.69843 |
| 604.745 | 0.62291 | 0.73274 | 0.57376 | 0.70269 | 0.70661 |
| 605.504 | 0.63171 | 0.74099 | 0.58189 | 0.71111 | 0.71438 |
| 606.263 | 0.64118 | 0.74877 | 0.58861 | 0.71927 | 0.72421 |
| 607.022 | 0.64955 | 0.75625 | 0.59579 | 0.72509 | 0.73122 |
| 607.78 | 0.65939 | 0.76162 | 0.60412 | 0.73275 | 0.74101 |
| 608.539 | 0.66843 | 0.76903 | 0.61168 | 0.74031 | 0.74974 |
| 609.298 | 0.676 | 0.77586 | 0.61827 | 0.74842 | 0.75662 |
| 610.056 | 0.68358 | 0.78357 | 0.62548 | 0.75625 | 0.76538 |
| 610.815 | 0.69074 | 0.79035 | 0.63199 | 0.7651 | 0.77396 |
| 611.573 | 0.6988 | 0.79765 | 0.63745 | 0.77257 | 0.78133 |
| 612.332 | 0.70502 | 0.80546 | 0.64565 | 0.78099 | 0.78742 |
| 613.09 | 0.71367 | 0.81284 | 0.65206 | 0.78798 | 0.79715 |
| 613.848 | 0.72138 | 0.81923 | 0.65897 | 0.79558 | 0.8053 |
| 614.606 | 0.72832 | 0.82648 | 0.66547 | 0.80514 | 0.81229 |
| 615.364 | 0.735 | 0.83393 | 0.67331 | 0.81409 | 0.8186 |
| 616.122 | 0.74169 | 0.84042 | 0.67857 | 0.82193 | 0.82518 |
| 616.88 | 0.74733 | 0.84716 | 0.68592 | 0.82965 | 0.83074 |
| 617.638 | 0.75303 | 0.85349 | 0.69165 | 0.83637 | 0.83716 |
| 618.396 | 0.75948 | 0.85815 | 0.69909 | 0.84182 | 0.84236 |
| 619.153 | 0.76731 | 0.8634 | 0.70484 | 0.84633 | 0.84914 |
| 619.911 | 0.7745 | 0.86772 | 0.71099 | 0.85201 | 0.85582 |
| 620.668 | 0.78245 | 0.87149 | 0.71512 | 0.85679 | 0.86338 |
| 621.426 | 0.78833 | 0.87771 | 0.72094 | 0.86384 | 0.86841 |
| 622.183 | 0.79364 | 0.88344 | 0.72621 | 0.86855 | 0.87347 |
| 622.941 | 0.79657 | 0.88989 | 0.73114 | 0.87605 | 0.87791 |
| 623.698 | 0.80146 | 0.89399 | 0.73672 | 0.88107 | 0.88271 |
| 624.455 | 0.80526 | 0.89928 | 0.74331 | 0.8875 | 0.88776 |
| 625.212 | 0.80944 | 0.90098 | 0.74762 | 0.89176 | 0.8917 |
| 625.969 | 0.81448 | 0.90574 | 0.75137 | 0.89734 | 0.89771 |
| 626.726 | 0.81949 | 0.90933 | 0.75671 | 0.9024 | 0.90324 |
| 627.483 | 0.82378 | 0.91254 | 0.76075 | 0.90586 | 0.90825 |
| 628.24 | 0.82757 | 0.91593 | 0.76508 | 0.9102 | 0.91282 |
| 628.997 | 0.83352 | 0.9212 | 0.77068 | 0.9153 | 0.91765 |
| 629.753 | 0.83924 | 0.92498 | 0.77639 | 0.91903 | 0.92248 |
| 630.51 | 0.84441 | 0.92839 | 0.77971 | 0.92436 | 0.9248 |
| 631.267 | 0.84796 | 0.93195 | 0.78354 | 0.92972 | 0.92898 |
| 632.023 | 0.85136 | 0.93583 | 0.78735 | 0.93524 | 0.93211 |
| 632.78 | 0.85497 | 0.93824 | 0.79094 | 0.93933 | 0.93723 |
| 633.536 | 0.8568 | 0.94126 | 0.79274 | 0.94447 | 0.94141 |
| 634.292 | 0.8598 | 0.9438 | 0.79786 | 0.94792 | 0.94823 |
| 635.048 | 0.86464 | 0.94718 | 0.80165 | 0.95375 | 0.95285 |
| 635.804 | 0.86981 | 0.949 | 0.8059 | 0.9572 | 0.95816 |
| 636.561 | 0.87347 | 0.95383 | 0.80905 | 0.96063 | 0.96222 |
| 637.316 | 0.87868 | 0.95391 | 0.81304 | 0.96243 | 0.96669 |
| 638.072 | 0.8831 | 0.95612 | 0.81566 | 0.96535 | 0.97079 |
| 638.828 | 0.88536 | 0.95811 | 0.82026 | 0.96873 | 0.97343 |
| 639.584 | 0.88749 | 0.96052 | 0.82192 | 0.97101 | 0.97497 |
| 640.34 | 0.88937 | 0.95964 | 0.82556 | 0.97393 | 0.97635 |
| 641.095 | 0.89108 | 0.96365 | 0.8285 | 0.97774 | 0.97549 |
| 641.851 | 0.89293 | 0.96718 | 0.83129 | 0.98126 | 0.97719 |
| 642.606 | 0.89416 | 0.96848 | 0.83289 | 0.98277 | 0.97788 |
| 643.362 | 0.89579 | 0.96941 | 0.836 | 0.98432 | 0.97979 |
| 644.117 | 0.89643 | 0.97174 | 0.83718 | 0.98742 | 0.98192 |
| 644.872 | 0.89741 | 0.97383 | 0.83972 | 0.98988 | 0.98641 |
| 645.628 | 0.89816 | 0.97155 | 0.84075 | 0.98932 | 0.98811 |
| 646.383 | 0.89872 | 0.97416 | 0.84157 | 0.98939 | 0.99121 |
| 647.138 | 0.90054 | 0.97578 | 0.84192 | 0.99259 | 0.9915 |
| 647.893 | 0.90165 | 0.97737 | 0.84343 | 0.99229 | 0.99182 |
| 648.648 | 0.9024 | 0.97788 | 0.84365 | 0.99237 | 0.99129 |
| 649.402 | 0.9028 | 0.98041 | 0.84546 | 0.99302 | 0.99109 |
| 650.157 | 0.9045 | 0.98187 | 0.84648 | 0.99555 | 0.9915 |
| 650.912 | 0.90376 | 0.98336 | 0.84704 | 0.99473 | 0.99388 |
| 651.667 | 0.90604 | 0.98279 | 0.84792 | 0.99524 | 0.994 |
| 652.421 | 0.90652 | 0.98202 | 0.84895 | 0.99558 | 0.99459 |
| 653.176 | 0.90831 | 0.98239 | 0.84939 | 0.99715 | 0.99497 |
| 653.93 | 0.90989 | 0.98156 | 0.85062 | 0.99716 | 0.99639 |
| 654.684 | 0.91032 | 0.98184 | 0.85111 | 0.99765 | 0.99463 |
| 655.439 | 0.91132 | 0.98348 | 0.85189 | 0.99674 | 0.99585 |
| 656.193 | 0.91166 | 0.98398 | 0.85243 | 0.99688 | 0.99723 |
| 656.947 | 0.91071 | 0.98348 | 0.85256 | 0.99751 | 0.99773 |
| 657.701 | 0.9119 | 0.98326 | 0.85134 | 0.99683 | 0.99649 |
| 658.455 | 0.91324 | 0.98256 | 0.85199 | 0.99746 | 0.9972 |
| 659.209 | 0.91261 | 0.98211 | 0.85084 | 0.9981 | 0.9968 |
| 659.963 | 0.91227 | 0.98354 | 0.85021 | 1 | 0.99626 |
| 660.716 | 0.91419 | 0.98448 | 0.85032 | 0.99989 | 0.99742 |
| 661.47 | 0.91378 | 0.98443 | 0.85087 | 0.99988 | 0.99736 |
| 662.224 | 0.91406 | 0.9853 | 0.85115 | 0.99893 | 0.99816 |
| 662.977 | 0.91456 | 0.98435 | 0.85057 | 0.99684 | 1 |
| 663.731 | 0.91467 | 0.98213 | 0.85063 | 0.99491 | 0.99997 |
| 664.484 | 0.91396 | 0.98254 | 0.85054 | 0.99451 | 0.99784 |
| 665.237 | 0.91296 | 0.98385 | 0.85038 | 0.99435 | 0.9963 |
| 665.991 | 0.91295 | 0.98289 | 0.84998 | 0.99476 | 0.99637 |
| 666.744 | 0.91013 | 0.98355 | 0.85106 | 0.99671 | 0.99278 |
| 667.497 | 0.90975 | 0.98561 | 0.85017 | 0.99704 | 0.99233 |
| 668.25 | 0.90779 | 0.98534 | 0.849 | 0.9968 | 0.99125 |
| 669.003 | 0.90678 | 0.98372 | 0.84719 | 0.99489 | 0.9916 |
| 669.756 | 0.90558 | 0.98378 | 0.84565 | 0.99181 | 0.98971 |
| 670.509 | 0.90626 | 0.98242 | 0.84451 | 0.9894 | 0.99047 |
| 671.261 | 0.90583 | 0.98133 | 0.84407 | 0.98912 | 0.98798 |
| 672.014 | 0.90444 | 0.97913 | 0.8435 | 0.98614 | 0.98539 |
| 672.767 | 0.905 | 0.97885 | 0.84375 | 0.98573 | 0.98305 |
| 673.519 | 0.9043 | 0.97836 | 0.84392 | 0.98712 | 0.98335 |
| 674.272 | 0.90468 | 0.97763 | 0.842 | 0.98573 | 0.98162 |
| 675.024 | 0.90287 | 0.97779 | 0.84074 | 0.984 | 0.97923 |
| 675.776 | 0.9027 | 0.97653 | 0.83857 | 0.98203 | 0.97862 |
| 676.529 | 0.90026 | 0.97522 | 0.83728 | 0.98152 | 0.97818 |
| 677.281 | 0.89966 | 0.97472 | 0.83509 | 0.97943 | 0.97407 |
| 678.033 | 0.89776 | 0.97466 | 0.83491 | 0.9806 | 0.97387 |
| 678.785 | 0.89932 | 0.97229 | 0.83448 | 0.97899 | 0.97525 |
| 679.537 | 0.8989 | 0.9732 | 0.83314 | 0.97988 | 0.97303 |
| 680.289 | 0.89896 | 0.97294 | 0.83111 | 0.97873 | 0.97132 |
| 681.041 | 0.89706 | 0.97173 | 0.83043 | 0.97776 | 0.97185 |
| 681.792 | 0.8956 | 0.97215 | 0.82904 | 0.97582 | 0.96837 |
| 682.544 | 0.89339 | 0.97373 | 0.82829 | 0.97573 | 0.96649 |
| 683.295 | 0.89374 | 0.97367 | 0.82913 | 0.97545 | 0.96775 |
| 684.047 | 0.89273 | 0.9718 | 0.82979 | 0.97154 | 0.96676 |
| 684.798 | 0.89246 | 0.97158 | 0.8288 | 0.96931 | 0.96385 |
| 685.55 | 0.89173 | 0.9699 | 0.82803 | 0.96788 | 0.96276 |
| 686.301 | 0.89182 | 0.96764 | 0.82613 | 0.96637 | 0.96114 |
| 687.052 | 0.89016 | 0.96636 | 0.82484 | 0.96232 | 0.95641 |
| 687.803 | 0.88967 | 0.96715 | 0.82351 | 0.9631 | 0.95479 |
| 688.555 | 0.88837 | 0.96688 | 0.82198 | 0.96256 | 0.95335 |
| 689.306 | 0.88799 | 0.96716 | 0.82134 | 0.96014 | 0.9527 |
| 690.057 | 0.88654 | 0.9667 | 0.82078 | 0.95836 | 0.94997 |
| 690.807 | 0.88644 | 0.96629 | 0.81898 | 0.95853 | 0.95056 |
| 691.558 | 0.88583 | 0.96461 | 0.81885 | 0.95667 | 0.95031 |
| 692.309 | 0.88501 | 0.96331 | 0.81924 | 0.95583 | 0.94761 |
| 693.06 | 0.88439 | 0.96198 | 0.81776 | 0.95609 | 0.94642 |
| 693.81 | 0.88262 | 0.96249 | 0.81697 | 0.95664 | 0.94628 |
| 694.561 | 0.8796 | 0.96084 | 0.81643 | 0.9551 | 0.94346 |
| 695.311 | 0.87763 | 0.96053 | 0.81393 | 0.9541 | 0.94149 |
| 696.061 | 0.87597 | 0.96103 | 0.81184 | 0.95272 | 0.94101 |
| 696.812 | 0.87459 | 0.95986 | 0.8113 | 0.95138 | 0.93807 |
| 697.562 | 0.87251 | 0.95795 | 0.80983 | 0.94793 | 0.93645 |
| 698.312 | 0.87378 | 0.95898 | 0.80834 | 0.9476 | 0.93663 |
| 699.062 | 0.87209 | 0.95923 | 0.8074 | 0.94857 | 0.93321 |
| 699.812 | 0.87076 | 0.95725 | 0.80677 | 0.94832 | 0.93261 |
| 700.562 | 0.86911 | 0.95496 | 0.805 | 0.94543 | 0.93183 |
| 701.312 | 0.86931 | 0.95313 | 0.80363 | 0.94501 | 0.92978 |
| 702.062 | 0.86637 | 0.95195 | 0.80341 | 0.94441 | 0.92855 |
| 702.811 | 0.86595 | 0.95045 | 0.80282 | 0.9394 | 0.92925 |
| 703.561 | 0.866 | 0.94844 | 0.80122 | 0.93572 | 0.92719 |
| 704.31 | 0.86575 | 0.94929 | 0.79962 | 0.93596 | 0.92655 |
| 705.06 | 0.86456 | 0.94914 | 0.7992 | 0.93409 | 0.92446 |
| 705.809 | 0.86419 | 0.94742 | 0.79668 | 0.93073 | 0.92245 |
| 706.559 | 0.86237 | 0.94553 | 0.795 | 0.93071 | 0.91959 |
| 707.308 | 0.861 | 0.94378 | 0.79293 | 0.92951 | 0.91853 |
| 708.057 | 0.85952 | 0.94275 | 0.79168 | 0.92535 | 0.91551 |
| 708.806 | 0.85926 | 0.94087 | 0.78983 | 0.92372 | 0.91397 |
| 709.555 | 0.85876 | 0.93862 | 0.78968 | 0.9221 | 0.91448 |
| 710.304 | 0.85696 | 0.93831 | 0.78776 | 0.92067 | 0.91356 |
| 711.053 | 0.85554 | 0.94049 | 0.78794 | 0.92109 | 0.91107 |
| 711.802 | 0.85313 | 0.93973 | 0.78689 | 0.92287 | 0.90866 |
| 712.551 | 0.85136 | 0.93894 | 0.78635 | 0.9203 | 0.90619 |
| 713.299 | 0.84884 | 0.93988 | 0.78562 | 0.91869 | 0.90132 |
| 714.048 | 0.84856 | 0.94035 | 0.78602 | 0.91765 | 0.89958 |
| 714.796 | 0.84826 | 0.93893 | 0.78485 | 0.91587 | 0.90021 |
| 715.545 | 0.84839 | 0.93771 | 0.7845 | 0.91389 | 0.90132 |
| 716.293 | 0.84825 | 0.93783 | 0.78414 | 0.91352 | 0.90162 |
| 717.041 | 0.84732 | 0.93646 | 0.78204 | 0.91249 | 0.90171 |
| 717.79 | 0.84728 | 0.93543 | 0.78077 | 0.91162 | 0.90153 |
| 718.538 | 0.84731 | 0.93463 | 0.78079 | 0.90973 | 0.89916 |
| 719.286 | 0.8475 | 0.93509 | 0.78073 | 0.91015 | 0.89705 |
| 720.034 | 0.84661 | 0.93556 | 0.78015 | 0.90977 | 0.89652 |
| 720.782 | 0.8465 | 0.93495 | 0.78039 | 0.90946 | 0.89601 |
| 721.53 | 0.84498 | 0.93409 | 0.78089 | 0.90881 | 0.89489 |
| 722.278 | 0.84328 | 0.93286 | 0.77949 | 0.90739 | 0.89417 |
| 723.025 | 0.84191 | 0.93076 | 0.77758 | 0.90534 | 0.89417 |
| 723.773 | 0.841 | 0.9291 | 0.77595 | 0.90306 | 0.89391 |
| 724.52 | 0.83911 | 0.92849 | 0.77455 | 0.90167 | 0.8915 |
| 725.268 | 0.83875 | 0.92821 | 0.77272 | 0.90107 | 0.88909 |
| 726.015 | 0.83812 | 0.92793 | 0.7715 | 0.89986 | 0.88717 |
| 726.763 | 0.83731 | 0.92745 | 0.77143 | 0.90048 | 0.88631 |
| 727.51 | 0.83671 | 0.92618 | 0.76979 | 0.90049 | 0.88235 |
| 728.257 | 0.83719 | 0.92473 | 0.76869 | 0.89904 | 0.882 |
| 729.004 | 0.83726 | 0.9229 | 0.76711 | 0.89609 | 0.88137 |
| 729.751 | 0.83541 | 0.92082 | 0.76703 | 0.89502 | 0.8783 |
| 730.498 | 0.83516 | 0.92025 | 0.76512 | 0.89078 | 0.87458 |
| 731.245 | 0.83337 | 0.91916 | 0.76452 | 0.88782 | 0.87436 |
| 731.992 | 0.83243 | 0.91795 | 0.76362 | 0.88552 | 0.87079 |
| 732.739 | 0.83012 | 0.91664 | 0.7621 | 0.88307 | 0.86709 |
| 733.485 | 0.82928 | 0.91648 | 0.76086 | 0.88248 | 0.86618 |
| 734.232 | 0.82661 | 0.91513 | 0.75967 | 0.88054 | 0.86547 |
| 734.979 | 0.82475 | 0.91475 | 0.75794 | 0.87946 | 0.86252 |
| 735.725 | 0.82365 | 0.9122 | 0.75623 | 0.87657 | 0.86224 |
| 736.471 | 0.82197 | 0.91008 | 0.75454 | 0.87558 | 0.86043 |
| 737.218 | 0.81944 | 0.90815 | 0.7515 | 0.87182 | 0.85931 |
| 737.964 | 0.81759 | 0.90552 | 0.75024 | 0.86786 | 0.85628 |
| 738.71 | 0.81639 | 0.90345 | 0.7485 | 0.86598 | 0.85465 |
| 739.456 | 0.81331 | 0.90331 | 0.747 | 0.86475 | 0.85119 |
| 740.202 | 0.81039 | 0.90091 | 0.74515 | 0.86103 | 0.84787 |
| 740.948 | 0.80792 | 0.89781 | 0.7436 | 0.85758 | 0.84458 |
| 741.694 | 0.80532 | 0.8957 | 0.74104 | 0.85622 | 0.84013 |
| 742.44 | 0.80125 | 0.89241 | 0.73813 | 0.85159 | 0.83581 |
| 743.186 | 0.79718 | 0.88926 | 0.73449 | 0.84798 | 0.83164 |
| 743.931 | 0.79396 | 0.88648 | 0.73203 | 0.84355 | 0.82823 |
| 744.677 | 0.79036 | 0.88379 | 0.72875 | 0.84053 | 0.82225 |
| 745.422 | 0.78766 | 0.88109 | 0.72651 | 0.83685 | 0.81868 |
| 746.168 | 0.78411 | 0.87796 | 0.72299 | 0.83389 | 0.81379 |
| 746.913 | 0.78077 | 0.8742 | 0.72067 | 0.82982 | 0.80893 |
| 747.658 | 0.77841 | 0.87222 | 0.71723 | 0.82742 | 0.80631 |
| 748.404 | 0.7736 | 0.86888 | 0.71366 | 0.82349 | 0.80256 |
| 749.149 | 0.76889 | 0.86528 | 0.70967 | 0.81846 | 0.79897 |
| 749.894 | 0.76494 | 0.86144 | 0.70653 | 0.8133 | 0.7947 |
| 750.639 | 0.76006 | 0.85791 | 0.70384 | 0.80838 | 0.79088 |
| 751.384 | 0.75457 | 0.85346 | 0.70024 | 0.80441 | 0.78414 |
| 752.128 | 0.75086 | 0.8495 | 0.6972 | 0.79982 | 0.78043 |
| 752.873 | 0.74572 | 0.8444 | 0.69288 | 0.79612 | 0.77452 |
| 753.618 | 0.74004 | 0.83957 | 0.68884 | 0.79078 | 0.76964 |
| 754.362 | 0.73555 | 0.83552 | 0.68431 | 0.78811 | 0.76514 |
| 755.107 | 0.72975 | 0.83156 | 0.6804 | 0.78292 | 0.76054 |
| 755.851 | 0.72445 | 0.82633 | 0.67554 | 0.77753 | 0.75488 |
| 756.596 | 0.71916 | 0.82274 | 0.67265 | 0.77234 | 0.75096 |
| 757.34 | 0.71487 | 0.81782 | 0.66922 | 0.7676 | 0.7476 |
| 758.084 | 0.70971 | 0.81208 | 0.66459 | 0.7616 | 0.74224 |
| 758.829 | 0.7059 | 0.80661 | 0.66081 | 0.75543 | 0.73799 |
| 759.573 | 0.70143 | 0.80143 | 0.65686 | 0.7503 | 0.73479 |
| 760.317 | 0.69551 | 0.79547 | 0.65214 | 0.74446 | 0.7307 |
| 761.061 | 0.6883 | 0.78979 | 0.64871 | 0.73979 | 0.72316 |
| 761.804 | 0.68282 | 0.78462 | 0.64465 | 0.73364 | 0.71898 |
| 762.548 | 0.67713 | 0.77912 | 0.6405 | 0.72971 | 0.7153 |
| 763.292 | 0.67086 | 0.77437 | 0.63707 | 0.72585 | 0.70884 |
| 764.036 | 0.66502 | 0.76982 | 0.63292 | 0.72265 | 0.70185 |
| 764.779 | 0.65878 | 0.76548 | 0.62814 | 0.71919 | 0.69864 |
| 765.523 | 0.65229 | 0.75991 | 0.62411 | 0.71492 | 0.69262 |
| 766.266 | 0.64576 | 0.7535 | 0.61919 | 0.70954 | 0.68646 |
| 767.009 | 0.63935 | 0.74736 | 0.6147 | 0.70471 | 0.68237 |
| 767.753 | 0.63365 | 0.7413 | 0.61036 | 0.69904 | 0.67825 |
| 768.496 | 0.62945 | 0.73421 | 0.60648 | 0.69305 | 0.67369 |
| 769.239 | 0.62334 | 0.72884 | 0.60226 | 0.68904 | 0.6686 |
| 769.982 | 0.61651 | 0.7233 | 0.59848 | 0.68454 | 0.66352 |
| 770.725 | 0.61022 | 0.71548 | 0.59489 | 0.67776 | 0.65755 |
| 771.468 | 0.60364 | 0.70879 | 0.59048 | 0.67177 | 0.65141 |
| 772.211 | 0.59786 | 0.70274 | 0.5861 | 0.66674 | 0.64599 |
| 772.953 | 0.5913 | 0.69529 | 0.58268 | 0.6604 | 0.6419 |
| 773.696 | 0.58542 | 0.68708 | 0.57854 | 0.65361 | 0.63736 |
| 774.439 | 0.5794 | 0.68178 | 0.57397 | 0.65057 | 0.63317 |
| 775.181 | 0.5742 | 0.67513 | 0.57026 | 0.64601 | 0.6298 |
| 775.924 | 0.56712 | 0.66898 | 0.56709 | 0.64123 | 0.62412 |
| 776.666 | 0.56162 | 0.66278 | 0.56319 | 0.63741 | 0.61898 |
| 777.408 | 0.55557 | 0.65673 | 0.56027 | 0.63452 | 0.61353 |
| 778.15 | 0.54991 | 0.65067 | 0.55639 | 0.62915 | 0.60904 |
| 778.893 | 0.54315 | 0.64402 | 0.55273 | 0.62462 | 0.60389 |
| 779.635 | 0.53731 | 0.63652 | 0.54909 | 0.61995 | 0.60042 |
| 780.377 | 0.53144 | 0.63009 | 0.54552 | 0.61512 | 0.59775 |
| 781.118 | 0.52506 | 0.62395 | 0.54117 | 0.61057 | 0.59409 |
| 781.86 | 0.51917 | 0.61616 | 0.53901 | 0.6055 | 0.59014 |
| 782.602 | 0.51355 | 0.60962 | 0.53586 | 0.60187 | 0.58594 |
| 783.344 | 0.50719 | 0.60428 | 0.53177 | 0.59702 | 0.5816 |
| 784.085 | 0.5012 | 0.59813 | 0.52879 | 0.59296 | 0.57734 |
| 784.827 | 0.49578 | 0.59204 | 0.52615 | 0.58811 | 0.57478 |
| 785.568 | 0.48994 | 0.58692 | 0.52185 | 0.58492 | 0.57018 |
| 786.31 | 0.48437 | 0.57943 | 0.51854 | 0.58071 | 0.56526 |
| 787.051 | 0.47977 | 0.57287 | 0.51625 | 0.57808 | 0.56047 |
| 787.792 | 0.47527 | 0.56644 | 0.51284 | 0.57351 | 0.55469 |
| 788.533 | 0.46962 | 0.56075 | 0.51012 | 0.56948 | 0.5485 |
| 789.275 | 0.46414 | 0.55498 | 0.50801 | 0.56604 | 0.54528 |
| 790.016 | 0.45954 | 0.54905 | 0.50594 | 0.56172 | 0.54364 |
| 790.757 | 0.45437 | 0.54351 | 0.50297 | 0.55821 | 0.54144 |
| 791.497 | 0.44885 | 0.53744 | 0.50047 | 0.55575 | 0.53967 |
| 792.238 | 0.44501 | 0.53064 | 0.49744 | 0.5526 | 0.53915 |
| 792.979 | 0.44112 | 0.52424 | 0.49436 | 0.54921 | 0.53587 |
| 793.719 | 0.43547 | 0.51823 | 0.49122 | 0.54619 | 0.53279 |
| 794.46 | 0.43117 | 0.51131 | 0.48854 | 0.54124 | 0.53047 |
| 795.201 | 0.42641 | 0.50551 | 0.48588 | 0.53838 | 0.52738 |
| 795.941 | 0.42163 | 0.50066 | 0.48402 | 0.53709 | 0.52219 |
| 796.681 | 0.41722 | 0.49564 | 0.4819 | 0.53413 | 0.5206 |
| 797.422 | 0.41279 | 0.4915 | 0.4793 | 0.53167 | 0.5167 |
| 798.162 | 0.40834 | 0.48755 | 0.47682 | 0.53072 | 0.51447 |
| 798.902 | 0.4041 | 0.48248 | 0.4747 | 0.52775 | 0.5118 |
| 799.642 | 0.39924 | 0.4769 | 0.47265 | 0.52438 | 0.51101 |
| 800.382 | 0.39449 | 0.47109 | 0.47004 | 0.52093 | 0.50766 |
| 801.122 | 0.3902 | 0.46627 | 0.46813 | 0.51788 | 0.50532 |
| 801.861 | 0.38593 | 0.46049 | 0.46587 | 0.51444 | 0.50269 |
| 802.601 | 0.38242 | 0.45604 | 0.46461 | 0.51309 | 0.50076 |
| 803.341 | 0.37911 | 0.45072 | 0.46269 | 0.51087 | 0.49826 |
| 804.08 | 0.37509 | 0.44609 | 0.46083 | 0.50898 | 0.49562 |
| 804.82 | 0.37156 | 0.44056 | 0.45905 | 0.50649 | 0.49506 |
| 805.559 | 0.36768 | 0.43635 | 0.45756 | 0.50344 | 0.49322 |
| 806.299 | 0.36371 | 0.43153 | 0.45514 | 0.50045 | 0.49085 |
| 807.038 | 0.36061 | 0.42763 | 0.45295 | 0.49764 | 0.48829 |
| 807.777 | 0.35709 | 0.42373 | 0.45202 | 0.49629 | 0.48627 |
| 808.516 | 0.35493 | 0.42065 | 0.45141 | 0.49463 | 0.484 |
| 809.255 | 0.35163 | 0.41585 | 0.44985 | 0.49332 | 0.48051 |
| 809.994 | 0.34836 | 0.41205 | 0.44826 | 0.49085 | 0.47886 |
| 810.733 | 0.34458 | 0.40951 | 0.44639 | 0.48996 | 0.47722 |
| 811.472 | 0.34218 | 0.40542 | 0.4446 | 0.48729 | 0.47591 |
| 812.211 | 0.33882 | 0.4009 | 0.44235 | 0.4851 | 0.47449 |
| 812.949 | 0.33668 | 0.39733 | 0.44152 | 0.48492 | 0.474 |
| 813.688 | 0.33392 | 0.39332 | 0.44049 | 0.48317 | 0.47216 |
| 814.427 | 0.33119 | 0.38853 | 0.43959 | 0.48101 | 0.47029 |
| 815.165 | 0.32882 | 0.38439 | 0.4387 | 0.47923 | 0.46943 |
| 815.903 | 0.32595 | 0.38024 | 0.43723 | 0.47899 | 0.46766 |
| 816.642 | 0.32307 | 0.37662 | 0.4365 | 0.47655 | 0.46665 |
| 817.38 | 0.32062 | 0.37355 | 0.43587 | 0.47552 | 0.46454 |
| 818.118 | 0.31835 | 0.37018 | 0.43586 | 0.47475 | 0.46348 |
| 818.856 | 0.31523 | 0.36767 | 0.43541 | 0.47252 | 0.46251 |
| 819.594 | 0.31311 | 0.36585 | 0.43532 | 0.47149 | 0.46159 |
| 820.332 | 0.31126 | 0.36408 | 0.43443 | 0.47143 | 0.45975 |
| 821.07 | 0.30945 | 0.36124 | 0.43323 | 0.47041 | 0.45889 |
| 821.808 | 0.30843 | 0.3589 | 0.43261 | 0.46919 | 0.45778 |
| 822.545 | 0.30726 | 0.35657 | 0.43185 | 0.47016 | 0.45623 |
| 823.283 | 0.30504 | 0.35435 | 0.43186 | 0.46905 | 0.45426 |
| 824.02 | 0.30354 | 0.35095 | 0.43239 | 0.46684 | 0.45409 |
| 824.758 | 0.30156 | 0.34901 | 0.4327 | 0.46653 | 0.45378 |
| 825.495 | 0.29924 | 0.34649 | 0.43284 | 0.46487 | 0.4532 |
| 826.232 | 0.29775 | 0.3441 | 0.43265 | 0.46489 | 0.45133 |
| 826.97 | 0.29656 | 0.34084 | 0.43149 | 0.46371 | 0.45133 |
| 827.707 | 0.29425 | 0.34005 | 0.42992 | 0.46412 | 0.44978 |
| 828.444 | 0.29374 | 0.33725 | 0.4296 | 0.46278 | 0.44934 |
| 829.181 | 0.29283 | 0.33568 | 0.42812 | 0.46311 | 0.44874 |
| 829.918 | 0.29119 | 0.33387 | 0.42758 | 0.46069 | 0.45047 |
| 830.655 | 0.2904 | 0.33273 | 0.4279 | 0.4605 | 0.45011 |
| 831.391 | 0.28951 | 0.33019 | 0.42827 | 0.45889 | 0.44931 |
| 832.128 | 0.28791 | 0.32912 | 0.42709 | 0.45839 | 0.44917 |
| 832.865 | 0.28702 | 0.32767 | 0.42807 | 0.45751 | 0.4502 |
| 833.601 | 0.28689 | 0.32634 | 0.42819 | 0.45703 | 0.44959 |
| 834.338 | 0.28606 | 0.32521 | 0.42807 | 0.45608 | 0.44914 |
| 835.074 | 0.28612 | 0.3238 | 0.4273 | 0.45557 | 0.45076 |
| 835.81 | 0.28579 | 0.32316 | 0.42783 | 0.45618 | 0.45097 |
| 836.547 | 0.2855 | 0.32181 | 0.42812 | 0.45548 | 0.45047 |
| 837.283 | 0.28567 | 0.32092 | 0.42886 | 0.45697 | 0.44943 |
| 838.019 | 0.28546 | 0.32038 | 0.42895 | 0.4568 | 0.45091 |
| 838.755 | 0.28438 | 0.3199 | 0.42979 | 0.45704 | 0.44992 |
| 839.491 | 0.28399 | 0.3182 | 0.4304 | 0.45563 | 0.44863 |
| 840.227 | 0.28311 | 0.31755 | 0.42912 | 0.45516 | 0.44885 |
| 840.962 | 0.28216 | 0.31685 | 0.42857 | 0.45502 | 0.44925 |
| 841.698 | 0.28268 | 0.31573 | 0.42949 | 0.45543 | 0.44844 |
| 842.434 | 0.28357 | 0.31597 | 0.43015 | 0.45428 | 0.44891 |
| 843.169 | 0.28483 | 0.31606 | 0.43063 | 0.4546 | 0.45057 |
| 843.905 | 0.28574 | 0.31684 | 0.43219 | 0.45553 | 0.44969 |
| 844.64 | 0.2856 | 0.31703 | 0.4332 | 0.45504 | 0.44905 |
| 845.375 | 0.28551 | 0.31689 | 0.43357 | 0.45298 | 0.44942 |
| 846.111 | 0.28508 | 0.31621 | 0.43383 | 0.45428 | 0.4494 |
| 846.846 | 0.28495 | 0.31681 | 0.43438 | 0.45435 | 0.44869 |
| 847.581 | 0.28518 | 0.31569 | 0.43377 | 0.45447 | 0.44949 |
| 848.316 | 0.28588 | 0.316 | 0.43366 | 0.45404 | 0.45046 |
| 849.051 | 0.28724 | 0.3176 | 0.43396 | 0.45695 | 0.45098 |
| 849.786 | 0.28834 | 0.31823 | 0.43379 | 0.45857 | 0.45127 |
| 850.52 | 0.28802 | 0.31822 | 0.43401 | 0.45956 | 0.45359 |
| 851.255 | 0.28855 | 0.31992 | 0.43513 | 0.46099 | 0.45171 |
| 851.99 | 0.289 | 0.32071 | 0.4354 | 0.46181 | 0.45183 |
| 852.724 | 0.2888 | 0.32136 | 0.4358 | 0.4615 | 0.45169 |
| 853.459 | 0.28848 | 0.32258 | 0.43676 | 0.46123 | 0.45114 |
| 854.193 | 0.28992 | 0.32301 | 0.43773 | 0.4611 | 0.45174 |
| 854.927 | 0.28997 | 0.32332 | 0.43833 | 0.46086 | 0.45467 |
| 855.662 | 0.29185 | 0.32321 | 0.4404 | 0.46094 | 0.45589 |
| 856.396 | 0.29346 | 0.3234 | 0.44129 | 0.4628 | 0.45769 |
| 857.13 | 0.29573 | 0.32429 | 0.44212 | 0.46368 | 0.46026 |
| 857.864 | 0.2972 | 0.32565 | 0.44242 | 0.46481 | 0.45951 |
| 858.598 | 0.29895 | 0.32662 | 0.44361 | 0.4651 | 0.45993 |
| 859.332 | 0.29957 | 0.32768 | 0.44331 | 0.4656 | 0.46006 |
| 860.065 | 0.3003 | 0.32951 | 0.44492 | 0.46682 | 0.45939 |
| 860.799 | 0.30174 | 0.32987 | 0.44583 | 0.46699 | 0.46011 |
| 861.533 | 0.30263 | 0.33107 | 0.44684 | 0.46759 | 0.46162 |
| 862.266 | 0.30408 | 0.33253 | 0.44777 | 0.46839 | 0.4612 |
| 863 | 0.30489 | 0.33475 | 0.44986 | 0.46971 | 0.46211 |
| 863.733 | 0.30595 | 0.33545 | 0.4497 | 0.46949 | 0.46293 |
| 864.466 | 0.3072 | 0.33734 | 0.45133 | 0.47094 | 0.46367 |
| 865.2 | 0.30814 | 0.33913 | 0.45263 | 0.47118 | 0.46331 |
| 865.933 | 0.30885 | 0.34042 | 0.4541 | 0.47122 | 0.46497 |
| 866.666 | 0.31142 | 0.34186 | 0.45408 | 0.47254 | 0.46611 |
| 867.399 | 0.31316 | 0.34415 | 0.45658 | 0.47331 | 0.46813 |
| 868.132 | 0.31464 | 0.34677 | 0.45749 | 0.47404 | 0.46769 |
| 868.865 | 0.31753 | 0.34844 | 0.45982 | 0.47772 | 0.46957 |
| 869.597 | 0.32035 | 0.3512 | 0.4618 | 0.48048 | 0.4713 |
| 870.33 | 0.32175 | 0.353 | 0.46393 | 0.48173 | 0.4729 |
| 871.063 | 0.32416 | 0.3545 | 0.46425 | 0.4832 | 0.47299 |
| 871.795 | 0.32595 | 0.35561 | 0.46557 | 0.48452 | 0.47423 |
| 872.528 | 0.32702 | 0.35773 | 0.46629 | 0.48372 | 0.47435 |
| 873.26 | 0.3289 | 0.35911 | 0.46686 | 0.48379 | 0.47353 |
| 873.992 | 0.33172 | 0.36179 | 0.46787 | 0.48555 | 0.47525 |
| 874.725 | 0.33319 | 0.36472 | 0.46967 | 0.48706 | 0.47625 |
| 875.457 | 0.33519 | 0.3677 | 0.47088 | 0.48896 | 0.47762 |
| 876.189 | 0.33769 | 0.37116 | 0.47269 | 0.49164 | 0.48038 |
| 876.921 | 0.33978 | 0.37408 | 0.47332 | 0.49367 | 0.48312 |
| 877.653 | 0.3417 | 0.37652 | 0.47529 | 0.49389 | 0.48398 |
| 878.385 | 0.34404 | 0.37829 | 0.47649 | 0.49403 | 0.48489 |
| 879.116 | 0.34665 | 0.38104 | 0.47893 | 0.49576 | 0.48617 |
| 879.848 | 0.34886 | 0.38341 | 0.47989 | 0.49663 | 0.48713 |
| 880.58 | 0.35196 | 0.38562 | 0.482 | 0.49826 | 0.48897 |
| 881.311 | 0.35419 | 0.3883 | 0.48288 | 0.50082 | 0.48959 |
| 882.043 | 0.35687 | 0.39193 | 0.48463 | 0.50379 | 0.49051 |
| 882.774 | 0.35865 | 0.39349 | 0.4857 | 0.50387 | 0.49223 |
| 883.505 | 0.36126 | 0.39536 | 0.48759 | 0.5046 | 0.49192 |
| 884.236 | 0.36296 | 0.3975 | 0.48949 | 0.50463 | 0.49196 |
| 884.968 | 0.3649 | 0.40021 | 0.49134 | 0.506 | 0.49348 |
| 885.699 | 0.36743 | 0.40211 | 0.49285 | 0.50664 | 0.49586 |
| 886.43 | 0.3709 | 0.40591 | 0.49401 | 0.50787 | 0.49762 |
| 887.161 | 0.37329 | 0.40897 | 0.49564 | 0.51015 | 0.49955 |
| 887.891 | 0.37607 | 0.4135 | 0.49767 | 0.51309 | 0.50109 |
| 888.622 | 0.37885 | 0.41631 | 0.49987 | 0.51466 | 0.50189 |
| 889.353 | 0.38221 | 0.42 | 0.50115 | 0.51757 | 0.504 |
| 890.083 | 0.38535 | 0.42314 | 0.5033 | 0.51987 | 0.50534 |
| 890.814 | 0.38835 | 0.42627 | 0.5054 | 0.52118 | 0.50803 |
| 891.544 | 0.39124 | 0.42872 | 0.50639 | 0.5233 | 0.51051 |
| 892.275 | 0.39437 | 0.43153 | 0.50785 | 0.52485 | 0.51294 |
| 893.005 | 0.39665 | 0.43326 | 0.51022 | 0.52526 | 0.51494 |
| 893.735 | 0.39918 | 0.43555 | 0.51155 | 0.52653 | 0.51805 |
| 894.465 | 0.40216 | 0.43932 | 0.51276 | 0.52906 | 0.51927 |
| 895.195 | 0.40501 | 0.44225 | 0.51377 | 0.52976 | 0.52013 |
| 895.925 | 0.40764 | 0.44579 | 0.51529 | 0.53133 | 0.52382 |
| 896.655 | 0.41021 | 0.45081 | 0.51657 | 0.53361 | 0.52515 |
| 897.385 | 0.41251 | 0.45479 | 0.51852 | 0.53467 | 0.52642 |
| 898.115 | 0.41529 | 0.45775 | 0.51938 | 0.53543 | 0.52789 |
| 898.844 | 0.41898 | 0.46208 | 0.52235 | 0.53872 | 0.53179 |
| 899.574 | 0.42235 | 0.46583 | 0.52513 | 0.54128 | 0.53124 |
| 900.304 | 0.42533 | 0.46899 | 0.52784 | 0.54354 | 0.53276 |
| 901.033 | 0.42934 | 0.47256 | 0.53004 | 0.54696 | 0.53407 |
| 901.762 | 0.4337 | 0.47576 | 0.5332 | 0.54864 | 0.53657 |
| 902.492 | 0.43659 | 0.47879 | 0.53492 | 0.5499 | 0.53812 |
| 903.221 | 0.43981 | 0.48317 | 0.53686 | 0.55098 | 0.54045 |
| 903.95 | 0.44346 | 0.48672 | 0.53904 | 0.55299 | 0.54209 |
| 904.679 | 0.44726 | 0.49057 | 0.54254 | 0.55512 | 0.54462 |
| 905.408 | 0.45032 | 0.4944 | 0.54481 | 0.55779 | 0.54658 |
| 906.137 | 0.45355 | 0.49801 | 0.54717 | 0.55921 | 0.54915 |
| 906.866 | 0.4563 | 0.50136 | 0.54912 | 0.56246 | 0.55115 |
| 907.594 | 0.45969 | 0.50507 | 0.55079 | 0.56498 | 0.55456 |
| 908.323 | 0.462 | 0.50918 | 0.55133 | 0.56724 | 0.55546 |
| 909.051 | 0.46513 | 0.5136 | 0.553 | 0.56896 | 0.55786 |
| 909.78 | 0.46884 | 0.51709 | 0.55526 | 0.57128 | 0.55818 |
| 910.508 | 0.47347 | 0.52151 | 0.55761 | 0.57239 | 0.56155 |
| 911.237 | 0.47726 | 0.52599 | 0.55992 | 0.57453 | 0.56243 |
| 911.965 | 0.48119 | 0.53021 | 0.56217 | 0.57699 | 0.56603 |
| 912.693 | 0.48497 | 0.53417 | 0.5654 | 0.58054 | 0.56869 |
| 913.421 | 0.48865 | 0.53948 | 0.56744 | 0.5839 | 0.57152 |
| 914.149 | 0.49186 | 0.54198 | 0.57036 | 0.58647 | 0.57321 |
| 914.877 | 0.49534 | 0.54573 | 0.57249 | 0.58838 | 0.57577 |
| 915.605 | 0.49896 | 0.54897 | 0.57493 | 0.58988 | 0.57926 |
| 916.333 | 0.50189 | 0.55261 | 0.57566 | 0.5916 | 0.58061 |
| 917.06 | 0.50528 | 0.55644 | 0.57886 | 0.59394 | 0.58374 |
| 917.788 | 0.50885 | 0.56157 | 0.57948 | 0.59617 | 0.58707 |
| 918.516 | 0.51192 | 0.56557 | 0.58237 | 0.59968 | 0.59006 |
| 919.243 | 0.51594 | 0.56939 | 0.58437 | 0.60266 | 0.59038 |
| 919.97 | 0.51958 | 0.57353 | 0.58709 | 0.60547 | 0.5927 |
| 920.698 | 0.52207 | 0.57776 | 0.58803 | 0.60757 | 0.59445 |
| 921.425 | 0.52565 | 0.58155 | 0.59094 | 0.61049 | 0.59493 |
| 922.152 | 0.53012 | 0.58574 | 0.59347 | 0.61251 | 0.59729 |
| 922.879 | 0.53342 | 0.59097 | 0.59669 | 0.61507 | 0.60135 |
| 923.606 | 0.53731 | 0.5956 | 0.5996 | 0.61732 | 0.60308 |
| 924.333 | 0.54212 | 0.59942 | 0.60207 | 0.62054 | 0.60565 |
| 925.06 | 0.5465 | 0.60333 | 0.60503 | 0.62279 | 0.60847 |
| 925.787 | 0.54962 | 0.60639 | 0.60786 | 0.62458 | 0.61139 |
| 926.513 | 0.55352 | 0.6095 | 0.6101 | 0.62669 | 0.61191 |
| 927.24 | 0.55782 | 0.61326 | 0.61135 | 0.6287 | 0.61465 |
| 927.966 | 0.56139 | 0.61715 | 0.61494 | 0.63077 | 0.61881 |
| 928.693 | 0.56509 | 0.62235 | 0.61677 | 0.63339 | 0.6215 |
| 929.419 | 0.56932 | 0.62705 | 0.61801 | 0.63511 | 0.62254 |
| 930.146 | 0.57298 | 0.63187 | 0.62071 | 0.63879 | 0.62706 |
| 930.872 | 0.57575 | 0.63712 | 0.62422 | 0.64217 | 0.62977 |
| 931.598 | 0.57927 | 0.6413 | 0.62643 | 0.64431 | 0.63054 |
| 932.324 | 0.58248 | 0.64509 | 0.62951 | 0.64804 | 0.6335 |
| 933.05 | 0.58704 | 0.65042 | 0.63245 | 0.6522 | 0.63612 |
| 933.776 | 0.5897 | 0.65493 | 0.63467 | 0.65386 | 0.63565 |
| 934.502 | 0.59351 | 0.65954 | 0.63795 | 0.65672 | 0.63867 |
| 935.227 | 0.59735 | 0.665 | 0.64086 | 0.66027 | 0.64106 |
| 935.953 | 0.60267 | 0.66875 | 0.64245 | 0.66169 | 0.64391 |
| 936.679 | 0.60641 | 0.67299 | 0.64523 | 0.66655 | 0.64746 |
| 937.404 | 0.61067 | 0.67853 | 0.64787 | 0.67126 | 0.65116 |
| 938.129 | 0.61522 | 0.68144 | 0.64961 | 0.67513 | 0.65393 |
| 938.855 | 0.62015 | 0.68604 | 0.65012 | 0.67784 | 0.65621 |
| 939.58 | 0.62286 | 0.69201 | 0.65303 | 0.68086 | 0.65999 |
| 940.305 | 0.62733 | 0.69609 | 0.65587 | 0.68237 | 0.66158 |
| 941.03 | 0.63291 | 0.69872 | 0.65881 | 0.68536 | 0.66469 |
| 941.755 | 0.63689 | 0.70468 | 0.66141 | 0.68617 | 0.66894 |
| 942.48 | 0.64129 | 0.70896 | 0.6649 | 0.69086 | 0.6725 |
| 943.205 | 0.64522 | 0.71362 | 0.66766 | 0.69646 | 0.67187 |
| 943.93 | 0.64819 | 0.71748 | 0.67062 | 0.69775 | 0.67595 |
| 944.655 | 0.65218 | 0.72262 | 0.67252 | 0.69864 | 0.67872 |
| 945.379 | 0.65618 | 0.72774 | 0.67544 | 0.70412 | 0.68183 |
| 946.104 | 0.66003 | 0.73027 | 0.6784 | 0.70609 | 0.6844 |
| 946.828 | 0.66434 | 0.73382 | 0.68068 | 0.70785 | 0.68983 |
| 947.553 | 0.66855 | 0.73795 | 0.68251 | 0.71109 | 0.69357 |
| 948.277 | 0.67284 | 0.7419 | 0.68507 | 0.71432 | 0.69779 |
| 949.001 | 0.67699 | 0.74623 | 0.68821 | 0.71726 | 0.6997 |
| 949.725 | 0.68117 | 0.75141 | 0.69188 | 0.72043 | 0.70244 |
| 950.449 | 0.6841 | 0.75626 | 0.69498 | 0.72371 | 0.7045 |
| 951.173 | 0.68695 | 0.76103 | 0.69772 | 0.72666 | 0.70719 |
| 951.897 | 0.69041 | 0.76487 | 0.70061 | 0.72957 | 0.70966 |
| 952.621 | 0.69486 | 0.76796 | 0.70306 | 0.73185 | 0.71302 |
| 953.345 | 0.69899 | 0.77261 | 0.70502 | 0.7339 | 0.71582 |
| 954.068 | 0.70461 | 0.77746 | 0.70807 | 0.73777 | 0.72016 |
| 954.792 | 0.71077 | 0.78265 | 0.71117 | 0.74186 | 0.72326 |
| 955.515 | 0.7143 | 0.78761 | 0.71536 | 0.74762 | 0.72727 |
| 956.239 | 0.71927 | 0.79352 | 0.71929 | 0.75272 | 0.73013 |
| 956.962 | 0.72391 | 0.79823 | 0.72319 | 0.75687 | 0.73491 |
| 957.686 | 0.72804 | 0.80296 | 0.72665 | 0.7608 | 0.73676 |
| 958.409 | 0.73301 | 0.80706 | 0.72997 | 0.76466 | 0.74074 |
| 959.132 | 0.73904 | 0.81257 | 0.733 | 0.76797 | 0.74362 |
| 959.855 | 0.74341 | 0.81451 | 0.73423 | 0.76878 | 0.74615 |
| 960.578 | 0.74671 | 0.81926 | 0.73754 | 0.77519 | 0.74797 |
| 961.301 | 0.75066 | 0.82206 | 0.73976 | 0.77781 | 0.75316 |
| 962.023 | 0.75287 | 0.82726 | 0.74292 | 0.78174 | 0.75532 |
| 962.746 | 0.7561 | 0.82944 | 0.74394 | 0.78295 | 0.75835 |
| 963.469 | 0.75743 | 0.83557 | 0.7468 | 0.78846 | 0.76148 |
| 964.191 | 0.76084 | 0.83735 | 0.74748 | 0.78773 | 0.76518 |
| 964.914 | 0.76401 | 0.84093 | 0.75016 | 0.79038 | 0.76639 |
| 965.636 | 0.76673 | 0.84207 | 0.7513 | 0.79018 | 0.76857 |
| 966.359 | 0.76877 | 0.8453 | 0.75436 | 0.7927 | 0.77016 |
| 967.081 | 0.7737 | 0.84546 | 0.75696 | 0.79326 | 0.77275 |
| 967.803 | 0.77598 | 0.84892 | 0.75924 | 0.79704 | 0.77598 |
| 968.525 | 0.77819 | 0.85229 | 0.76141 | 0.79902 | 0.77665 |
| 969.247 | 0.78216 | 0.85587 | 0.76442 | 0.80243 | 0.78163 |
| 969.969 | 0.78571 | 0.85983 | 0.76773 | 0.80532 | 0.78502 |
| 970.691 | 0.78764 | 0.86513 | 0.77017 | 0.80892 | 0.78819 |
| 971.412 | 0.79278 | 0.86963 | 0.77343 | 0.81233 | 0.78957 |
| 972.134 | 0.79685 | 0.87394 | 0.77524 | 0.8161 | 0.79598 |
| 972.856 | 0.80156 | 0.87724 | 0.77901 | 0.81884 | 0.79824 |
| 973.577 | 0.80604 | 0.87969 | 0.78051 | 0.82242 | 0.80031 |
| 974.299 | 0.81129 | 0.88247 | 0.78398 | 0.82357 | 0.80406 |
| 975.02 | 0.81401 | 0.88448 | 0.78669 | 0.82545 | 0.8075 |
| 975.741 | 0.81831 | 0.88724 | 0.78978 | 0.82865 | 0.81023 |
| 976.462 | 0.81982 | 0.89132 | 0.79039 | 0.83204 | 0.81288 |
| 977.184 | 0.82306 | 0.89667 | 0.7923 | 0.83552 | 0.8166 |
| 977.905 | 0.82465 | 0.90039 | 0.79348 | 0.84001 | 0.8175 |
| 978.626 | 0.82797 | 0.90314 | 0.79603 | 0.84195 | 0.82105 |
| 979.346 | 0.83125 | 0.90588 | 0.7995 | 0.84291 | 0.82319 |
| 980.067 | 0.83469 | 0.90772 | 0.80153 | 0.84501 | 0.82531 |
| 980.788 | 0.83772 | 0.90974 | 0.80441 | 0.84586 | 0.82769 |
| 981.509 | 0.84174 | 0.91238 | 0.80644 | 0.8492 | 0.83211 |
| 982.229 | 0.8443 | 0.91673 | 0.8088 | 0.85242 | 0.83466 |
| 982.95 | 0.84674 | 0.9207 | 0.80978 | 0.8562 | 0.83723 |
| 983.67 | 0.84878 | 0.92569 | 0.81232 | 0.86034 | 0.83734 |
| 984.39 | 0.8516 | 0.92958 | 0.8146 | 0.86396 | 0.84028 |
| 985.111 | 0.85372 | 0.93268 | 0.81782 | 0.86592 | 0.84243 |
| 985.831 | 0.8573 | 0.93623 | 0.82033 | 0.86861 | 0.8462 |
| 986.551 | 0.86048 | 0.93987 | 0.82263 | 0.87107 | 0.8474 |
| 987.271 | 0.86488 | 0.94212 | 0.82587 | 0.87236 | 0.85344 |
| 987.991 | 0.86768 | 0.94433 | 0.8282 | 0.87455 | 0.85662 |
| 988.71 | 0.87207 | 0.94785 | 0.83052 | 0.87646 | 0.86112 |
| 989.43 | 0.87464 | 0.94977 | 0.8323 | 0.87956 | 0.86288 |
| 990.15 | 0.87739 | 0.95267 | 0.83606 | 0.88428 | 0.86684 |
| 990.869 | 0.88022 | 0.95628 | 0.83729 | 0.88902 | 0.86903 |
| 991.589 | 0.88325 | 0.95973 | 0.83983 | 0.89355 | 0.87153 |
| 992.308 | 0.88571 | 0.96175 | 0.84187 | 0.8964 | 0.87289 |
| 993.028 | 0.88899 | 0.96474 | 0.84467 | 0.89957 | 0.8753 |
| 993.747 | 0.89155 | 0.96535 | 0.84607 | 0.89975 | 0.87891 |
| 994.466 | 0.89511 | 0.96876 | 0.85066 | 0.90439 | 0.88052 |
| 995.185 | 0.89762 | 0.97124 | 0.85369 | 0.90745 | 0.8831 |
| 995.904 | 0.90006 | 0.97276 | 0.85764 | 0.91081 | 0.88796 |
| 996.623 | 0.90288 | 0.97626 | 0.85978 | 0.91285 | 0.88971 |
| 997.342 | 0.90455 | 0.97829 | 0.86187 | 0.91551 | 0.89011 |
| 998.061 | 0.90492 | 0.97793 | 0.86218 | 0.91384 | 0.89355 |
| 998.78 | 0.90654 | 0.97826 | 0.86394 | 0.91266 | 0.89585 |
| 999.498 | 0.90777 | 0.98606 | 0.86159 | 0.91419 | 0.88833 |
| 1000.217 | 0.45315 | 0.9481 | 0.75797 | 0.76426 | 0.4934 |
| 1000.935 | 0.52536 | 0.8575 | 0.68267 | 0.75984 | 0.66493 |
| 1001.654 | 0.53096 | 0.7095 | 0.67391 | 0.62533 | 0.54972 |
| 1002.372 | 0.55155 | 0.69499 | 0.64362 | 0.59008 | 0.59893 |
| 1003.09 | 0.53068 | 0.69734 | 0.63991 | 0.61404 | 0.57517 |
| 1003.808 | 0.52905 | 0.70847 | 0.61151 | 0.60682 | 0.58876 |
| 1004.526 | 0.89412 | 0.972 | 0.85031 | 0.90072 | 0.88812 |
| 1005.244 | 0.90775 | 0.9821 | 0.85525 | 0.91617 | 0.89361 |
| 1005.962 | 0.91331 | 0.98496 | 0.86179 | 0.91939 | 0.89782 |
| 1006.68 | 0.91592 | 0.98796 | 0.86493 | 0.92045 | 0.90017 |
| 1007.398 | 0.91752 | 0.99056 | 0.8663 | 0.92189 | 0.89983 |
| 1008.115 | 0.92097 | 0.99427 | 0.86905 | 0.92549 | 0.90112 |
| 1008.833 | 0.92168 | 0.99553 | 0.87175 | 0.92453 | 0.90284 |
| 1009.55 | 0.92196 | 0.99792 | 0.87149 | 0.92502 | 0.90246 |
| 1010.268 | 0.92387 | 0.99952 | 0.87116 | 0.92628 | 0.90198 |
| 1010.985 | 0.92868 | 1 | 0.87533 | 0.92741 | 0.90902 |
| 1011.702 | 1 | 0.88492 | 0.87821 | 0.49282 | 0.78612 |
| 1012.419 | 0.83875 | 0.79088 | 1 | 0.64943 | 0.87633 |

Date of figure 4(b)

| categories | wavelength corresponding to the lowest absorption value in the blue–green region | *h*° | w(Cr_2_O_3_+Fe_2_O_3_) |
| --- | --- | --- | --- |
| 480-490 | 480.103 | 170.85 | 0.025 |
|  | 483.184 | 174.99 | 0.021 |
|  | 483.954 | 169.12 | 0.018 |
|  | 483.954 | 169.93 | 0.02 |
|  | 483.954 | 164.54 | 0.025 |
|  | 485.493 | 169.01 | 0.023 |
|  | 485.493 | 168.90 | 0.024 |
|  | 486.263 | 164.00 | 0.026 |
|  | 489.341 | 170.58 | 0.025 |
| Mean value | 484.638 | 169.10 | 0.023 |
| 490-500 | 490.88 | 166.13 | 0.021 |
|  | 492.419 | 165.27 | 0.018 |
|  | 493.957 | 163.46 | 0.025 |
|  | 493.957 | 165.43 | 0.031 |
|  | 494.726 | 166.69 | 0.022 |
|  | 495.495 | 168.66 | 0.015 |
|  | 495.495 | 162.46 | 0.023 |
|  | 496.264 | 165.59 | 0.027 |
|  | 496.264 | 161.46 | 0.029 |
|  | 497.801 | 165.76 | 0.028 |
|  | 498.57 | 158.33 | 0.024 |
|  | 499.339 | 162.62 | 0.026 |
| Mean value | 495.431 | 164.32 | 0.024 |
| 500-510 | 500.876 | 167.02 | 0.01 |
|  | 500.876 | 168.25 | 0.022 |
|  | 500.876 | 161.83 | 0.026 |
|  | 502.412 | 162.21 | 0.024 |
|  | 503.181 | 161.74 | 0.03 |
|  | 503.949 | 162.40 | 0.023 |
|  | 503.949 | 157.47 | 0.026 |
|  | 503.949 | 159.70 | 0.029 |
|  | 504.717 | 163.07 | 0.03 |
|  | 505.485 | 157.22 | 0.037 |
|  | 507.021 | 156.09 | 0.055 |
|  | 507.789 | 164.78 | 0.037 |
|  | 509.324 | 159.19 | 0.033 |
|  | 509.324 | 159.60 | 0.034 |
| Mean value | 504.552 | 161.47 | 0.030 |
| 510-520 | 510.092 | 159.98 | 0.04 |
|  | 510.859 | 162.62 | 0.031 |
|  | 513.929 | 150.86 | 0.042 |
| Mean value | 511.627 | 157.82 | 0.038 |
| 520-530 | 520.832 | 151.76 | 0.046 |
|  | 521.598 | 153.37 | 0.034 |
| Mean value | 521.215 | 152.57 | 0.040 |
| 530-540 | 536.151 | 147.29 | 0.037 |
| Mean value | 536.151 | 147.29 | 0.037 |

Date of figure 4(c)

| *C** | 22.59 | 29.76 | 45.91 | 52.82 |
| --- | --- | --- | --- | --- |
| wavelength | absorbance | | | |
| 223.933 | 0.96251 | 0.89017 | 0.90335 | 0.89585 |
| 224.724 | 0.92321 | 0.85625 | 0.88898 | 0.90497 |
| 225.516 | 0.93687 | 0.88587 | 0.9012 | 0.9039 |
| 226.307 | 0.92922 | 0.87914 | 0.90372 | 0.90338 |
| 227.098 | 0.930 | 0.89 | 0.908 | 0.91058 |
| 227.889 | 0.930 | 0.89 | 0.915 | 0.91192 |
| 228.68 | 0.92813 | 0.8922 | 0.92054 | 0.91954 |
| 229.471 | 0.92603 | 0.89326 | 0.92588 | 0.92051 |
| 230.262 | 0.92895 | 0.90097 | 0.92423 | 0.92437 |
| 231.053 | 0.92552 | 0.90247 | 0.92672 | 0.92026 |
| 231.844 | 0.92731 | 0.90949 | 0.92339 | 0.9246 |
| 232.635 | 0.92646 | 0.91301 | 0.91896 | 0.9288 |
| 233.425 | 0.93103 | 0.91299 | 0.92331 | 0.93182 |
| 234.216 | 0.92827 | 0.91115 | 0.9271 | 0.93676 |
| 235.007 | 0.93066 | 0.90811 | 0.92925 | 0.93834 |
| 235.797 | 0.92859 | 0.90356 | 0.92935 | 0.93854 |
| 236.588 | 0.92638 | 0.8963 | 0.93232 | 0.93582 |
| 237.378 | 0.917 | 0.89 | 0.934 | 0.93592 |
| 238.168 | 0.91105 | 0.89116 | 0.93604 | 0.93474 |
| 238.959 | 0.90699 | 0.88819 | 0.93713 | 0.93787 |
| 239.749 | 0.90107 | 0.8814 | 0.94284 | 0.93866 |
| 240.539 | 0.89352 | 0.87717 | 0.94337 | 0.9377 |
| 241.329 | 0.88875 | 0.86997 | 0.93879 | 0.93573 |
| 242.119 | 0.8822 | 0.86176 | 0.93809 | 0.93016 |
| 242.909 | 0.87317 | 0.85251 | 0.93593 | 0.92645 |
| 243.699 | 0.86455 | 0.84637 | 0.92904 | 0.92624 |
| 244.489 | 0.85742 | 0.83473 | 0.92714 | 0.92388 |
| 245.279 | 0.84858 | 0.82356 | 0.92393 | 0.92175 |
| 246.068 | 0.83884 | 0.81429 | 0.91973 | 0.92206 |
| 246.858 | 0.82915 | 0.8057 | 0.91521 | 0.92127 |
| 247.648 | 0.8196 | 0.79346 | 0.91484 | 0.91871 |
| 248.437 | 0.80852 | 0.78738 | 0.91183 | 0.91695 |
| 249.227 | 0.79898 | 0.77951 | 0.90644 | 0.91282 |
| 250.016 | 0.79034 | 0.76967 | 0.90407 | 0.90969 |
| 250.805 | 0.78131 | 0.76158 | 0.9048 | 0.90494 |
| 251.595 | 0.77487 | 0.75424 | 0.90301 | 0.89652 |
| 252.384 | 0.76679 | 0.74559 | 0.89952 | 0.89367 |
| 253.173 | 0.76092 | 0.73605 | 0.90214 | 0.89091 |
| 253.962 | 0.7567 | 0.72963 | 0.90047 | 0.88771 |
| 254.751 | 0.75006 | 0.72022 | 0.89652 | 0.88572 |
| 255.54 | 0.74379 | 0.71441 | 0.89492 | 0.88588 |
| 256.329 | 0.74187 | 0.70881 | 0.89582 | 0.88278 |
| 257.118 | 0.7379 | 0.70464 | 0.89313 | 0.88138 |
| 257.907 | 0.73349 | 0.69866 | 0.8909 | 0.8784 |
| 258.695 | 0.73082 | 0.69471 | 0.88775 | 0.87453 |
| 259.484 | 0.7282 | 0.68886 | 0.88628 | 0.87143 |
| 260.273 | 0.72392 | 0.68364 | 0.88464 | 0.86941 |
| 261.061 | 0.72017 | 0.68096 | 0.88346 | 0.86519 |
| 261.85 | 0.71801 | 0.67847 | 0.88378 | 0.86357 |
| 262.638 | 0.71637 | 0.67541 | 0.88499 | 0.86076 |
| 263.426 | 0.71434 | 0.6732 | 0.88392 | 0.85862 |
| 264.215 | 0.7123 | 0.67 | 0.883 | 0.8573 |
| 265.003 | 0.71108 | 0.66593 | 0.88424 | 0.85325 |
| 265.791 | 0.71004 | 0.66187 | 0.88234 | 0.85071 |
| 266.579 | 0.7081 | 0.65949 | 0.88191 | 0.84929 |
| 267.367 | 0.70804 | 0.65601 | 0.8835 | 0.84615 |
| 268.155 | 0.70761 | 0.65165 | 0.883 | 0.84129 |
| 268.943 | 0.707 | 0.64779 | 0.88043 | 0.84061 |
| 269.731 | 0.70682 | 0.64536 | 0.882 | 0.83819 |
| 270.519 | 0.70745 | 0.64341 | 0.88234 | 0.83764 |
| 271.306 | 0.70696 | 0.64249 | 0.88086 | 0.83678 |
| 272.094 | 0.70714 | 0.64235 | 0.87884 | 0.83505 |
| 272.882 | 0.70891 | 0.64148 | 0.88085 | 0.83473 |
| 273.669 | 0.70687 | 0.63907 | 0.88199 | 0.83521 |
| 274.457 | 0.70632 | 0.63589 | 0.88246 | 0.83488 |
| 275.244 | 0.7054 | 0.63415 | 0.88373 | 0.83399 |
| 276.031 | 0.70362 | 0.63282 | 0.88614 | 0.83346 |
| 276.819 | 0.70062 | 0.63091 | 0.8874 | 0.8293 |
| 277.606 | 0.70138 | 0.63008 | 0.88566 | 0.82588 |
| 278.393 | 0.7015 | 0.63058 | 0.8844 | 0.82109 |
| 279.18 | 0.70231 | 0.62798 | 0.88299 | 0.81964 |
| 279.967 | 0.70512 | 0.62516 | 0.88523 | 0.81902 |
| 280.754 | 0.70653 | 0.62524 | 0.88274 | 0.82052 |
| 281.541 | 0.70797 | 0.62335 | 0.88325 | 0.82044 |
| 282.328 | 0.70832 | 0.62056 | 0.8846 | 0.82125 |
| 283.115 | 0.70709 | 0.61878 | 0.88523 | 0.81908 |
| 283.901 | 0.70546 | 0.61621 | 0.88215 | 0.81495 |
| 284.688 | 0.70485 | 0.61245 | 0.88264 | 0.81367 |
| 285.475 | 0.70383 | 0.60989 | 0.8823 | 0.81124 |
| 286.261 | 0.70393 | 0.60771 | 0.87987 | 0.80917 |
| 287.048 | 0.703 | 0.60383 | 0.8794 | 0.80721 |
| 287.834 | 0.70297 | 0.60151 | 0.87951 | 0.80701 |
| 288.62 | 0.70338 | 0.59892 | 0.87705 | 0.80356 |
| 289.407 | 0.70209 | 0.59614 | 0.87757 | 0.79765 |
| 290.193 | 0.70065 | 0.59266 | 0.87762 | 0.79373 |
| 290.979 | 0.70066 | 0.58974 | 0.87789 | 0.78959 |
| 291.765 | 0.69906 | 0.58761 | 0.87651 | 0.78678 |
| 292.551 | 0.69577 | 0.58534 | 0.87746 | 0.78334 |
| 293.337 | 0.69455 | 0.58402 | 0.87548 | 0.78313 |
| 294.123 | 0.69353 | 0.58073 | 0.87685 | 0.78038 |
| 294.909 | 0.69204 | 0.57935 | 0.87408 | 0.77907 |
| 295.695 | 0.69038 | 0.57679 | 0.87435 | 0.77546 |
| 296.48 | 0.69248 | 0.57433 | 0.87267 | 0.77381 |
| 297.266 | 0.69243 | 0.57238 | 0.87098 | 0.77251 |
| 298.052 | 0.69211 | 0.57211 | 0.86979 | 0.77088 |
| 298.837 | 0.69125 | 0.57076 | 0.87063 | 0.76936 |
| 299.623 | 0.693 | 0.56976 | 0.87262 | 0.7691 |
| 300.408 | 0.69123 | 0.56815 | 0.87487 | 0.76725 |
| 301.193 | 0.68954 | 0.56598 | 0.87593 | 0.7652 |
| 301.979 | 0.68883 | 0.56404 | 0.87657 | 0.76344 |
| 302.764 | 0.68892 | 0.56284 | 0.87687 | 0.76067 |
| 303.549 | 0.68841 | 0.5617 | 0.87794 | 0.75795 |
| 304.334 | 0.68808 | 0.56104 | 0.87891 | 0.7556 |
| 305.119 | 0.68964 | 0.5597 | 0.88294 | 0.75419 |
| 305.904 | 0.69023 | 0.55948 | 0.88166 | 0.7536 |
| 306.689 | 0.69195 | 0.55886 | 0.88311 | 0.75246 |
| 307.474 | 0.69296 | 0.55739 | 0.88175 | 0.75338 |
| 308.259 | 0.69351 | 0.55608 | 0.88222 | 0.75373 |
| 309.043 | 0.69392 | 0.55522 | 0.87996 | 0.7519 |
| 309.828 | 0.69449 | 0.55329 | 0.88127 | 0.7489 |
| 310.612 | 0.69457 | 0.55141 | 0.88012 | 0.74773 |
| 311.397 | 0.69288 | 0.55016 | 0.87868 | 0.74554 |
| 312.181 | 0.69378 | 0.54922 | 0.8782 | 0.74335 |
| 312.966 | 0.69377 | 0.54821 | 0.87766 | 0.74104 |
| 313.75 | 0.69386 | 0.5473 | 0.88015 | 0.74069 |
| 314.534 | 0.69342 | 0.54692 | 0.88261 | 0.73912 |
| 315.319 | 0.69427 | 0.54625 | 0.88699 | 0.73643 |
| 316.103 | 0.69423 | 0.54615 | 0.88598 | 0.73444 |
| 316.887 | 0.69331 | 0.54544 | 0.88745 | 0.7331 |
| 317.671 | 0.69248 | 0.54432 | 0.88752 | 0.7321 |
| 318.455 | 0.69372 | 0.54319 | 0.88735 | 0.73117 |
| 319.239 | 0.69421 | 0.54211 | 0.88494 | 0.73122 |
| 320.022 | 0.6951 | 0.54129 | 0.88682 | 0.73075 |
| 320.806 | 0.69647 | 0.54016 | 0.88623 | 0.73133 |
| 321.59 | 0.69727 | 0.54056 | 0.88497 | 0.73069 |
| 322.373 | 0.69626 | 0.54 | 0.88744 | 0.73013 |
| 323.157 | 0.69602 | 0.54013 | 0.88835 | 0.72792 |
| 323.941 | 0.69566 | 0.53894 | 0.88813 | 0.72661 |
| 324.724 | 0.69643 | 0.53909 | 0.8903 | 0.72516 |
| 325.507 | 0.69693 | 0.53904 | 0.89267 | 0.72268 |
| 326.291 | 0.69694 | 0.53899 | 0.88999 | 0.72067 |
| 327.074 | 0.69706 | 0.53867 | 0.89036 | 0.72063 |
| 327.857 | 0.6969 | 0.53815 | 0.8927 | 0.71929 |
| 328.64 | 0.69555 | 0.5373 | 0.89371 | 0.71782 |
| 329.423 | 0.69645 | 0.53508 | 0.89256 | 0.71888 |
| 330.206 | 0.69676 | 0.53402 | 0.89375 | 0.71784 |
| 330.989 | 0.69807 | 0.53327 | 0.89503 | 0.71498 |
| 331.772 | 0.70006 | 0.53294 | 0.89334 | 0.71463 |
| 332.555 | 0.70148 | 0.53328 | 0.89233 | 0.71366 |
| 333.338 | 0.70117 | 0.53436 | 0.89323 | 0.7114 |
| 334.12 | 0.70147 | 0.53483 | 0.89328 | 0.71112 |
| 334.903 | 0.70028 | 0.53449 | 0.89243 | 0.71177 |
| 335.685 | 0.69897 | 0.53426 | 0.8935 | 0.71086 |
| 336.468 | 0.69741 | 0.53376 | 0.89338 | 0.70957 |
| 337.25 | 0.69741 | 0.53224 | 0.89524 | 0.70861 |
| 338.033 | 0.6974 | 0.53182 | 0.89481 | 0.70671 |
| 338.815 | 0.69901 | 0.53105 | 0.89662 | 0.7054 |
| 339.597 | 0.7001 | 0.53039 | 0.89646 | 0.70433 |
| 340.379 | 0.70169 | 0.52999 | 0.899 | 0.70394 |
| 341.161 | 0.70158 | 0.53052 | 0.89673 | 0.7032 |
| 341.943 | 0.7025 | 0.53106 | 0.89742 | 0.70378 |
| 342.725 | 0.70145 | 0.53134 | 0.89569 | 0.7037 |
| 343.507 | 0.70142 | 0.53232 | 0.89529 | 0.70385 |
| 344.289 | 0.70313 | 0.53273 | 0.89351 | 0.70561 |
| 345.071 | 0.70499 | 0.5329 | 0.89392 | 0.70717 |
| 345.852 | 0.70598 | 0.53259 | 0.8944 | 0.70905 |
| 346.634 | 0.70731 | 0.53354 | 0.8956 | 0.70993 |
| 347.416 | 0.70867 | 0.5342 | 0.89689 | 0.71252 |
| 348.197 | 0.70948 | 0.5346 | 0.89817 | 0.71296 |
| 348.979 | 0.71065 | 0.53516 | 0.89789 | 0.71439 |
| 349.76 | 0.71199 | 0.53662 | 0.89804 | 0.71685 |
| 350.541 | 0.71472 | 0.53827 | 0.89925 | 0.71995 |
| 351.323 | 0.71733 | 0.54031 | 0.89991 | 0.72333 |
| 352.104 | 0.71948 | 0.54323 | 0.90189 | 0.72784 |
| 352.885 | 0.72168 | 0.5461 | 0.90553 | 0.73295 |
| 353.666 | 0.72526 | 0.5488 | 0.90723 | 0.73641 |
| 354.447 | 0.72864 | 0.55185 | 0.90839 | 0.74268 |
| 355.228 | 0.73092 | 0.55481 | 0.91101 | 0.74895 |
| 356.009 | 0.7333 | 0.55817 | 0.91225 | 0.75669 |
| 356.79 | 0.73662 | 0.56281 | 0.91609 | 0.76419 |
| 357.57 | 0.73873 | 0.56649 | 0.92072 | 0.77216 |
| 358.351 | 0.7424 | 0.56982 | 0.92585 | 0.77943 |
| 359.132 | 0.74629 | 0.57336 | 0.92765 | 0.78593 |
| 359.912 | 0.74952 | 0.57776 | 0.93095 | 0.79406 |
| 360.693 | 0.75568 | 0.58174 | 0.9329 | 0.80121 |
| 361.473 | 0.76087 | 0.58685 | 0.93584 | 0.81099 |
| 362.253 | 0.76546 | 0.59344 | 0.93859 | 0.81799 |
| 363.034 | 0.76932 | 0.60009 | 0.94451 | 0.82641 |
| 363.814 | 0.77736 | 0.60584 | 0.94917 | 0.83407 |
| 364.594 | 0.78143 | 0.61253 | 0.95545 | 0.84187 |
| 365.374 | 0.78694 | 0.61888 | 0.95998 | 0.84767 |
| 366.154 | 0.79129 | 0.62453 | 0.96138 | 0.85834 |
| 366.934 | 0.79668 | 0.63066 | 0.96358 | 0.86747 |
| 367.714 | 0.79912 | 0.63713 | 0.96895 | 0.87378 |
| 368.494 | 0.80247 | 0.64307 | 0.97124 | 0.88188 |
| 369.274 | 0.80633 | 0.64853 | 0.9721 | 0.89177 |
| 370.053 | 0.81064 | 0.65443 | 0.97762 | 0.89798 |
| 370.833 | 0.81538 | 0.65998 | 0.98485 | 0.90548 |
| 371.612 | 0.81998 | 0.66481 | 0.98512 | 0.91109 |
| 372.392 | 0.82578 | 0.67011 | 0.98906 | 0.91736 |
| 373.171 | 0.83209 | 0.67593 | 0.99487 | 0.92186 |
| 373.951 | 0.83843 | 0.68034 | 1.00138 | 0.92841 |
| 374.73 | 0.84384 | 0.68434 | 1.00283 | 0.93077 |
| 375.509 | 0.84893 | 0.6878 | 1.00657 | 0.93621 |
| 376.289 | 0.85244 | 0.69236 | 1.00716 | 0.94015 |
| 377.068 | 0.85557 | 0.69649 | 1.01181 | 0.94429 |
| 377.847 | 0.85884 | 0.70148 | 1.01244 | 0.94858 |
| 378.626 | 0.86227 | 0.70644 | 1.01217 | 0.95273 |
| 379.405 | 0.8661 | 0.71108 | 1.01177 | 0.95535 |
| 380.183 | 0.8677 | 0.71372 | 1.01373 | 0.95996 |
| 380.962 | 0.86759 | 0.71647 | 1.01871 | 0.96324 |
| 381.741 | 0.86715 | 0.71864 | 1.0185 | 0.96411 |
| 382.52 | 0.86952 | 0.72082 | 1.02114 | 0.96443 |
| 383.298 | 0.86773 | 0.72174 | 1.02353 | 0.9673 |
| 384.077 | 0.87023 | 0.72207 | 1.02424 | 0.96776 |
| 384.855 | 0.87114 | 0.72201 | 1.02057 | 0.96676 |
| 385.634 | 0.87124 | 0.72229 | 1.02103 | 0.96773 |
| 386.412 | 0.86864 | 0.72192 | 1.02206 | 0.96893 |
| 387.19 | 0.86994 | 0.72222 | 1.02295 | 0.96815 |
| 387.968 | 0.86912 | 0.72283 | 1.0225 | 0.96755 |
| 388.747 | 0.8697 | 0.72248 | 1.02129 | 0.96812 |
| 389.525 | 0.86981 | 0.72179 | 1.02163 | 0.96773 |
| 390.303 | 0.87122 | 0.72078 | 1.0208 | 0.96828 |
| 391.081 | 0.87007 | 0.71844 | 1.01812 | 0.96767 |
| 391.858 | 0.87027 | 0.71613 | 1.01793 | 0.96668 |
| 392.636 | 0.87095 | 0.71546 | 1.01834 | 0.96451 |
| 393.414 | 0.86971 | 0.71279 | 1.01972 | 0.96306 |
| 394.192 | 0.86731 | 0.71008 | 1.01982 | 0.96145 |
| 394.969 | 0.86731 | 0.70943 | 1.02109 | 0.95906 |
| 395.747 | 0.86461 | 0.70741 | 1.02038 | 0.95551 |
| 396.524 | 0.86239 | 0.70521 | 1.01951 | 0.95338 |
| 397.302 | 0.86031 | 0.70269 | 1.01499 | 0.94924 |
| 398.079 | 0.85876 | 0.70136 | 1.0106 | 0.94601 |
| 398.856 | 0.85775 | 0.6982 | 1.00985 | 0.94361 |
| 399.634 | 0.85788 | 0.696 | 1.00871 | 0.94262 |
| 400.411 | 0.85702 | 0.69203 | 1.00646 | 0.94127 |
| 401.188 | 0.85613 | 0.68959 | 1.00474 | 0.93934 |
| 401.965 | 0.85619 | 0.68611 | 1.00427 | 0.93716 |
| 402.742 | 0.85257 | 0.68327 | 1.00001 | 0.93485 |
| 403.519 | 0.8505 | 0.68011 | 0.99775 | 0.93214 |
| 404.296 | 0.84712 | 0.67797 | 0.99544 | 0.92951 |
| 405.072 | 0.8459 | 0.67545 | 0.99408 | 0.9269 |
| 405.849 | 0.84302 | 0.67195 | 0.9924 | 0.92282 |
| 406.626 | 0.84274 | 0.66878 | 0.99129 | 0.91856 |
| 407.402 | 0.84003 | 0.66528 | 0.98802 | 0.91494 |
| 408.179 | 0.83794 | 0.66137 | 0.98555 | 0.90967 |
| 408.955 | 0.83535 | 0.65795 | 0.98451 | 0.9046 |
| 409.732 | 0.83245 | 0.65429 | 0.98119 | 0.90057 |
| 410.508 | 0.8291 | 0.65067 | 0.97852 | 0.89693 |
| 411.284 | 0.82721 | 0.64619 | 0.97668 | 0.89132 |
| 412.06 | 0.82605 | 0.64219 | 0.9738 | 0.88736 |
| 412.836 | 0.82339 | 0.63712 | 0.96849 | 0.88164 |
| 413.612 | 0.82117 | 0.6331 | 0.96661 | 0.87537 |
| 414.388 | 0.81905 | 0.62863 | 0.96443 | 0.86947 |
| 415.164 | 0.81596 | 0.62417 | 0.96088 | 0.86455 |
| 415.94 | 0.81156 | 0.62017 | 0.95675 | 0.85839 |
| 416.716 | 0.80877 | 0.61568 | 0.95297 | 0.85252 |
| 417.492 | 0.80519 | 0.61059 | 0.94872 | 0.84604 |
| 418.267 | 0.80114 | 0.6054 | 0.94316 | 0.83879 |
| 419.043 | 0.79723 | 0.60078 | 0.93798 | 0.83044 |
| 419.818 | 0.79483 | 0.59552 | 0.93269 | 0.82197 |
| 420.594 | 0.79076 | 0.59029 | 0.92735 | 0.81421 |
| 421.369 | 0.78808 | 0.58546 | 0.92199 | 0.80577 |
| 422.144 | 0.78521 | 0.58022 | 0.91661 | 0.79747 |
| 422.92 | 0.78232 | 0.57508 | 0.91057 | 0.7889 |
| 423.695 | 0.77828 | 0.56931 | 0.90552 | 0.78033 |
| 424.47 | 0.77573 | 0.56421 | 0.90087 | 0.77201 |
| 425.245 | 0.77204 | 0.55894 | 0.89471 | 0.76424 |
| 426.02 | 0.7691 | 0.55382 | 0.88882 | 0.75569 |
| 426.795 | 0.76604 | 0.5493 | 0.88347 | 0.74774 |
| 427.57 | 0.76331 | 0.54502 | 0.87824 | 0.73992 |
| 428.345 | 0.76044 | 0.54135 | 0.87317 | 0.73117 |
| 429.119 | 0.75802 | 0.53724 | 0.8683 | 0.72218 |
| 429.894 | 0.75487 | 0.5336 | 0.86349 | 0.71483 |
| 430.669 | 0.75217 | 0.53005 | 0.85828 | 0.70726 |
| 431.443 | 0.75041 | 0.52679 | 0.8532 | 0.69989 |
| 432.217 | 0.74763 | 0.52305 | 0.8483 | 0.69321 |
| 432.992 | 0.74541 | 0.52052 | 0.8432 | 0.68737 |
| 433.766 | 0.74342 | 0.51821 | 0.83827 | 0.68075 |
| 434.54 | 0.74161 | 0.51565 | 0.83473 | 0.67469 |
| 435.315 | 0.73953 | 0.51323 | 0.83132 | 0.66885 |
| 436.089 | 0.73879 | 0.51115 | 0.82734 | 0.66345 |
| 436.863 | 0.73744 | 0.50859 | 0.82418 | 0.65759 |
| 437.637 | 0.73597 | 0.50594 | 0.82117 | 0.65186 |
| 438.411 | 0.73473 | 0.50391 | 0.81817 | 0.64759 |
| 439.185 | 0.73374 | 0.5024 | 0.81487 | 0.64342 |
| 439.958 | 0.73224 | 0.50056 | 0.81194 | 0.6396 |
| 440.732 | 0.73144 | 0.49917 | 0.80949 | 0.63614 |
| 441.506 | 0.73053 | 0.49783 | 0.8069 | 0.63368 |
| 442.279 | 0.7297 | 0.4966 | 0.80434 | 0.62968 |
| 443.053 | 0.72909 | 0.49476 | 0.80225 | 0.62615 |
| 443.826 | 0.72802 | 0.49314 | 0.7995 | 0.62265 |
| 444.6 | 0.72719 | 0.49173 | 0.79648 | 0.62004 |
| 445.373 | 0.72729 | 0.49061 | 0.79413 | 0.61633 |
| 446.146 | 0.72672 | 0.4891 | 0.79105 | 0.61371 |
| 446.919 | 0.72591 | 0.48772 | 0.78869 | 0.6106 |
| 447.693 | 0.72602 | 0.48668 | 0.78682 | 0.60817 |
| 448.466 | 0.72566 | 0.48525 | 0.7848 | 0.60485 |
| 449.239 | 0.72527 | 0.48388 | 0.78171 | 0.6024 |
| 450.012 | 0.72492 | 0.48228 | 0.78008 | 0.59934 |
| 450.784 | 0.72421 | 0.48146 | 0.77781 | 0.59661 |
| 451.557 | 0.72353 | 0.48012 | 0.77468 | 0.5934 |
| 452.33 | 0.72262 | 0.47879 | 0.7721 | 0.5904 |
| 453.103 | 0.72174 | 0.47716 | 0.77048 | 0.58704 |
| 453.875 | 0.72185 | 0.47571 | 0.76763 | 0.58387 |
| 454.648 | 0.72172 | 0.47397 | 0.76513 | 0.58044 |
| 455.42 | 0.72047 | 0.47258 | 0.76287 | 0.57625 |
| 456.192 | 0.72054 | 0.47118 | 0.76039 | 0.5726 |
| 456.965 | 0.72032 | 0.46983 | 0.75749 | 0.56867 |
| 457.737 | 0.71899 | 0.46857 | 0.75489 | 0.56481 |
| 458.509 | 0.71775 | 0.46729 | 0.75178 | 0.56104 |
| 459.281 | 0.71795 | 0.46579 | 0.749 | 0.55751 |
| 460.053 | 0.7168 | 0.4644 | 0.74629 | 0.55349 |
| 460.825 | 0.71618 | 0.46289 | 0.74334 | 0.54934 |
| 461.597 | 0.71554 | 0.46149 | 0.74044 | 0.54526 |
| 462.369 | 0.71564 | 0.45973 | 0.73706 | 0.54047 |
| 463.141 | 0.71485 | 0.45852 | 0.73377 | 0.53617 |
| 463.913 | 0.71477 | 0.45719 | 0.73 | 0.53167 |
| 464.684 | 0.7138 | 0.45588 | 0.72665 | 0.52723 |
| 465.456 | 0.7139 | 0.45405 | 0.72314 | 0.52246 |
| 466.227 | 0.71332 | 0.45269 | 0.71969 | 0.5185 |
| 466.999 | 0.71304 | 0.45082 | 0.71691 | 0.51396 |
| 467.77 | 0.71256 | 0.4492 | 0.71412 | 0.50952 |
| 468.541 | 0.71193 | 0.44773 | 0.71154 | 0.50547 |
| 469.313 | 0.71105 | 0.44637 | 0.70846 | 0.50115 |
| 470.084 | 0.71051 | 0.44488 | 0.70562 | 0.49662 |
| 470.855 | 0.7098 | 0.44341 | 0.70234 | 0.4922 |
| 471.626 | 0.70951 | 0.44197 | 0.69931 | 0.48773 |
| 472.397 | 0.7095 | 0.44056 | 0.69574 | 0.4829 |
| 473.168 | 0.70912 | 0.43956 | 0.69278 | 0.47861 |
| 473.939 | 0.7085 | 0.43851 | 0.68964 | 0.47425 |
| 474.71 | 0.70849 | 0.43735 | 0.6865 | 0.46999 |
| 475.48 | 0.70802 | 0.43628 | 0.68319 | 0.46626 |
| 476.251 | 0.70752 | 0.43536 | 0.68035 | 0.46251 |
| 477.021 | 0.70749 | 0.43427 | 0.67716 | 0.45862 |
| 477.792 | 0.70765 | 0.43329 | 0.67442 | 0.45472 |
| 478.562 | 0.70736 | 0.43231 | 0.67186 | 0.45102 |
| 479.333 | 0.70728 | 0.43125 | 0.66901 | 0.44697 |
| 480.103 | 0.70778 | 0.43015 | 0.6667 | 0.4431 |
| 480.873 | 0.70755 | 0.42933 | 0.66447 | 0.4397 |
| 481.643 | 0.70736 | 0.42859 | 0.6623 | 0.43686 |
| 482.414 | 0.70748 | 0.42802 | 0.65956 | 0.434 |
| 483.184 | 0.70706 | 0.42736 | 0.65724 | 0.43137 |
| 483.954 | 0.70682 | 0.42688 | 0.65503 | 0.42896 |
| 484.723 | 0.70718 | 0.42614 | 0.65243 | 0.42655 |
| 485.493 | 0.70753 | 0.42554 | 0.6502 | 0.42423 |
| 486.263 | 0.70804 | 0.42482 | 0.64857 | 0.42197 |
| 487.033 | 0.70839 | 0.42431 | 0.64684 | 0.42005 |
| 487.802 | 0.70876 | 0.42387 | 0.64457 | 0.41823 |
| 488.572 | 0.70898 | 0.42355 | 0.64279 | 0.41652 |
| 489.341 | 0.7094 | 0.4231 | 0.64118 | 0.41485 |
| 490.111 | 0.70906 | 0.42326 | 0.63945 | 0.41334 |
| 490.88 | 0.70996 | 0.42307 | 0.63808 | 0.41219 |
| 491.649 | 0.70982 | 0.4226 | 0.63715 | 0.41106 |
| 492.419 | 0.71031 | 0.42248 | 0.63597 | 0.41022 |
| 493.188 | 0.71062 | 0.4223 | 0.63463 | 0.40945 |
| 493.957 | 0.71171 | 0.42192 | 0.63404 | 0.4088 |
| 494.726 | 0.7117 | 0.42181 | 0.63308 | 0.40793 |
| 495.495 | 0.71283 | 0.42198 | 0.63228 | 0.40749 |
| 496.264 | 0.71309 | 0.42189 | 0.63195 | 0.40701 |
| 497.032 | 0.71365 | 0.42183 | 0.63177 | 0.40665 |
| 497.801 | 0.7143 | 0.4222 | 0.63083 | 0.40645 |
| 498.57 | 0.71484 | 0.42246 | 0.63034 | 0.40632 |
| 499.339 | 0.71502 | 0.42271 | 0.62964 | 0.40597 |
| 500.107 | 0.71544 | 0.42281 | 0.62881 | 0.40581 |
| 500.876 | 0.71583 | 0.42323 | 0.6282 | 0.40557 |
| 501.644 | 0.71611 | 0.4233 | 0.62773 | 0.40567 |
| 502.412 | 0.71657 | 0.42373 | 0.62732 | 0.40569 |
| 503.181 | 0.71721 | 0.42391 | 0.62704 | 0.40575 |
| 503.949 | 0.7178 | 0.4243 | 0.62693 | 0.40581 |
| 504.717 | 0.71854 | 0.42441 | 0.62675 | 0.40606 |
| 505.485 | 0.71933 | 0.42461 | 0.62664 | 0.40639 |
| 506.253 | 0.72029 | 0.42451 | 0.62643 | 0.4067 |
| 507.021 | 0.72107 | 0.42484 | 0.62635 | 0.40724 |
| 507.789 | 0.72237 | 0.42517 | 0.62623 | 0.40785 |
| 508.556 | 0.72289 | 0.4258 | 0.62641 | 0.40834 |
| 509.324 | 0.72339 | 0.42624 | 0.62635 | 0.40867 |
| 510.092 | 0.72358 | 0.42667 | 0.62633 | 0.40923 |
| 510.859 | 0.72399 | 0.42705 | 0.62619 | 0.40997 |
| 511.627 | 0.72432 | 0.42774 | 0.62659 | 0.41043 |
| 512.394 | 0.72497 | 0.42776 | 0.62644 | 0.41119 |
| 513.162 | 0.72541 | 0.42803 | 0.6268 | 0.41195 |
| 513.929 | 0.72631 | 0.42876 | 0.62725 | 0.41281 |
| 514.696 | 0.72705 | 0.42918 | 0.62802 | 0.41346 |
| 515.463 | 0.72739 | 0.42959 | 0.62835 | 0.4141 |
| 516.231 | 0.72795 | 0.43021 | 0.62884 | 0.41467 |
| 516.998 | 0.72858 | 0.43107 | 0.62952 | 0.4154 |
| 517.765 | 0.72931 | 0.43143 | 0.63012 | 0.41624 |
| 518.531 | 0.72996 | 0.43173 | 0.63067 | 0.41715 |
| 519.298 | 0.73041 | 0.43226 | 0.63101 | 0.41798 |
| 520.065 | 0.73134 | 0.43279 | 0.63157 | 0.41897 |
| 520.832 | 0.73172 | 0.43299 | 0.63181 | 0.41989 |
| 521.598 | 0.73201 | 0.43349 | 0.6323 | 0.42079 |
| 522.365 | 0.73259 | 0.43416 | 0.63292 | 0.42174 |
| 523.131 | 0.73348 | 0.43446 | 0.63398 | 0.42304 |
| 523.898 | 0.73396 | 0.43517 | 0.63469 | 0.42429 |
| 524.664 | 0.73506 | 0.43599 | 0.63565 | 0.42547 |
| 525.43 | 0.73568 | 0.43637 | 0.63653 | 0.42646 |
| 526.197 | 0.73654 | 0.43696 | 0.6371 | 0.42768 |
| 526.963 | 0.7372 | 0.43761 | 0.63723 | 0.42874 |
| 527.729 | 0.73768 | 0.43803 | 0.63814 | 0.42977 |
| 528.495 | 0.73797 | 0.43839 | 0.63896 | 0.43091 |
| 529.261 | 0.73911 | 0.43914 | 0.63994 | 0.43228 |
| 530.027 | 0.73953 | 0.43969 | 0.64059 | 0.43339 |
| 530.792 | 0.74013 | 0.44025 | 0.64213 | 0.43445 |
| 531.558 | 0.7413 | 0.44084 | 0.6432 | 0.43551 |
| 532.324 | 0.74234 | 0.44118 | 0.64386 | 0.43646 |
| 533.089 | 0.74285 | 0.44171 | 0.64466 | 0.43744 |
| 533.855 | 0.74366 | 0.44244 | 0.64638 | 0.43869 |
| 534.62 | 0.74442 | 0.44311 | 0.64726 | 0.44007 |
| 535.386 | 0.74514 | 0.44365 | 0.64818 | 0.44161 |
| 536.151 | 0.74544 | 0.44458 | 0.64959 | 0.44333 |
| 536.916 | 0.74657 | 0.44533 | 0.65082 | 0.44477 |
| 537.682 | 0.74736 | 0.4458 | 0.65174 | 0.44643 |
| 538.447 | 0.74815 | 0.44631 | 0.653 | 0.44804 |
| 539.212 | 0.74878 | 0.44714 | 0.65432 | 0.44947 |
| 539.977 | 0.74964 | 0.44783 | 0.65572 | 0.4512 |
| 540.742 | 0.75011 | 0.4487 | 0.65703 | 0.45314 |
| 541.506 | 0.75092 | 0.44967 | 0.65858 | 0.45483 |
| 542.271 | 0.75137 | 0.45071 | 0.6601 | 0.45676 |
| 543.036 | 0.75202 | 0.45172 | 0.66149 | 0.45876 |
| 543.801 | 0.75291 | 0.45246 | 0.66319 | 0.46088 |
| 544.565 | 0.75335 | 0.45336 | 0.66502 | 0.46301 |
| 545.33 | 0.75404 | 0.45413 | 0.66659 | 0.46518 |
| 546.094 | 0.75507 | 0.455 | 0.6684 | 0.46726 |
| 546.858 | 0.75588 | 0.45595 | 0.67027 | 0.46982 |
| 547.623 | 0.7564 | 0.45717 | 0.67205 | 0.47222 |
| 548.387 | 0.75745 | 0.45799 | 0.67427 | 0.4747 |
| 549.151 | 0.75789 | 0.45905 | 0.67635 | 0.47726 |
| 549.915 | 0.75898 | 0.46005 | 0.67827 | 0.48024 |
| 550.679 | 0.76012 | 0.4608 | 0.68062 | 0.48287 |
| 551.443 | 0.76158 | 0.46169 | 0.68257 | 0.48543 |
| 552.207 | 0.76285 | 0.46285 | 0.68418 | 0.4884 |
| 552.971 | 0.76417 | 0.46391 | 0.68603 | 0.49136 |
| 553.734 | 0.76509 | 0.46493 | 0.6886 | 0.49424 |
| 554.498 | 0.76556 | 0.46608 | 0.69074 | 0.49729 |
| 555.262 | 0.76639 | 0.4673 | 0.69342 | 0.50049 |
| 556.025 | 0.767 | 0.46854 | 0.69607 | 0.5036 |
| 556.789 | 0.76773 | 0.46988 | 0.69884 | 0.50701 |
| 557.552 | 0.76867 | 0.47114 | 0.70136 | 0.51054 |
| 558.315 | 0.76985 | 0.47255 | 0.70411 | 0.51418 |
| 559.078 | 0.77067 | 0.47395 | 0.70674 | 0.51811 |
| 559.842 | 0.77173 | 0.475 | 0.70977 | 0.52191 |
| 560.605 | 0.77311 | 0.47643 | 0.71241 | 0.52584 |
| 561.368 | 0.77397 | 0.47797 | 0.71542 | 0.52983 |
| 562.131 | 0.77503 | 0.47943 | 0.71842 | 0.53387 |
| 562.894 | 0.77628 | 0.48092 | 0.72146 | 0.53792 |
| 563.656 | 0.77736 | 0.48251 | 0.72434 | 0.54228 |
| 564.419 | 0.7784 | 0.48405 | 0.72769 | 0.5465 |
| 565.182 | 0.77964 | 0.48544 | 0.73083 | 0.55092 |
| 565.945 | 0.78082 | 0.48694 | 0.73419 | 0.5555 |
| 566.707 | 0.78224 | 0.48845 | 0.73759 | 0.56002 |
| 567.47 | 0.78364 | 0.49034 | 0.74103 | 0.56468 |
| 568.232 | 0.78476 | 0.49206 | 0.74475 | 0.56942 |
| 568.994 | 0.78608 | 0.49383 | 0.74806 | 0.57449 |
| 569.757 | 0.78718 | 0.49561 | 0.75153 | 0.57969 |
| 570.519 | 0.78823 | 0.49759 | 0.75526 | 0.58481 |
| 571.281 | 0.78927 | 0.4994 | 0.75898 | 0.59019 |
| 572.043 | 0.79065 | 0.50115 | 0.7624 | 0.59555 |
| 572.805 | 0.79184 | 0.50309 | 0.76641 | 0.60096 |
| 573.567 | 0.79298 | 0.50513 | 0.77026 | 0.60614 |
| 574.329 | 0.79453 | 0.50728 | 0.77383 | 0.61223 |
| 575.09 | 0.79597 | 0.50931 | 0.77816 | 0.61792 |
| 575.852 | 0.79763 | 0.51162 | 0.78219 | 0.62392 |
| 576.614 | 0.7993 | 0.51383 | 0.78627 | 0.62973 |
| 577.375 | 0.80112 | 0.51612 | 0.7903 | 0.63582 |
| 578.137 | 0.80222 | 0.51839 | 0.79454 | 0.64141 |
| 578.898 | 0.804 | 0.52064 | 0.79817 | 0.64721 |
| 579.66 | 0.8054 | 0.52291 | 0.80267 | 0.65301 |
| 580.421 | 0.80683 | 0.52541 | 0.8072 | 0.6589 |
| 581.182 | 0.80847 | 0.52782 | 0.81192 | 0.66516 |
| 581.943 | 0.80998 | 0.53015 | 0.81664 | 0.67178 |
| 582.704 | 0.81128 | 0.53256 | 0.82151 | 0.67813 |
| 583.465 | 0.81284 | 0.535 | 0.82616 | 0.68484 |
| 584.226 | 0.81463 | 0.53746 | 0.8307 | 0.69111 |
| 584.987 | 0.81623 | 0.53979 | 0.83518 | 0.69731 |
| 585.748 | 0.8183 | 0.54211 | 0.83958 | 0.70321 |
| 586.509 | 0.81995 | 0.54474 | 0.84416 | 0.70973 |
| 587.269 | 0.82186 | 0.54746 | 0.84844 | 0.71587 |
| 588.03 | 0.82346 | 0.55018 | 0.85283 | 0.72247 |
| 588.79 | 0.82535 | 0.553 | 0.85725 | 0.72907 |
| 589.551 | 0.82722 | 0.55588 | 0.8618 | 0.73547 |
| 590.311 | 0.8291 | 0.55878 | 0.86637 | 0.7417 |
| 591.072 | 0.831 | 0.56153 | 0.87174 | 0.74817 |
| 591.832 | 0.83283 | 0.5644 | 0.87654 | 0.75416 |
| 592.592 | 0.83473 | 0.56737 | 0.88075 | 0.76023 |
| 593.352 | 0.83641 | 0.57008 | 0.88522 | 0.76641 |
| 594.112 | 0.83832 | 0.57279 | 0.88974 | 0.77238 |
| 594.872 | 0.84002 | 0.57567 | 0.89374 | 0.77839 |
| 595.632 | 0.84204 | 0.57851 | 0.89814 | 0.78475 |
| 596.392 | 0.84346 | 0.58141 | 0.90309 | 0.79097 |
| 597.151 | 0.84548 | 0.58459 | 0.90724 | 0.7968 |
| 597.911 | 0.84718 | 0.58771 | 0.91115 | 0.80295 |
| 598.671 | 0.84904 | 0.59064 | 0.91503 | 0.80873 |
| 599.43 | 0.85104 | 0.59363 | 0.91889 | 0.81442 |
| 600.19 | 0.85347 | 0.59673 | 0.92298 | 0.82032 |
| 600.949 | 0.85522 | 0.59962 | 0.92753 | 0.82619 |
| 601.708 | 0.85754 | 0.60272 | 0.93191 | 0.83175 |
| 602.468 | 0.85938 | 0.6059 | 0.93595 | 0.83733 |
| 603.227 | 0.86129 | 0.60887 | 0.93997 | 0.84285 |
| 603.986 | 0.86322 | 0.61162 | 0.94381 | 0.84792 |
| 604.745 | 0.86536 | 0.61466 | 0.94779 | 0.85277 |
| 605.504 | 0.86737 | 0.6175 | 0.95113 | 0.85786 |
| 606.263 | 0.86974 | 0.62031 | 0.95512 | 0.86257 |
| 607.022 | 0.87166 | 0.62328 | 0.95879 | 0.86778 |
| 607.78 | 0.87362 | 0.62648 | 0.9624 | 0.8728 |
| 608.539 | 0.87571 | 0.62948 | 0.96594 | 0.8783 |
| 609.298 | 0.87767 | 0.63231 | 0.97019 | 0.88321 |
| 610.056 | 0.87913 | 0.63549 | 0.97353 | 0.88844 |
| 610.815 | 0.88129 | 0.63874 | 0.97666 | 0.89296 |
| 611.573 | 0.8831 | 0.64183 | 0.9799 | 0.89759 |
| 612.332 | 0.88489 | 0.64506 | 0.98332 | 0.90203 |
| 613.09 | 0.88674 | 0.6484 | 0.98638 | 0.90714 |
| 613.848 | 0.88907 | 0.65166 | 0.98968 | 0.91214 |
| 614.606 | 0.8908 | 0.65475 | 0.99302 | 0.91696 |
| 615.364 | 0.89275 | 0.6575 | 0.99643 | 0.92136 |
| 616.122 | 0.89481 | 0.66035 | 0.99984 | 0.92611 |
| 616.88 | 0.89719 | 0.66318 | 1.00296 | 0.93019 |
| 617.638 | 0.89895 | 0.66592 | 1.00569 | 0.93358 |
| 618.396 | 0.90087 | 0.66842 | 1.00887 | 0.93755 |
| 619.153 | 0.90314 | 0.67127 | 1.0114 | 0.94159 |
| 619.911 | 0.9045 | 0.67383 | 1.0138 | 0.94482 |
| 620.668 | 0.90583 | 0.67659 | 1.01591 | 0.94838 |
| 621.426 | 0.9078 | 0.67905 | 1.01905 | 0.95177 |
| 622.183 | 0.90952 | 0.68166 | 1.0219 | 0.9551 |
| 622.941 | 0.91112 | 0.68425 | 1.0247 | 0.95816 |
| 623.698 | 0.91318 | 0.68654 | 1.02721 | 0.96104 |
| 624.455 | 0.91455 | 0.68872 | 1.03004 | 0.96412 |
| 625.212 | 0.91594 | 0.6911 | 1.03199 | 0.96723 |
| 625.969 | 0.91761 | 0.6934 | 1.03356 | 0.96997 |
| 626.726 | 0.91867 | 0.69541 | 1.0358 | 0.97293 |
| 627.483 | 0.92015 | 0.69772 | 1.03735 | 0.97586 |
| 628.24 | 0.92204 | 0.69981 | 1.03926 | 0.97801 |
| 628.997 | 0.92339 | 0.70199 | 1.04118 | 0.98079 |
| 629.753 | 0.92497 | 0.70405 | 1.04312 | 0.98347 |
| 630.51 | 0.9269 | 0.70635 | 1.04447 | 0.98517 |
| 631.267 | 0.92845 | 0.70859 | 1.04627 | 0.98766 |
| 632.023 | 0.92991 | 0.71095 | 1.04803 | 0.99077 |
| 632.78 | 0.93165 | 0.71305 | 1.04937 | 0.99349 |
| 633.536 | 0.93264 | 0.71491 | 1.05175 | 0.99552 |
| 634.292 | 0.93344 | 0.71677 | 1.05375 | 0.99841 |
| 635.048 | 0.93466 | 0.71889 | 1.05564 | 1.00084 |
| 635.804 | 0.93589 | 0.72078 | 1.05731 | 1.00225 |
| 636.561 | 0.93728 | 0.72242 | 1.0598 | 1.00372 |
| 637.316 | 0.93857 | 0.72459 | 1.06058 | 1.006 |
| 638.072 | 0.94016 | 0.72649 | 1.0616 | 1.00778 |
| 638.828 | 0.9411 | 0.72809 | 1.06305 | 1.00939 |
| 639.584 | 0.94233 | 0.72974 | 1.06406 | 1.01092 |
| 640.34 | 0.94313 | 0.73121 | 1.06415 | 1.01269 |
| 641.095 | 0.94445 | 0.73245 | 1.06533 | 1.01385 |
| 641.851 | 0.94499 | 0.73341 | 1.06695 | 1.01516 |
| 642.606 | 0.94621 | 0.73445 | 1.06781 | 1.01635 |
| 643.362 | 0.94686 | 0.73548 | 1.06852 | 1.01759 |
| 644.117 | 0.94761 | 0.73673 | 1.07003 | 1.01846 |
| 644.872 | 0.94771 | 0.73776 | 1.07122 | 1.01929 |
| 645.628 | 0.94842 | 0.73883 | 1.07133 | 1.02031 |
| 646.383 | 0.9491 | 0.7392 | 1.0723 | 1.02081 |
| 647.138 | 0.94938 | 0.73978 | 1.07302 | 1.02149 |
| 647.893 | 0.94973 | 0.73989 | 1.07326 | 1.0221 |
| 648.648 | 0.95073 | 0.73976 | 1.0735 | 1.02226 |
| 649.402 | 0.95095 | 0.74005 | 1.07378 | 1.02219 |
| 650.157 | 0.9512 | 0.74049 | 1.07382 | 1.02284 |
| 650.912 | 0.95173 | 0.74027 | 1.07444 | 1.02368 |
| 651.667 | 0.95181 | 0.74067 | 1.0744 | 1.02349 |
| 652.421 | 0.95193 | 0.74109 | 1.07452 | 1.02376 |
| 653.176 | 0.9523 | 0.74123 | 1.07462 | 1.02427 |
| 653.93 | 0.95246 | 0.74145 | 1.07409 | 1.02467 |
| 654.684 | 0.95267 | 0.74184 | 1.07415 | 1.02414 |
| 655.439 | 0.95296 | 0.74211 | 1.07442 | 1.02472 |
| 656.193 | 0.95291 | 0.74221 | 1.07442 | 1.02484 |
| 656.947 | 0.95289 | 0.74238 | 1.07549 | 1.02494 |
| 657.701 | 0.95264 | 0.74243 | 1.07642 | 1.02466 |
| 658.455 | 0.95258 | 0.74254 | 1.07611 | 1.02494 |
| 659.209 | 0.95263 | 0.74281 | 1.07644 | 1.02523 |
| 659.963 | 0.95279 | 0.74327 | 1.07676 | 1.02594 |
| 660.716 | 0.95323 | 0.74354 | 1.07607 | 1.02575 |
| 661.47 | 0.95378 | 0.74389 | 1.07597 | 1.02585 |
| 662.224 | 0.95445 | 0.74412 | 1.07629 | 1.0261 |
| 662.977 | 0.95464 | 0.7437 | 1.07612 | 1.02556 |
| 663.731 | 0.95527 | 0.74354 | 1.0761 | 1.02548 |
| 664.484 | 0.95517 | 0.74327 | 1.07687 | 1.02524 |
| 665.237 | 0.95492 | 0.74292 | 1.07673 | 1.02543 |
| 665.991 | 0.95475 | 0.74267 | 1.07666 | 1.02513 |
| 666.744 | 0.95464 | 0.74266 | 1.07641 | 1.02536 |
| 667.497 | 0.95384 | 0.74238 | 1.07683 | 1.02492 |
| 668.25 | 0.95357 | 0.74191 | 1.07638 | 1.02522 |
| 669.003 | 0.95346 | 0.74161 | 1.07562 | 1.02474 |
| 669.756 | 0.95341 | 0.74116 | 1.07512 | 1.02433 |
| 670.509 | 0.95333 | 0.74023 | 1.07486 | 1.02365 |
| 671.261 | 0.95344 | 0.7401 | 1.07429 | 1.02309 |
| 672.014 | 0.95346 | 0.73995 | 1.07355 | 1.0227 |
| 672.767 | 0.9539 | 0.73954 | 1.07371 | 1.02228 |
| 673.519 | 0.95351 | 0.73908 | 1.07396 | 1.02165 |
| 674.272 | 0.95319 | 0.73893 | 1.07375 | 1.02109 |
| 675.024 | 0.95309 | 0.73826 | 1.07325 | 1.02087 |
| 675.776 | 0.95252 | 0.73745 | 1.07312 | 1.02035 |
| 676.529 | 0.95152 | 0.73684 | 1.07269 | 1.01947 |
| 677.281 | 0.95119 | 0.73632 | 1.07194 | 1.01919 |
| 678.033 | 0.95106 | 0.7358 | 1.07158 | 1.01873 |
| 678.785 | 0.95084 | 0.73501 | 1.07108 | 1.01807 |
| 679.537 | 0.95121 | 0.73464 | 1.07072 | 1.0172 |
| 680.289 | 0.95156 | 0.73411 | 1.07069 | 1.01709 |
| 681.041 | 0.95145 | 0.73362 | 1.07057 | 1.0169 |
| 681.792 | 0.951 | 0.73335 | 1.07034 | 1.01684 |
| 682.544 | 0.95052 | 0.73312 | 1.0701 | 1.01588 |
| 683.295 | 0.94999 | 0.73267 | 1.06967 | 1.0154 |
| 684.047 | 0.94918 | 0.73215 | 1.06894 | 1.01459 |
| 684.798 | 0.94847 | 0.73154 | 1.06843 | 1.01368 |
| 685.55 | 0.94813 | 0.73102 | 1.06776 | 1.0129 |
| 686.301 | 0.94776 | 0.73045 | 1.06752 | 1.01252 |
| 687.052 | 0.94773 | 0.72989 | 1.0672 | 1.01148 |
| 687.803 | 0.94777 | 0.72983 | 1.06707 | 1.01094 |
| 688.555 | 0.94769 | 0.72936 | 1.06644 | 1.01014 |
| 689.306 | 0.94745 | 0.72871 | 1.06654 | 1.00915 |
| 690.057 | 0.94761 | 0.72801 | 1.06588 | 1.00842 |
| 690.807 | 0.94732 | 0.72741 | 1.06529 | 1.00879 |
| 691.558 | 0.94715 | 0.72643 | 1.06515 | 1.00811 |
| 692.309 | 0.94713 | 0.72596 | 1.06494 | 1.00739 |
| 693.06 | 0.94724 | 0.72545 | 1.06444 | 1.00691 |
| 693.81 | 0.94659 | 0.72497 | 1.06424 | 1.00671 |
| 694.561 | 0.94656 | 0.7245 | 1.06364 | 1.00534 |
| 695.311 | 0.94619 | 0.72407 | 1.06297 | 1.00505 |
| 696.061 | 0.94577 | 0.7237 | 1.06311 | 1.00454 |
| 696.812 | 0.94558 | 0.7233 | 1.06279 | 1.00423 |
| 697.562 | 0.94523 | 0.72279 | 1.06182 | 1.00366 |
| 698.312 | 0.94496 | 0.72239 | 1.06215 | 1.00321 |
| 699.062 | 0.94449 | 0.72198 | 1.06197 | 1.00245 |
| 699.812 | 0.94381 | 0.7212 | 1.06148 | 1.00213 |
| 700.562 | 0.9438 | 0.72041 | 1.06051 | 1.00144 |
| 701.312 | 0.94369 | 0.71983 | 1.06035 | 1.00073 |
| 702.062 | 0.94314 | 0.71936 | 1.05991 | 1.00035 |
| 702.811 | 0.94303 | 0.71882 | 1.05869 | 0.99963 |
| 703.561 | 0.94342 | 0.71852 | 1.05793 | 0.99866 |
| 704.31 | 0.94286 | 0.71826 | 1.05784 | 0.99785 |
| 705.06 | 0.94272 | 0.71792 | 1.05749 | 0.99723 |
| 705.809 | 0.9423 | 0.71727 | 1.05659 | 0.99665 |
| 706.559 | 0.94171 | 0.71674 | 1.05676 | 0.99622 |
| 707.308 | 0.94125 | 0.71603 | 1.05633 | 0.99589 |
| 708.057 | 0.9408 | 0.71547 | 1.05538 | 0.99527 |
| 708.806 | 0.94038 | 0.71492 | 1.05532 | 0.99475 |
| 709.555 | 0.94059 | 0.7142 | 1.05462 | 0.994 |
| 710.304 | 0.94058 | 0.71357 | 1.05375 | 0.99308 |
| 711.053 | 0.94048 | 0.71318 | 1.05353 | 0.99222 |
| 711.802 | 0.9406 | 0.71234 | 1.05367 | 0.99165 |
| 712.551 | 0.94069 | 0.71169 | 1.05282 | 0.99114 |
| 713.299 | 0.94042 | 0.71157 | 1.05281 | 0.99059 |
| 714.048 | 0.94024 | 0.71113 | 1.05295 | 0.99001 |
| 714.796 | 0.94 | 0.71065 | 1.05236 | 0.98975 |
| 715.545 | 0.93981 | 0.71067 | 1.05154 | 0.98921 |
| 716.293 | 0.93941 | 0.71031 | 1.05125 | 0.98836 |
| 717.041 | 0.93926 | 0.70967 | 1.05053 | 0.98776 |
| 717.79 | 0.93885 | 0.70962 | 1.05018 | 0.98778 |
| 718.538 | 0.93879 | 0.70961 | 1.04944 | 0.98722 |
| 719.286 | 0.93844 | 0.7091 | 1.04988 | 0.98691 |
| 720.034 | 0.93841 | 0.70886 | 1.04963 | 0.98661 |
| 720.782 | 0.93773 | 0.7087 | 1.05005 | 0.98654 |
| 721.53 | 0.93803 | 0.70834 | 1.04933 | 0.98578 |
| 722.278 | 0.93802 | 0.70779 | 1.0494 | 0.98584 |
| 723.025 | 0.9378 | 0.70738 | 1.04843 | 0.98533 |
| 723.773 | 0.9378 | 0.70716 | 1.04799 | 0.98486 |
| 724.52 | 0.93799 | 0.70663 | 1.04692 | 0.98411 |
| 725.268 | 0.93778 | 0.7061 | 1.04669 | 0.98353 |
| 726.015 | 0.93767 | 0.70555 | 1.04638 | 0.98244 |
| 726.763 | 0.93748 | 0.70531 | 1.0467 | 0.9818 |
| 727.51 | 0.93742 | 0.70472 | 1.04595 | 0.98073 |
| 728.257 | 0.93732 | 0.70465 | 1.04569 | 0.98009 |
| 729.004 | 0.93729 | 0.70414 | 1.04514 | 0.97949 |
| 729.751 | 0.9371 | 0.70388 | 1.04441 | 0.97887 |
| 730.498 | 0.93729 | 0.70347 | 1.04335 | 0.97841 |
| 731.245 | 0.93713 | 0.70294 | 1.0433 | 0.97813 |
| 731.992 | 0.93656 | 0.70216 | 1.0426 | 0.97699 |
| 732.739 | 0.93611 | 0.7016 | 1.0419 | 0.97599 |
| 733.485 | 0.93517 | 0.70075 | 1.04138 | 0.97462 |
| 734.232 | 0.9344 | 0.69964 | 1.04066 | 0.97314 |
| 734.979 | 0.93396 | 0.69892 | 1.03979 | 0.97154 |
| 735.725 | 0.93383 | 0.6979 | 1.03911 | 0.97036 |
| 736.471 | 0.93391 | 0.69717 | 1.03829 | 0.96868 |
| 737.218 | 0.93381 | 0.69654 | 1.03756 | 0.96744 |
| 737.964 | 0.93355 | 0.69564 | 1.03633 | 0.96603 |
| 738.71 | 0.9326 | 0.69471 | 1.03544 | 0.96459 |
| 739.456 | 0.93169 | 0.69405 | 1.03429 | 0.96282 |
| 740.202 | 0.93055 | 0.6927 | 1.03329 | 0.9612 |
| 740.948 | 0.92966 | 0.69147 | 1.03196 | 0.9597 |
| 741.694 | 0.92901 | 0.6905 | 1.03119 | 0.95824 |
| 742.44 | 0.92884 | 0.68932 | 1.02941 | 0.95632 |
| 743.186 | 0.92857 | 0.68807 | 1.02821 | 0.95465 |
| 743.931 | 0.92776 | 0.68696 | 1.02664 | 0.95287 |
| 744.677 | 0.9272 | 0.68539 | 1.02527 | 0.95077 |
| 745.422 | 0.92678 | 0.68403 | 1.02368 | 0.94861 |
| 746.168 | 0.92592 | 0.68267 | 1.02269 | 0.9469 |
| 746.913 | 0.92504 | 0.68109 | 1.02121 | 0.94512 |
| 747.658 | 0.92457 | 0.67948 | 1.02016 | 0.94324 |
| 748.404 | 0.92452 | 0.67801 | 1.01819 | 0.94113 |
| 749.149 | 0.92396 | 0.67629 | 1.0162 | 0.93834 |
| 749.894 | 0.9231 | 0.67482 | 1.01406 | 0.93555 |
| 750.639 | 0.92283 | 0.67341 | 1.01239 | 0.93227 |
| 751.384 | 0.92193 | 0.67213 | 1.01019 | 0.92907 |
| 752.128 | 0.92068 | 0.67073 | 1.00876 | 0.92573 |
| 752.873 | 0.91963 | 0.66939 | 1.00713 | 0.92329 |
| 753.618 | 0.91891 | 0.6677 | 1.00557 | 0.92051 |
| 754.362 | 0.91771 | 0.66605 | 1.00356 | 0.91817 |
| 755.107 | 0.9171 | 0.66443 | 1.00115 | 0.9158 |
| 755.851 | 0.91615 | 0.6629 | 0.99903 | 0.91293 |
| 756.596 | 0.91525 | 0.66132 | 0.99694 | 0.91003 |
| 757.34 | 0.91436 | 0.65951 | 0.99463 | 0.90681 |
| 758.084 | 0.91372 | 0.65789 | 0.99215 | 0.90372 |
| 758.829 | 0.91287 | 0.65621 | 0.99025 | 0.90027 |
| 759.573 | 0.9123 | 0.65457 | 0.98756 | 0.897 |
| 760.317 | 0.91126 | 0.65274 | 0.98511 | 0.89352 |
| 761.061 | 0.91043 | 0.65139 | 0.98216 | 0.89066 |
| 761.804 | 0.90919 | 0.64961 | 0.97949 | 0.88705 |
| 762.548 | 0.90842 | 0.64779 | 0.97736 | 0.88367 |
| 763.292 | 0.90758 | 0.64613 | 0.97504 | 0.88082 |
| 764.036 | 0.90669 | 0.64449 | 0.97279 | 0.87756 |
| 764.779 | 0.9058 | 0.64266 | 0.97104 | 0.87367 |
| 765.523 | 0.90491 | 0.64109 | 0.96843 | 0.87001 |
| 766.266 | 0.90404 | 0.63963 | 0.96569 | 0.86663 |
| 767.009 | 0.90299 | 0.63771 | 0.9634 | 0.86268 |
| 767.753 | 0.90247 | 0.63638 | 0.9608 | 0.85902 |
| 768.496 | 0.90202 | 0.63489 | 0.95802 | 0.85569 |
| 769.239 | 0.90165 | 0.63329 | 0.95624 | 0.85234 |
| 769.982 | 0.90073 | 0.63161 | 0.95378 | 0.84843 |
| 770.725 | 0.8999 | 0.63047 | 0.95082 | 0.84453 |
| 771.468 | 0.89909 | 0.62868 | 0.94784 | 0.84028 |
| 772.211 | 0.89796 | 0.6272 | 0.94511 | 0.83632 |
| 772.953 | 0.89708 | 0.62582 | 0.94168 | 0.83217 |
| 773.696 | 0.89632 | 0.62448 | 0.93865 | 0.82826 |
| 774.439 | 0.89582 | 0.62282 | 0.93599 | 0.82429 |
| 775.181 | 0.89489 | 0.62139 | 0.9336 | 0.82044 |
| 775.924 | 0.89419 | 0.61996 | 0.93072 | 0.81683 |
| 776.666 | 0.89378 | 0.61855 | 0.92873 | 0.81333 |
| 777.408 | 0.89334 | 0.61718 | 0.92597 | 0.80989 |
| 778.15 | 0.89297 | 0.61584 | 0.9237 | 0.80641 |
| 778.893 | 0.8922 | 0.61461 | 0.92125 | 0.80285 |
| 779.635 | 0.8919 | 0.61339 | 0.91879 | 0.79866 |
| 780.377 | 0.89122 | 0.61202 | 0.91605 | 0.79459 |
| 781.118 | 0.89041 | 0.61076 | 0.91378 | 0.79038 |
| 781.86 | 0.88961 | 0.60959 | 0.91044 | 0.78671 |
| 782.602 | 0.88954 | 0.60828 | 0.90783 | 0.78302 |
| 783.344 | 0.8892 | 0.60679 | 0.90542 | 0.7796 |
| 784.085 | 0.88857 | 0.60574 | 0.90274 | 0.77602 |
| 784.827 | 0.88842 | 0.60448 | 0.90015 | 0.77261 |
| 785.568 | 0.88797 | 0.60346 | 0.89849 | 0.76893 |
| 786.31 | 0.88747 | 0.60227 | 0.89554 | 0.76524 |
| 787.051 | 0.88693 | 0.60142 | 0.89313 | 0.76151 |
| 787.792 | 0.88656 | 0.60044 | 0.89076 | 0.758 |
| 788.533 | 0.88587 | 0.59959 | 0.88882 | 0.75427 |
| 789.275 | 0.88553 | 0.59857 | 0.88645 | 0.75075 |
| 790.016 | 0.88491 | 0.59782 | 0.88449 | 0.74733 |
| 790.757 | 0.88408 | 0.59692 | 0.88199 | 0.7443 |
| 791.497 | 0.88366 | 0.59573 | 0.87989 | 0.74074 |
| 792.238 | 0.88353 | 0.59484 | 0.87743 | 0.73763 |
| 792.979 | 0.88304 | 0.59392 | 0.87493 | 0.73432 |
| 793.719 | 0.88275 | 0.5931 | 0.87296 | 0.73113 |
| 794.46 | 0.88278 | 0.59235 | 0.87077 | 0.72731 |
| 795.201 | 0.88222 | 0.59175 | 0.86865 | 0.72401 |
| 795.941 | 0.88177 | 0.59098 | 0.86654 | 0.72076 |
| 796.681 | 0.88147 | 0.59026 | 0.86486 | 0.71772 |
| 797.422 | 0.88152 | 0.58968 | 0.8625 | 0.71495 |
| 798.162 | 0.8815 | 0.58878 | 0.86099 | 0.71202 |
| 798.902 | 0.88168 | 0.58818 | 0.85903 | 0.7095 |
| 799.642 | 0.88157 | 0.58757 | 0.85728 | 0.7068 |
| 800.382 | 0.88106 | 0.58688 | 0.85533 | 0.70394 |
| 801.122 | 0.88081 | 0.58605 | 0.85365 | 0.70095 |
| 801.861 | 0.88013 | 0.58551 | 0.85144 | 0.69837 |
| 802.601 | 0.88006 | 0.58481 | 0.84966 | 0.69551 |
| 803.341 | 0.87985 | 0.5844 | 0.84757 | 0.69276 |
| 804.08 | 0.88025 | 0.58392 | 0.84569 | 0.69011 |
| 804.82 | 0.87974 | 0.58352 | 0.84374 | 0.68741 |
| 805.559 | 0.87973 | 0.58285 | 0.84229 | 0.68461 |
| 806.299 | 0.87903 | 0.58241 | 0.84051 | 0.6821 |
| 807.038 | 0.87881 | 0.5818 | 0.83899 | 0.67932 |
| 807.777 | 0.87847 | 0.5813 | 0.83739 | 0.67683 |
| 808.516 | 0.87854 | 0.58087 | 0.83633 | 0.67462 |
| 809.255 | 0.87829 | 0.58072 | 0.83452 | 0.67245 |
| 809.994 | 0.87835 | 0.58042 | 0.83303 | 0.67023 |
| 810.733 | 0.87832 | 0.58008 | 0.83189 | 0.66791 |
| 811.472 | 0.87838 | 0.57973 | 0.83048 | 0.66565 |
| 812.211 | 0.87783 | 0.5795 | 0.82907 | 0.66335 |
| 812.949 | 0.87739 | 0.57917 | 0.82776 | 0.66114 |
| 813.688 | 0.87706 | 0.57872 | 0.82645 | 0.659 |
| 814.427 | 0.87683 | 0.57829 | 0.82492 | 0.65744 |
| 815.165 | 0.87672 | 0.57808 | 0.82336 | 0.65568 |
| 815.903 | 0.87716 | 0.57767 | 0.82166 | 0.65392 |
| 816.642 | 0.87744 | 0.57722 | 0.8208 | 0.65221 |
| 817.38 | 0.87764 | 0.57709 | 0.81986 | 0.65037 |
| 818.118 | 0.87749 | 0.57688 | 0.81859 | 0.64836 |
| 818.856 | 0.877 | 0.57678 | 0.81782 | 0.64656 |
| 819.594 | 0.87667 | 0.57653 | 0.81722 | 0.6448 |
| 820.332 | 0.87678 | 0.57656 | 0.81619 | 0.64333 |
| 821.07 | 0.87699 | 0.5764 | 0.81536 | 0.64179 |
| 821.808 | 0.87724 | 0.5765 | 0.81477 | 0.64039 |
| 822.545 | 0.87799 | 0.57629 | 0.81413 | 0.63897 |
| 823.283 | 0.87811 | 0.57611 | 0.81334 | 0.63774 |
| 824.02 | 0.87836 | 0.57601 | 0.81225 | 0.63645 |
| 824.758 | 0.87792 | 0.57594 | 0.81121 | 0.63518 |
| 825.495 | 0.87733 | 0.5757 | 0.81026 | 0.63388 |
| 826.232 | 0.87699 | 0.57551 | 0.8097 | 0.63274 |
| 826.97 | 0.87697 | 0.57557 | 0.80883 | 0.6314 |
| 827.707 | 0.87688 | 0.57565 | 0.80862 | 0.63047 |
| 828.444 | 0.87736 | 0.57576 | 0.80814 | 0.62968 |
| 829.181 | 0.8781 | 0.57551 | 0.80755 | 0.62887 |
| 829.918 | 0.87823 | 0.57553 | 0.80683 | 0.62813 |
| 830.655 | 0.87846 | 0.57543 | 0.80632 | 0.62726 |
| 831.391 | 0.8784 | 0.57513 | 0.80584 | 0.62625 |
| 832.128 | 0.87828 | 0.57515 | 0.80552 | 0.62539 |
| 832.865 | 0.87838 | 0.57536 | 0.80511 | 0.62492 |
| 833.601 | 0.87821 | 0.5755 | 0.80454 | 0.62443 |
| 834.338 | 0.87848 | 0.57578 | 0.80446 | 0.62399 |
| 835.074 | 0.87879 | 0.57597 | 0.80405 | 0.62341 |
| 835.81 | 0.87916 | 0.5759 | 0.80398 | 0.62315 |
| 836.547 | 0.87889 | 0.57605 | 0.80362 | 0.62268 |
| 837.283 | 0.87915 | 0.57621 | 0.80354 | 0.6221 |
| 838.019 | 0.8791 | 0.57638 | 0.80317 | 0.62182 |
| 838.755 | 0.87902 | 0.57681 | 0.80312 | 0.62197 |
| 839.491 | 0.87895 | 0.57697 | 0.80272 | 0.62157 |
| 840.227 | 0.87924 | 0.57705 | 0.80297 | 0.62135 |
| 840.962 | 0.87955 | 0.57723 | 0.80277 | 0.6213 |
| 841.698 | 0.87957 | 0.57723 | 0.80302 | 0.62132 |
| 842.434 | 0.87967 | 0.57705 | 0.80307 | 0.62091 |
| 843.169 | 0.88032 | 0.57728 | 0.80317 | 0.62088 |
| 843.905 | 0.88065 | 0.57745 | 0.80286 | 0.621 |
| 844.64 | 0.88039 | 0.57774 | 0.80308 | 0.6209 |
| 845.375 | 0.88092 | 0.57786 | 0.80272 | 0.62104 |
| 846.111 | 0.88156 | 0.578 | 0.80253 | 0.62129 |
| 846.846 | 0.88108 | 0.57827 | 0.80258 | 0.62164 |
| 847.581 | 0.88115 | 0.57851 | 0.803 | 0.62164 |
| 848.316 | 0.88185 | 0.57853 | 0.80311 | 0.62211 |
| 849.051 | 0.8822 | 0.57902 | 0.8036 | 0.62224 |
| 849.786 | 0.88196 | 0.57962 | 0.80423 | 0.62273 |
| 850.52 | 0.88205 | 0.57992 | 0.80469 | 0.62298 |
| 851.255 | 0.88239 | 0.58022 | 0.80534 | 0.62358 |
| 851.99 | 0.88246 | 0.58081 | 0.80564 | 0.62403 |
| 852.724 | 0.88243 | 0.58096 | 0.80602 | 0.6247 |
| 853.459 | 0.88277 | 0.58107 | 0.8064 | 0.62526 |
| 854.193 | 0.88335 | 0.58154 | 0.80657 | 0.62605 |
| 854.927 | 0.88308 | 0.58199 | 0.80669 | 0.62662 |
| 855.662 | 0.88286 | 0.58215 | 0.80689 | 0.62731 |
| 856.396 | 0.88278 | 0.58264 | 0.80721 | 0.62813 |
| 857.13 | 0.88326 | 0.5831 | 0.80737 | 0.62892 |
| 857.864 | 0.88344 | 0.58331 | 0.80815 | 0.62976 |
| 858.598 | 0.88431 | 0.58357 | 0.80831 | 0.63059 |
| 859.332 | 0.88482 | 0.58412 | 0.80905 | 0.63145 |
| 860.065 | 0.88528 | 0.58455 | 0.80978 | 0.63207 |
| 860.799 | 0.88498 | 0.58506 | 0.81015 | 0.63288 |
| 861.533 | 0.88516 | 0.58533 | 0.81035 | 0.63359 |
| 862.266 | 0.88516 | 0.58583 | 0.8111 | 0.63458 |
| 863 | 0.88556 | 0.58606 | 0.81177 | 0.63596 |
| 863.733 | 0.88556 | 0.58639 | 0.81268 | 0.63732 |
| 864.466 | 0.88627 | 0.58666 | 0.81411 | 0.63861 |
| 865.2 | 0.88642 | 0.5871 | 0.81498 | 0.63984 |
| 865.933 | 0.88663 | 0.58747 | 0.81566 | 0.64119 |
| 866.666 | 0.88683 | 0.58774 | 0.81656 | 0.64247 |
| 867.399 | 0.88726 | 0.58824 | 0.81737 | 0.64365 |
| 868.132 | 0.88708 | 0.58874 | 0.81781 | 0.64496 |
| 868.865 | 0.88739 | 0.58923 | 0.81882 | 0.64653 |
| 869.597 | 0.88765 | 0.58982 | 0.81976 | 0.64774 |
| 870.33 | 0.88795 | 0.59061 | 0.82079 | 0.64885 |
| 871.063 | 0.88828 | 0.59102 | 0.82105 | 0.65033 |
| 871.795 | 0.88865 | 0.59162 | 0.82185 | 0.65165 |
| 872.528 | 0.88882 | 0.59242 | 0.82255 | 0.65308 |
| 873.26 | 0.88914 | 0.59295 | 0.8238 | 0.65473 |
| 873.992 | 0.88936 | 0.59334 | 0.82461 | 0.65634 |
| 874.725 | 0.88974 | 0.59411 | 0.82618 | 0.65798 |
| 875.457 | 0.89031 | 0.59458 | 0.82749 | 0.65957 |
| 876.189 | 0.8909 | 0.5949 | 0.82884 | 0.66101 |
| 876.921 | 0.89125 | 0.59545 | 0.82997 | 0.66269 |
| 877.653 | 0.89158 | 0.59611 | 0.83096 | 0.6644 |
| 878.385 | 0.89161 | 0.59655 | 0.83189 | 0.66604 |
| 879.116 | 0.89173 | 0.59711 | 0.83287 | 0.6678 |
| 879.848 | 0.89213 | 0.59771 | 0.83393 | 0.66962 |
| 880.58 | 0.89262 | 0.59819 | 0.83498 | 0.67128 |
| 881.311 | 0.89286 | 0.59858 | 0.83639 | 0.67281 |
| 882.043 | 0.89352 | 0.5991 | 0.8375 | 0.67472 |
| 882.774 | 0.89379 | 0.59981 | 0.83859 | 0.67656 |
| 883.505 | 0.89444 | 0.60026 | 0.8396 | 0.6785 |
| 884.236 | 0.89454 | 0.60089 | 0.84047 | 0.68044 |
| 884.968 | 0.89467 | 0.60171 | 0.84141 | 0.68245 |
| 885.699 | 0.89515 | 0.60222 | 0.84243 | 0.68432 |
| 886.43 | 0.89557 | 0.6029 | 0.84352 | 0.68632 |
| 887.161 | 0.89545 | 0.60368 | 0.84478 | 0.68834 |
| 887.891 | 0.89587 | 0.60436 | 0.84636 | 0.69042 |
| 888.622 | 0.89656 | 0.6049 | 0.84776 | 0.69262 |
| 889.353 | 0.8969 | 0.60589 | 0.84934 | 0.69461 |
| 890.083 | 0.89724 | 0.60676 | 0.85074 | 0.69658 |
| 890.814 | 0.8979 | 0.60743 | 0.85169 | 0.69849 |
| 891.544 | 0.898 | 0.60798 | 0.85278 | 0.70056 |
| 892.275 | 0.89839 | 0.60898 | 0.85406 | 0.70261 |
| 893.005 | 0.89866 | 0.60956 | 0.85509 | 0.70472 |
| 893.735 | 0.89938 | 0.6098 | 0.85617 | 0.70693 |
| 894.465 | 0.89949 | 0.6107 | 0.8584 | 0.70913 |
| 895.195 | 0.90021 | 0.61147 | 0.85992 | 0.71131 |
| 895.925 | 0.90042 | 0.61211 | 0.86159 | 0.71332 |
| 896.655 | 0.90068 | 0.61277 | 0.86322 | 0.71521 |
| 897.385 | 0.90101 | 0.61372 | 0.86508 | 0.7176 |
| 898.115 | 0.90154 | 0.61436 | 0.86576 | 0.71995 |
| 898.844 | 0.90198 | 0.61513 | 0.86745 | 0.72201 |
| 899.574 | 0.90254 | 0.6159 | 0.86853 | 0.72448 |
| 900.304 | 0.90342 | 0.6169 | 0.86993 | 0.72713 |
| 901.033 | 0.90375 | 0.61781 | 0.87106 | 0.72927 |
| 901.762 | 0.90435 | 0.61856 | 0.873 | 0.73144 |
| 902.492 | 0.90476 | 0.61949 | 0.87414 | 0.734 |
| 903.221 | 0.90537 | 0.62001 | 0.87553 | 0.73617 |
| 903.95 | 0.90558 | 0.62078 | 0.87724 | 0.73823 |
| 904.679 | 0.90576 | 0.62148 | 0.87915 | 0.74088 |
| 905.408 | 0.90569 | 0.6222 | 0.88071 | 0.74326 |
| 906.137 | 0.90566 | 0.62311 | 0.8823 | 0.74536 |
| 906.866 | 0.90611 | 0.62407 | 0.88422 | 0.74781 |
| 907.594 | 0.90637 | 0.6248 | 0.8858 | 0.75038 |
| 908.323 | 0.90677 | 0.62564 | 0.88718 | 0.7526 |
| 909.051 | 0.90749 | 0.62686 | 0.88873 | 0.75498 |
| 909.78 | 0.90807 | 0.62762 | 0.89037 | 0.75764 |
| 910.508 | 0.90832 | 0.62861 | 0.892 | 0.76037 |
| 911.237 | 0.90865 | 0.62945 | 0.89351 | 0.76296 |
| 911.965 | 0.90957 | 0.6303 | 0.89548 | 0.76541 |
| 912.693 | 0.91026 | 0.63089 | 0.89729 | 0.7679 |
| 913.421 | 0.91091 | 0.6318 | 0.89934 | 0.77034 |
| 914.149 | 0.91139 | 0.63264 | 0.90084 | 0.77271 |
| 914.877 | 0.91185 | 0.63384 | 0.90258 | 0.77528 |
| 915.605 | 0.91213 | 0.63493 | 0.90405 | 0.77772 |
| 916.333 | 0.91216 | 0.63583 | 0.90543 | 0.78032 |
| 917.06 | 0.91275 | 0.63687 | 0.90672 | 0.78299 |
| 917.788 | 0.91312 | 0.63778 | 0.90841 | 0.78532 |
| 918.516 | 0.91385 | 0.63868 | 0.91015 | 0.78776 |
| 919.243 | 0.91407 | 0.63958 | 0.91177 | 0.79054 |
| 919.97 | 0.91468 | 0.64059 | 0.91369 | 0.79314 |
| 920.698 | 0.91521 | 0.64143 | 0.91578 | 0.7959 |
| 921.425 | 0.91557 | 0.64247 | 0.9177 | 0.79872 |
| 922.152 | 0.91632 | 0.64315 | 0.91949 | 0.80141 |
| 922.879 | 0.91699 | 0.64398 | 0.92138 | 0.80378 |
| 923.606 | 0.9177 | 0.64486 | 0.92313 | 0.8064 |
| 924.333 | 0.9181 | 0.64589 | 0.92472 | 0.80848 |
| 925.06 | 0.91895 | 0.64696 | 0.92622 | 0.81089 |
| 925.787 | 0.91902 | 0.64817 | 0.9273 | 0.81336 |
| 926.513 | 0.91949 | 0.6491 | 0.92868 | 0.81578 |
| 927.24 | 0.91984 | 0.65036 | 0.93014 | 0.8182 |
| 927.966 | 0.92002 | 0.65125 | 0.93183 | 0.82109 |
| 928.693 | 0.9203 | 0.652 | 0.93366 | 0.82362 |
| 929.419 | 0.92091 | 0.65278 | 0.93552 | 0.82639 |
| 930.146 | 0.9212 | 0.65403 | 0.93758 | 0.82888 |
| 930.872 | 0.92157 | 0.65498 | 0.93975 | 0.83168 |
| 931.598 | 0.92237 | 0.65598 | 0.94149 | 0.83445 |
| 932.324 | 0.92266 | 0.65687 | 0.94317 | 0.83711 |
| 933.05 | 0.92256 | 0.65816 | 0.94522 | 0.83948 |
| 933.776 | 0.92384 | 0.65916 | 0.94694 | 0.84231 |
| 934.502 | 0.92469 | 0.65996 | 0.94854 | 0.84502 |
| 935.227 | 0.92575 | 0.66105 | 0.95023 | 0.84715 |
| 935.953 | 0.92629 | 0.66252 | 0.9524 | 0.84937 |
| 936.679 | 0.92757 | 0.66331 | 0.95437 | 0.85197 |
| 937.404 | 0.92757 | 0.66441 | 0.95652 | 0.85427 |
| 938.129 | 0.92795 | 0.66595 | 0.95796 | 0.85687 |
| 938.855 | 0.92758 | 0.66714 | 0.95973 | 0.85948 |
| 939.58 | 0.92864 | 0.66825 | 0.96106 | 0.86278 |
| 940.305 | 0.92903 | 0.66964 | 0.96329 | 0.86536 |
| 941.03 | 0.9296 | 0.67054 | 0.96453 | 0.86829 |
| 941.755 | 0.93042 | 0.67138 | 0.96651 | 0.87118 |
| 942.48 | 0.93109 | 0.67277 | 0.96888 | 0.87433 |
| 943.205 | 0.93178 | 0.67358 | 0.97139 | 0.8768 |
| 943.93 | 0.93249 | 0.67473 | 0.97257 | 0.8799 |
| 944.655 | 0.93332 | 0.67593 | 0.97475 | 0.88272 |
| 945.379 | 0.93358 | 0.67735 | 0.97692 | 0.88559 |
| 946.104 | 0.93458 | 0.67835 | 0.9781 | 0.8881 |
| 946.828 | 0.93506 | 0.67988 | 0.9799 | 0.89054 |
| 947.553 | 0.93519 | 0.68099 | 0.98195 | 0.893 |
| 948.277 | 0.93535 | 0.68235 | 0.98348 | 0.8955 |
| 949.001 | 0.93628 | 0.68335 | 0.9856 | 0.89758 |
| 949.725 | 0.93672 | 0.68469 | 0.98736 | 0.90008 |
| 950.449 | 0.93709 | 0.68582 | 0.98896 | 0.90299 |
| 951.173 | 0.93823 | 0.68704 | 0.9909 | 0.90576 |
| 951.897 | 0.93898 | 0.68798 | 0.99272 | 0.90859 |
| 952.621 | 0.93934 | 0.68933 | 0.99411 | 0.9114 |
| 953.345 | 0.94003 | 0.69013 | 0.99616 | 0.91384 |
| 954.068 | 0.94097 | 0.69153 | 0.99804 | 0.91609 |
| 954.792 | 0.94127 | 0.69264 | 0.99968 | 0.91823 |
| 955.515 | 0.94227 | 0.6941 | 1.00193 | 0.92079 |
| 956.239 | 0.94311 | 0.69538 | 1.00414 | 0.92326 |
| 956.962 | 0.94331 | 0.69667 | 1.0064 | 0.92574 |
| 957.686 | 0.94379 | 0.69764 | 1.00814 | 0.92805 |
| 958.409 | 0.9443 | 0.699 | 1.01027 | 0.93045 |
| 959.132 | 0.94458 | 0.70014 | 1.01218 | 0.93264 |
| 959.855 | 0.94492 | 0.70146 | 1.01363 | 0.93483 |
| 960.578 | 0.94577 | 0.70284 | 1.01558 | 0.93752 |
| 961.301 | 0.94648 | 0.70447 | 1.01708 | 0.9402 |
| 962.023 | 0.9471 | 0.70574 | 1.01873 | 0.94281 |
| 962.746 | 0.94734 | 0.70692 | 1.01995 | 0.9449 |
| 963.469 | 0.94797 | 0.70753 | 1.02159 | 0.94693 |
| 964.191 | 0.94853 | 0.70862 | 1.02208 | 0.9483 |
| 964.914 | 0.94894 | 0.7093 | 1.02387 | 0.95073 |
| 965.636 | 0.94972 | 0.71018 | 1.02476 | 0.95242 |
| 966.359 | 0.95029 | 0.7115 | 1.02648 | 0.95398 |
| 967.081 | 0.9512 | 0.71269 | 1.02756 | 0.9559 |
| 967.803 | 0.95183 | 0.7135 | 1.02933 | 0.95806 |
| 968.525 | 0.95246 | 0.71446 | 1.03049 | 0.95876 |
| 969.247 | 0.95328 | 0.71551 | 1.03212 | 0.96069 |
| 969.969 | 0.95439 | 0.71641 | 1.03325 | 0.9631 |
| 970.691 | 0.95466 | 0.7176 | 1.03514 | 0.96498 |
| 971.412 | 0.95516 | 0.71907 | 1.03688 | 0.96672 |
| 972.134 | 0.95583 | 0.72008 | 1.03868 | 0.96932 |
| 972.856 | 0.9561 | 0.72123 | 1.04014 | 0.97099 |
| 973.577 | 0.95682 | 0.72229 | 1.04186 | 0.97269 |
| 974.299 | 0.95775 | 0.72344 | 1.04289 | 0.97459 |
| 975.02 | 0.95839 | 0.72431 | 1.04443 | 0.97653 |
| 975.741 | 0.95898 | 0.72566 | 1.04582 | 0.979 |
| 976.462 | 0.959 | 0.72701 | 1.04705 | 0.98105 |
| 977.184 | 0.95945 | 0.728 | 1.04888 | 0.98304 |
| 977.905 | 0.96 | 0.72912 | 1.0509 | 0.98511 |
| 978.626 | 0.96035 | 0.7304 | 1.05201 | 0.9875 |
| 979.346 | 0.96047 | 0.73143 | 1.05355 | 0.98901 |
| 980.067 | 0.96179 | 0.73231 | 1.05544 | 0.99064 |
| 980.788 | 0.96246 | 0.73364 | 1.05633 | 0.99263 |
| 981.509 | 0.96244 | 0.73461 | 1.05821 | 0.99438 |
| 982.229 | 0.96338 | 0.73555 | 1.05912 | 0.99618 |
| 982.95 | 0.96407 | 0.73661 | 1.06082 | 0.99767 |
| 983.67 | 0.96446 | 0.73751 | 1.06221 | 0.9996 |
| 984.39 | 0.96491 | 0.73892 | 1.06358 | 1.00154 |
| 985.111 | 0.9652 | 0.74015 | 1.06452 | 1.00319 |
| 985.831 | 0.96564 | 0.74126 | 1.06619 | 1.00428 |
| 986.551 | 0.96619 | 0.74233 | 1.06725 | 1.006 |
| 987.271 | 0.96666 | 0.74377 | 1.06866 | 1.00776 |
| 987.991 | 0.96638 | 0.74441 | 1.07012 | 1.00922 |
| 988.71 | 0.96707 | 0.7455 | 1.07105 | 1.01059 |
| 989.43 | 0.96798 | 0.7467 | 1.07274 | 1.01299 |
| 990.15 | 0.96842 | 0.748 | 1.07424 | 1.01505 |
| 990.869 | 0.96906 | 0.74903 | 1.0757 | 1.01696 |
| 991.589 | 0.97026 | 0.7503 | 1.07742 | 1.01894 |
| 992.308 | 0.97119 | 0.75135 | 1.07868 | 1.02145 |
| 993.028 | 0.97122 | 0.75273 | 1.07991 | 1.02255 |
| 993.747 | 0.97171 | 0.75386 | 1.08064 | 1.02363 |
| 994.466 | 0.97165 | 0.7551 | 1.08166 | 1.02532 |
| 995.185 | 0.97258 | 0.75587 | 1.08232 | 1.02651 |
| 995.904 | 0.97275 | 0.75724 | 1.08349 | 1.02747 |
| 996.623 | 0.97345 | 0.75802 | 1.08519 | 1.0288 |
| 997.342 | 0.97396 | 0.75853 | 1.08604 | 1.02971 |
| 998.061 | 0.97495 | 0.75889 | 1.08693 | 1.03006 |
| 998.78 | 0.97408 | 0.7599 | 1.08827 | 1.03031 |
| 999.498 | 0.97498 | 0.75962 | 1.09117 | 1.02996 |
| 1000.217 | 0.87997 | 0.80466 | 1.03217 | 0.85115 |
| 1000.935 | 0.89157 | 0.82854 | 0.93172 | 0.86194 |
| 1001.654 | 0.90038 | 0.82196 | 0.90267 | 0.86571 |
| 1002.372 | 0.90899 | 0.82597 | 0.89723 | 0.88185 |
| 1003.09 | 0.91303 | 0.82858 | 0.90018 | 0.88572 |
| 1003.808 | 0.91037 | 0.84274 | 0.90573 | 0.87993 |
| 1004.526 | 0.97227 | 0.76261 | 1.07953 | 1.02531 |
| 1005.244 | 0.97465 | 0.7625 | 1.08655 | 1.03114 |
| 1005.962 | 0.97498 | 0.76293 | 1.0883 | 1.03284 |
| 1006.68 | 0.97566 | 0.7631 | 1.08987 | 1.03437 |
| 1007.398 | 0.97583 | 0.76321 | 1.09075 | 1.03527 |
| 1008.115 | 0.97627 | 0.76404 | 1.09227 | 1.03641 |
| 1008.833 | 0.97619 | 0.76441 | 1.09228 | 1.03655 |
| 1009.55 | 0.97658 | 0.76442 | 1.09314 | 1.03701 |
| 1010.268 | 0.9763 | 0.76442 | 1.09324 | 1.03695 |
| 1010.985 | 0.97668 | 0.76415 | 1.09416 | 1.03722 |
| 1011.702 | 0.96542 | 0.78568 | 1.0432 | 1.06021 |
| 1012.419 | 0.93035 | 0.7989 | 1.02314 | 0.99611 |

Date of figure 4(d)

| categories | 660nm-area | *C** | w(NiO)% |
| --- | --- | --- | --- |
| 15-25 | 18.5508 | 19.0525 | 0.47 |
|  | 22.525 | 19.4745 | 0.49 |
|  | 24.4733 | 22.5881 | 0.55 |
|  | 24.7831 | 23.462 | 0.534 |
| Mean value | 22.583 | 21.144 | 0.511 |
| 25-35 | 26.02 | 21.4118 | 0.551 |
|  | 26.0291 | 22.5046 | 0.38 |
|  | 28.5954 | 24.4992 | 0.748 |
|  | 30.0892 | 28.7647 | 1.023 |
|  | 33.0791 | 30.5957 | 0.759 |
|  | 33.946 | 33.9172 | 1.377 |
| Mean value | 29.626 | 26.949 | 0.806 |
| 35-45 | 37.6319 | 29.7591 | 0.884 |
|  | 39.9599 | 39.4808 | 1.267 |
|  | 40.8644 | 39.4105 | 1.66 |
|  | 41.2336 | 43.0107 | 2.525 |
|  | 42.0388 | 39.6197 | 1.127 |
|  | 42.1363 | 39.636 | 1.286 |
|  | 42.3504 | 41.3131 | 1.536 |
|  | 42.4704 | 36.0725 | 1.082 |
|  | 42.6217 | 39.4719 | 1.482 |
|  | 44.8483 | 37.567 | 1.197 |
| Mean value | 41.616 | 38.534 | 1.405 |
| 45-55 | 45.1626 | 45.0209 | 2.752 |
|  | 46.8814 | 43.3922 | 2.292 |
|  | 46.9686 | 40.9754 | 1.275 |
|  | 47.2135 | 38.868 | 1.192 |
|  | 48.0133 | 41.1179 | 1.443 |
|  | 51.5531 | 45.3689 | 1.799 |
|  | 52.6954 | 47.3936 | 2.016 |
|  | 52.7337 | 45.2403 | 2.972 |
|  | 53.2669 | 44.9955 | 3.007 |
|  | 54.611 | 46.6596 | 2.762 |
| Mean value | 49.910 | 43.903 | 2.151 |
| 55-65 | 57.1567 | 47.1264 | 2.958 |
|  | 57.4362 | 42.2841 | 2.949 |
|  | 57.9473 | 45.3329 | 2.299 |
|  | 58.2641 | 46.23 | 2.156 |
|  | 60.1502 | 48.4054 | 2.345 |
|  | 60.8085 | 41.2159 | 1.716 |
| Mean value | 58.627 | 45.099 | 2.404 |
| 65-75 | 66.3169 | 45.9051 | 2.619 |
|  | 68.0551 | 47.2674 | 3.27 |
|  | 74.2546 | 50.087 | 3.752 |
|  | 74.318 | 47.5353 | 3.25 |
| Mean value | 70.736 | 47.699 | 3.223 |
| 85-95 | 93.3794 | 52.8176 | 5.631 |
| Mean value | 93.379 | 52.818 | 5.631 |

Date of figure 4(e)

| categories | sum of absorption peaks at 380 and 660nm | *L** | w(Cr_2_O_3_)% |
| --- | --- | --- | --- |
| 1.4-1.6 | 1.466952 | 63.99 | 0.014 |
| Mean value | 1.46695228 | 63.99 | 0.014 |
| 1.6-1.8 | 1.669012 | 58.38 | 0.01 |
|  | 1.704522 | 57.54 | 0.012 |
|  | 1.799093 | 56.51 | 0.014 |
| Mean value | 1.724208945 | 57.47666667 | 0.012 |
| 1.8-2.0 | 1.82156 | 53.6 | 0.019 |
|  | 1.826507 | 54.26 | 0.011 |
|  | 1.845131 | 52.37 | 0.019 |
|  | 1.854759 | 49.94 | 0.015 |
|  | 1.858884 | 56.79 | 0.013 |
|  | 1.875853 | 52.22 | 0.008 |
|  | 1.898736 | 51.58 | 0.019 |
|  | 1.907202 | 50.22 | 0.015 |
|  | 1.92386 | 52.87 | 0.01 |
|  | 1.940353 | 53.02 | 0.014 |
|  | 1.944705 | 50.43 | 0.026 |
|  | 1.948041 | 44.82 | 0.023 |
|  | 1.954983 | 47.81 | 0.017 |
|  | 1.977234 | 48.28 | 0.025 |
|  | 1.982513 | 47.53 | 0.022 |
|  | 1.987472 | 42.21 | 0.012 |
|  | 1.995032 | 52.57 | 0.01 |
| Mean value | 1.91428383 | 50.61882353 | 0.016352941 |
| 2.0-2.2 | 2.026033 | 50.68 | 0.014 |
|  | 2.04409 | 50.13 | 0.018 |
|  | 2.047979 | 49.75 | 0.013 |
|  | 2.050382 | 44.94 | 0.029 |
|  | 2.056693 | 43.65 | 0.027 |
|  | 2.057828 | 66.1 | 0.014 |
|  | 2.061395 | 47.14 | 0.027 |
|  | 2.083684 | 48.73 | 0.015 |
|  | 2.085747 | 45.76 | 0.022 |
|  | 2.08892 | 41.83 | 0.033 |
|  | 2.089073 | 49.77 | 0.013 |
|  | 2.094399 | 41.73 | 0.035 |
|  | 2.10111 | 45.4 | 0.021 |
|  | 2.113652 | 47.25 | 0.016 |
|  | 2.11993 | 39.39 | 0.032 |
|  | 2.135836 | 46.73 | 0.014 |
|  | 2.157593 | 46.92 | 0.018 |
|  | 2.168947 | 41.02 | 0.035 |
| Mean value | 2.087960527 | 47.05111111 | 0.022 |
| 2.2-2.4 | 2.206832 | 41.07 | 0.03 |
|  | 2.34815 | 49.89 | 0.018 |
| Mean value | 2.277490848 | 45.48 | 0.024 |

Date of figure 4(f)

| a* | b* | 660+386 | 660 area |
| --- | --- | --- | --- |
| -36.93 | 12.12 | 1.898736 | 47.2135 |
| -40.34 | 14.92 | 2.056693 | 41.2336 |
| -51.71 | 10.76 | 1.995032 | 93.3794 |
| -36.69 | 14.58 | 1.954983 | 39.9599 |
| -47.46 | 9.52 | 2.089073 | 60.1502 |
| -37.9 | 21.13 | 2.08892 | 46.8814 |
| -48.33 | 13.15 | 2.061395 | 74.2546 |
| -35.02 | 8.65 | 2.047979 | 42.4704 |
| -46.32 | 10.68 | 1.875853 | 74.318 |
| -42.29 | 16.07 | 1.948041 | 52.7337 |
| -23.04 | 4.43 | 1.669012 | 24.7831 |
| -22.24 | 3.95 | 1.826507 | 24.4733 |
| -29.59 | 7.78 | 1.92386 | 33.0791 |
| -39.07 | 12.35 | 1.907202 | 46.9686 |
| -38.17 | 9.81 | 2.113652 | 40.8644 |
| -44.28 | 16.13 | 2.050382 | 57.1567 |
| -43.21 | 13.71 | 2.085747 | 57.9473 |
| -43.21 | 19.16 | 2.206832 | 68.0551 |
| -43.81 | 13.71 | 2.10111 | 66.3169 |
| -41.51 | 17.43 | 2.168947 | 45.1626 |
| -39.64 | 21.29 | 2.094399 | 53.2669 |
| -44.81 | 11.37 | 2.04409 | 58.2641 |
| -27.65 | 7.93 | 2.026033 | 30.0892 |
| -33.46 | 5.55 | 2.083684 | 33.946 |
| -31.61 | 20.3 | 1.854759 | 44.8483 |
| -24.05 | 4.67 | 1.858884 | 28.5954 |
| -44.31 | 14.62 | 1.977234 | 54.611 |
| -37.58 | 12.6 | 1.82156 | 42.1363 |
| -37.67 | 11.79 | 1.944705 | 42.6217 |
| -21.33 | 1.87 | 1.704522 | 26.02 |
| -39.89 | 10.37 | 2.34815 | 60.8085 |
| -28.96 | 6.85 | 1.466952 | 37.6319 |
| -19.11 | 3.75 | 1.940353 | 22.525 |
| -45.34 | 13.8 | 1.845131 | 52.6954 |
| -37.98 | 11.28 | 2.057828 | 42.0388 |
| -37.8 | 18.95 | 2.11993 | 57.4362 |
| -21.69 | 6 | 1.799093 | 26.0291 |
| -43.2 | 13.86 | 2.135836 | 51.5531 |
| -38.54 | 14.33 | 1.982513 | 48.0133 |
| -38.16 | 15.83 | 2.157593 | 42.3504 |
| -18.81 | 3.03 | 1.987472 | 18.5508 |

Date of figure 4(g)(h)

| Raman shift | FD-01 | F-01 |  | F-10 | FD-20 | FD-21 |
| --- | --- | --- | --- | --- | --- | --- |
| 100.377 | 0.00502 | 0.02518 |  | 0.03602 | 0.02577 | 0.00148 |
| 100.899 | 0.00461 | 0.02248 |  | 0.04264 | 0.02538 | 0.00969 |
| 101.421 | 0.00781 | 0.02116 |  | 0.03954 | 0.02331 | 0.00836 |
| 101.943 | 0.00712 | 0.01979 |  | 0.04087 | 0.02160 | 0.00731 |
| 102.465 | 0.00683 | 0.02063 |  | 0.04191 | 0.02037 | 0.00694 |
| 102.988 | 0.00686 | 0.02106 |  | 0.04336 | 0.01955 | 0.00651 |
| 103.51 | 0.00720 | 0.02154 |  | 0.04394 | 0.01925 | 0.00601 |
| 104.034 | 0.00749 | 0.02212 |  | 0.04399 | 0.01940 | 0.00609 |
| 104.556 | 0.00793 | 0.02315 |  | 0.04347 | 0.02006 | 0.00565 |
| 105.078 | 0.00862 | 0.02406 |  | 0.04434 | 0.02129 | 0.00870 |
| 105.6 | 0.00960 | 0.02480 |  | 0.04534 | 0.02325 | 0.00972 |
| 106.122 | 0.01077 | 0.02553 |  | 0.04641 | 0.02587 | 0.01066 |
| 106.643 | 0.01199 | 0.02627 |  | 0.04735 | 0.02914 | 0.01158 |
| 107.165 | 0.01311 | 0.02702 |  | 0.04825 | 0.03224 | 0.01224 |
| 107.687 | 0.01228 | 0.02776 |  | 0.04858 | 0.03463 | 0.01265 |
| 108.209 | 0.01009 | 0.02681 |  | 0.04738 | 0.03087 | 0.01239 |
| 108.731 | 0.00901 | 0.02606 |  | 0.04657 | 0.03017 | 0.01214 |
| 109.254 | 0.00813 | 0.02563 |  | 0.04619 | 0.02993 | 0.01096 |
| 109.776 | 0.00782 | 0.02543 |  | 0.04656 | 0.02993 | 0.00973 |
| 110.298 | 0.00805 | 0.02550 |  | 0.04795 | 0.03083 | 0.00967 |
| 110.819 | 0.00881 | 0.02581 |  | 0.05032 | 0.03177 | 0.01094 |
| 111.341 | 0.00962 | 0.02632 |  | 0.04665 | 0.03249 | 0.01251 |
| 111.863 | 0.01041 | 0.02712 |  | 0.04626 | 0.03306 | 0.01389 |
| 112.384 | 0.01120 | 0.02818 |  | 0.04679 | 0.03411 | 0.01485 |
| 112.906 | 0.01197 | 0.02956 |  | 0.04785 | 0.03421 | 0.01572 |
| 113.427 | 0.01277 | 0.03113 |  | 0.04998 | 0.03309 | 0.01662 |
| 113.949 | 0.01359 | 0.03260 |  | 0.05219 | 0.03265 | 0.01706 |
| 114.47 | 0.01445 | 0.03393 |  | 0.05422 | 0.03272 | 0.01632 |
| 114.992 | 0.01536 | 0.03457 |  | 0.05609 | 0.03421 | 0.01702 |
| 115.513 | 0.01631 | 0.03506 |  | 0.05778 | 0.03643 | 0.01854 |
| 116.034 | 0.01740 | 0.03518 |  | 0.05922 | 0.03891 | 0.02008 |
| 116.556 | 0.01855 | 0.03544 |  | 0.06045 | 0.04172 | 0.02154 |
| 117.077 | 0.01946 | 0.03646 |  | 0.06176 | 0.04461 | 0.02319 |
| 117.598 | 0.02028 | 0.03801 |  | 0.06368 | 0.04711 | 0.02400 |
| 118.119 | 0.02050 | 0.04007 |  | 0.06638 | 0.04914 | 0.02512 |
| 118.641 | 0.02113 | 0.04261 |  | 0.06921 | 0.05110 | 0.02617 |
| 119.162 | 0.02212 | 0.04566 |  | 0.07187 | 0.05271 | 0.02829 |
| 119.683 | 0.02385 | 0.04918 |  | 0.07451 | 0.05419 | 0.03089 |
| 120.204 | 0.02819 | 0.05320 |  | 0.07684 | 0.05555 | 0.03484 |
| 120.725 | 0.03100 | 0.05770 |  | 0.07952 | 0.05789 | 0.03885 |
| 121.246 | 0.03382 | 0.06267 |  | 0.08364 | 0.06212 | 0.04388 |
| 121.767 | 0.03882 | 0.06812 |  | 0.08866 | 0.06796 | 0.04895 |
| 122.288 | 0.04426 | 0.07448 |  | 0.09558 | 0.07534 | 0.05424 |
| 122.809 | 0.04986 | 0.08268 |  | 0.10513 | 0.08430 | 0.06033 |
| 123.33 | 0.05594 | 0.09341 |  | 0.11759 | 0.09501 | 0.06948 |
| 123.851 | 0.06296 | 0.10673 |  | 0.13303 | 0.10769 | 0.08057 |
| 124.37 | 0.07025 | 0.12375 |  | 0.15164 | 0.12398 | 0.09375 |
| 124.891 | 0.08198 | 0.14507 |  | 0.17370 | 0.14425 | 0.10872 |
| 125.411 | 0.09454 | 0.17025 |  | 0.19895 | 0.16773 | 0.12680 |
| 125.932 | 0.10964 | 0.19950 |  | 0.23139 | 0.19350 | 0.14968 |
| 126.453 | 0.12538 | 0.23256 |  | 0.26957 | 0.21953 | 0.17358 |
| 126.974 | 0.13902 | 0.26914 |  | 0.31298 | 0.24403 | 0.19805 |
| 127.495 | 0.15009 | 0.30925 |  | 0.35170 | 0.26442 | 0.22275 |
| 128.015 | 0.15217 | 0.34559 |  | 0.38290 | 0.27825 | 0.23733 |
| 128.536 | 0.15181 | 0.35767 |  | 0.38264 | 0.28458 | 0.23426 |
| 129.056 | 0.14508 | 0.34257 |  | 0.35699 | 0.28089 | 0.22683 |
| 129.575 | 0.13513 | 0.31945 |  | 0.33454 | 0.25830 | 0.21419 |
| 130.095 | 0.12317 | 0.27797 |  | 0.29372 | 0.23798 | 0.19709 |
| 130.616 | 0.10941 | 0.24422 |  | 0.25393 | 0.21387 | 0.17630 |
| 131.137 | 0.09593 | 0.20850 |  | 0.21490 | 0.18875 | 0.15381 |
| 131.657 | 0.08449 | 0.18034 |  | 0.18217 | 0.16583 | 0.13711 |
| 132.177 | 0.07431 | 0.15584 |  | 0.15311 | 0.15482 | 0.12211 |
| 132.698 | 0.06572 | 0.13494 |  | 0.13948 | 0.14161 | 0.10550 |
| 133.216 | 0.06052 | 0.11791 |  | 0.12758 | 0.13022 | 0.09246 |
| 133.737 | 0.05563 | 0.10504 |  | 0.11669 | 0.12030 | 0.08207 |
| 134.257 | 0.05136 | 0.09516 |  | 0.10726 | 0.11174 | 0.07334 |
| 134.777 | 0.04765 | 0.08757 |  | 0.09926 | 0.10434 | 0.06775 |
| 135.297 | 0.04446 | 0.07978 |  | 0.09287 | 0.09815 | 0.06302 |
| 135.818 | 0.04189 | 0.07318 |  | 0.08818 | 0.09324 | 0.05890 |
| 136.336 | 0.03991 | 0.06810 |  | 0.08432 | 0.08958 | 0.05554 |
| 136.856 | 0.03842 | 0.06406 |  | 0.08100 | 0.08716 | 0.05290 |
| 137.376 | 0.03755 | 0.06134 |  | 0.07859 | 0.08591 | 0.05095 |
| 137.896 | 0.03719 | 0.05988 |  | 0.07652 | 0.08459 | 0.04968 |
| 138.416 | 0.03738 | 0.05933 |  | 0.07557 | 0.08150 | 0.04909 |
| 138.934 | 0.03721 | 0.05911 |  | 0.07507 | 0.08036 | 0.04916 |
| 139.454 | 0.03638 | 0.05815 |  | 0.07487 | 0.07928 | 0.04991 |
| 139.974 | 0.03558 | 0.05776 |  | 0.07466 | 0.07933 | 0.05137 |
| 140.494 | 0.03481 | 0.05734 |  | 0.07410 | 0.08017 | 0.05327 |
| 141.012 | 0.03399 | 0.05697 |  | 0.07348 | 0.08185 | 0.05508 |
| 141.532 | 0.03324 | 0.05661 |  | 0.07287 | 0.08417 | 0.05586 |
| 142.052 | 0.03388 | 0.05621 |  | 0.07223 | 0.08716 | 0.05603 |
| 142.572 | 0.03425 | 0.05572 |  | 0.07162 | 0.09055 | 0.05524 |
| 143.09 | 0.03435 | 0.05484 |  | 0.07103 | 0.09145 | 0.05425 |
| 143.609 | 0.03473 | 0.05429 |  | 0.07057 | 0.09108 | 0.05304 |
| 144.129 | 0.03526 | 0.05373 |  | 0.07023 | 0.08957 | 0.05230 |
| 144.649 | 0.03455 | 0.05311 |  | 0.07000 | 0.08688 | 0.05020 |
| 145.167 | 0.03339 | 0.05245 |  | 0.06984 | 0.08310 | 0.04771 |
| 145.686 | 0.03228 | 0.05173 |  | 0.06978 | 0.07930 | 0.04501 |
| 146.206 | 0.03135 | 0.05095 |  | 0.06976 | 0.07611 | 0.04208 |
| 146.723 | 0.03130 | 0.05010 |  | 0.06993 | 0.07315 | 0.04002 |
| 147.243 | 0.03079 | 0.04918 |  | 0.06950 | 0.06990 | 0.03892 |
| 147.763 | 0.03083 | 0.04820 |  | 0.06789 | 0.06742 | 0.03797 |
| 148.28 | 0.03018 | 0.04715 |  | 0.06678 | 0.06559 | 0.03736 |
| 148.8 | 0.02980 | 0.04604 |  | 0.06599 | 0.06461 | 0.03765 |
| 149.319 | 0.02930 | 0.04484 |  | 0.06524 | 0.06362 | 0.03640 |
| 149.836 | 0.02916 | 0.04360 |  | 0.06465 | 0.06268 | 0.03561 |
| 150.356 | 0.02972 | 0.04246 |  | 0.06423 | 0.06192 | 0.03535 |
| 150.875 | 0.03080 | 0.04143 |  | 0.06392 | 0.06028 | 0.03250 |
| 151.392 | 0.03127 | 0.04052 |  | 0.06372 | 0.05845 | 0.03131 |
| 151.912 | 0.03041 | 0.04011 |  | 0.06362 | 0.05691 | 0.03060 |
| 152.431 | 0.02737 | 0.04013 |  | 0.06358 | 0.05669 | 0.03154 |
| 152.948 | 0.02551 | 0.04036 |  | 0.06360 | 0.05784 | 0.03088 |
| 153.468 | 0.02614 | 0.04032 |  | 0.06355 | 0.05885 | 0.03089 |
| 153.987 | 0.02636 | 0.04016 |  | 0.06361 | 0.05923 | 0.03141 |
| 154.504 | 0.02680 | 0.03993 |  | 0.06336 | 0.05944 | 0.03065 |
| 155.023 | 0.02612 | 0.03958 |  | 0.06298 | 0.05773 | 0.02802 |
| 155.54 | 0.02566 | 0.03907 |  | 0.06229 | 0.05610 | 0.02887 |
| 156.059 | 0.02537 | 0.03845 |  | 0.06122 | 0.05395 | 0.02869 |
| 156.578 | 0.02531 | 0.03768 |  | 0.06047 | 0.05270 | 0.02842 |
| 157.095 | 0.02416 | 0.03684 |  | 0.06006 | 0.05239 | 0.02825 |
| 157.614 | 0.02347 | 0.03623 |  | 0.05963 | 0.05300 | 0.02815 |
| 158.131 | 0.02257 | 0.03585 |  | 0.05991 | 0.05459 | 0.02812 |
| 158.65 | 0.02254 | 0.03609 |  | 0.06025 | 0.05580 | 0.02799 |
| 159.169 | 0.02321 | 0.03624 |  | 0.06086 | 0.05648 | 0.02764 |
| 159.686 | 0.02429 | 0.03662 |  | 0.06145 | 0.05689 | 0.02688 |
| 160.205 | 0.02546 | 0.03647 |  | 0.06166 | 0.05598 | 0.02650 |
| 160.722 | 0.02650 | 0.03653 |  | 0.06167 | 0.05427 | 0.02782 |
| 161.24 | 0.02742 | 0.03660 |  | 0.06125 | 0.05329 | 0.02746 |
| 161.757 | 0.02812 | 0.03697 |  | 0.06088 | 0.05276 | 0.02718 |
| 162.276 | 0.02767 | 0.03864 |  | 0.06032 | 0.05276 | 0.02694 |
| 162.792 | 0.02775 | 0.04117 |  | 0.05961 | 0.05325 | 0.02683 |
| 163.311 | 0.02805 | 0.04177 |  | 0.06017 | 0.05554 | 0.02693 |
| 163.83 | 0.02861 | 0.04129 |  | 0.06244 | 0.05613 | 0.02735 |
| 164.346 | 0.02914 | 0.04087 |  | 0.06370 | 0.05727 | 0.02813 |
| 164.865 | 0.02960 | 0.04082 |  | 0.06402 | 0.05809 | 0.02904 |
| 165.381 | 0.02986 | 0.04130 |  | 0.06333 | 0.05894 | 0.03074 |
| 165.9 | 0.02995 | 0.04170 |  | 0.06283 | 0.05973 | 0.03201 |
| 166.416 | 0.03004 | 0.04224 |  | 0.06276 | 0.06078 | 0.03311 |
| 166.935 | 0.03040 | 0.04298 |  | 0.06213 | 0.06192 | 0.03398 |
| 167.451 | 0.03096 | 0.04386 |  | 0.06215 | 0.06300 | 0.03437 |
| 167.97 | 0.03237 | 0.04494 |  | 0.06285 | 0.06391 | 0.03466 |
| 168.486 | 0.03368 | 0.04538 |  | 0.06387 | 0.06451 | 0.03491 |
| 169.005 | 0.03476 | 0.04520 |  | 0.06489 | 0.06392 | 0.03522 |
| 169.521 | 0.03477 | 0.04525 |  | 0.06614 | 0.06147 | 0.03540 |
| 170.037 | 0.03479 | 0.04547 |  | 0.06733 | 0.06223 | 0.03603 |
| 170.555 | 0.03483 | 0.04588 |  | 0.06849 | 0.06489 | 0.03800 |
| 171.071 | 0.03492 | 0.04650 |  | 0.06936 | 0.06656 | 0.03889 |
| 171.59 | 0.03549 | 0.04713 |  | 0.07041 | 0.06765 | 0.03911 |
| 172.106 | 0.03602 | 0.04803 |  | 0.07039 | 0.06826 | 0.03936 |
| 172.624 | 0.03674 | 0.04915 |  | 0.07011 | 0.06874 | 0.03973 |
| 173.14 | 0.03744 | 0.05020 |  | 0.06986 | 0.06934 | 0.04025 |
| 173.658 | 0.03801 | 0.05099 |  | 0.07101 | 0.06983 | 0.04098 |
| 174.174 | 0.03849 | 0.05118 |  | 0.07277 | 0.07003 | 0.04185 |
| 174.69 | 0.03930 | 0.05154 |  | 0.07541 | 0.07027 | 0.04305 |
| 175.208 | 0.04089 | 0.05198 |  | 0.07788 | 0.07081 | 0.04458 |
| 175.724 | 0.04206 | 0.05263 |  | 0.07964 | 0.07173 | 0.04605 |
| 176.242 | 0.04329 | 0.05412 |  | 0.08112 | 0.07299 | 0.04750 |
| 176.758 | 0.04454 | 0.05610 |  | 0.08096 | 0.07456 | 0.04925 |
| 177.274 | 0.04584 | 0.05822 |  | 0.08053 | 0.07652 | 0.05121 |
| 177.792 | 0.04715 | 0.06097 |  | 0.08055 | 0.07887 | 0.05161 |
| 178.307 | 0.04850 | 0.06332 |  | 0.08075 | 0.08154 | 0.05182 |
| 178.825 | 0.04987 | 0.06497 |  | 0.08098 | 0.08429 | 0.05334 |
| 179.341 | 0.05124 | 0.06619 |  | 0.08192 | 0.08697 | 0.05852 |
| 179.857 | 0.05268 | 0.06742 |  | 0.08289 | 0.08953 | 0.05890 |
| 180.374 | 0.05439 | 0.06811 |  | 0.08413 | 0.09194 | 0.05939 |
| 180.89 | 0.05636 | 0.06935 |  | 0.08528 | 0.09412 | 0.05977 |
| 181.406 | 0.05797 | 0.07075 |  | 0.08642 | 0.09621 | 0.06079 |
| 181.923 | 0.05967 | 0.07224 |  | 0.08764 | 0.09817 | 0.06090 |
| 182.439 | 0.06142 | 0.07353 |  | 0.08837 | 0.09997 | 0.06235 |
| 182.954 | 0.06340 | 0.07511 |  | 0.09045 | 0.10175 | 0.06497 |
| 183.472 | 0.06570 | 0.07751 |  | 0.09256 | 0.10420 | 0.06796 |
| 183.987 | 0.06835 | 0.08038 |  | 0.09515 | 0.10662 | 0.07140 |
| 184.503 | 0.07153 | 0.08366 |  | 0.09828 | 0.10827 | 0.07454 |
| 185.02 | 0.07513 | 0.08736 |  | 0.10167 | 0.10976 | 0.07676 |
| 185.536 | 0.07870 | 0.09084 |  | 0.10491 | 0.11037 | 0.07900 |
| 186.051 | 0.08159 | 0.09361 |  | 0.10752 | 0.11317 | 0.08152 |
| 186.568 | 0.08459 | 0.09596 |  | 0.11023 | 0.11776 | 0.08439 |
| 187.084 | 0.08772 | 0.09799 |  | 0.11255 | 0.12410 | 0.08717 |
| 187.599 | 0.09096 | 0.09964 |  | 0.11470 | 0.12912 | 0.08983 |
| 188.114 | 0.09422 | 0.10156 |  | 0.11744 | 0.13365 | 0.09252 |
| 188.632 | 0.09706 | 0.10533 |  | 0.12043 | 0.13592 | 0.09556 |
| 189.147 | 0.10001 | 0.11095 |  | 0.12363 | 0.13842 | 0.09927 |
| 189.662 | 0.10333 | 0.11502 |  | 0.12742 | 0.14162 | 0.10363 |
| 190.179 | 0.10714 | 0.11912 |  | 0.13150 | 0.14403 | 0.10787 |
| 190.694 | 0.11135 | 0.12218 |  | 0.13640 | 0.14747 | 0.11217 |
| 191.209 | 0.11596 | 0.12561 |  | 0.14174 | 0.15056 | 0.11659 |
| 191.724 | 0.12094 | 0.12494 |  | 0.14734 | 0.15494 | 0.12102 |
| 192.239 | 0.12627 | 0.12691 |  | 0.15202 | 0.16048 | 0.12550 |
| 192.756 | 0.13161 | 0.13003 |  | 0.15477 | 0.16547 | 0.13000 |
| 193.271 | 0.13674 | 0.13474 |  | 0.15873 | 0.16964 | 0.13422 |
| 193.786 | 0.14195 | 0.13903 |  | 0.16257 | 0.17348 | 0.13785 |
| 194.301 | 0.14641 | 0.14306 |  | 0.16666 | 0.17762 | 0.14122 |
| 194.818 | 0.15104 | 0.14738 |  | 0.17106 | 0.18150 | 0.14624 |
| 195.333 | 0.15500 | 0.15218 |  | 0.17585 | 0.18412 | 0.15207 |
| 195.848 | 0.16186 | 0.15728 |  | 0.18099 | 0.18881 | 0.15718 |
| 196.362 | 0.16938 | 0.16274 |  | 0.18647 | 0.19479 | 0.16228 |
| 196.877 | 0.17707 | 0.16843 |  | 0.19230 | 0.20222 | 0.16673 |
| 197.394 | 0.18477 | 0.17487 |  | 0.19848 | 0.20981 | 0.17212 |
| 197.909 | 0.19213 | 0.18221 |  | 0.20500 | 0.21700 | 0.17905 |
| 198.423 | 0.19921 | 0.18932 |  | 0.21188 | 0.22384 | 0.18622 |
| 198.938 | 0.20601 | 0.19608 |  | 0.21878 | 0.23027 | 0.19362 |
| 199.452 | 0.21242 | 0.20122 |  | 0.22359 | 0.23645 | 0.20186 |
| 199.967 | 0.21816 | 0.20641 |  | 0.22893 | 0.24219 | 0.20976 |
| 200.484 | 0.22334 | 0.21087 |  | 0.23347 | 0.24760 | 0.21676 |
| 200.998 | 0.22843 | 0.21340 |  | 0.23732 | 0.25271 | 0.21976 |
| 201.513 | 0.23328 | 0.21781 |  | 0.24141 | 0.25757 | 0.22176 |
| 202.027 | 0.23777 | 0.22191 |  | 0.24633 | 0.26284 | 0.22511 |
| 202.541 | 0.24206 | 0.22890 |  | 0.25238 | 0.26804 | 0.22801 |
| 203.056 | 0.24571 | 0.23445 |  | 0.25804 | 0.27169 | 0.23265 |
| 203.57 | 0.24910 | 0.23959 |  | 0.26320 | 0.27438 | 0.23725 |
| 204.087 | 0.25245 | 0.24386 |  | 0.26657 | 0.27593 | 0.24309 |
| 204.601 | 0.25689 | 0.24683 |  | 0.26875 | 0.27591 | 0.24758 |
| 205.115 | 0.26094 | 0.24802 |  | 0.26650 | 0.27720 | 0.24962 |
| 205.63 | 0.26362 | 0.24727 |  | 0.27060 | 0.27808 | 0.25137 |
| 206.144 | 0.26537 | 0.24593 |  | 0.27246 | 0.27853 | 0.25202 |
| 206.658 | 0.26635 | 0.24468 |  | 0.27332 | 0.27869 | 0.25127 |
| 207.172 | 0.26589 | 0.24301 |  | 0.27348 | 0.27884 | 0.25101 |
| 207.686 | 0.26359 | 0.24149 |  | 0.27297 | 0.27870 | 0.25065 |
| 208.2 | 0.26174 | 0.24014 |  | 0.27170 | 0.28781 | 0.25188 |
| 208.714 | 0.26009 | 0.23913 |  | 0.26937 | 0.28384 | 0.25203 |
| 209.228 | 0.25849 | 0.23866 |  | 0.26628 | 0.28025 | 0.25070 |
| 209.742 | 0.25647 | 0.23731 |  | 0.26242 | 0.28037 | 0.24896 |
| 210.257 | 0.25277 | 0.23535 |  | 0.25777 | 0.28012 | 0.24657 |
| 210.773 | 0.24792 | 0.23220 |  | 0.25242 | 0.27864 | 0.24370 |
| 211.286 | 0.24258 | 0.22776 |  | 0.24644 | 0.27567 | 0.23909 |
| 211.8 | 0.23754 | 0.22252 |  | 0.24155 | 0.27335 | 0.23406 |
| 212.314 | 0.23239 | 0.21753 |  | 0.23719 | 0.26686 | 0.22969 |
| 212.828 | 0.22661 | 0.21357 |  | 0.23383 | 0.25983 | 0.22583 |
| 213.342 | 0.22253 | 0.21012 |  | 0.22895 | 0.25866 | 0.22267 |
| 213.856 | 0.21904 | 0.20572 |  | 0.22426 | 0.25592 | 0.21997 |
| 214.37 | 0.21426 | 0.20029 |  | 0.21917 | 0.25238 | 0.21625 |
| 214.883 | 0.20878 | 0.19419 |  | 0.21350 | 0.24805 | 0.21135 |
| 215.397 | 0.20322 | 0.18884 |  | 0.20810 | 0.24377 | 0.20527 |
| 215.911 | 0.19723 | 0.18391 |  | 0.20349 | 0.23806 | 0.19769 |
| 216.424 | 0.19132 | 0.17951 |  | 0.19922 | 0.23378 | 0.19127 |
| 216.938 | 0.18556 | 0.17532 |  | 0.19508 | 0.23010 | 0.18579 |
| 217.452 | 0.18016 | 0.17134 |  | 0.19076 | 0.22673 | 0.18222 |
| 217.965 | 0.17498 | 0.16725 |  | 0.18617 | 0.22452 | 0.18027 |
| 218.479 | 0.17007 | 0.16251 |  | 0.18086 | 0.22365 | 0.17744 |
| 218.992 | 0.16538 | 0.15698 |  | 0.17560 | 0.22079 | 0.17431 |
| 219.506 | 0.16095 | 0.15149 |  | 0.17023 | 0.21739 | 0.16920 |
| 220.017 | 0.15679 | 0.14632 |  | 0.16448 | 0.21321 | 0.16485 |
| 220.53 | 0.15289 | 0.14178 |  | 0.15910 | 0.20838 | 0.16025 |
| 221.044 | 0.14924 | 0.13753 |  | 0.15504 | 0.20230 | 0.15576 |
| 221.557 | 0.14582 | 0.13363 |  | 0.15159 | 0.19697 | 0.15147 |
| 222.071 | 0.14159 | 0.13003 |  | 0.14819 | 0.19264 | 0.14733 |
| 222.584 | 0.13607 | 0.12682 |  | 0.14665 | 0.18922 | 0.14307 |
| 223.097 | 0.13050 | 0.12392 |  | 0.14529 | 0.18694 | 0.13896 |
| 223.61 | 0.12626 | 0.12130 |  | 0.14296 | 0.18455 | 0.13512 |
| 224.124 | 0.12148 | 0.11880 |  | 0.13833 | 0.18209 | 0.13224 |
| 224.637 | 0.11818 | 0.11592 |  | 0.13320 | 0.17951 | 0.12967 |
| 225.15 | 0.11568 | 0.11301 |  | 0.12817 | 0.17676 | 0.12818 |
| 225.663 | 0.11298 | 0.10988 |  | 0.12569 | 0.17391 | 0.12655 |
| 226.174 | 0.10945 | 0.10689 |  | 0.12338 | 0.17088 | 0.12121 |
| 226.687 | 0.10629 | 0.10374 |  | 0.12172 | 0.16620 | 0.11701 |
| 227.2 | 0.10323 | 0.10093 |  | 0.12008 | 0.16114 | 0.11369 |
| 227.713 | 0.10023 | 0.09888 |  | 0.11848 | 0.15566 | 0.11149 |
| 228.226 | 0.09693 | 0.09721 |  | 0.11709 | 0.15019 | 0.11006 |
| 228.739 | 0.09424 | 0.09484 |  | 0.11534 | 0.14581 | 0.10518 |
| 229.252 | 0.09129 | 0.09213 |  | 0.11418 | 0.14211 | 0.10083 |
| 229.765 | 0.08929 | 0.08949 |  | 0.11164 | 0.13901 | 0.09574 |
| 230.276 | 0.08706 | 0.08724 |  | 0.10806 | 0.13651 | 0.09200 |
| 230.789 | 0.08426 | 0.08509 |  | 0.10499 | 0.13461 | 0.08987 |
| 231.302 | 0.08204 | 0.08310 |  | 0.10243 | 0.13329 | 0.08798 |
| 231.814 | 0.07996 | 0.08129 |  | 0.10045 | 0.13254 | 0.08655 |
| 232.327 | 0.07783 | 0.07969 |  | 0.09903 | 0.13228 | 0.08512 |
| 232.838 | 0.07490 | 0.07825 |  | 0.09834 | 0.13148 | 0.08341 |
| 233.351 | 0.07220 | 0.07692 |  | 0.09806 | 0.12895 | 0.08118 |
| 233.863 | 0.06990 | 0.07560 |  | 0.09811 | 0.12606 | 0.07896 |
| 234.376 | 0.06783 | 0.07431 |  | 0.09757 | 0.12248 | 0.07666 |
| 234.889 | 0.06642 | 0.07259 |  | 0.09584 | 0.11917 | 0.07435 |
| 235.401 | 0.06529 | 0.07078 |  | 0.09439 | 0.11595 | 0.07197 |
| 235.912 | 0.06424 | 0.06887 |  | 0.09278 | 0.11257 | 0.06959 |
| 236.424 | 0.06212 | 0.06714 |  | 0.09080 | 0.10899 | 0.06759 |
| 236.937 | 0.06051 | 0.06548 |  | 0.08871 | 0.10538 | 0.06571 |
| 237.449 | 0.06109 | 0.06393 |  | 0.08632 | 0.10156 | 0.06439 |
| 237.96 | 0.05811 | 0.06247 |  | 0.08383 | 0.09813 | 0.06342 |
| 238.472 | 0.05562 | 0.06107 |  | 0.08134 | 0.09859 | 0.06263 |
| 238.984 | 0.05356 | 0.05980 |  | 0.07893 | 0.09735 | 0.05996 |
| 239.497 | 0.05184 | 0.05862 |  | 0.07840 | 0.09517 | 0.05738 |
| 240.007 | 0.05046 | 0.05754 |  | 0.07779 | 0.09178 | 0.05524 |
| 240.519 | 0.04920 | 0.05653 |  | 0.07817 | 0.08919 | 0.05353 |
| 241.032 | 0.04821 | 0.05548 |  | 0.07853 | 0.08786 | 0.05219 |
| 241.544 | 0.04745 | 0.05449 |  | 0.07874 | 0.08658 | 0.05112 |
| 242.054 | 0.04691 | 0.05366 |  | 0.07888 | 0.08597 | 0.05023 |
| 242.566 | 0.04660 | 0.05302 |  | 0.07876 | 0.08551 | 0.04963 |
| 243.078 | 0.04648 | 0.05262 |  | 0.07837 | 0.08531 | 0.04849 |
| 243.588 | 0.04656 | 0.05253 |  | 0.07777 | 0.08519 | 0.04714 |
| 244.101 | 0.04634 | 0.05252 |  | 0.07693 | 0.08505 | 0.04587 |
| 244.613 | 0.04576 | 0.05230 |  | 0.07600 | 0.08471 | 0.04477 |
| 245.123 | 0.04432 | 0.05084 |  | 0.07503 | 0.08366 | 0.04374 |
| 245.635 | 0.04259 | 0.04955 |  | 0.07402 | 0.08206 | 0.04296 |
| 246.147 | 0.04126 | 0.04855 |  | 0.07309 | 0.07842 | 0.04218 |
| 246.659 | 0.04008 | 0.04774 |  | 0.07222 | 0.07498 | 0.04135 |
| 247.169 | 0.03920 | 0.04702 |  | 0.07147 | 0.07041 | 0.04042 |
| 247.68 | 0.03863 | 0.04638 |  | 0.07081 | 0.07176 | 0.03936 |
| 248.192 | 0.03839 | 0.04572 |  | 0.07032 | 0.07275 | 0.03849 |
| 248.702 | 0.03822 | 0.04559 |  | 0.07014 | 0.07242 | 0.03778 |
| 249.214 | 0.03807 | 0.04573 |  | 0.07021 | 0.07231 | 0.03733 |
| 249.724 | 0.03790 | 0.04595 |  | 0.07062 | 0.07216 | 0.03702 |
| 250.236 | 0.03763 | 0.04601 |  | 0.07133 | 0.07191 | 0.03679 |
| 250.747 | 0.03416 | 0.04588 |  | 0.07261 | 0.07149 | 0.03671 |
| 251.257 | 0.03651 | 0.04559 |  | 0.07400 | 0.07094 | 0.03568 |
| 251.769 | 0.03714 | 0.04515 |  | 0.07398 | 0.07034 | 0.03490 |
| 252.28 | 0.03693 | 0.04455 |  | 0.07365 | 0.06962 | 0.03488 |
| 252.79 | 0.03671 | 0.04380 |  | 0.07288 | 0.06875 | 0.03509 |
| 253.301 | 0.03646 | 0.04297 |  | 0.07224 | 0.06778 | 0.03474 |
| 253.811 | 0.03613 | 0.04239 |  | 0.07183 | 0.06665 | 0.03412 |
| 254.322 | 0.03571 | 0.04261 |  | 0.07155 | 0.06550 | 0.03360 |
| 254.834 | 0.03498 | 0.04348 |  | 0.07138 | 0.06518 | 0.03320 |
| 255.343 | 0.03470 | 0.04509 |  | 0.07133 | 0.06557 | 0.03292 |
| 255.855 | 0.03521 | 0.04542 |  | 0.07140 | 0.06636 | 0.03277 |
| 256.364 | 0.03558 | 0.04408 |  | 0.07161 | 0.06753 | 0.03276 |
| 256.876 | 0.03684 | 0.04192 |  | 0.07193 | 0.06872 | 0.03286 |
| 257.385 | 0.03545 | 0.04036 |  | 0.07238 | 0.06971 | 0.03309 |
| 257.896 | 0.03527 | 0.04059 |  | 0.07295 | 0.07049 | 0.03346 |
| 258.406 | 0.03562 | 0.04151 |  | 0.07364 | 0.07123 | 0.03394 |
| 258.917 | 0.03621 | 0.04287 |  | 0.07447 | 0.07214 | 0.03450 |
| 259.428 | 0.03708 | 0.04409 |  | 0.07550 | 0.07304 | 0.03515 |
| 259.937 | 0.03775 | 0.04540 |  | 0.07701 | 0.07409 | 0.03575 |
| 260.449 | 0.03976 | 0.04668 |  | 0.07967 | 0.07484 | 0.03630 |
| 260.958 | 0.04211 | 0.04801 |  | 0.08341 | 0.07768 | 0.03683 |
| 261.469 | 0.04515 | 0.04926 |  | 0.08664 | 0.08034 | 0.03762 |
| 261.978 | 0.04812 | 0.05061 |  | 0.08987 | 0.08276 | 0.03974 |
| 262.489 | 0.05142 | 0.05205 |  | 0.09294 | 0.08480 | 0.04319 |
| 262.998 | 0.05476 | 0.05378 |  | 0.09593 | 0.08624 | 0.04714 |
| 263.509 | 0.05735 | 0.05587 |  | 0.09917 | 0.08739 | 0.04938 |
| 264.018 | 0.05936 | 0.05733 |  | 0.10239 | 0.08820 | 0.05209 |
| 264.529 | 0.05862 | 0.05736 |  | 0.10565 | 0.08871 | 0.05139 |
| 265.038 | 0.05645 | 0.05694 |  | 0.10870 | 0.08878 | 0.04901 |
| 265.549 | 0.05324 | 0.05574 |  | 0.10894 | 0.08843 | 0.04703 |
| 266.058 | 0.04973 | 0.05371 |  | 0.10574 | 0.08733 | 0.04517 |
| 266.568 | 0.04623 | 0.05114 |  | 0.09906 | 0.08513 | 0.04326 |
| 267.077 | 0.04200 | 0.04867 |  | 0.09197 | 0.08241 | 0.04100 |
| 267.586 | 0.03764 | 0.04602 |  | 0.08466 | 0.07925 | 0.03862 |
| 268.097 | 0.03399 | 0.04334 |  | 0.07699 | 0.07539 | 0.03555 |
| 268.605 | 0.03089 | 0.04108 |  | 0.07639 | 0.07233 | 0.03319 |
| 269.116 | 0.02883 | 0.03915 |  | 0.07260 | 0.06853 | 0.03106 |
| 269.625 | 0.02750 | 0.03758 |  | 0.06932 | 0.06579 | 0.02938 |
| 270.135 | 0.02675 | 0.03657 |  | 0.06694 | 0.06351 | 0.02775 |
| 270.644 | 0.02601 | 0.03586 |  | 0.06534 | 0.06226 | 0.02617 |
| 271.153 | 0.02488 | 0.03545 |  | 0.06456 | 0.06210 | 0.02470 |
| 271.663 | 0.02368 | 0.03497 |  | 0.06479 | 0.06191 | 0.02340 |
| 272.172 | 0.02258 | 0.03458 |  | 0.06516 | 0.06110 | 0.02227 |
| 272.682 | 0.02165 | 0.03413 |  | 0.06610 | 0.06093 | 0.02143 |
| 273.191 | 0.02081 | 0.03371 |  | 0.06374 | 0.05957 | 0.02101 |
| 273.699 | 0.02014 | 0.03331 |  | 0.06179 | 0.05883 | 0.02105 |
| 274.209 | 0.01961 | 0.03291 |  | 0.05995 | 0.05792 | 0.02100 |
| 274.718 | 0.01953 | 0.03252 |  | 0.05859 | 0.05746 | 0.02076 |
| 275.228 | 0.01945 | 0.03214 |  | 0.05803 | 0.05750 | 0.02012 |
| 275.736 | 0.01951 | 0.03178 |  | 0.05837 | 0.05779 | 0.01992 |
| 276.245 | 0.01950 | 0.03144 |  | 0.05844 | 0.05803 | 0.01980 |
| 276.755 | 0.01934 | 0.03114 |  | 0.05863 | 0.05832 | 0.01969 |
| 277.263 | 0.01890 | 0.03089 |  | 0.05891 | 0.05856 | 0.01958 |
| 277.771 | 0.01833 | 0.03090 |  | 0.05915 | 0.05886 | 0.01947 |
| 278.282 | 0.01785 | 0.03112 |  | 0.05923 | 0.05807 | 0.01933 |
| 278.79 | 0.01746 | 0.03119 |  | 0.05922 | 0.05761 | 0.01791 |
| 279.298 | 0.01724 | 0.03063 |  | 0.05920 | 0.05710 | 0.01688 |
| 279.808 | 0.01705 | 0.02976 |  | 0.05888 | 0.05643 | 0.01620 |
| 280.316 | 0.01693 | 0.02920 |  | 0.05845 | 0.05568 | 0.01591 |
| 280.824 | 0.01666 | 0.02862 |  | 0.05792 | 0.05489 | 0.01619 |
| 281.334 | 0.01589 | 0.02812 |  | 0.05732 | 0.05444 | 0.01614 |
| 281.842 | 0.01637 | 0.02759 |  | 0.05666 | 0.05440 | 0.01621 |
| 282.35 | 0.01675 | 0.02728 |  | 0.05602 | 0.05466 | 0.01635 |
| 282.858 | 0.01605 | 0.02702 |  | 0.05559 | 0.05498 | 0.01650 |
| 283.368 | 0.01574 | 0.02704 |  | 0.05528 | 0.05476 | 0.01672 |
| 283.876 | 0.01527 | 0.02740 |  | 0.05508 | 0.05439 | 0.01631 |
| 284.384 | 0.01540 | 0.02775 |  | 0.05509 | 0.05371 | 0.01392 |
| 284.893 | 0.01546 | 0.02810 |  | 0.05551 | 0.05319 | 0.01378 |
| 285.401 | 0.01566 | 0.02860 |  | 0.05601 | 0.05274 | 0.01359 |
| 285.909 | 0.01584 | 0.02904 |  | 0.05664 | 0.05237 | 0.01520 |
| 286.417 | 0.01606 | 0.02904 |  | 0.05742 | 0.05207 | 0.01508 |
| 286.926 | 0.01612 | 0.02894 |  | 0.05836 | 0.05180 | 0.01464 |
| 287.434 | 0.01588 | 0.02826 |  | 0.05913 | 0.05154 | 0.01399 |
| 287.942 | 0.01526 | 0.02799 |  | 0.05959 | 0.05161 | 0.01341 |
| 288.449 | 0.01436 | 0.02830 |  | 0.05898 | 0.05172 | 0.01292 |
| 288.957 | 0.01299 | 0.02863 |  | 0.05791 | 0.05171 | 0.01252 |
| 289.466 | 0.01500 | 0.02873 |  | 0.05672 | 0.05154 | 0.01223 |
| 289.974 | 0.01544 | 0.02867 |  | 0.05538 | 0.05066 | 0.01294 |
| 290.481 | 0.01482 | 0.02850 |  | 0.05442 | 0.04997 | 0.01372 |
| 290.989 | 0.01431 | 0.02821 |  | 0.05401 | 0.05005 | 0.01447 |
| 291.498 | 0.01394 | 0.02773 |  | 0.05393 | 0.04998 | 0.01526 |
| 292.006 | 0.01242 | 0.02713 |  | 0.05395 | 0.04969 | 0.01512 |
| 292.513 | 0.01339 | 0.02640 |  | 0.05440 | 0.04903 | 0.01483 |
| 293.02 | 0.01280 | 0.02569 |  | 0.05503 | 0.04837 | 0.01240 |
| 293.528 | 0.01231 | 0.02640 |  | 0.05587 | 0.04804 | 0.01516 |
| 294.035 | 0.01198 | 0.02694 |  | 0.05637 | 0.04784 | 0.01445 |
| 294.544 | 0.01178 | 0.02738 |  | 0.05626 | 0.04772 | 0.01358 |
| 295.052 | 0.01172 | 0.02775 |  | 0.05634 | 0.04780 | 0.01233 |
| 295.559 | 0.01172 | 0.02814 |  | 0.05532 | 0.04819 | 0.01137 |
| 296.066 | 0.01225 | 0.02844 |  | 0.05477 | 0.04898 | 0.01082 |
| 296.573 | 0.01274 | 0.02796 |  | 0.05501 | 0.05067 | 0.01068 |
| 297.08 | 0.01332 | 0.02794 |  | 0.05506 | 0.05265 | 0.01093 |
| 297.587 | 0.01341 | 0.02816 |  | 0.05518 | 0.05433 | 0.01159 |
| 298.096 | 0.01269 | 0.02861 |  | 0.05581 | 0.05552 | 0.01264 |
| 298.603 | 0.01230 | 0.02933 |  | 0.05584 | 0.05619 | 0.01336 |
| 299.11 | 0.01199 | 0.02980 |  | 0.05541 | 0.05620 | 0.01357 |
| 299.617 | 0.01204 | 0.02996 |  | 0.05490 | 0.05480 | 0.01310 |
| 300.124 | 0.01356 | 0.02978 |  | 0.05443 | 0.05260 | 0.01314 |
| 300.631 | 0.01367 | 0.02924 |  | 0.05420 | 0.05205 | 0.01308 |
| 301.138 | 0.01359 | 0.02865 |  | 0.05401 | 0.05234 | 0.01296 |
| 301.645 | 0.01328 | 0.02813 |  | 0.05395 | 0.05425 | 0.01285 |
| 302.152 | 0.01309 | 0.02777 |  | 0.05398 | 0.05532 | 0.01277 |
| 302.659 | 0.01279 | 0.02761 |  | 0.05413 | 0.05588 | 0.01271 |
| 303.165 | 0.01274 | 0.02750 |  | 0.05437 | 0.05591 | 0.01268 |
| 303.674 | 0.01270 | 0.02767 |  | 0.05472 | 0.05533 | 0.01267 |
| 304.181 | 0.01265 | 0.02805 |  | 0.05519 | 0.05403 | 0.01270 |
| 304.688 | 0.01258 | 0.02850 |  | 0.05571 | 0.05213 | 0.01277 |
| 305.194 | 0.01247 | 0.02966 |  | 0.05628 | 0.05039 | 0.01291 |
| 305.701 | 0.01229 | 0.02896 |  | 0.05664 | 0.04891 | 0.01320 |
| 306.208 | 0.01198 | 0.02787 |  | 0.05651 | 0.04956 | 0.01337 |
| 306.714 | 0.01155 | 0.02728 |  | 0.05595 | 0.05038 | 0.01261 |
| 307.221 | 0.01124 | 0.02708 |  | 0.05497 | 0.05067 | 0.01180 |
| 307.727 | 0.01107 | 0.02728 |  | 0.05409 | 0.05099 | 0.01104 |
| 308.234 | 0.01163 | 0.02677 |  | 0.05326 | 0.04939 | 0.01055 |
| 308.74 | 0.01182 | 0.02661 |  | 0.05238 | 0.04745 | 0.01013 |
| 309.247 | 0.01225 | 0.02611 |  | 0.05256 | 0.04326 | 0.00999 |
| 309.753 | 0.01210 | 0.02574 |  | 0.05290 | 0.04169 | 0.00992 |
| 310.26 | 0.01212 | 0.02600 |  | 0.05352 | 0.04323 | 0.00991 |
| 310.766 | 0.01288 | 0.02657 |  | 0.05421 | 0.05185 | 0.00997 |
| 311.272 | 0.01304 | 0.02717 |  | 0.05498 | 0.06135 | 0.01006 |
| 311.779 | 0.01323 | 0.02746 |  | 0.05568 | 0.05183 | 0.01018 |
| 312.285 | 0.01343 | 0.02777 |  | 0.05622 | 0.05078 | 0.01035 |
| 312.791 | 0.01363 | 0.02765 |  | 0.05571 | 0.05258 | 0.01059 |
| 313.297 | 0.01380 | 0.02743 |  | 0.05499 | 0.05161 | 0.01090 |
| 313.804 | 0.01396 | 0.02718 |  | 0.05336 | 0.05088 | 0.01143 |
| 314.31 | 0.01411 | 0.02695 |  | 0.05288 | 0.05007 | 0.01218 |
| 314.816 | 0.01423 | 0.02671 |  | 0.05244 | 0.04931 | 0.01268 |
| 315.322 | 0.01431 | 0.02677 |  | 0.05270 | 0.04902 | 0.01266 |
| 315.826 | 0.01445 | 0.02704 |  | 0.05304 | 0.04977 | 0.01254 |
| 316.332 | 0.01460 | 0.02748 |  | 0.05354 | 0.05138 | 0.01236 |
| 316.838 | 0.01473 | 0.02793 |  | 0.05416 | 0.05410 | 0.01235 |
| 317.344 | 0.01478 | 0.02838 |  | 0.05468 | 0.05685 | 0.01291 |
| 317.85 | 0.01471 | 0.02884 |  | 0.05442 | 0.05813 | 0.01336 |
| 318.356 | 0.01455 | 0.02914 |  | 0.05457 | 0.05773 | 0.01389 |
| 318.862 | 0.01414 | 0.02937 |  | 0.05463 | 0.05645 | 0.01452 |
| 319.368 | 0.01375 | 0.02951 |  | 0.05562 | 0.05593 | 0.01458 |
| 319.874 | 0.01334 | 0.02956 |  | 0.05825 | 0.05536 | 0.01384 |
| 320.38 | 0.01298 | 0.02958 |  | 0.05871 | 0.05481 | 0.01262 |
| 320.883 | 0.01272 | 0.02891 |  | 0.05888 | 0.05440 | 0.01152 |
| 321.389 | 0.01266 | 0.02802 |  | 0.05707 | 0.05409 | 0.01096 |
| 321.895 | 0.01274 | 0.02736 |  | 0.05566 | 0.05397 | 0.01141 |
| 322.401 | 0.01295 | 0.02698 |  | 0.05477 | 0.05393 | 0.01235 |
| 322.906 | 0.01325 | 0.02683 |  | 0.05484 | 0.05394 | 0.01293 |
| 323.412 | 0.01372 | 0.02671 |  | 0.05476 | 0.05377 | 0.01362 |
| 323.918 | 0.01372 | 0.02686 |  | 0.05465 | 0.05279 | 0.01420 |
| 324.421 | 0.01281 | 0.02723 |  | 0.05463 | 0.05209 | 0.01507 |
| 324.927 | 0.01209 | 0.02756 |  | 0.05495 | 0.05156 | 0.01522 |
| 325.432 | 0.01159 | 0.02780 |  | 0.05530 | 0.05122 | 0.01446 |
| 325.938 | 0.01085 | 0.02770 |  | 0.05568 | 0.05103 | 0.01349 |
| 326.443 | 0.01034 | 0.02763 |  | 0.05610 | 0.05104 | 0.01315 |
| 326.949 | 0.01036 | 0.02751 |  | 0.05655 | 0.05122 | 0.01329 |
| 327.452 | 0.01065 | 0.02743 |  | 0.05691 | 0.05152 | 0.01338 |
| 327.958 | 0.01108 | 0.02747 |  | 0.05605 | 0.05185 | 0.01345 |
| 328.463 | 0.01176 | 0.02763 |  | 0.05666 | 0.05217 | 0.01305 |
| 328.968 | 0.01229 | 0.02827 |  | 0.05762 | 0.05165 | 0.01271 |
| 329.474 | 0.01288 | 0.02855 |  | 0.05829 | 0.05016 | 0.01223 |
| 329.977 | 0.01038 | 0.02885 |  | 0.05910 | 0.04948 | 0.01158 |
| 330.482 | 0.01152 | 0.02866 |  | 0.05940 | 0.04953 | 0.01078 |
| 330.987 | 0.01181 | 0.02859 |  | 0.05894 | 0.05060 | 0.00982 |
| 331.493 | 0.01202 | 0.02857 |  | 0.05805 | 0.05215 | 0.00889 |
| 331.996 | 0.01223 | 0.02859 |  | 0.05708 | 0.05360 | 0.00954 |
| 332.501 | 0.01242 | 0.02867 |  | 0.05659 | 0.05505 | 0.01093 |
| 333.006 | 0.01253 | 0.02874 |  | 0.05579 | 0.05561 | 0.01216 |
| 333.511 | 0.01256 | 0.02881 |  | 0.05565 | 0.05566 | 0.01282 |
| 334.014 | 0.01251 | 0.02889 |  | 0.05590 | 0.05558 | 0.01304 |
| 334.519 | 0.01241 | 0.02898 |  | 0.05690 | 0.05539 | 0.01305 |
| 335.024 | 0.01226 | 0.02908 |  | 0.05757 | 0.05524 | 0.01299 |
| 335.529 | 0.01207 | 0.02919 |  | 0.05790 | 0.05540 | 0.01300 |
| 336.032 | 0.01179 | 0.02930 |  | 0.05792 | 0.05565 | 0.01313 |
| 336.537 | 0.01171 | 0.02946 |  | 0.05798 | 0.05596 | 0.01324 |
| 337.042 | 0.01219 | 0.02978 |  | 0.05773 | 0.05629 | 0.01339 |
| 337.545 | 0.01285 | 0.03021 |  | 0.05738 | 0.05662 | 0.01357 |
| 338.05 | 0.01340 | 0.03081 |  | 0.05737 | 0.05692 | 0.01379 |
| 338.555 | 0.01370 | 0.03151 |  | 0.05787 | 0.05585 | 0.01404 |
| 339.057 | 0.01343 | 0.03145 |  | 0.05827 | 0.05524 | 0.01433 |
| 339.562 | 0.01325 | 0.03132 |  | 0.05849 | 0.05500 | 0.01457 |
| 340.067 | 0.01316 | 0.03096 |  | 0.05836 | 0.05504 | 0.01430 |
| 340.57 | 0.01306 | 0.03034 |  | 0.05820 | 0.05530 | 0.01339 |
| 341.074 | 0.01281 | 0.02940 |  | 0.05796 | 0.05617 | 0.01300 |
| 341.579 | 0.01247 | 0.02934 |  | 0.05771 | 0.05693 | 0.01349 |
| 342.082 | 0.01328 | 0.02911 |  | 0.05724 | 0.05781 | 0.01449 |
| 342.586 | 0.01331 | 0.02963 |  | 0.05669 | 0.05867 | 0.01517 |
| 343.091 | 0.01343 | 0.03073 |  | 0.05623 | 0.05946 | 0.01584 |
| 343.593 | 0.01368 | 0.03157 |  | 0.05659 | 0.06015 | 0.01644 |
| 344.098 | 0.01363 | 0.03253 |  | 0.05801 | 0.06066 | 0.01671 |
| 344.602 | 0.01374 | 0.03318 |  | 0.05771 | 0.06105 | 0.01714 |
| 345.105 | 0.01397 | 0.03349 |  | 0.05746 | 0.06117 | 0.01731 |
| 345.609 | 0.01435 | 0.03359 |  | 0.05747 | 0.06114 | 0.01737 |
| 346.112 | 0.01487 | 0.03381 |  | 0.05757 | 0.06118 | 0.01735 |
| 346.616 | 0.01537 | 0.03412 |  | 0.05781 | 0.06135 | 0.01734 |
| 347.12 | 0.01597 | 0.03450 |  | 0.05837 | 0.06176 | 0.01750 |
| 347.623 | 0.01594 | 0.03503 |  | 0.05933 | 0.06240 | 0.01825 |
| 348.127 | 0.01595 | 0.03602 |  | 0.06101 | 0.06319 | 0.01956 |
| 348.629 | 0.01621 | 0.03693 |  | 0.06107 | 0.06411 | 0.02139 |
| 349.133 | 0.01638 | 0.03818 |  | 0.06251 | 0.06509 | 0.02296 |
| 349.636 | 0.01706 | 0.03966 |  | 0.06379 | 0.06617 | 0.02487 |
| 350.14 | 0.01862 | 0.04153 |  | 0.06502 | 0.06725 | 0.02605 |
| 350.644 | 0.02114 | 0.04382 |  | 0.06701 | 0.06844 | 0.02733 |
| 351.146 | 0.02459 | 0.04660 |  | 0.06919 | 0.07000 | 0.02972 |
| 351.65 | 0.02898 | 0.04991 |  | 0.07272 | 0.07223 | 0.03310 |
| 352.152 | 0.03321 | 0.05374 |  | 0.07723 | 0.07621 | 0.03750 |
| 352.656 | 0.03875 | 0.05794 |  | 0.08267 | 0.08195 | 0.04282 |
| 353.158 | 0.04769 | 0.06288 |  | 0.08923 | 0.08752 | 0.04913 |
| 353.662 | 0.05577 | 0.07539 |  | 0.09700 | 0.09389 | 0.05647 |
| 354.164 | 0.06476 | 0.08817 |  | 0.10577 | 0.10035 | 0.06486 |
| 354.668 | 0.07202 | 0.09667 |  | 0.11559 | 0.10566 | 0.07472 |
| 355.17 | 0.07668 | 0.10170 |  | 0.12637 | 0.11060 | 0.08327 |
| 355.674 | 0.07274 | 0.10314 |  | 0.13819 | 0.11251 | 0.08356 |
| 356.176 | 0.06434 | 0.09782 |  | 0.14273 | 0.11081 | 0.07471 |
| 356.68 | 0.05507 | 0.09019 |  | 0.11280 | 0.10627 | 0.06385 |
| 357.181 | 0.05147 | 0.08045 |  | 0.09987 | 0.09946 | 0.05257 |
| 357.685 | 0.04674 | 0.06844 |  | 0.08672 | 0.09044 | 0.04503 |
| 358.187 | 0.04243 | 0.05858 |  | 0.08366 | 0.08188 | 0.03894 |
| 358.69 | 0.03880 | 0.05237 |  | 0.07917 | 0.07525 | 0.03438 |
| 359.192 | 0.03623 | 0.04732 |  | 0.07559 | 0.07110 | 0.03104 |
| 359.696 | 0.03422 | 0.04670 |  | 0.07184 | 0.06805 | 0.02773 |
| 360.197 | 0.03268 | 0.04475 |  | 0.06909 | 0.06655 | 0.02566 |
| 360.699 | 0.03141 | 0.04326 |  | 0.06710 | 0.06685 | 0.02489 |
| 361.203 | 0.02972 | 0.03953 |  | 0.06557 | 0.06824 | 0.02403 |
| 361.704 | 0.02814 | 0.03717 |  | 0.06462 | 0.06887 | 0.02315 |
| 362.208 | 0.02664 | 0.03521 |  | 0.06415 | 0.06856 | 0.02261 |
| 362.709 | 0.02529 | 0.03368 |  | 0.06404 | 0.06757 | 0.02184 |
| 363.213 | 0.02402 | 0.03253 |  | 0.06415 | 0.06633 | 0.02102 |
| 363.714 | 0.02286 | 0.03173 |  | 0.06269 | 0.06547 | 0.01992 |
| 364.215 | 0.02158 | 0.03132 |  | 0.06140 | 0.06447 | 0.01855 |
| 364.719 | 0.02029 | 0.03123 |  | 0.05906 | 0.06327 | 0.01762 |
| 365.22 | 0.01925 | 0.03134 |  | 0.05942 | 0.06213 | 0.01742 |
| 365.723 | 0.01841 | 0.03145 |  | 0.05897 | 0.06094 | 0.01770 |
| 366.225 | 0.01801 | 0.03163 |  | 0.05916 | 0.05979 | 0.01745 |
| 366.726 | 0.01806 | 0.03191 |  | 0.05902 | 0.05875 | 0.01721 |
| 367.229 | 0.01892 | 0.03235 |  | 0.05927 | 0.05806 | 0.01705 |
| 367.73 | 0.01968 | 0.03186 |  | 0.05995 | 0.05768 | 0.01647 |
| 368.231 | 0.02045 | 0.03136 |  | 0.06098 | 0.05741 | 0.01573 |
| 368.735 | 0.02085 | 0.03087 |  | 0.06232 | 0.05731 | 0.01565 |
| 369.236 | 0.02026 | 0.03040 |  | 0.06390 | 0.05736 | 0.01589 |
| 369.737 | 0.01945 | 0.03008 |  | 0.06185 | 0.05762 | 0.01639 |
| 370.24 | 0.01850 | 0.02997 |  | 0.06087 | 0.05600 | 0.01704 |
| 370.741 | 0.01741 | 0.03019 |  | 0.05998 | 0.05497 | 0.01719 |
| 371.242 | 0.01621 | 0.03043 |  | 0.05869 | 0.05462 | 0.01725 |
| 371.745 | 0.01512 | 0.03068 |  | 0.05806 | 0.05495 | 0.01680 |
| 372.246 | 0.01436 | 0.03081 |  | 0.05758 | 0.05689 | 0.01650 |
| 372.747 | 0.01526 | 0.03066 |  | 0.05747 | 0.06648 | 0.01646 |
| 373.25 | 0.01605 | 0.03044 |  | 0.05763 | 0.05439 | 0.01553 |
| 373.751 | 0.01714 | 0.03010 |  | 0.05790 | 0.05816 | 0.01424 |
| 374.251 | 0.01797 | 0.02964 |  | 0.05822 | 0.05766 | 0.01356 |
| 374.752 | 0.01847 | 0.02907 |  | 0.05804 | 0.06103 | 0.01342 |
| 375.255 | 0.01864 | 0.02836 |  | 0.05786 | 0.06347 | 0.01385 |
| 375.756 | 0.01854 | 0.02900 |  | 0.05774 | 0.06510 | 0.01485 |
| 376.257 | 0.01824 | 0.03010 |  | 0.05752 | 0.06589 | 0.01585 |
| 376.759 | 0.01769 | 0.03239 |  | 0.05779 | 0.06558 | 0.01684 |
| 377.26 | 0.01700 | 0.03231 |  | 0.05837 | 0.06394 | 0.01725 |
| 377.761 | 0.01635 | 0.03183 |  | 0.05933 | 0.06070 | 0.01674 |
| 378.261 | 0.01575 | 0.03058 |  | 0.06083 | 0.05793 | 0.01626 |
| 378.764 | 0.01529 | 0.03123 |  | 0.06188 | 0.05638 | 0.01554 |
| 379.264 | 0.01497 | 0.03230 |  | 0.06028 | 0.05720 | 0.01464 |
| 379.765 | 0.01470 | 0.03320 |  | 0.06000 | 0.06031 | 0.01390 |
| 380.265 | 0.01451 | 0.03345 |  | 0.06040 | 0.06620 | 0.01282 |
| 380.766 | 0.01438 | 0.03189 |  | 0.06085 | 0.06724 | 0.01407 |
| 381.268 | 0.01432 | 0.03061 |  | 0.06132 | 0.06754 | 0.01454 |
| 381.769 | 0.01445 | 0.02997 |  | 0.06176 | 0.06608 | 0.01540 |
| 382.269 | 0.01476 | 0.02932 |  | 0.06214 | 0.06333 | 0.01574 |
| 382.769 | 0.01521 | 0.03175 |  | 0.06246 | 0.06217 | 0.01627 |
| 383.27 | 0.01560 | 0.03209 |  | 0.06276 | 0.06136 | 0.01638 |
| 383.772 | 0.01588 | 0.03223 |  | 0.06253 | 0.06092 | 0.01549 |
| 384.272 | 0.01615 | 0.03236 |  | 0.06084 | 0.06052 | 0.01444 |
| 384.773 | 0.01638 | 0.03244 |  | 0.05841 | 0.06009 | 0.01410 |
| 385.273 | 0.01654 | 0.03247 |  | 0.05871 | 0.05972 | 0.01413 |
| 385.773 | 0.01658 | 0.03240 |  | 0.05926 | 0.05857 | 0.01446 |
| 386.275 | 0.01650 | 0.03241 |  | 0.05997 | 0.05718 | 0.01465 |
| 386.776 | 0.01613 | 0.03256 |  | 0.06088 | 0.05619 | 0.01476 |
| 387.276 | 0.01565 | 0.03288 |  | 0.06197 | 0.05555 | 0.01491 |
| 387.776 | 0.01500 | 0.03338 |  | 0.06326 | 0.05643 | 0.01549 |
| 388.276 | 0.01421 | 0.03405 |  | 0.06391 | 0.05771 | 0.01626 |
| 388.776 | 0.01326 | 0.03492 |  | 0.06406 | 0.05953 | 0.01723 |
| 389.276 | 0.01296 | 0.03594 |  | 0.06189 | 0.06123 | 0.01835 |
| 389.776 | 0.01378 | 0.03884 |  | 0.06100 | 0.06214 | 0.01966 |
| 390.278 | 0.01486 | 0.04026 |  | 0.06163 | 0.06213 | 0.02108 |
| 390.778 | 0.01625 | 0.04005 |  | 0.06314 | 0.06174 | 0.02278 |
| 391.278 | 0.01781 | 0.04032 |  | 0.06477 | 0.06260 | 0.02462 |
| 391.777 | 0.01889 | 0.04211 |  | 0.06756 | 0.06550 | 0.02677 |
| 392.277 | 0.01942 | 0.04568 |  | 0.07091 | 0.07030 | 0.03079 |
| 392.777 | 0.01796 | 0.05042 |  | 0.07467 | 0.07549 | 0.03910 |
| 393.277 | 0.01777 | 0.05545 |  | 0.07851 | 0.08080 | 0.04635 |
| 393.777 | 0.01776 | 0.05951 |  | 0.07998 | 0.08651 | 0.05065 |
| 394.276 | 0.01840 | 0.06053 |  | 0.07967 | 0.08997 | 0.05378 |
| 394.776 | 0.01877 | 0.06077 |  | 0.07738 | 0.08867 | 0.04663 |
| 395.276 | 0.01896 | 0.06015 |  | 0.07488 | 0.08664 | 0.04234 |
| 395.775 | 0.01902 | 0.05870 |  | 0.07202 | 0.08426 | 0.03897 |
| 396.275 | 0.01897 | 0.05640 |  | 0.06939 | 0.08203 | 0.03729 |
| 396.777 | 0.01873 | 0.05331 |  | 0.06812 | 0.08022 | 0.03511 |
| 397.276 | 0.01833 | 0.05082 |  | 0.06822 | 0.07883 | 0.03335 |
| 397.776 | 0.01743 | 0.04907 |  | 0.06946 | 0.07778 | 0.03186 |
| 398.275 | 0.01716 | 0.04777 |  | 0.07163 | 0.07707 | 0.03077 |
| 398.775 | 0.01791 | 0.04742 |  | 0.07481 | 0.07677 | 0.03014 |
| 399.274 | 0.01898 | 0.04877 |  | 0.07595 | 0.07688 | 0.02955 |
| 399.774 | 0.02027 | 0.05111 |  | 0.07680 | 0.07744 | 0.02952 |
| 400.273 | 0.02088 | 0.05409 |  | 0.07787 | 0.07887 | 0.02995 |
| 400.773 | 0.02158 | 0.05700 |  | 0.07930 | 0.08055 | 0.03084 |
| 401.272 | 0.02225 | 0.06009 |  | 0.08335 | 0.08123 | 0.03056 |
| 401.771 | 0.02281 | 0.06276 |  | 0.08797 | 0.08148 | 0.02967 |
| 402.271 | 0.02318 | 0.06426 |  | 0.09381 | 0.08121 | 0.02848 |
| 402.77 | 0.02330 | 0.06364 |  | 0.09686 | 0.08043 | 0.02679 |
| 403.269 | 0.02293 | 0.06023 |  | 0.09938 | 0.07908 | 0.02577 |
| 403.766 | 0.02204 | 0.05567 |  | 0.09909 | 0.07726 | 0.02492 |
| 404.265 | 0.02084 | 0.05203 |  | 0.09370 | 0.07496 | 0.02376 |
| 404.765 | 0.01988 | 0.04830 |  | 0.08660 | 0.07227 | 0.02229 |
| 405.264 | 0.01911 | 0.04468 |  | 0.07850 | 0.06919 | 0.02048 |
| 405.763 | 0.01849 | 0.04152 |  | 0.07227 | 0.06629 | 0.01861 |
| 406.262 | 0.01802 | 0.03905 |  | 0.06835 | 0.06363 | 0.01721 |
| 406.761 | 0.01770 | 0.03744 |  | 0.06947 | 0.06057 | 0.01556 |
| 407.26 | 0.01757 | 0.03663 |  | 0.06862 | 0.05916 | 0.01438 |
| 407.759 | 0.01760 | 0.03629 |  | 0.06655 | 0.05865 | 0.01458 |
| 408.258 | 0.01779 | 0.03614 |  | 0.06508 | 0.05981 | 0.01533 |
| 408.757 | 0.01792 | 0.03608 |  | 0.06391 | 0.06131 | 0.01558 |
| 409.256 | 0.01687 | 0.03627 |  | 0.06296 | 0.06281 | 0.01566 |
| 409.755 | 0.01629 | 0.03499 |  | 0.06220 | 0.06362 | 0.01546 |
| 410.251 | 0.01598 | 0.03258 |  | 0.06099 | 0.06146 | 0.01520 |
| 410.75 | 0.01596 | 0.03108 |  | 0.06067 | 0.06026 | 0.01492 |
| 411.249 | 0.01611 | 0.02998 |  | 0.06141 | 0.05919 | 0.01489 |
| 411.748 | 0.01657 | 0.03046 |  | 0.06226 | 0.05815 | 0.01482 |
| 412.247 | 0.01719 | 0.03115 |  | 0.06249 | 0.05731 | 0.01516 |
| 412.745 | 0.01793 | 0.03200 |  | 0.06215 | 0.05637 | 0.01533 |
| 413.244 | 0.01822 | 0.03266 |  | 0.06172 | 0.05647 | 0.01545 |
| 413.743 | 0.01853 | 0.03322 |  | 0.06082 | 0.05752 | 0.01560 |
| 414.239 | 0.01892 | 0.03382 |  | 0.06027 | 0.05960 | 0.01578 |
| 414.738 | 0.01871 | 0.03422 |  | 0.05980 | 0.06228 | 0.01597 |
| 415.237 | 0.01815 | 0.03456 |  | 0.05944 | 0.06327 | 0.01616 |
| 415.735 | 0.01733 | 0.03474 |  | 0.05930 | 0.06287 | 0.01634 |
| 416.234 | 0.01629 | 0.03464 |  | 0.05934 | 0.06184 | 0.01655 |
| 416.73 | 0.01567 | 0.03408 |  | 0.05932 | 0.06125 | 0.01675 |
| 417.229 | 0.01601 | 0.03313 |  | 0.05942 | 0.05957 | 0.01696 |
| 417.727 | 0.01793 | 0.03281 |  | 0.05922 | 0.05772 | 0.01708 |
| 418.225 | 0.01816 | 0.03293 |  | 0.05941 | 0.05597 | 0.01671 |
| 418.724 | 0.01822 | 0.03363 |  | 0.06042 | 0.05513 | 0.01639 |
| 419.22 | 0.01796 | 0.03414 |  | 0.06176 | 0.05469 | 0.01617 |
| 419.719 | 0.01770 | 0.03473 |  | 0.06329 | 0.05496 | 0.01620 |
| 420.217 | 0.01751 | 0.03509 |  | 0.06479 | 0.05573 | 0.01635 |
| 420.715 | 0.01745 | 0.03507 |  | 0.06504 | 0.05708 | 0.01667 |
| 421.213 | 0.01753 | 0.03489 |  | 0.06493 | 0.05895 | 0.01685 |
| 421.71 | 0.01772 | 0.03471 |  | 0.06462 | 0.06142 | 0.01723 |
| 422.208 | 0.01801 | 0.03459 |  | 0.06438 | 0.06427 | 0.01788 |
| 422.706 | 0.01839 | 0.03457 |  | 0.06424 | 0.06390 | 0.01717 |
| 423.204 | 0.01807 | 0.03456 |  | 0.06406 | 0.06417 | 0.01701 |
| 423.7 | 0.01823 | 0.03458 |  | 0.06451 | 0.06406 | 0.01732 |
| 424.198 | 0.01879 | 0.03465 |  | 0.06545 | 0.06305 | 0.01777 |
| 424.696 | 0.01925 | 0.03475 |  | 0.06639 | 0.06277 | 0.01828 |
| 425.192 | 0.01980 | 0.03497 |  | 0.06699 | 0.06259 | 0.01883 |
| 425.69 | 0.02030 | 0.03581 |  | 0.06694 | 0.06253 | 0.01945 |
| 426.189 | 0.02062 | 0.03559 |  | 0.06670 | 0.06257 | 0.02007 |
| 426.686 | 0.02084 | 0.03538 |  | 0.06732 | 0.06275 | 0.02051 |
| 427.182 | 0.02094 | 0.03507 |  | 0.06785 | 0.06306 | 0.02077 |
| 427.68 | 0.02107 | 0.03517 |  | 0.06815 | 0.06352 | 0.02091 |
| 428.178 | 0.02128 | 0.03544 |  | 0.06819 | 0.06414 | 0.02073 |
| 428.674 | 0.02159 | 0.03609 |  | 0.06782 | 0.06493 | 0.02032 |
| 429.172 | 0.02224 | 0.03730 |  | 0.06686 | 0.06589 | 0.01990 |
| 429.67 | 0.02281 | 0.03758 |  | 0.06592 | 0.06680 | 0.01926 |
| 430.165 | 0.02353 | 0.03778 |  | 0.06548 | 0.06672 | 0.01892 |
| 430.663 | 0.02364 | 0.03781 |  | 0.06537 | 0.06605 | 0.01983 |
| 431.161 | 0.02359 | 0.03774 |  | 0.06541 | 0.06571 | 0.02072 |
| 431.656 | 0.02144 | 0.03761 |  | 0.06562 | 0.06574 | 0.02165 |
| 432.154 | 0.02496 | 0.03748 |  | 0.06592 | 0.06603 | 0.02265 |
| 432.652 | 0.02510 | 0.03733 |  | 0.06636 | 0.06645 | 0.02281 |
| 433.147 | 0.02529 | 0.03720 |  | 0.06687 | 0.06702 | 0.02307 |
| 433.645 | 0.02551 | 0.03714 |  | 0.06721 | 0.06776 | 0.02328 |
| 434.141 | 0.02575 | 0.03744 |  | 0.06761 | 0.06855 | 0.02348 |
| 434.638 | 0.02604 | 0.03817 |  | 0.06775 | 0.06922 | 0.02373 |
| 435.136 | 0.02637 | 0.03926 |  | 0.06767 | 0.06923 | 0.02402 |
| 435.631 | 0.02674 | 0.04041 |  | 0.06676 | 0.06756 | 0.02440 |
| 436.128 | 0.02712 | 0.04140 |  | 0.06628 | 0.06585 | 0.02488 |
| 436.624 | 0.02736 | 0.04232 |  | 0.06616 | 0.06462 | 0.02563 |
| 437.121 | 0.02763 | 0.04314 |  | 0.06644 | 0.06489 | 0.02514 |
| 437.619 | 0.02814 | 0.04386 |  | 0.06841 | 0.06622 | 0.02591 |
| 438.114 | 0.02789 | 0.04429 |  | 0.07095 | 0.06793 | 0.02699 |
| 438.611 | 0.02962 | 0.04495 |  | 0.07321 | 0.07142 | 0.02858 |
| 439.106 | 0.03057 | 0.04542 |  | 0.07529 | 0.07381 | 0.03035 |
| 439.604 | 0.03169 | 0.04575 |  | 0.07672 | 0.07456 | 0.03109 |
| 440.099 | 0.03296 | 0.04598 |  | 0.07678 | 0.07557 | 0.03160 |
| 440.596 | 0.03439 | 0.04648 |  | 0.07572 | 0.07651 | 0.03153 |
| 441.091 | 0.03589 | 0.04749 |  | 0.07440 | 0.07785 | 0.03200 |
| 441.588 | 0.03714 | 0.04842 |  | 0.07386 | 0.07903 | 0.03295 |
| 442.084 | 0.03855 | 0.04946 |  | 0.07507 | 0.08155 | 0.03441 |
| 442.581 | 0.04016 | 0.05063 |  | 0.07618 | 0.08349 | 0.03633 |
| 443.078 | 0.04188 | 0.05191 |  | 0.07906 | 0.08594 | 0.03843 |
| 443.573 | 0.04373 | 0.05334 |  | 0.08315 | 0.08596 | 0.03951 |
| 444.07 | 0.04556 | 0.05488 |  | 0.08586 | 0.08573 | 0.04071 |
| 444.565 | 0.04784 | 0.05656 |  | 0.08753 | 0.08703 | 0.04305 |
| 445.062 | 0.05077 | 0.05831 |  | 0.08777 | 0.08751 | 0.04691 |
| 445.557 | 0.05307 | 0.06101 |  | 0.08806 | 0.08786 | 0.04902 |
| 446.051 | 0.05578 | 0.06410 |  | 0.08911 | 0.09085 | 0.05119 |
| 446.548 | 0.05852 | 0.06666 |  | 0.09116 | 0.09436 | 0.05363 |
| 447.043 | 0.06166 | 0.06862 |  | 0.09454 | 0.09895 | 0.05643 |
| 447.54 | 0.06504 | 0.07012 |  | 0.09847 | 0.10461 | 0.05954 |
| 448.035 | 0.07145 | 0.07154 |  | 0.10277 | 0.11036 | 0.06390 |
| 448.532 | 0.07698 | 0.07583 |  | 0.10720 | 0.11583 | 0.06831 |
| 449.026 | 0.08176 | 0.08021 |  | 0.11199 | 0.12111 | 0.07377 |
| 449.523 | 0.08654 | 0.08479 |  | 0.11663 | 0.12620 | 0.07997 |
| 450.018 | 0.09133 | 0.08947 |  | 0.12078 | 0.13110 | 0.08501 |
| 450.514 | 0.09504 | 0.09500 |  | 0.12486 | 0.13595 | 0.08922 |
| 451.009 | 0.09900 | 0.09938 |  | 0.12887 | 0.14068 | 0.09294 |
| 451.504 | 0.10295 | 0.10235 |  | 0.13278 | 0.14535 | 0.09651 |
| 452 | 0.10776 | 0.10502 |  | 0.13662 | 0.14993 | 0.10003 |
| 452.495 | 0.11360 | 0.10809 |  | 0.14071 | 0.15459 | 0.10393 |
| 452.991 | 0.11992 | 0.11296 |  | 0.14417 | 0.15945 | 0.11122 |
| 453.486 | 0.12773 | 0.11958 |  | 0.14862 | 0.16505 | 0.11751 |
| 453.98 | 0.13721 | 0.12907 |  | 0.15496 | 0.17178 | 0.12525 |
| 454.477 | 0.14837 | 0.13855 |  | 0.16345 | 0.17966 | 0.13468 |
| 454.971 | 0.16142 | 0.14841 |  | 0.17412 | 0.18934 | 0.14626 |
| 455.467 | 0.17635 | 0.15955 |  | 0.18693 | 0.20130 | 0.15992 |
| 455.962 | 0.19375 | 0.17212 |  | 0.20177 | 0.21618 | 0.17424 |
| 456.456 | 0.21387 | 0.18537 |  | 0.21883 | 0.23421 | 0.19115 |
| 456.952 | 0.23498 | 0.20242 |  | 0.23796 | 0.25533 | 0.21102 |
| 457.447 | 0.26157 | 0.22264 |  | 0.25947 | 0.27942 | 0.23374 |
| 457.941 | 0.29364 | 0.24619 |  | 0.28220 | 0.30756 | 0.25974 |
| 458.437 | 0.33017 | 0.27361 |  | 0.30868 | 0.34054 | 0.29034 |
| 458.931 | 0.37246 | 0.30650 |  | 0.34016 | 0.37892 | 0.32659 |
| 459.425 | 0.42066 | 0.34534 |  | 0.37899 | 0.42228 | 0.36979 |
| 459.922 | 0.47461 | 0.38986 |  | 0.42468 | 0.47108 | 0.41924 |
| 460.416 | 0.53450 | 0.44276 |  | 0.47715 | 0.52533 | 0.47303 |
| 460.91 | 0.60039 | 0.50449 |  | 0.53614 | 0.58524 | 0.53564 |
| 461.406 | 0.67234 | 0.56807 |  | 0.60080 | 0.65147 | 0.60104 |
| 461.9 | 0.75100 | 0.63960 |  | 0.67119 | 0.72540 | 0.67273 |
| 462.394 | 0.83487 | 0.71364 |  | 0.74928 | 0.80631 | 0.74920 |
| 462.888 | 0.90684 | 0.78696 |  | 0.83157 | 0.88211 | 0.82027 |
| 463.384 | 0.96110 | 0.85660 |  | 0.90135 | 0.93311 | 0.87907 |
| 463.878 | 0.99057 | 0.91980 |  | 0.96133 | 0.97981 | 0.94991 |
| 464.372 | 1.00000 | 0.97251 |  | 0.98788 | 0.99901 | 0.98458 |
| 464.867 | 0.99074 | 1.00000 |  | 1.00000 | 1.00000 | 1.00000 |
| 465.361 | 0.96100 | 0.98660 |  | 0.97851 | 0.98288 | 0.98961 |
| 465.855 | 0.91247 | 0.94410 |  | 0.93740 | 0.94765 | 0.95651 |
| 466.349 | 0.84206 | 0.88522 |  | 0.88688 | 0.87738 | 0.88744 |
| 466.845 | 0.76213 | 0.81855 |  | 0.81237 | 0.81886 | 0.82862 |
| 467.338 | 0.69031 | 0.75001 |  | 0.74361 | 0.74124 | 0.74953 |
| 467.832 | 0.62268 | 0.67205 |  | 0.67355 | 0.67085 | 0.68369 |
| 468.326 | 0.55729 | 0.60445 |  | 0.60803 | 0.61149 | 0.61731 |
| 468.819 | 0.49728 | 0.53945 |  | 0.54883 | 0.55328 | 0.55678 |
| 469.315 | 0.44401 | 0.48564 |  | 0.49515 | 0.50001 | 0.49937 |
| 469.809 | 0.39787 | 0.43733 |  | 0.44742 | 0.45252 | 0.44548 |
| 470.302 | 0.36052 | 0.39878 |  | 0.40487 | 0.41042 | 0.40126 |
| 470.796 | 0.32863 | 0.36174 |  | 0.36812 | 0.37404 | 0.36342 |
| 471.289 | 0.30028 | 0.32901 |  | 0.33689 | 0.34373 | 0.32889 |
| 471.785 | 0.27519 | 0.29931 |  | 0.31074 | 0.32134 | 0.30055 |
| 472.278 | 0.25349 | 0.27487 |  | 0.28921 | 0.30148 | 0.27700 |
| 472.772 | 0.23422 | 0.25435 |  | 0.26946 | 0.28432 | 0.25681 |
| 473.265 | 0.21728 | 0.23706 |  | 0.25169 | 0.26863 | 0.23982 |
| 473.758 | 0.20193 | 0.22219 |  | 0.23652 | 0.25386 | 0.22404 |
| 474.252 | 0.18831 | 0.20968 |  | 0.22332 | 0.23977 | 0.20605 |
| 474.747 | 0.17651 | 0.19845 |  | 0.21130 | 0.22629 | 0.18985 |
| 475.24 | 0.16563 | 0.18697 |  | 0.20057 | 0.21398 | 0.17623 |
| 475.734 | 0.15503 | 0.17532 |  | 0.19086 | 0.19988 | 0.16630 |
| 476.227 | 0.14611 | 0.16428 |  | 0.18270 | 0.18532 | 0.15900 |
| 476.72 | 0.13828 | 0.15589 |  | 0.17536 | 0.17803 | 0.14886 |
| 477.213 | 0.13167 | 0.14776 |  | 0.16878 | 0.17326 | 0.14017 |
| 477.707 | 0.12617 | 0.14079 |  | 0.16266 | 0.17057 | 0.13300 |
| 478.2 | 0.12169 | 0.13539 |  | 0.15712 | 0.16680 | 0.13101 |
| 478.695 | 0.11706 | 0.13111 |  | 0.15216 | 0.16104 | 0.12583 |
| 479.188 | 0.11234 | 0.12690 |  | 0.14772 | 0.15416 | 0.12056 |
| 479.681 | 0.10786 | 0.12270 |  | 0.14385 | 0.15093 | 0.11584 |
| 480.174 | 0.10378 | 0.11848 |  | 0.14049 | 0.15114 | 0.11150 |
| 480.667 | 0.10005 | 0.11452 |  | 0.13775 | 0.15102 | 0.10762 |
| 481.16 | 0.09669 | 0.11086 |  | 0.13472 | 0.14758 | 0.10417 |
| 481.653 | 0.09368 | 0.10821 |  | 0.13124 | 0.14354 | 0.10115 |
| 482.146 | 0.09098 | 0.10555 |  | 0.12891 | 0.13925 | 0.09857 |
| 482.639 | 0.08875 | 0.10283 |  | 0.12704 | 0.13496 | 0.09579 |
| 483.132 | 0.08685 | 0.10053 |  | 0.12679 | 0.13100 | 0.09284 |
| 483.624 | 0.08448 | 0.09838 |  | 0.12740 | 0.12770 | 0.08995 |
| 484.117 | 0.08301 | 0.09631 |  | 0.12534 | 0.12625 | 0.08728 |
| 484.61 | 0.08167 | 0.09433 |  | 0.12294 | 0.12487 | 0.08484 |
| 485.103 | 0.07988 | 0.09240 |  | 0.11998 | 0.12434 | 0.08206 |
| 485.596 | 0.07812 | 0.09024 |  | 0.11675 | 0.12159 | 0.07822 |
| 486.088 | 0.07635 | 0.08796 |  | 0.11535 | 0.11892 | 0.07643 |
| 486.581 | 0.07460 | 0.08612 |  | 0.12300 | 0.11712 | 0.07530 |
| 487.074 | 0.07281 | 0.08466 |  | 0.11063 | 0.11574 | 0.07509 |
| 487.566 | 0.07099 | 0.08365 |  | 0.10916 | 0.11503 | 0.07481 |
| 488.059 | 0.06922 | 0.08303 |  | 0.10783 | 0.11483 | 0.07387 |
| 488.551 | 0.06759 | 0.08265 |  | 0.10891 | 0.11546 | 0.07308 |
| 489.044 | 0.06643 | 0.08209 |  | 0.10574 | 0.11657 | 0.07237 |
| 489.537 | 0.06564 | 0.08050 |  | 0.10411 | 0.11842 | 0.07067 |
| 490.029 | 0.06562 | 0.07875 |  | 0.10300 | 0.12026 | 0.06924 |
| 490.522 | 0.06593 | 0.07572 |  | 0.10247 | 0.11999 | 0.06861 |
| 491.014 | 0.06657 | 0.07421 |  | 0.10251 | 0.11916 | 0.06793 |
| 491.506 | 0.06745 | 0.07332 |  | 0.10375 | 0.11915 | 0.06746 |
| 491.999 | 0.06838 | 0.07299 |  | 0.10533 | 0.11966 | 0.06830 |
| 492.491 | 0.06870 | 0.07326 |  | 0.09484 | 0.12087 | 0.06894 |
| 492.983 | 0.06914 | 0.07385 |  | 0.09305 | 0.12287 | 0.06861 |
| 493.476 | 0.06913 | 0.07356 |  | 0.09894 | 0.12557 | 0.06923 |
| 493.968 | 0.06940 | 0.07245 |  | 0.10933 | 0.12963 | 0.06977 |
| 494.46 | 0.06997 | 0.07043 |  | 0.10582 | 0.13280 | 0.07086 |
| 494.953 | 0.07108 | 0.07143 |  | 0.10530 | 0.13444 | 0.07252 |
| 495.445 | 0.07277 | 0.07293 |  | 0.10430 | 0.13688 | 0.07462 |
| 495.937 | 0.07489 | 0.07529 |  | 0.10318 | 0.13898 | 0.07733 |
| 496.427 | 0.07748 | 0.07720 |  | 0.10281 | 0.14321 | 0.08058 |
| 496.919 | 0.08068 | 0.07666 |  | 0.10285 | 0.14875 | 0.08446 |
| 497.411 | 0.08452 | 0.07765 |  | 0.10330 | 0.15552 | 0.08869 |
| 497.903 | 0.08898 | 0.07964 |  | 0.10459 | 0.16349 | 0.09353 |
| 498.395 | 0.09422 | 0.08191 |  | 0.10644 | 0.17235 | 0.09901 |
| 498.887 | 0.10020 | 0.08453 |  | 0.10856 | 0.18200 | 0.10454 |
| 499.379 | 0.10695 | 0.08753 |  | 0.11125 | 0.19238 | 0.11113 |
| 499.871 | 0.11445 | 0.09092 |  | 0.11308 | 0.20362 | 0.11995 |
| 500.361 | 0.12250 | 0.09403 |  | 0.11348 | 0.21564 | 0.12960 |
| 500.853 | 0.13072 | 0.09759 |  | 0.11379 | 0.22843 | 0.13961 |
| 501.345 | 0.13690 | 0.10138 |  | 0.11675 | 0.24191 | 0.14803 |
| 501.837 | 0.14209 | 0.10549 |  | 0.12202 | 0.25367 | 0.15610 |
| 502.329 | 0.14610 | 0.10982 |  | 0.12653 | 0.26209 | 0.16144 |
| 502.821 | 0.14740 | 0.11492 |  | 0.12997 | 0.26813 | 0.16418 |
| 503.31 | 0.14565 | 0.11439 |  | 0.12048 | 0.26918 | 0.16462 |
| 503.802 | 0.14175 | 0.11117 |  | 0.11634 | 0.26524 | 0.16217 |
| 504.294 | 0.13617 | 0.10764 |  | 0.11488 | 0.25653 | 0.15769 |
| 504.786 | 0.12860 | 0.10488 |  | 0.11358 | 0.24406 | 0.15107 |
| 505.277 | 0.11961 | 0.10106 |  | 0.11192 | 0.23128 | 0.14223 |
| 505.767 | 0.11117 | 0.09640 |  | 0.11052 | 0.21772 | 0.13180 |
| 506.259 | 0.10330 | 0.09173 |  | 0.10910 | 0.20378 | 0.12168 |
| 506.75 | 0.09698 | 0.08699 |  | 0.10763 | 0.18984 | 0.11215 |
| 507.242 | 0.09061 | 0.08304 |  | 0.10617 | 0.17729 | 0.10313 |
| 507.731 | 0.08414 | 0.07979 |  | 0.10469 | 0.16563 | 0.09502 |
| 508.223 | 0.07845 | 0.07665 |  | 0.10317 | 0.15528 | 0.08980 |
| 508.714 | 0.07352 | 0.07337 |  | 0.10155 | 0.14605 | 0.08351 |
| 509.206 | 0.06943 | 0.07029 |  | 0.09979 | 0.13787 | 0.07802 |
| 509.695 | 0.06626 | 0.06744 |  | 0.09791 | 0.13069 | 0.07301 |
| 510.186 | 0.06284 | 0.06484 |  | 0.09594 | 0.12451 | 0.06841 |
| 510.678 | 0.05900 | 0.06247 |  | 0.09388 | 0.11946 | 0.06418 |
| 511.169 | 0.05565 | 0.06040 |  | 0.09177 | 0.11504 | 0.06059 |
| 511.659 | 0.05296 | 0.05862 |  | 0.08951 | 0.11138 | 0.05797 |
| 512.15 | 0.05125 | 0.05725 |  | 0.08718 | 0.10847 | 0.05644 |
| 512.641 | 0.04962 | 0.05613 |  | 0.08481 | 0.10639 | 0.05485 |
| 513.13 | 0.04828 | 0.05519 |  | 0.08266 | 0.10578 | 0.05269 |
| 513.622 | 0.04689 | 0.05365 |  | 0.08167 | 0.10713 | 0.05062 |
| 514.113 | 0.04551 | 0.05182 |  | 0.08077 | 0.10141 | 0.04869 |
| 514.602 | 0.04400 | 0.05172 |  | 0.08044 | 0.09597 | 0.04691 |
| 515.093 | 0.04265 | 0.05691 |  | 0.08090 | 0.09373 | 0.04530 |
| 515.584 | 0.04142 | 0.05262 |  | 0.08163 | 0.09291 | 0.04330 |
| 516.075 | 0.04026 | 0.05091 |  | 0.08240 | 0.09208 | 0.04141 |
| 516.564 | 0.03921 | 0.04959 |  | 0.08238 | 0.09142 | 0.03986 |
| 517.055 | 0.03824 | 0.04936 |  | 0.08242 | 0.09063 | 0.04057 |
| 517.544 | 0.03740 | 0.04874 |  | 0.08172 | 0.08891 | 0.04054 |
| 518.035 | 0.03644 | 0.04775 |  | 0.08089 | 0.08787 | 0.03993 |
| 518.526 | 0.03561 | 0.04737 |  | 0.07943 | 0.08712 | 0.03903 |
| 519.015 | 0.03492 | 0.04726 |  | 0.07789 | 0.08641 | 0.03786 |
| 519.506 | 0.03461 | 0.04738 |  | 0.07636 | 0.08558 | 0.03721 |
| 519.997 | 0.03611 | 0.04875 |  | 0.07498 | 0.08467 | 0.03679 |
| 520.486 | 0.03430 | 0.04931 |  | 0.07428 | 0.08353 | 0.03660 |
| 520.977 | 0.03220 | 0.04890 |  | 0.07366 | 0.08233 | 0.03613 |
| 521.465 | 0.03160 | 0.04839 |  | 0.07323 | 0.08010 | 0.03564 |
| 521.956 | 0.03216 | 0.04771 |  | 0.07297 | 0.07781 | 0.03518 |
| 522.447 | 0.03220 | 0.04703 |  | 0.07264 | 0.07567 | 0.03360 |
| 522.936 | 0.03214 | 0.04627 |  | 0.07193 | 0.07401 | 0.03364 |
| 523.426 | 0.03169 | 0.04572 |  | 0.07081 | 0.07440 | 0.03312 |
| 523.915 | 0.03126 | 0.04512 |  | 0.07119 | 0.07456 | 0.03192 |
| 524.406 | 0.03089 | 0.04445 |  | 0.07251 | 0.07557 | 0.03050 |
| 524.894 | 0.03047 | 0.04365 |  | 0.07333 | 0.07706 | 0.02945 |
| 525.385 | 0.02965 | 0.04283 |  | 0.07399 | 0.07898 | 0.02872 |
| 525.875 | 0.02884 | 0.04196 |  | 0.07432 | 0.08023 | 0.02728 |
| 526.364 | 0.02827 | 0.04108 |  | 0.07421 | 0.07865 | 0.02688 |
| 526.854 | 0.02799 | 0.04014 |  | 0.07387 | 0.07713 | 0.02752 |
| 527.343 | 0.02797 | 0.04010 |  | 0.07324 | 0.07552 | 0.02778 |
| 527.833 | 0.02797 | 0.04038 |  | 0.07237 | 0.07403 | 0.02712 |
| 528.322 | 0.02777 | 0.04069 |  | 0.07134 | 0.07270 | 0.02656 |
| 528.812 | 0.02760 | 0.04101 |  | 0.07029 | 0.07146 | 0.02607 |
| 529.301 | 0.02744 | 0.04135 |  | 0.06973 | 0.07026 | 0.02583 |
| 529.791 | 0.02723 | 0.04166 |  | 0.06952 | 0.06920 | 0.02613 |
| 530.279 | 0.02701 | 0.04156 |  | 0.06937 | 0.06864 | 0.02693 |
| 530.77 | 0.02674 | 0.04143 |  | 0.06926 | 0.06941 | 0.02755 |
| 531.258 | 0.02468 | 0.04128 |  | 0.06920 | 0.07315 | 0.02821 |
| 531.748 | 0.02437 | 0.04108 |  | 0.06938 | 0.07875 | 0.02804 |
| 532.236 | 0.02496 | 0.04102 |  | 0.06942 | 0.06793 | 0.02724 |
| 532.726 | 0.02550 | 0.04103 |  | 0.06896 | 0.06806 | 0.02642 |
| 533.215 | 0.02517 | 0.04065 |  | 0.06815 | 0.06865 | 0.02543 |
| 533.705 | 0.02481 | 0.04023 |  | 0.06714 | 0.06830 | 0.02291 |
| 534.193 | 0.02450 | 0.03976 |  | 0.06639 | 0.06839 | 0.02073 |
| 534.681 | 0.02446 | 0.03979 |  | 0.06586 | 0.06870 | 0.02103 |
| 535.171 | 0.02441 | 0.03994 |  | 0.06530 | 0.06939 | 0.02196 |
| 535.659 | 0.02460 | 0.04037 |  | 0.06495 | 0.07076 | 0.02326 |
| 536.149 | 0.02468 | 0.04135 |  | 0.06472 | 0.07223 | 0.02451 |
| 536.637 | 0.02463 | 0.04141 |  | 0.06469 | 0.07280 | 0.02548 |
| 537.127 | 0.02451 | 0.04106 |  | 0.06495 | 0.07323 | 0.02563 |
| 537.615 | 0.02440 | 0.04054 |  | 0.06572 | 0.07210 | 0.02377 |
| 538.103 | 0.02415 | 0.03991 |  | 0.06672 | 0.07048 | 0.02245 |
| 538.593 | 0.02332 | 0.03910 |  | 0.06746 | 0.06890 | 0.02133 |
| 539.08 | 0.02287 | 0.03824 |  | 0.06784 | 0.06742 | 0.02046 |
| 539.57 | 0.02182 | 0.03776 |  | 0.06799 | 0.06604 | 0.01989 |
| 540.058 | 0.02202 | 0.03750 |  | 0.06793 | 0.06528 | 0.01961 |
| 540.546 | 0.02238 | 0.03788 |  | 0.06768 | 0.06478 | 0.01959 |
| 541.036 | 0.02278 | 0.03865 |  | 0.06718 | 0.06452 | 0.01984 |
| 541.523 | 0.02247 | 0.03617 |  | 0.06653 | 0.06444 | 0.02044 |
| 542.011 | 0.02148 | 0.03505 |  | 0.06580 | 0.06466 | 0.02118 |
| 542.501 | 0.02085 | 0.03447 |  | 0.06521 | 0.06352 | 0.02163 |
| 542.988 | 0.02056 | 0.03486 |  | 0.06610 | 0.06248 | 0.02140 |
| 543.476 | 0.02062 | 0.03560 |  | 0.06649 | 0.06159 | 0.02113 |
| 543.965 | 0.02088 | 0.03605 |  | 0.06622 | 0.06218 | 0.02062 |
| 544.453 | 0.02020 | 0.03642 |  | 0.06548 | 0.06260 | 0.02014 |
| 544.94 | 0.01959 | 0.03670 |  | 0.06505 | 0.06382 | 0.01937 |
| 545.43 | 0.01940 | 0.03681 |  | 0.06447 | 0.06594 | 0.01841 |
| 545.917 | 0.01935 | 0.03684 |  | 0.06427 | 0.06665 | 0.01775 |
| 546.405 | 0.01946 | 0.03680 |  | 0.06458 | 0.06649 | 0.01763 |
| 546.894 | 0.01973 | 0.03684 |  | 0.06519 | 0.06535 | 0.01859 |
| 547.382 | 0.02007 | 0.03689 |  | 0.06598 | 0.06319 | 0.01941 |
| 547.869 | 0.02013 | 0.03697 |  | 0.06709 | 0.06194 | 0.01996 |
| 548.358 | 0.02000 | 0.03687 |  | 0.06797 | 0.06155 | 0.02016 |
| 548.846 | 0.01964 | 0.03666 |  | 0.06707 | 0.06157 | 0.02049 |
| 549.333 | 0.01917 | 0.03637 |  | 0.06664 | 0.06233 | 0.02037 |
| 549.82 | 0.01870 | 0.03600 |  | 0.06665 | 0.06342 | 0.01997 |
| 550.31 | 0.01802 | 0.03551 |  | 0.06771 | 0.06406 | 0.01938 |
| 550.797 | 0.01789 | 0.03502 |  | 0.06773 | 0.06450 | 0.01877 |
| 551.284 | 0.01806 | 0.03467 |  | 0.06722 | 0.06470 | 0.01809 |
| 551.771 | 0.01862 | 0.03466 |  | 0.06622 | 0.06477 | 0.01735 |
| 552.26 | 0.01906 | 0.03479 |  | 0.06382 | 0.06463 | 0.01572 |
| 552.747 | 0.01942 | 0.03486 |  | 0.06214 | 0.06423 | 0.01559 |
| 553.234 | 0.01967 | 0.03494 |  | 0.06085 | 0.06365 | 0.01588 |
| 553.721 | 0.01982 | 0.03500 |  | 0.05976 | 0.06271 | 0.01646 |
| 554.208 | 0.01985 | 0.03513 |  | 0.05908 | 0.06166 | 0.01695 |
| 554.698 | 0.01983 | 0.03526 |  | 0.05888 | 0.06117 | 0.01729 |
| 555.184 | 0.01962 | 0.03525 |  | 0.05907 | 0.06101 | 0.01750 |
| 555.671 | 0.01926 | 0.03502 |  | 0.05966 | 0.06224 | 0.01759 |
| 556.158 | 0.01870 | 0.03430 |  | 0.06064 | 0.06344 | 0.01756 |
| 556.645 | 0.01828 | 0.03357 |  | 0.06193 | 0.06362 | 0.01735 |
| 557.134 | 0.01804 | 0.03304 |  | 0.06296 | 0.06228 | 0.01699 |
| 557.621 | 0.01830 | 0.03309 |  | 0.06391 | 0.06129 | 0.01639 |
| 558.108 | 0.01976 | 0.03341 |  | 0.06446 | 0.05888 | 0.01586 |
| 558.595 | 0.01930 | 0.03415 |  | 0.06482 | 0.05727 | 0.01539 |
| 559.081 | 0.01921 | 0.03429 |  | 0.06486 | 0.05678 | 0.01515 |
| 559.568 | 0.01905 | 0.03423 |  | 0.06468 | 0.05619 | 0.01523 |
| 560.055 | 0.01857 | 0.03404 |  | 0.06404 | 0.05735 | 0.01553 |
| 560.543 | 0.01801 | 0.03373 |  | 0.06313 | 0.05886 | 0.01592 |
| 561.03 | 0.01753 | 0.03332 |  | 0.06208 | 0.06130 | 0.01648 |
| 561.517 | 0.01712 | 0.03282 |  | 0.06097 | 0.06195 | 0.01703 |
| 562.003 | 0.01680 | 0.03288 |  | 0.06049 | 0.06273 | 0.01751 |
| 562.49 | 0.01655 | 0.03300 |  | 0.05990 | 0.06216 | 0.01786 |
| 562.977 | 0.01639 | 0.03288 |  | 0.05953 | 0.05966 | 0.01787 |
| 563.463 | 0.01630 | 0.03247 |  | 0.05929 | 0.05904 | 0.01779 |
| 563.95 | 0.01626 | 0.03164 |  | 0.05929 | 0.05840 | 0.01757 |
| 564.436 | 0.01629 | 0.03110 |  | 0.05948 | 0.05980 | 0.01718 |
| 564.924 | 0.01641 | 0.03093 |  | 0.06002 | 0.06194 | 0.01660 |
| 565.411 | 0.01660 | 0.03105 |  | 0.06107 | 0.06036 | 0.01594 |
| 565.897 | 0.01686 | 0.03132 |  | 0.06119 | 0.05866 | 0.01555 |
| 566.384 | 0.01716 | 0.03177 |  | 0.06072 | 0.05836 | 0.01553 |
| 566.87 | 0.01756 | 0.03236 |  | 0.06083 | 0.05820 | 0.01598 |
| 567.356 | 0.01800 | 0.03318 |  | 0.06090 | 0.05867 | 0.01589 |
| 567.843 | 0.01778 | 0.03390 |  | 0.06084 | 0.05831 | 0.01566 |
| 568.329 | 0.01767 | 0.03393 |  | 0.06079 | 0.05766 | 0.01533 |
| 568.815 | 0.01803 | 0.03367 |  | 0.06068 | 0.05713 | 0.01498 |
| 569.301 | 0.01842 | 0.03311 |  | 0.06049 | 0.05666 | 0.01470 |
| 569.788 | 0.01764 | 0.03223 |  | 0.06023 | 0.05631 | 0.01455 |
| 570.274 | 0.01691 | 0.03174 |  | 0.05994 | 0.05597 | 0.01446 |
| 570.76 | 0.01668 | 0.03114 |  | 0.05963 | 0.05591 | 0.01447 |
| 571.246 | 0.01673 | 0.03078 |  | 0.05950 | 0.05627 | 0.01449 |
| 571.732 | 0.01666 | 0.03075 |  | 0.05952 | 0.05704 | 0.01457 |
| 572.218 | 0.01658 | 0.03075 |  | 0.05957 | 0.05788 | 0.01467 |
| 572.704 | 0.01673 | 0.03076 |  | 0.05963 | 0.05828 | 0.01475 |
| 573.19 | 0.01690 | 0.03079 |  | 0.05970 | 0.05827 | 0.01478 |
| 573.676 | 0.01705 | 0.03076 |  | 0.05981 | 0.05761 | 0.01464 |
| 574.162 | 0.01716 | 0.03060 |  | 0.05997 | 0.05760 | 0.01474 |
| 574.648 | 0.01717 | 0.03040 |  | 0.05988 | 0.05772 | 0.01568 |
| 575.134 | 0.01706 | 0.03011 |  | 0.05991 | 0.05746 | 0.01635 |
| 575.62 | 0.01681 | 0.02976 |  | 0.05909 | 0.05679 | 0.01605 |
| 576.106 | 0.01662 | 0.02937 |  | 0.05817 | 0.05613 | 0.01547 |
| 576.591 | 0.01642 | 0.02992 |  | 0.05705 | 0.05542 | 0.01555 |
| 577.077 | 0.01652 | 0.03019 |  | 0.05548 | 0.05470 | 0.01549 |
| 577.563 | 0.01665 | 0.03093 |  | 0.05476 | 0.05377 | 0.01538 |
| 578.049 | 0.01670 | 0.03117 |  | 0.05442 | 0.05409 | 0.01539 |
| 578.532 | 0.01671 | 0.03100 |  | 0.05477 | 0.05423 | 0.01546 |
| 579.018 | 0.01665 | 0.03068 |  | 0.05597 | 0.05484 | 0.01536 |
| 579.504 | 0.01645 | 0.03018 |  | 0.05544 | 0.05543 | 0.01497 |
| 579.99 | 0.01613 | 0.02970 |  | 0.05471 | 0.05609 | 0.01483 |
| 580.475 | 0.01560 | 0.02938 |  | 0.05466 | 0.05688 | 0.01478 |
| 580.961 | 0.01531 | 0.02934 |  | 0.05482 | 0.05783 | 0.01464 |
| 581.446 | 0.01618 | 0.02994 |  | 0.05457 | 0.05790 | 0.01445 |
| 581.932 | 0.01754 | 0.03026 |  | 0.05414 | 0.05720 | 0.01414 |
| 582.417 | 0.01755 | 0.03059 |  | 0.05387 | 0.05649 | 0.01379 |
| 582.901 | 0.01682 | 0.03047 |  | 0.05382 | 0.05656 | 0.01380 |
| 583.386 | 0.01640 | 0.03019 |  | 0.05402 | 0.05701 | 0.01391 |
| 583.872 | 0.01608 | 0.03002 |  | 0.05441 | 0.05754 | 0.01396 |
| 584.357 | 0.01579 | 0.02987 |  | 0.05497 | 0.05812 | 0.01405 |
| 584.842 | 0.01579 | 0.02983 |  | 0.05552 | 0.05827 | 0.01419 |
| 585.328 | 0.01621 | 0.02987 |  | 0.05621 | 0.05719 | 0.01427 |
| 585.811 | 0.01657 | 0.02989 |  | 0.05703 | 0.05336 | 0.01438 |
| 586.296 | 0.01603 | 0.02988 |  | 0.05767 | 0.05486 | 0.01453 |
| 586.782 | 0.01695 | 0.02972 |  | 0.05800 | 0.05832 | 0.01464 |
| 587.267 | 0.01689 | 0.02960 |  | 0.05743 | 0.05901 | 0.01470 |
| 587.752 | 0.01694 | 0.02961 |  | 0.05632 | 0.05915 | 0.01471 |
| 588.237 | 0.01711 | 0.02959 |  | 0.05548 | 0.05935 | 0.01465 |
| 588.721 | 0.01732 | 0.02959 |  | 0.05502 | 0.05897 | 0.01452 |
| 589.206 | 0.01749 | 0.02937 |  | 0.05458 | 0.05811 | 0.01442 |
| 589.691 | 0.01753 | 0.02927 |  | 0.05475 | 0.05725 | 0.01437 |
| 590.176 | 0.01744 | 0.02934 |  | 0.05500 | 0.05563 | 0.01448 |
| 590.659 | 0.01737 | 0.02933 |  | 0.05503 | 0.05466 | 0.01468 |
| 591.144 | 0.01742 | 0.02918 |  | 0.05496 | 0.05359 | 0.01478 |
| 591.629 | 0.01739 | 0.02907 |  | 0.05451 | 0.05282 | 0.01477 |
| 592.114 | 0.01715 | 0.02899 |  | 0.05414 | 0.05279 | 0.01462 |
| 592.597 | 0.01682 | 0.02895 |  | 0.05363 | 0.05295 | 0.01436 |
| 593.082 | 0.01647 | 0.02892 |  | 0.05296 | 0.05329 | 0.01398 |
| 593.567 | 0.01625 | 0.02884 |  | 0.05215 | 0.05374 | 0.01394 |
| 594.052 | 0.01622 | 0.02869 |  | 0.05140 | 0.05388 | 0.01465 |
| 594.535 | 0.01693 | 0.02814 |  | 0.05122 | 0.05367 | 0.01555 |
| 595.02 | 0.01695 | 0.02763 |  | 0.05161 | 0.05276 | 0.01630 |
| 595.504 | 0.01686 | 0.02749 |  | 0.05210 | 0.05147 | 0.01652 |
| 595.987 | 0.01690 | 0.02749 |  | 0.05255 | 0.05040 | 0.01640 |
| 596.472 | 0.01688 | 0.02741 |  | 0.05293 | 0.04973 | 0.01598 |
| 596.957 | 0.01678 | 0.02741 |  | 0.05281 | 0.04973 | 0.01538 |
| 597.441 | 0.01683 | 0.02740 |  | 0.05151 | 0.05029 | 0.01484 |
| 597.924 | 0.01673 | 0.02736 |  | 0.05001 | 0.05114 | 0.01439 |
| 598.409 | 0.01651 | 0.02752 |  | 0.04860 | 0.05218 | 0.01406 |
| 598.893 | 0.01611 | 0.02827 |  | 0.04763 | 0.05342 | 0.01378 |
| 599.376 | 0.01576 | 0.02868 |  | 0.04718 | 0.05404 | 0.01358 |
| 599.86 | 0.01567 | 0.02860 |  | 0.04736 | 0.05441 | 0.01343 |
| 600.345 | 0.01620 | 0.02859 |  | 0.04820 | 0.05423 | 0.01336 |
| 600.828 | 0.01679 | 0.02831 |  | 0.04861 | 0.05369 | 0.01334 |
| 601.312 | 0.01724 | 0.02768 |  | 0.04866 | 0.05321 | 0.01340 |
| 601.794 | 0.01719 | 0.02701 |  | 0.04853 | 0.05224 | 0.01357 |
| 602.279 | 0.01608 | 0.02647 |  | 0.04871 | 0.05158 | 0.01376 |
| 602.763 | 0.01578 | 0.02604 |  | 0.04897 | 0.05115 | 0.01380 |
| 603.246 | 0.01550 | 0.02564 |  | 0.04933 | 0.05157 | 0.01357 |
| 603.73 | 0.01538 | 0.02528 |  | 0.04968 | 0.05227 | 0.01344 |
| 604.215 | 0.01561 | 0.02523 |  | 0.05007 | 0.05346 | 0.01331 |
| 604.697 | 0.01555 | 0.02514 |  | 0.05045 | 0.05444 | 0.01293 |
| 605.181 | 0.01538 | 0.02518 |  | 0.05069 | 0.05473 | 0.01269 |
| 605.663 | 0.01518 | 0.02527 |  | 0.05072 | 0.05428 | 0.01312 |
| 606.148 | 0.01511 | 0.02554 |  | 0.05034 | 0.05344 | 0.01351 |
| 606.63 | 0.01524 | 0.02598 |  | 0.04947 | 0.05267 | 0.01409 |
| 607.114 | 0.01557 | 0.02671 |  | 0.04854 | 0.05194 | 0.01441 |
| 607.598 | 0.01570 | 0.02785 |  | 0.04738 | 0.05200 | 0.01398 |
| 608.08 | 0.01529 | 0.02893 |  | 0.04608 | 0.05286 | 0.01378 |
| 608.564 | 0.01439 | 0.02721 |  | 0.04512 | 0.05405 | 0.01382 |
| 609.047 | 0.01346 | 0.02658 |  | 0.04521 | 0.05391 | 0.01394 |
| 609.531 | 0.01318 | 0.02585 |  | 0.04530 | 0.05319 | 0.01395 |
| 610.013 | 0.01321 | 0.02528 |  | 0.04563 | 0.05227 | 0.01408 |
| 610.497 | 0.01347 | 0.02496 |  | 0.04542 | 0.04920 | 0.01438 |
| 610.979 | 0.01402 | 0.02491 |  | 0.04452 | 0.04809 | 0.01485 |
| 611.463 | 0.01224 | 0.02481 |  | 0.04393 | 0.04808 | 0.01562 |
| 611.945 | 0.01615 | 0.02476 |  | 0.04366 | 0.04865 | 0.01468 |
| 612.428 | 0.01584 | 0.02475 |  | 0.04359 | 0.04918 | 0.01378 |
| 612.91 | 0.01729 | 0.02478 |  | 0.04367 | 0.04962 | 0.01326 |
| 613.394 | 0.01454 | 0.02452 |  | 0.04375 | 0.04958 | 0.01307 |
| 613.416 | 0.01441 | 0.02446 |  | 0.04366 | 0.04954 | 0.01306 |
| 613.906 | 0.01286 | 0.02445 |  | 0.04412 | 0.04906 | 0.01296 |
| 614.394 | 0.01335 | 0.02412 |  | 0.04484 | 0.04800 | 0.01313 |
| 614.882 | 0.01460 | 0.02508 |  | 0.04508 | 0.04647 | 0.01350 |
| 615.37 | 0.01411 | 0.02621 |  | 0.04501 | 0.04467 | 0.01390 |
| 615.859 | 0.01483 | 0.02643 |  | 0.04388 | 0.04413 | 0.01420 |
| 616.347 | 0.01511 | 0.02613 |  | 0.04282 | 0.04426 | 0.01433 |
| 616.835 | 0.01507 | 0.02536 |  | 0.04244 | 0.04511 | 0.01426 |
| 617.323 | 0.01507 | 0.02441 |  | 0.04238 | 0.04603 | 0.01404 |
| 617.812 | 0.01506 | 0.02456 |  | 0.04245 | 0.04686 | 0.01362 |
| 618.3 | 0.01507 | 0.02458 |  | 0.04262 | 0.04763 | 0.01305 |
| 618.788 | 0.01512 | 0.02462 |  | 0.04283 | 0.04823 | 0.01232 |
| 619.275 | 0.01517 | 0.02505 |  | 0.04307 | 0.04782 | 0.01156 |
| 619.763 | 0.01518 | 0.02540 |  | 0.04309 | 0.04732 | 0.01221 |
| 620.252 | 0.01515 | 0.02556 |  | 0.04315 | 0.04710 | 0.01225 |
| 620.74 | 0.01502 | 0.02532 |  | 0.04323 | 0.04740 | 0.01196 |
| 621.227 | 0.01469 | 0.02492 |  | 0.04336 | 0.04882 | 0.01164 |
| 621.715 | 0.01443 | 0.02463 |  | 0.04350 | 0.04813 | 0.01134 |
| 622.202 | 0.01421 | 0.02432 |  | 0.04371 | 0.04585 | 0.01122 |
| 622.689 | 0.01418 | 0.02509 |  | 0.04397 | 0.04533 | 0.01155 |
| 623.179 | 0.01426 | 0.02516 |  | 0.04423 | 0.04652 | 0.01192 |
| 623.666 | 0.01459 | 0.02517 |  | 0.04447 | 0.04964 | 0.01237 |
| 624.154 | 0.01505 | 0.02506 |  | 0.04445 | 0.04712 | 0.01292 |
| 624.641 | 0.01553 | 0.02469 |  | 0.04422 | 0.04544 | 0.01327 |
| 625.128 | 0.01596 | 0.02434 |  | 0.04353 | 0.04416 | 0.01297 |
| 625.615 | 0.01601 | 0.02412 |  | 0.04289 | 0.04296 | 0.01267 |
| 626.103 | 0.01562 | 0.02393 |  | 0.04210 | 0.04255 | 0.01226 |
| 626.59 | 0.01517 | 0.02370 |  | 0.04127 | 0.04376 | 0.01184 |
| 627.079 | 0.01471 | 0.02353 |  | 0.04093 | 0.04450 | 0.01143 |
| 627.566 | 0.01441 | 0.02349 |  | 0.04083 | 0.04525 | 0.01154 |
| 628.053 | 0.01429 | 0.02361 |  | 0.04117 | 0.04614 | 0.01185 |
| 628.54 | 0.01444 | 0.02390 |  | 0.04151 | 0.04712 | 0.01225 |
| 629.027 | 0.01493 | 0.02436 |  | 0.04171 | 0.04798 | 0.01266 |
| 629.514 | 0.01561 | 0.02497 |  | 0.04193 | 0.04869 | 0.01298 |
| 630.001 | 0.01570 | 0.02574 |  | 0.04219 | 0.04920 | 0.01282 |
| 630.488 | 0.01563 | 0.02664 |  | 0.04250 | 0.04953 | 0.01213 |
| 630.975 | 0.01556 | 0.02700 |  | 0.04284 | 0.04955 | 0.01168 |
| 631.462 | 0.01539 | 0.02723 |  | 0.04331 | 0.04952 | 0.01167 |
| 631.949 | 0.01515 | 0.02737 |  | 0.04377 | 0.04939 | 0.01201 |
| 632.436 | 0.01490 | 0.02748 |  | 0.04420 | 0.04907 | 0.01275 |
| 632.923 | 0.01468 | 0.02737 |  | 0.04419 | 0.04864 | 0.01249 |
| 633.412 | 0.01461 | 0.02721 |  | 0.04322 | 0.04819 | 0.01187 |
| 633.898 | 0.01498 | 0.02683 |  | 0.04216 | 0.04768 | 0.01174 |
| 634.385 | 0.01554 | 0.02647 |  | 0.04132 | 0.04716 | 0.01181 |
| 634.872 | 0.01598 | 0.02613 |  | 0.04028 | 0.04658 | 0.01205 |
| 635.359 | 0.01595 | 0.02612 |  | 0.03979 | 0.04619 | 0.01242 |
| 635.845 | 0.01561 | 0.02545 |  | 0.04000 | 0.04500 | 0.01298 |
| 636.332 | 0.01527 | 0.02456 |  | 0.04068 | 0.04409 | 0.01360 |
| 636.819 | 0.01483 | 0.02378 |  | 0.04122 | 0.04355 | 0.01412 |
| 637.305 | 0.01387 | 0.02272 |  | 0.04172 | 0.04357 | 0.01440 |
| 637.792 | 0.01338 | 0.02263 |  | 0.04215 | 0.04678 | 0.01385 |
| 638.278 | 0.01348 | 0.02331 |  | 0.04262 | 0.04858 | 0.01307 |
| 638.765 | 0.01386 | 0.02420 |  | 0.04304 | 0.04712 | 0.01250 |
| 639.251 | 0.01432 | 0.02494 |  | 0.04344 | 0.04726 | 0.01239 |
| 639.738 | 0.01377 | 0.02536 |  | 0.04379 | 0.04726 | 0.01304 |
| 640.224 | 0.01284 | 0.02517 |  | 0.04408 | 0.04727 | 0.01351 |
| 640.709 | 0.01308 | 0.02543 |  | 0.04433 | 0.04711 | 0.01419 |
| 641.195 | 0.01311 | 0.02566 |  | 0.04451 | 0.04772 | 0.01441 |
| 641.682 | 0.01319 | 0.02557 |  | 0.04463 | 0.04858 | 0.01380 |
| 642.168 | 0.01348 | 0.02544 |  | 0.04466 | 0.04881 | 0.01303 |
| 642.654 | 0.01394 | 0.02655 |  | 0.04464 | 0.04848 | 0.01238 |
| 643.141 | 0.01391 | 0.02699 |  | 0.04452 | 0.04703 | 0.01191 |
| 643.627 | 0.01398 | 0.02629 |  | 0.04430 | 0.04609 | 0.01167 |
| 644.113 | 0.01425 | 0.02520 |  | 0.04397 | 0.04627 | 0.01158 |
| 644.599 | 0.01481 | 0.02433 |  | 0.04353 | 0.04628 | 0.01162 |
| 645.086 | 0.01534 | 0.02464 |  | 0.04305 | 0.04642 | 0.01181 |
| 645.572 | 0.01521 | 0.02531 |  | 0.04256 | 0.04667 | 0.01212 |
| 646.058 | 0.01509 | 0.02572 |  | 0.04222 | 0.04733 | 0.01252 |
| 646.544 | 0.01463 | 0.02657 |  | 0.04205 | 0.04786 | 0.01278 |
| 647.028 | 0.01415 | 0.02686 |  | 0.04197 | 0.04663 | 0.01209 |
| 647.514 | 0.01390 | 0.02715 |  | 0.04206 | 0.04591 | 0.01187 |
| 648 | 0.01446 | 0.02633 |  | 0.04236 | 0.04536 | 0.01171 |
| 648.486 | 0.01470 | 0.02562 |  | 0.04334 | 0.04491 | 0.01252 |
| 648.972 | 0.01480 | 0.02559 |  | 0.04505 | 0.04417 | 0.01305 |
| 649.458 | 0.01475 | 0.02565 |  | 0.04644 | 0.04406 | 0.01414 |
| 649.944 | 0.01469 | 0.02590 |  | 0.04662 | 0.04427 | 0.01459 |
| 650.43 | 0.01444 | 0.02603 |  | 0.04412 | 0.04466 | 0.01417 |
| 650.914 | 0.01420 | 0.02597 |  | 0.04292 | 0.04520 | 0.01355 |
| 651.4 | 0.01426 | 0.02558 |  | 0.04171 | 0.04589 | 0.01244 |
| 651.886 | 0.01423 | 0.02518 |  | 0.04069 | 0.04679 | 0.01156 |
| 652.371 | 0.01434 | 0.02536 |  | 0.04188 | 0.04783 | 0.01122 |
| 652.857 | 0.01465 | 0.02545 |  | 0.04210 | 0.04891 | 0.01116 |
| 653.343 | 0.01499 | 0.02572 |  | 0.04258 | 0.04997 | 0.01151 |
| 653.827 | 0.01518 | 0.02610 |  | 0.04347 | 0.05104 | 0.01158 |
| 654.312 | 0.01502 | 0.02660 |  | 0.04409 | 0.05202 | 0.01281 |
| 654.798 | 0.01459 | 0.02717 |  | 0.04420 | 0.05202 | 0.01323 |
| 655.284 | 0.01392 | 0.02783 |  | 0.04394 | 0.05277 | 0.01341 |
| 655.769 | 0.01319 | 0.02841 |  | 0.04337 | 0.05249 | 0.01272 |
| 656.253 | 0.01216 | 0.02813 |  | 0.04251 | 0.05179 | 0.01150 |
| 656.738 | 0.01177 | 0.02754 |  | 0.04286 | 0.05084 | 0.01142 |
| 657.224 | 0.01215 | 0.02515 |  | 0.04349 | 0.04975 | 0.01170 |
| 657.71 | 0.01264 | 0.02568 |  | 0.04411 | 0.04885 | 0.01196 |
| 658.193 | 0.01309 | 0.02691 |  | 0.04435 | 0.04805 | 0.01213 |
| 658.678 | 0.01354 | 0.02663 |  | 0.04294 | 0.04743 | 0.01239 |
| 659.164 | 0.01402 | 0.02595 |  | 0.04271 | 0.04708 | 0.01245 |
| 659.649 | 0.01450 | 0.02674 |  | 0.04247 | 0.04660 | 0.01255 |
| 660.133 | 0.01491 | 0.02767 |  | 0.04227 | 0.04625 | 0.01255 |
| 660.618 | 0.01529 | 0.02480 |  | 0.04200 | 0.04605 | 0.01198 |
| 661.103 | 0.01550 | 0.02491 |  | 0.04169 | 0.04621 | 0.01142 |
| 661.589 | 0.01556 | 0.02547 |  | 0.04145 | 0.04777 | 0.01115 |
| 662.072 | 0.01537 | 0.02474 |  | 0.04130 | 0.04922 | 0.01100 |
| 662.557 | 0.01485 | 0.02447 |  | 0.04123 | 0.05063 | 0.01092 |
| 663.042 | 0.01461 | 0.02446 |  | 0.04133 | 0.05174 | 0.01096 |
| 663.528 | 0.01458 | 0.02470 |  | 0.04153 | 0.05118 | 0.01181 |
| 664.011 | 0.01463 | 0.02514 |  | 0.04186 | 0.05077 | 0.01219 |
| 664.496 | 0.01481 | 0.02574 |  | 0.04231 | 0.05049 | 0.01200 |
| 664.981 | 0.01513 | 0.02644 |  | 0.04287 | 0.05037 | 0.01189 |
| 665.464 | 0.01562 | 0.02740 |  | 0.04353 | 0.05032 | 0.01171 |
| 665.949 | 0.01536 | 0.02834 |  | 0.04424 | 0.05125 | 0.01156 |
| 666.434 | 0.01517 | 0.02845 |  | 0.04502 | 0.05245 | 0.01158 |
| 666.917 | 0.01523 | 0.02830 |  | 0.04506 | 0.05365 | 0.01186 |
| 667.402 | 0.01555 | 0.02815 |  | 0.04614 | 0.05464 | 0.01245 |
| 667.887 | 0.01486 | 0.02769 |  | 0.04881 | 0.05532 | 0.01334 |
| 668.37 | 0.01464 | 0.02717 |  | 0.04901 | 0.05485 | 0.01448 |
| 668.855 | 0.01409 | 0.02742 |  | 0.04821 | 0.05428 | 0.01487 |
| 669.34 | 0.01374 | 0.02849 |  | 0.04680 | 0.05343 | 0.01500 |
| 669.823 | 0.01368 | 0.02960 |  | 0.04532 | 0.05253 | 0.01475 |
| 670.308 | 0.01406 | 0.03073 |  | 0.04492 | 0.05176 | 0.01421 |
| 670.791 | 0.01538 | 0.03184 |  | 0.04455 | 0.05101 | 0.01347 |
| 671.276 | 0.01653 | 0.03286 |  | 0.04440 | 0.05013 | 0.01249 |
| 671.76 | 0.01668 | 0.03239 |  | 0.04485 | 0.04955 | 0.01281 |
| 672.243 | 0.01680 | 0.03292 |  | 0.04551 | 0.04999 | 0.01312 |
| 672.728 | 0.01690 | 0.03345 |  | 0.04537 | 0.05173 | 0.01341 |
| 673.21 | 0.01541 | 0.03377 |  | 0.04516 | 0.05311 | 0.01299 |
| 673.695 | 0.01498 | 0.03398 |  | 0.04498 | 0.05439 | 0.01160 |
| 674.18 | 0.01468 | 0.03408 |  | 0.04538 | 0.05511 | 0.01202 |
| 674.662 | 0.01454 | 0.03412 |  | 0.04567 | 0.05571 | 0.01345 |
| 675.147 | 0.01448 | 0.03409 |  | 0.04587 | 0.05453 | 0.01534 |
| 675.629 | 0.01456 | 0.03394 |  | 0.04602 | 0.05352 | 0.01532 |
| 676.114 | 0.01475 | 0.03326 |  | 0.04626 | 0.05269 | 0.01478 |
| 676.596 | 0.01505 | 0.03234 |  | 0.04664 | 0.05203 | 0.01508 |
| 677.081 | 0.01543 | 0.03289 |  | 0.04745 | 0.05151 | 0.01433 |
| 677.565 | 0.01595 | 0.03497 |  | 0.04834 | 0.05181 | 0.01316 |
| 678.048 | 0.01657 | 0.03467 |  | 0.04928 | 0.05229 | 0.01147 |
| 678.532 | 0.01649 | 0.03361 |  | 0.04955 | 0.05296 | 0.00980 |
| 679.015 | 0.01642 | 0.03177 |  | 0.04968 | 0.05372 | 0.01157 |
| 679.499 | 0.01643 | 0.03024 |  | 0.04883 | 0.05452 | 0.01382 |
| 679.981 | 0.01653 | 0.02937 |  | 0.04774 | 0.05524 | 0.01337 |
| 680.466 | 0.01668 | 0.02918 |  | 0.04683 | 0.05545 | 0.01409 |
| 680.948 | 0.01690 | 0.03154 |  | 0.04615 | 0.05574 | 0.01439 |
| 681.432 | 0.01678 | 0.03106 |  | 0.04568 | 0.05593 | 0.01421 |
| 681.914 | 0.01664 | 0.03066 |  | 0.04539 | 0.05600 | 0.01428 |
| 682.399 | 0.01636 | 0.03035 |  | 0.04528 | 0.05592 | 0.01481 |
| 682.881 | 0.01620 | 0.03021 |  | 0.04532 | 0.05517 | 0.01526 |
| 683.365 | 0.01613 | 0.03115 |  | 0.04550 | 0.05408 | 0.01613 |
| 683.847 | 0.01614 | 0.03145 |  | 0.04578 | 0.05444 | 0.01721 |
| 684.331 | 0.01616 | 0.02985 |  | 0.04631 | 0.05449 | 0.01774 |
| 684.813 | 0.01618 | 0.02907 |  | 0.04683 | 0.05434 | 0.01780 |
| 685.297 | 0.01616 | 0.02863 |  | 0.04727 | 0.05509 | 0.01824 |
| 685.779 | 0.01605 | 0.02852 |  | 0.04762 | 0.05727 | 0.01844 |
| 686.263 | 0.01590 | 0.02869 |  | 0.04779 | 0.05632 | 0.01815 |
| 686.745 | 0.01576 | 0.02911 |  | 0.04756 | 0.05356 | 0.01773 |
| 687.227 | 0.01559 | 0.02975 |  | 0.04729 | 0.05092 | 0.01709 |
| 687.711 | 0.01542 | 0.02926 |  | 0.04679 | 0.05145 | 0.01641 |
| 688.193 | 0.01532 | 0.02856 |  | 0.04624 | 0.05625 | 0.01621 |
| 688.677 | 0.01526 | 0.02860 |  | 0.04568 | 0.05793 | 0.01619 |
| 689.159 | 0.01522 | 0.02906 |  | 0.04556 | 0.05831 | 0.01638 |
| 689.643 | 0.01523 | 0.02999 |  | 0.04565 | 0.05845 | 0.01699 |
| 690.124 | 0.01527 | 0.03103 |  | 0.04597 | 0.05864 | 0.01774 |
| 690.606 | 0.01553 | 0.03208 |  | 0.04717 | 0.05896 | 0.01846 |
| 691.09 | 0.01666 | 0.03305 |  | 0.04837 | 0.05854 | 0.01908 |
| 691.571 | 0.01750 | 0.03384 |  | 0.04958 | 0.05833 | 0.01970 |
| 692.053 | 0.01822 | 0.03465 |  | 0.05078 | 0.05842 | 0.02038 |
| 692.537 | 0.01916 | 0.03533 |  | 0.05174 | 0.05871 | 0.02119 |
| 693.018 | 0.01985 | 0.03594 |  | 0.05226 | 0.05908 | 0.02205 |
| 693.502 | 0.02002 | 0.03643 |  | 0.05253 | 0.05934 | 0.02291 |
| 693.984 | 0.01998 | 0.03677 |  | 0.05250 | 0.05965 | 0.02376 |
| 694.465 | 0.01971 | 0.03683 |  | 0.05219 | 0.06001 | 0.02450 |
| 694.949 | 0.01990 | 0.03622 |  | 0.05146 | 0.06046 | 0.02510 |
| 695.43 | 0.02039 | 0.03716 |  | 0.05266 | 0.06097 | 0.02553 |
| 695.912 | 0.02020 | 0.03802 |  | 0.05406 | 0.06152 | 0.02576 |
| 696.395 | 0.02005 | 0.03896 |  | 0.05552 | 0.06213 | 0.02586 |
| 696.877 | 0.02025 | 0.04004 |  | 0.05699 | 0.06272 | 0.02552 |
| 697.358 | 0.01991 | 0.04041 |  | 0.05829 | 0.06290 | 0.02494 |
| 697.841 | 0.01812 | 0.04013 |  | 0.05968 | 0.06262 | 0.02443 |
| 698.323 | 0.01848 | 0.03951 |  | 0.05940 | 0.05928 | 0.02380 |
| 698.804 | 0.01817 | 0.03854 |  | 0.05791 | 0.05697 | 0.02313 |
| 699.287 | 0.01810 | 0.03781 |  | 0.05495 | 0.05497 | 0.02240 |
| 699.769 | 0.01834 | 0.03808 |  | 0.05351 | 0.05340 | 0.02163 |
| 700.25 | 0.01829 | 0.03765 |  | 0.05279 | 0.05479 | 0.02033 |
| 700.731 | 0.01803 | 0.03630 |  | 0.05308 | 0.05385 | 0.01902 |
| 701.214 | 0.01765 | 0.03457 |  | 0.05556 | 0.05265 | 0.01773 |
| 701.695 | 0.01697 | 0.03275 |  | 0.05479 | 0.05157 | 0.01669 |
| 702.177 | 0.01620 | 0.03114 |  | 0.05193 | 0.04957 | 0.01597 |
| 702.66 | 0.01558 | 0.02980 |  | 0.04863 | 0.04849 | 0.01543 |
| 703.141 | 0.01518 | 0.02875 |  | 0.04684 | 0.04858 | 0.01529 |
| 703.622 | 0.01495 | 0.02783 |  | 0.04745 | 0.05037 | 0.01575 |
| 704.103 | 0.01490 | 0.02701 |  | 0.04695 | 0.05205 | 0.01672 |
| 704.586 | 0.01500 | 0.02636 |  | 0.04651 | 0.05333 | 0.01736 |
| 705.067 | 0.01528 | 0.02614 |  | 0.04653 | 0.05336 | 0.01749 |
| 705.548 | 0.01575 | 0.02655 |  | 0.04666 | 0.05264 | 0.01758 |
| 706.029 | 0.01630 | 0.02950 |  | 0.04684 | 0.04957 | 0.01689 |
| 706.51 | 0.01636 | 0.02619 |  | 0.04705 | 0.04818 | 0.01618 |
| 706.993 | 0.01625 | 0.02667 |  | 0.04740 | 0.04764 | 0.01536 |
| 707.473 | 0.01616 | 0.02651 |  | 0.04747 | 0.04827 | 0.01455 |
| 707.954 | 0.01592 | 0.02627 |  | 0.04673 | 0.04982 | 0.01387 |
| 708.435 | 0.01525 | 0.02600 |  | 0.04557 | 0.05169 | 0.01319 |
| 708.916 | 0.01434 | 0.02569 |  | 0.04425 | 0.05205 | 0.01256 |
| 709.399 | 0.01375 | 0.02544 |  | 0.04294 | 0.05085 | 0.01201 |
| 709.879 | 0.01284 | 0.02529 |  | 0.04204 | 0.04979 | 0.01155 |
| 710.36 | 0.01238 | 0.02508 |  | 0.04209 | 0.04900 | 0.01135 |
| 710.841 | 0.01227 | 0.02491 |  | 0.04265 | 0.04863 | 0.01192 |
| 711.321 | 0.01268 | 0.02475 |  | 0.04359 | 0.04780 | 0.01286 |
| 711.804 | 0.01342 | 0.02466 |  | 0.04495 | 0.04535 | 0.01327 |
| 712.285 | 0.01406 | 0.02468 |  | 0.04644 | 0.04347 | 0.01373 |
| 712.765 | 0.01409 | 0.02484 |  | 0.04712 | 0.04211 | 0.01415 |
| 713.246 | 0.01417 | 0.02449 |  | 0.04712 | 0.04176 | 0.01454 |
| 713.726 | 0.01444 | 0.02549 |  | 0.04656 | 0.04169 | 0.01422 |
| 714.207 | 0.01468 | 0.02582 |  | 0.04538 | 0.04189 | 0.01458 |
| 714.687 | 0.01525 | 0.02612 |  | 0.04511 | 0.04238 | 0.01514 |
| 715.168 | 0.01573 | 0.02634 |  | 0.04580 | 0.04326 | 0.01516 |
| 715.65 | 0.01531 | 0.02643 |  | 0.04552 | 0.04442 | 0.01489 |
| 716.131 | 0.01458 | 0.02639 |  | 0.04469 | 0.04516 | 0.01445 |
| 716.611 | 0.01378 | 0.02619 |  | 0.04352 | 0.04552 | 0.01392 |
| 717.091 | 0.01305 | 0.02586 |  | 0.04243 | 0.04453 | 0.01310 |
| 717.572 | 0.01293 | 0.02538 |  | 0.04144 | 0.04430 | 0.01269 |
| 718.052 | 0.01330 | 0.02486 |  | 0.04075 | 0.04455 | 0.01180 |
| 718.532 | 0.01357 | 0.02456 |  | 0.04079 | 0.04552 | 0.01110 |
| 719.013 | 0.01385 | 0.02444 |  | 0.04134 | 0.04649 | 0.01012 |
| 719.493 | 0.01419 | 0.02456 |  | 0.04243 | 0.04762 | 0.00957 |
| 719.973 | 0.01403 | 0.02474 |  | 0.04410 | 0.04672 | 0.00938 |
| 720.453 | 0.01339 | 0.02498 |  | 0.04498 | 0.04596 | 0.00958 |
| 720.933 | 0.01323 | 0.02517 |  | 0.04528 | 0.04542 | 0.01017 |
| 721.415 | 0.01326 | 0.02534 |  | 0.04493 | 0.04517 | 0.01208 |
| 721.895 | 0.01351 | 0.02537 |  | 0.04416 | 0.04538 | 0.01330 |
| 722.376 | 0.01368 | 0.02501 |  | 0.04331 | 0.04716 | 0.01346 |
| 722.856 | 0.01388 | 0.02464 |  | 0.04213 | 0.04621 | 0.01210 |
| 723.336 | 0.01409 | 0.02425 |  | 0.04088 | 0.04480 | 0.01096 |
| 723.816 | 0.01371 | 0.02385 |  | 0.03962 | 0.04383 | 0.01128 |
| 724.296 | 0.01343 | 0.02342 |  | 0.03893 | 0.04310 | 0.01172 |
| 724.776 | 0.01355 | 0.02327 |  | 0.03869 | 0.04322 | 0.01214 |
| 725.255 | 0.01390 | 0.02308 |  | 0.03894 | 0.04363 | 0.01253 |
| 725.735 | 0.01457 | 0.02284 |  | 0.03990 | 0.04427 | 0.01287 |
| 726.215 | 0.01535 | 0.02253 |  | 0.04059 | 0.04479 | 0.01319 |
| 726.695 | 0.01556 | 0.02221 |  | 0.04122 | 0.04257 | 0.01353 |
| 727.175 | 0.01565 | 0.02195 |  | 0.04122 | 0.04163 | 0.01306 |
| 727.655 | 0.01564 | 0.02201 |  | 0.04124 | 0.04117 | 0.01253 |
| 728.134 | 0.01561 | 0.02301 |  | 0.04105 | 0.04096 | 0.01182 |
| 728.614 | 0.01557 | 0.02455 |  | 0.04064 | 0.04181 | 0.01128 |
| 729.094 | 0.01548 | 0.02546 |  | 0.04011 | 0.04339 | 0.01090 |
| 729.572 | 0.01536 | 0.02600 |  | 0.03969 | 0.04504 | 0.01065 |
| 730.051 | 0.01479 | 0.02596 |  | 0.03955 | 0.04579 | 0.01057 |
| 730.531 | 0.01442 | 0.02572 |  | 0.03871 | 0.04635 | 0.01062 |
| 731.01 | 0.01437 | 0.02449 |  | 0.03940 | 0.04664 | 0.01086 |
| 731.49 | 0.01449 | 0.02366 |  | 0.04032 | 0.04678 | 0.01117 |
| 731.97 | 0.01415 | 0.02315 |  | 0.04117 | 0.04660 | 0.01066 |
| 732.449 | 0.01387 | 0.02297 |  | 0.04195 | 0.04633 | 0.01032 |
| 732.929 | 0.01382 | 0.02308 |  | 0.04237 | 0.04601 | 0.01011 |
| 733.408 | 0.01393 | 0.02342 |  | 0.04225 | 0.04577 | 0.01004 |
| 733.888 | 0.01425 | 0.02397 |  | 0.04151 | 0.04578 | 0.01017 |
| 734.367 | 0.01453 | 0.02468 |  | 0.04136 | 0.04580 | 0.01079 |
| 734.846 | 0.01427 | 0.02548 |  | 0.04110 | 0.04456 | 0.01155 |
| 735.326 | 0.01417 | 0.02645 |  | 0.04164 | 0.04411 | 0.01236 |
| 735.803 | 0.01409 | 0.02662 |  | 0.04317 | 0.04351 | 0.01262 |
| 736.283 | 0.01212 | 0.02635 |  | 0.04383 | 0.04303 | 0.01217 |
| 736.762 | 0.01243 | 0.02538 |  | 0.04442 | 0.04265 | 0.00954 |
| 737.241 | 0.01214 | 0.02422 |  | 0.04485 | 0.04231 | 0.01447 |
| 737.72 | 0.01608 | 0.02365 |  | 0.04508 | 0.04252 | 0.01342 |
| 738.2 | 0.01598 | 0.02320 |  | 0.04495 | 0.04316 | 0.01293 |
| 738.679 | 0.01544 | 0.02284 |  | 0.04464 | 0.04376 | 0.01232 |
| 739.156 | 0.01499 | 0.02260 |  | 0.04417 | 0.04428 | 0.01162 |
| 739.635 | 0.01464 | 0.02248 |  | 0.04369 | 0.04405 | 0.01119 |
| 740.114 | 0.01439 | 0.02246 |  | 0.04320 | 0.04388 | 0.01083 |
| 740.593 | 0.01434 | 0.02256 |  | 0.04276 | 0.04349 | 0.01090 |
| 741.072 | 0.01420 | 0.02276 |  | 0.04225 | 0.04292 | 0.01124 |
| 741.552 | 0.01325 | 0.02303 |  | 0.04181 | 0.04251 | 0.01226 |
| 742.029 | 0.01227 | 0.02329 |  | 0.04143 | 0.04162 | 0.01326 |
| 742.508 | 0.01229 | 0.02361 |  | 0.04116 | 0.04473 | 0.01165 |
| 742.987 | 0.01256 | 0.02386 |  | 0.04113 | 0.04780 | 0.00955 |
| 743.465 | 0.01280 | 0.02389 |  | 0.04153 | 0.04863 | 0.00920 |
| 743.944 | 0.01307 | 0.02357 |  | 0.04187 | 0.04834 | 0.00874 |
| 744.421 | 0.01346 | 0.02292 |  | 0.04225 | 0.04812 | 0.00853 |
| 744.9 | 0.01390 | 0.02234 |  | 0.04266 | 0.04806 | 0.00882 |
| 745.379 | 0.01450 | 0.02206 |  | 0.04278 | 0.04801 | 0.00996 |
| 745.858 | 0.01499 | 0.02183 |  | 0.04247 | 0.04776 | 0.01197 |
| 746.335 | 0.01543 | 0.02189 |  | 0.04193 | 0.04724 | 0.01235 |
| 746.813 | 0.01494 | 0.02253 |  | 0.04182 | 0.04682 | 0.01230 |
| 747.292 | 0.01388 | 0.02316 |  | 0.04176 | 0.04648 | 0.01230 |
| 747.771 | 0.01266 | 0.02297 |  | 0.04169 | 0.04623 | 0.01233 |
| 748.248 | 0.01356 | 0.02413 |  | 0.04178 | 0.04612 | 0.01243 |
| 748.726 | 0.01382 | 0.02454 |  | 0.04194 | 0.04612 | 0.01252 |
| 749.205 | 0.01408 | 0.02495 |  | 0.04215 | 0.04623 | 0.01263 |
| 749.684 | 0.01413 | 0.02536 |  | 0.04238 | 0.04640 | 0.01255 |
| 750.16 | 0.01361 | 0.02577 |  | 0.04261 | 0.04622 | 0.01182 |
| 750.639 | 0.01325 | 0.02602 |  | 0.04266 | 0.04518 | 0.01117 |
| 751.117 | 0.01297 | 0.02593 |  | 0.04278 | 0.04379 | 0.01113 |
| 751.594 | 0.01283 | 0.02559 |  | 0.04288 | 0.04330 | 0.01109 |
| 752.072 | 0.01282 | 0.02470 |  | 0.04298 | 0.04300 | 0.01109 |
| 752.551 | 0.01215 | 0.02411 |  | 0.04284 | 0.04304 | 0.01111 |
| 753.027 | 0.01233 | 0.02368 |  | 0.04215 | 0.04333 | 0.01117 |
| 753.506 | 0.01305 | 0.02343 |  | 0.04096 | 0.04386 | 0.01127 |
| 753.984 | 0.01319 | 0.02335 |  | 0.04017 | 0.04449 | 0.01141 |
| 754.46 | 0.01341 | 0.02352 |  | 0.04044 | 0.04531 | 0.01167 |
| 754.939 | 0.01305 | 0.02377 |  | 0.04039 | 0.04638 | 0.01152 |
| 755.417 | 0.01305 | 0.02386 |  | 0.04036 | 0.04754 | 0.01124 |
| 755.893 | 0.01301 | 0.02381 |  | 0.04038 | 0.04862 | 0.01097 |
| 756.372 | 0.01300 | 0.02357 |  | 0.04046 | 0.04894 | 0.01065 |
| 756.85 | 0.01299 | 0.02339 |  | 0.04056 | 0.04833 | 0.01115 |
| 757.326 | 0.01296 | 0.02369 |  | 0.04071 | 0.04682 | 0.01188 |
| 757.804 | 0.01300 | 0.02433 |  | 0.04090 | 0.04520 | 0.01236 |
| 758.282 | 0.01281 | 0.02442 |  | 0.04114 | 0.04360 | 0.01269 |
| 758.759 | 0.01222 | 0.02428 |  | 0.04142 | 0.04218 | 0.01285 |
| 759.237 | 0.01188 | 0.02373 |  | 0.04179 | 0.04161 | 0.01295 |
| 759.713 | 0.01223 | 0.02305 |  | 0.04228 | 0.04160 | 0.01265 |
| 760.191 | 0.01354 | 0.02293 |  | 0.04311 | 0.04209 | 0.01265 |
| 760.669 | 0.01447 | 0.02274 |  | 0.04389 | 0.04298 | 0.01270 |
| 761.145 | 0.01545 | 0.02268 |  | 0.04442 | 0.04418 | 0.01283 |
| 761.623 | 0.01502 | 0.02273 |  | 0.04460 | 0.04553 | 0.01300 |
| 762.099 | 0.01472 | 0.02284 |  | 0.04356 | 0.04619 | 0.01257 |
| 762.577 | 0.01477 | 0.02295 |  | 0.04245 | 0.04608 | 0.01230 |
| 763.053 | 0.01504 | 0.02269 |  | 0.04179 | 0.04615 | 0.01121 |
| 763.531 | 0.01499 | 0.02205 |  | 0.04158 | 0.04668 | 0.01052 |
| 764.009 | 0.01405 | 0.02284 |  | 0.04201 | 0.04768 | 0.01011 |
| 764.484 | 0.01354 | 0.02350 |  | 0.04302 | 0.04924 | 0.01037 |
| 764.962 | 0.01511 | 0.02407 |  | 0.04412 | 0.04991 | 0.01075 |
| 765.438 | 0.01413 | 0.02452 |  | 0.04489 | 0.04864 | 0.01135 |
| 765.916 | 0.01250 | 0.02492 |  | 0.04527 | 0.04833 | 0.01188 |
| 766.392 | 0.01197 | 0.02509 |  | 0.04533 | 0.04912 | 0.01162 |
| 766.869 | 0.01030 | 0.02500 |  | 0.04505 | 0.04900 | 0.01113 |
| 767.345 | 0.01522 | 0.02474 |  | 0.04453 | 0.04781 | 0.01136 |
| 767.823 | 0.01196 | 0.02437 |  | 0.04368 | 0.04685 | 0.01178 |
| 768.298 | 0.01124 | 0.02370 |  | 0.04254 | 0.04630 | 0.01238 |
| 768.776 | 0.01165 | 0.02314 |  | 0.04101 | 0.04587 | 0.01311 |
| 769.251 | 0.01282 | 0.02313 |  | 0.04010 | 0.04541 | 0.01321 |
| 769.729 | 0.01433 | 0.02291 |  | 0.04009 | 0.04509 | 0.01310 |
| 770.205 | 0.01454 | 0.02317 |  | 0.04040 | 0.04492 | 0.01283 |
| 770.682 | 0.01489 | 0.02368 |  | 0.04083 | 0.04495 | 0.01250 |
| 771.158 | 0.01518 | 0.02415 |  | 0.04141 | 0.04521 | 0.01215 |
| 771.635 | 0.01535 | 0.02438 |  | 0.04185 | 0.04565 | 0.01182 |
| 772.111 | 0.01537 | 0.02457 |  | 0.04246 | 0.04631 | 0.01169 |
| 772.588 | 0.01525 | 0.02458 |  | 0.04304 | 0.04716 | 0.01152 |
| 773.063 | 0.01502 | 0.02456 |  | 0.04350 | 0.04816 | 0.01144 |
| 773.539 | 0.01469 | 0.02450 |  | 0.04386 | 0.04914 | 0.01139 |
| 774.016 | 0.01439 | 0.02444 |  | 0.04376 | 0.04989 | 0.01143 |
| 774.491 | 0.01467 | 0.02435 |  | 0.04309 | 0.05043 | 0.01157 |
| 774.969 | 0.01486 | 0.02426 |  | 0.04216 | 0.05030 | 0.01187 |
| 775.444 | 0.01495 | 0.02421 |  | 0.04154 | 0.05013 | 0.01252 |
| 775.921 | 0.01470 | 0.02422 |  | 0.04130 | 0.04934 | 0.01324 |
| 776.396 | 0.01467 | 0.02432 |  | 0.04124 | 0.04862 | 0.01648 |
| 776.872 | 0.01528 | 0.02451 |  | 0.04133 | 0.04800 | 0.01564 |
| 777.349 | 0.01591 | 0.02478 |  | 0.04166 | 0.04830 | 0.01310 |
| 777.824 | 0.01569 | 0.02513 |  | 0.04216 | 0.04928 | 0.01386 |
| 778.301 | 0.01563 | 0.02558 |  | 0.04286 | 0.05045 | 0.01388 |
| 778.776 | 0.01571 | 0.02608 |  | 0.04360 | 0.05189 | 0.01416 |
| 779.251 | 0.01596 | 0.02655 |  | 0.04385 | 0.05257 | 0.01423 |
| 779.728 | 0.01627 | 0.02694 |  | 0.04366 | 0.05258 | 0.01408 |
| 780.203 | 0.01596 | 0.02699 |  | 0.04363 | 0.05173 | 0.01380 |
| 780.678 | 0.01535 | 0.02660 |  | 0.04381 | 0.05145 | 0.01341 |
| 781.155 | 0.01498 | 0.02522 |  | 0.04414 | 0.05097 | 0.01291 |
| 781.63 | 0.01484 | 0.02314 |  | 0.04457 | 0.05015 | 0.01234 |
| 782.105 | 0.01497 | 0.02108 |  | 0.04516 | 0.04939 | 0.01172 |
| 782.582 | 0.01540 | 0.02327 |  | 0.04589 | 0.04871 | 0.01181 |
| 783.057 | 0.01644 | 0.02310 |  | 0.04704 | 0.04822 | 0.01359 |
| 783.532 | 0.01695 | 0.02309 |  | 0.04807 | 0.04787 | 0.01547 |
| 784.008 | 0.01730 | 0.02328 |  | 0.04729 | 0.04767 | 0.01751 |
| 784.483 | 0.01707 | 0.02370 |  | 0.04617 | 0.04764 | 0.01518 |
| 784.958 | 0.01681 | 0.02437 |  | 0.04578 | 0.04774 | 0.01301 |
| 785.435 | 0.01654 | 0.02552 |  | 0.04526 | 0.04801 | 0.01202 |
| 785.909 | 0.01630 | 0.02630 |  | 0.04489 | 0.04846 | 0.01303 |
| 786.384 | 0.01647 | 0.02665 |  | 0.04483 | 0.04912 | 0.01490 |
| 786.861 | 0.01696 | 0.02622 |  | 0.04491 | 0.05008 | 0.01800 |
| 787.335 | 0.01789 | 0.02634 |  | 0.04528 | 0.05250 | 0.01235 |
| 787.81 | 0.01798 | 0.02702 |  | 0.04581 | 0.05340 | 0.01251 |
| 788.285 | 0.01808 | 0.02761 |  | 0.04654 | 0.05427 | 0.01273 |
| 788.761 | 0.01856 | 0.02803 |  | 0.04701 | 0.05343 | 0.01249 |
| 789.236 | 0.01615 | 0.02837 |  | 0.04638 | 0.05159 | 0.01697 |
| 789.71 | 0.02020 | 0.02867 |  | 0.04648 | 0.05157 | 0.01508 |
| 790.185 | 0.02103 | 0.02870 |  | 0.04671 | 0.05223 | 0.01411 |
| 790.661 | 0.02170 | 0.02867 |  | 0.04709 | 0.05286 | 0.01482 |
| 791.136 | 0.02229 | 0.02851 |  | 0.04772 | 0.05301 | 0.01619 |
| 791.61 | 0.02243 | 0.02824 |  | 0.04851 | 0.05313 | 0.01767 |
| 792.084 | 0.02240 | 0.02798 |  | 0.04964 | 0.05243 | 0.01835 |
| 792.561 | 0.02165 | 0.02781 |  | 0.05093 | 0.05095 | 0.01938 |
| 793.035 | 0.02131 | 0.02796 |  | 0.05232 | 0.04918 | 0.01851 |
| 793.509 | 0.02147 | 0.02820 |  | 0.05351 | 0.05356 | 0.01695 |
| 793.984 | 0.02300 | 0.02858 |  | 0.05211 | 0.05681 | 0.01552 |
| 794.458 | 0.02379 | 0.02910 |  | 0.04997 | 0.05792 | 0.01550 |
| 794.934 | 0.02484 | 0.02979 |  | 0.04887 | 0.05834 | 0.01654 |
| 795.409 | 0.02550 | 0.03021 |  | 0.04851 | 0.05875 | 0.01870 |
| 795.883 | 0.02573 | 0.03010 |  | 0.04850 | 0.05846 | 0.02130 |
| 796.357 | 0.02565 | 0.03037 |  | 0.04948 | 0.05782 | 0.02106 |
| 796.831 | 0.02558 | 0.02970 |  | 0.04994 | 0.05681 | 0.02142 |
| 797.305 | 0.02534 | 0.02889 |  | 0.05031 | 0.05544 | 0.02166 |
| 797.781 | 0.02494 | 0.02825 |  | 0.05071 | 0.05518 | 0.02169 |
| 798.255 | 0.02451 | 0.02811 |  | 0.05117 | 0.05499 | 0.02175 |
| 798.729 | 0.02421 | 0.02841 |  | 0.05165 | 0.05483 | 0.02189 |
| 799.203 | 0.02399 | 0.02914 |  | 0.05214 | 0.05472 | 0.02196 |
| 799.677 | 0.02386 | 0.03078 |  | 0.05265 | 0.05465 | 0.02192 |
| 800.151 | 0.02396 | 0.03099 |  | 0.05319 | 0.05285 | 0.02176 |
| 800.625 | 0.02408 | 0.03110 |  | 0.05374 | 0.05085 | 0.02145 |
| 801.099 | 0.02413 | 0.03140 |  | 0.05434 | 0.05343 | 0.02074 |
| 801.575 | 0.02425 | 0.03199 |  | 0.05439 | 0.05443 | 0.02085 |
| 802.049 | 0.02440 | 0.03265 |  | 0.05387 | 0.05494 | 0.02110 |
| 802.523 | 0.02466 | 0.03263 |  | 0.05394 | 0.05551 | 0.02126 |
| 802.997 | 0.02504 | 0.03208 |  | 0.05542 | 0.05625 | 0.02213 |
| 803.47 | 0.02577 | 0.03134 |  | 0.05702 | 0.05714 | 0.02305 |
| 803.944 | 0.02670 | 0.03072 |  | 0.05870 | 0.05871 | 0.02396 |
| 804.418 | 0.02775 | 0.03035 |  | 0.06043 | 0.05967 | 0.02463 |
| 804.892 | 0.02777 | 0.03022 |  | 0.06145 | 0.05882 | 0.02487 |
| 805.365 | 0.02734 | 0.03085 |  | 0.06186 | 0.05659 | 0.02489 |
| 805.839 | 0.02677 | 0.03169 |  | 0.06225 | 0.05525 | 0.02440 |
| 806.313 | 0.02742 | 0.03267 |  | 0.06270 | 0.05451 | 0.02387 |
| 806.786 | 0.02855 | 0.03322 |  | 0.06339 | 0.05429 | 0.02327 |
| 807.26 | 0.02873 | 0.03343 |  | 0.06420 | 0.05452 | 0.02202 |
| 807.733 | 0.02880 | 0.03339 |  | 0.06518 | 0.05515 | 0.02103 |
| 808.209 | 0.02828 | 0.03305 |  | 0.06567 | 0.05603 | 0.02037 |
| 808.682 | 0.02721 | 0.03244 |  | 0.06582 | 0.05635 | 0.01984 |
| 809.156 | 0.02646 | 0.03155 |  | 0.06559 | 0.05744 | 0.01929 |
| 809.629 | 0.02589 | 0.03045 |  | 0.06487 | 0.05584 | 0.01874 |
| 810.103 | 0.02523 | 0.02934 |  | 0.06381 | 0.05422 | 0.01831 |
| 810.576 | 0.02420 | 0.02894 |  | 0.06247 | 0.05274 | 0.01701 |
| 811.05 | 0.02335 | 0.02931 |  | 0.06099 | 0.05144 | 0.01684 |
| 811.523 | 0.02268 | 0.02961 |  | 0.05949 | 0.05071 | 0.01733 |
| 811.996 | 0.02215 | 0.02994 |  | 0.05798 | 0.05028 | 0.01769 |
| 812.47 | 0.02167 | 0.03025 |  | 0.05639 | 0.05020 | 0.01692 |
| 812.941 | 0.02115 | 0.02713 |  | 0.05480 | 0.05034 | 0.01610 |
| 813.414 | 0.02061 | 0.02598 |  | 0.05333 | 0.05094 | 0.01548 |
| 813.888 | 0.02010 | 0.02523 |  | 0.05220 | 0.05009 | 0.01510 |
| 814.361 | 0.01963 | 0.02483 |  | 0.05138 | 0.04918 | 0.01509 |
| 814.834 | 0.01922 | 0.02487 |  | 0.05050 | 0.04749 | 0.01516 |
| 815.307 | 0.01880 | 0.02509 |  | 0.04933 | 0.04644 | 0.01483 |
| 815.78 | 0.01851 | 0.02656 |  | 0.04836 | 0.04676 | 0.01444 |
| 816.253 | 0.01820 | 0.02744 |  | 0.04771 | 0.04820 | 0.01402 |
| 816.727 | 0.01792 | 0.02752 |  | 0.04716 | 0.04818 | 0.01367 |
| 817.2 | 0.01760 | 0.02715 |  | 0.04674 | 0.04760 | 0.01356 |
| 817.673 | 0.01726 | 0.02516 |  | 0.04647 | 0.04681 | 0.01356 |
| 818.146 | 0.01691 | 0.02458 |  | 0.04633 | 0.04618 | 0.01379 |
| 818.619 | 0.01656 | 0.02425 |  | 0.04640 | 0.04552 | 0.01111 |
| 819.092 | 0.01620 | 0.02403 |  | 0.04643 | 0.04463 | 0.01072 |
| 819.565 | 0.01590 | 0.02398 |  | 0.04615 | 0.04373 | 0.01132 |
| 820.036 | 0.01560 | 0.02410 |  | 0.04577 | 0.04420 | 0.01300 |
| 820.508 | 0.01530 | 0.02440 |  | 0.04528 | 0.04485 | 0.01382 |
| 820.981 | 0.01506 | 0.02513 |  | 0.04477 | 0.04552 | 0.01371 |
| 821.454 | 0.01487 | 0.02509 |  | 0.04444 | 0.04613 | 0.01351 |
| 821.927 | 0.01481 | 0.02470 |  | 0.04404 | 0.04584 | 0.01332 |
| 822.4 | 0.01479 | 0.02414 |  | 0.04364 | 0.04639 | 0.01306 |
| 822.873 | 0.01505 | 0.02356 |  | 0.04326 | 0.04638 | 0.01282 |
| 823.343 | 0.01548 | 0.02287 |  | 0.04289 | 0.04514 | 0.01261 |
| 823.816 | 0.01604 | 0.02240 |  | 0.04262 | 0.04365 | 0.01262 |
| 824.289 | 0.01641 | 0.02233 |  | 0.04234 | 0.04320 | 0.01296 |
| 824.761 | 0.01584 | 0.02270 |  | 0.04252 | 0.04398 | 0.01358 |
| 825.234 | 0.01520 | 0.02326 |  | 0.04261 | 0.04468 | 0.01439 |
| 825.707 | 0.01513 | 0.02402 |  | 0.04340 | 0.04516 | 0.01469 |
| 826.177 | 0.01488 | 0.02467 |  | 0.04399 | 0.04553 | 0.01449 |
| 826.65 | 0.01654 | 0.02508 |  | 0.04425 | 0.04586 | 0.01406 |
| 827.122 | 0.01639 | 0.02485 |  | 0.04430 | 0.04611 | 0.01359 |
| 827.595 | 0.01621 | 0.02447 |  | 0.04247 | 0.04590 | 0.01304 |
| 828.068 | 0.01599 | 0.02399 |  | 0.04137 | 0.04538 | 0.01243 |
| 828.538 | 0.01527 | 0.02355 |  | 0.04074 | 0.04450 | 0.01203 |
| 829.01 | 0.01502 | 0.02297 |  | 0.03974 | 0.04354 | 0.01187 |
| 829.483 | 0.01511 | 0.02264 |  | 0.04458 | 0.04271 | 0.01330 |
| 829.955 | 0.01493 | 0.02302 |  | 0.04367 | 0.04161 | 0.01416 |
| 830.428 | 0.01528 | 0.02399 |  | 0.04417 | 0.04084 | 0.01448 |
| 830.898 | 0.01617 | 0.02503 |  | 0.04412 | 0.04122 | 0.01407 |
| 831.37 | 0.01678 | 0.02588 |  | 0.04400 | 0.04275 | 0.01347 |
| 831.843 | 0.01727 | 0.02630 |  | 0.04359 | 0.04516 | 0.01309 |
| 832.315 | 0.01756 | 0.02647 |  | 0.04303 | 0.04713 | 0.01224 |
| 832.785 | 0.01773 | 0.02631 |  | 0.04259 | 0.04666 | 0.01146 |
| 833.258 | 0.01781 | 0.02547 |  | 0.04321 | 0.04114 | 0.01163 |
| 833.73 | 0.01766 | 0.02457 |  | 0.04615 | 0.04642 | 0.01237 |
| 834.2 | 0.01740 | 0.02372 |  | 0.04540 | 0.04821 | 0.01320 |
| 834.672 | 0.01687 | 0.02357 |  | 0.04241 | 0.04711 | 0.01356 |
| 835.144 | 0.01622 | 0.02363 |  | 0.04191 | 0.04553 | 0.01392 |
| 835.616 | 0.01548 | 0.02387 |  | 0.04211 | 0.04378 | 0.01397 |
| 836.086 | 0.01465 | 0.02425 |  | 0.04184 | 0.04240 | 0.01381 |
| 836.558 | 0.01450 | 0.02465 |  | 0.04243 | 0.04158 | 0.01363 |
| 837.031 | 0.01431 | 0.02490 |  | 0.04301 | 0.04123 | 0.01353 |
| 837.501 | 0.01438 | 0.02506 |  | 0.04358 | 0.04170 | 0.01345 |
| 837.973 | 0.01473 | 0.02502 |  | 0.04412 | 0.04309 | 0.01343 |
| 838.444 | 0.01511 | 0.02450 |  | 0.04440 | 0.04368 | 0.01341 |
| 838.914 | 0.01538 | 0.02409 |  | 0.04409 | 0.04369 | 0.01347 |
| 839.386 | 0.01528 | 0.02372 |  | 0.04383 | 0.04328 | 0.01355 |
| 839.858 | 0.01523 | 0.02369 |  | 0.04335 | 0.04285 | 0.01370 |
| 840.328 | 0.01524 | 0.02370 |  | 0.04292 | 0.04250 | 0.01361 |
| 840.8 | 0.01530 | 0.02379 |  | 0.04253 | 0.04227 | 0.01297 |
| 841.27 | 0.01540 | 0.02390 |  | 0.04236 | 0.04227 | 0.01261 |
| 841.742 | 0.01551 | 0.02403 |  | 0.04226 | 0.04190 | 0.01219 |
| 842.213 | 0.01568 | 0.02416 |  | 0.04215 | 0.04146 | 0.01196 |
| 842.683 | 0.01588 | 0.02348 |  | 0.04194 | 0.04096 | 0.01203 |
| 843.155 | 0.01622 | 0.02664 |  | 0.04160 | 0.04037 | 0.01234 |
| 843.626 | 0.01609 | 0.02380 |  | 0.04120 | 0.03971 | 0.01264 |
| 844.096 | 0.01590 | 0.02314 |  | 0.04073 | 0.03871 | 0.01317 |
| 844.568 | 0.01533 | 0.02330 |  | 0.04035 | 0.03812 | 0.01382 |
| 845.037 | 0.01479 | 0.02316 |  | 0.04001 | 0.03919 | 0.01445 |
| 845.509 | 0.01435 | 0.02308 |  | 0.03988 | 0.04122 | 0.01475 |
| 845.979 | 0.01411 | 0.02307 |  | 0.04005 | 0.04443 | 0.01373 |
| 846.45 | 0.01404 | 0.02307 |  | 0.04026 | 0.04644 | 0.01222 |
| 846.922 | 0.01423 | 0.02304 |  | 0.04041 | 0.04738 | 0.01142 |
| 847.391 | 0.01485 | 0.02304 |  | 0.04025 | 0.04382 | 0.01106 |
| 847.863 | 0.01494 | 0.02305 |  | 0.03975 | 0.04160 | 0.01101 |
| 848.332 | 0.01485 | 0.02308 |  | 0.03893 | 0.04304 | 0.01146 |
| 848.804 | 0.01459 | 0.02312 |  | 0.03848 | 0.04355 | 0.01202 |
| 849.273 | 0.01398 | 0.02318 |  | 0.03922 | 0.04432 | 0.01249 |
| 849.744 | 0.01365 | 0.02331 |  | 0.04061 | 0.04468 | 0.01299 |
| 850.214 | 0.01373 | 0.02347 |  | 0.04231 | 0.04464 | 0.01305 |
| 850.685 | 0.01396 | 0.02354 |  | 0.04336 | 0.04421 | 0.01323 |
| 851.154 | 0.01435 | 0.02348 |  | 0.04392 | 0.04337 | 0.01345 |
| 851.626 | 0.01480 | 0.02308 |  | 0.04401 | 0.04215 | 0.01346 |
| 852.095 | 0.01506 | 0.02280 |  | 0.04358 | 0.04058 | 0.01328 |
| 852.566 | 0.01515 | 0.02274 |  | 0.04258 | 0.03892 | 0.01291 |
| 853.035 | 0.01416 | 0.02274 |  | 0.04144 | 0.04172 | 0.01235 |
| 853.506 | 0.01410 | 0.02281 |  | 0.04055 | 0.04219 | 0.01196 |
| 853.976 | 0.01400 | 0.02289 |  | 0.04012 | 0.04224 | 0.01172 |
| 854.447 | 0.01409 | 0.02302 |  | 0.04003 | 0.04193 | 0.01170 |
| 854.916 | 0.01436 | 0.02324 |  | 0.04016 | 0.04215 | 0.01202 |
| 855.387 | 0.01456 | 0.02338 |  | 0.04057 | 0.04238 | 0.01204 |
| 855.856 | 0.01467 | 0.02350 |  | 0.04130 | 0.04264 | 0.01159 |
| 856.327 | 0.01433 | 0.02344 |  | 0.04229 | 0.04285 | 0.01140 |
| 856.796 | 0.01392 | 0.02329 |  | 0.04352 | 0.04299 | 0.01127 |
| 857.267 | 0.01375 | 0.02343 |  | 0.04488 | 0.04276 | 0.01183 |
| 857.736 | 0.01380 | 0.02363 |  | 0.04582 | 0.04283 | 0.01257 |
| 858.205 | 0.01407 | 0.02393 |  | 0.04605 | 0.04287 | 0.01341 |
| 858.676 | 0.01454 | 0.02405 |  | 0.04467 | 0.04284 | 0.01330 |
| 859.145 | 0.01464 | 0.02405 |  | 0.04255 | 0.04276 | 0.01312 |
| 859.616 | 0.01456 | 0.02386 |  | 0.04018 | 0.04264 | 0.01300 |
| 860.084 | 0.01472 | 0.02396 |  | 0.03903 | 0.04249 | 0.01296 |
| 860.553 | 0.01498 | 0.02434 |  | 0.04010 | 0.04222 | 0.01266 |
| 861.024 | 0.01522 | 0.02462 |  | 0.04272 | 0.04218 | 0.01196 |
| 861.493 | 0.01545 | 0.02483 |  | 0.04070 | 0.04223 | 0.01097 |
| 861.964 | 0.01555 | 0.02495 |  | 0.03969 | 0.04243 | 0.01054 |
| 862.432 | 0.01557 | 0.02496 |  | 0.03936 | 0.04266 | 0.01039 |
| 862.901 | 0.01524 | 0.02492 |  | 0.03917 | 0.04237 | 0.01054 |
| 863.372 | 0.01454 | 0.02478 |  | 0.03915 | 0.04193 | 0.01101 |
| 863.84 | 0.01381 | 0.02456 |  | 0.03929 | 0.04122 | 0.01108 |
| 864.309 | 0.01257 | 0.02426 |  | 0.03958 | 0.04090 | 0.01080 |
| 864.78 | 0.01231 | 0.02392 |  | 0.03997 | 0.04164 | 0.01086 |
| 865.248 | 0.01318 | 0.02363 |  | 0.04044 | 0.04253 | 0.01120 |
| 865.717 | 0.01470 | 0.02325 |  | 0.04097 | 0.04356 | 0.01205 |
| 866.187 | 0.01466 | 0.02291 |  | 0.04148 | 0.04381 | 0.01300 |
| 866.656 | 0.01495 | 0.02228 |  | 0.04189 | 0.04370 | 0.01338 |
| 867.124 | 0.01523 | 0.02100 |  | 0.04191 | 0.04341 | 0.01349 |
| 867.595 | 0.01510 | 0.02069 |  | 0.04180 | 0.04241 | 0.01316 |
| 868.063 | 0.01503 | 0.02043 |  | 0.04177 | 0.04129 | 0.01245 |
| 868.531 | 0.01507 | 0.02093 |  | 0.04175 | 0.04063 | 0.01176 |
| 869.002 | 0.01516 | 0.02252 |  | 0.04169 | 0.04038 | 0.01118 |
| 869.47 | 0.01526 | 0.02351 |  | 0.04162 | 0.04084 | 0.01088 |
| 869.938 | 0.01522 | 0.02349 |  | 0.04154 | 0.04170 | 0.01081 |
| 870.407 | 0.01520 | 0.02237 |  | 0.04140 | 0.04238 | 0.01096 |
| 870.877 | 0.01498 | 0.02227 |  | 0.04122 | 0.04271 | 0.01128 |
| 871.345 | 0.01485 | 0.02218 |  | 0.04099 | 0.04297 | 0.01179 |
| 871.814 | 0.01475 | 0.02230 |  | 0.04065 | 0.04316 | 0.01244 |
| 872.282 | 0.01458 | 0.02277 |  | 0.04024 | 0.04321 | 0.01302 |
| 872.752 | 0.01441 | 0.02377 |  | 0.03971 | 0.04315 | 0.01256 |
| 873.22 | 0.01415 | 0.02514 |  | 0.03896 | 0.04286 | 0.01190 |
| 873.688 | 0.01450 | 0.02499 |  | 0.03811 | 0.04249 | 0.01111 |
| 874.156 | 0.01514 | 0.02484 |  | 0.03766 | 0.04225 | 0.01029 |
| 874.626 | 0.01531 | 0.02497 |  | 0.03793 | 0.04218 | 0.00958 |
| 875.094 | 0.01524 | 0.02487 |  | 0.03976 | 0.04269 | 0.01017 |
| 875.563 | 0.01494 | 0.02359 |  | 0.04287 | 0.04303 | 0.01155 |
| 876.031 | 0.01456 | 0.02267 |  | 0.04324 | 0.04323 | 0.01216 |
| 876.501 | 0.01433 | 0.02151 |  | 0.04047 | 0.04334 | 0.01249 |
| 876.969 | 0.01426 | 0.02148 |  | 0.03730 | 0.04315 | 0.01301 |
| 877.436 | 0.01434 | 0.02196 |  | 0.03545 | 0.04261 | 0.01185 |
| 877.904 | 0.01456 | 0.02267 |  | 0.04177 | 0.04117 | 0.01156 |
| 878.372 | 0.01489 | 0.02346 |  | 0.04597 | 0.04145 | 0.01132 |
| 878.84 | 0.01497 | 0.02426 |  | 0.04333 | 0.04263 | 0.01288 |
| 879.31 | 0.01495 | 0.02404 |  | 0.04221 | 0.04494 | 0.01278 |
| 879.778 | 0.01487 | 0.02344 |  | 0.04333 | 0.04662 | 0.01246 |
| 880.246 | 0.01480 | 0.02265 |  | 0.04430 | 0.04763 | 0.01186 |
| 880.713 | 0.01471 | 0.02161 |  | 0.04507 | 0.04744 | 0.01173 |
| 881.181 | 0.01529 | 0.02090 |  | 0.04560 | 0.04616 | 0.01138 |
| 881.649 | 0.01503 | 0.02129 |  | 0.04565 | 0.04429 | 0.01055 |
| 882.117 | 0.01473 | 0.02423 |  | 0.04544 | 0.04207 | 0.01010 |
| 882.586 | 0.01441 | 0.02526 |  | 0.04436 | 0.04105 | 0.01059 |
| 883.054 | 0.01427 | 0.02503 |  | 0.04340 | 0.04102 | 0.01106 |
| 883.521 | 0.01452 | 0.02482 |  | 0.04248 | 0.04189 | 0.01147 |
| 883.989 | 0.01439 | 0.02464 |  | 0.04179 | 0.04214 | 0.01185 |
| 884.457 | 0.01388 | 0.02470 |  | 0.04133 | 0.04295 | 0.01221 |
| 884.924 | 0.01354 | 0.02441 |  | 0.04127 | 0.04374 | 0.01267 |
| 885.392 | 0.01380 | 0.02318 |  | 0.04144 | 0.04462 | 0.01310 |
| 885.859 | 0.01414 | 0.02285 |  | 0.04187 | 0.04521 | 0.01248 |
| 886.327 | 0.01444 | 0.02322 |  | 0.04248 | 0.04556 | 0.01195 |
| 886.794 | 0.01478 | 0.02436 |  | 0.04319 | 0.04577 | 0.01169 |
| 887.264 | 0.01510 | 0.02518 |  | 0.04368 | 0.04585 | 0.01166 |
| 887.731 | 0.01533 | 0.02487 |  | 0.04246 | 0.04571 | 0.01188 |
| 888.198 | 0.01566 | 0.02478 |  | 0.04048 | 0.04542 | 0.01233 |
| 888.666 | 0.01597 | 0.02543 |  | 0.04004 | 0.04501 | 0.01276 |
| 889.133 | 0.01629 | 0.02497 |  | 0.03993 | 0.04455 | 0.01270 |
| 889.6 | 0.01643 | 0.02376 |  | 0.04072 | 0.04494 | 0.01257 |
| 890.068 | 0.01649 | 0.02271 |  | 0.04144 | 0.04536 | 0.01232 |
| 890.535 | 0.01621 | 0.02644 |  | 0.04198 | 0.04245 | 0.01210 |
| 891.002 | 0.01520 | 0.02661 |  | 0.04229 | 0.04149 | 0.01204 |
| 891.469 | 0.01411 | 0.02621 |  | 0.04234 | 0.04112 | 0.01209 |
| 891.937 | 0.01525 | 0.02547 |  | 0.04191 | 0.04131 | 0.01222 |
| 892.404 | 0.01539 | 0.02454 |  | 0.04126 | 0.04201 | 0.01245 |
| 892.871 | 0.01531 | 0.02339 |  | 0.03982 | 0.04314 | 0.01275 |
| 893.338 | 0.01482 | 0.02228 |  | 0.03868 | 0.04431 | 0.01311 |
| 893.805 | 0.01433 | 0.02154 |  | 0.03847 | 0.04413 | 0.01323 |
| 894.272 | 0.01402 | 0.02150 |  | 0.03885 | 0.04405 | 0.01263 |
| 894.739 | 0.01411 | 0.02179 |  | 0.03988 | 0.04440 | 0.01229 |
| 895.206 | 0.01450 | 0.02236 |  | 0.04000 | 0.04491 | 0.01238 |
| 895.673 | 0.01572 | 0.02307 |  | 0.04040 | 0.04609 | 0.01290 |
| 896.14 | 0.01599 | 0.02370 |  | 0.04090 | 0.04690 | 0.01359 |
| 896.607 | 0.01622 | 0.02399 |  | 0.04133 | 0.04585 | 0.01396 |
| 897.074 | 0.01603 | 0.02423 |  | 0.04162 | 0.04457 | 0.01419 |
| 897.541 | 0.01605 | 0.02396 |  | 0.04174 | 0.04315 | 0.01419 |
| 898.006 | 0.01533 | 0.02300 |  | 0.04151 | 0.04251 | 0.01412 |
| 898.473 | 0.01437 | 0.02126 |  | 0.04104 | 0.04394 | 0.01403 |
| 898.94 | 0.01381 | 0.02184 |  | 0.04034 | 0.04762 | 0.01387 |
| 899.406 | 0.01305 | 0.02310 |  | 0.03962 | 0.04270 | 0.01365 |
| 899.873 | 0.01252 | 0.02477 |  | 0.03906 | 0.04233 | 0.01341 |
| 900.34 | 0.01218 | 0.02593 |  | 0.03869 | 0.04188 | 0.01298 |
| 900.807 | 0.01207 | 0.02527 |  | 0.03852 | 0.04132 | 0.01259 |
| 901.273 | 0.01218 | 0.02460 |  | 0.03854 | 0.04075 | 0.01247 |
| 901.74 | 0.01250 | 0.02406 |  | 0.03894 | 0.04035 | 0.01260 |
| 902.207 | 0.01283 | 0.02353 |  | 0.03952 | 0.03958 | 0.01285 |
| 902.673 | 0.01340 | 0.02316 |  | 0.04033 | 0.03904 | 0.01305 |
| 903.138 | 0.01390 | 0.02274 |  | 0.04118 | 0.04049 | 0.01306 |
| 903.605 | 0.01438 | 0.02243 |  | 0.04175 | 0.04234 | 0.01246 |
| 904.071 | 0.01502 | 0.02223 |  | 0.04210 | 0.04281 | 0.01181 |
| 904.538 | 0.01465 | 0.02213 |  | 0.04194 | 0.04232 | 0.01135 |
| 905.004 | 0.01343 | 0.02212 |  | 0.04157 | 0.04111 | 0.01088 |
| 905.471 | 0.01277 | 0.02221 |  | 0.04100 | 0.04044 | 0.01167 |
| 905.935 | 0.01255 | 0.02234 |  | 0.04058 | 0.03959 | 0.01266 |
| 906.402 | 0.01278 | 0.02257 |  | 0.04091 | 0.03888 | 0.01305 |
| 906.868 | 0.01345 | 0.02274 |  | 0.04148 | 0.03841 | 0.01228 |
| 907.334 | 0.01352 | 0.02300 |  | 0.04224 | 0.03813 | 0.01183 |
| 907.801 | 0.01321 | 0.02323 |  | 0.04289 | 0.03810 | 0.01155 |
| 908.267 | 0.01304 | 0.02342 |  | 0.04332 | 0.03825 | 0.01137 |
| 908.732 | 0.01344 | 0.02332 |  | 0.04336 | 0.03897 | 0.01123 |
| 909.198 | 0.01389 | 0.02303 |  | 0.04308 | 0.03995 | 0.01116 |
| 909.664 | 0.01381 | 0.02254 |  | 0.04243 | 0.04087 | 0.01118 |
| 910.13 | 0.01391 | 0.02208 |  | 0.04171 | 0.04175 | 0.01130 |
| 910.596 | 0.01377 | 0.02221 |  | 0.04131 | 0.04134 | 0.01148 |
| 911.061 | 0.01369 | 0.02263 |  | 0.04099 | 0.04083 | 0.01170 |
| 911.527 | 0.01314 | 0.02228 |  | 0.04073 | 0.03937 | 0.01192 |
| 911.993 | 0.01334 | 0.02206 |  | 0.04055 | 0.03993 | 0.01198 |
| 912.459 | 0.01364 | 0.02175 |  | 0.04041 | 0.04112 | 0.01197 |
| 912.923 | 0.01400 | 0.02120 |  | 0.04031 | 0.04206 | 0.01175 |
| 913.389 | 0.01453 | 0.02111 |  | 0.04023 | 0.04287 | 0.01131 |
| 913.855 | 0.01474 | 0.02139 |  | 0.04017 | 0.04339 | 0.01053 |
| 914.321 | 0.01485 | 0.02225 |  | 0.04011 | 0.04357 | 0.00915 |
| 914.786 | 0.01468 | 0.02486 |  | 0.04007 | 0.04352 | 0.00885 |
| 915.252 | 0.01433 | 0.02555 |  | 0.04002 | 0.04294 | 0.00885 |
| 915.717 | 0.01393 | 0.02528 |  | 0.03971 | 0.04173 | 0.00931 |
| 916.181 | 0.01391 | 0.02303 |  | 0.03951 | 0.04029 | 0.01000 |
| 916.647 | 0.01384 | 0.02176 |  | 0.03932 | 0.03941 | 0.01049 |
| 917.113 | 0.01376 | 0.02185 |  | 0.03988 | 0.04082 | 0.01092 |
| 917.577 | 0.01368 | 0.02191 |  | 0.04100 | 0.04152 | 0.01112 |
| 918.043 | 0.01336 | 0.02239 |  | 0.04160 | 0.04273 | 0.01130 |
| 918.509 | 0.01359 | 0.02289 |  | 0.04204 | 0.03846 | 0.01139 |
| 918.973 | 0.01357 | 0.02341 |  | 0.04226 | 0.03788 | 0.01142 |
| 919.438 | 0.01359 | 0.02391 |  | 0.04212 | 0.03797 | 0.01138 |
| 919.904 | 0.01365 | 0.02386 |  | 0.04197 | 0.03847 | 0.01121 |
| 920.368 | 0.01375 | 0.02384 |  | 0.04142 | 0.03937 | 0.01105 |
| 920.833 | 0.01388 | 0.02356 |  | 0.04068 | 0.04064 | 0.01090 |
| 921.299 | 0.01404 | 0.02317 |  | 0.03986 | 0.04228 | 0.01099 |
| 921.763 | 0.01424 | 0.02276 |  | 0.03947 | 0.04135 | 0.01125 |
| 922.228 | 0.01448 | 0.02237 |  | 0.03958 | 0.04094 | 0.01158 |
| 922.694 | 0.01476 | 0.02213 |  | 0.03988 | 0.04096 | 0.01184 |
| 923.158 | 0.01503 | 0.02210 |  | 0.04058 | 0.04196 | 0.01057 |
| 923.623 | 0.01476 | 0.02211 |  | 0.04048 | 0.04308 | 0.01112 |
| 924.089 | 0.01427 | 0.02220 |  | 0.03994 | 0.04197 | 0.01154 |
| 924.552 | 0.01381 | 0.02233 |  | 0.03940 | 0.04133 | 0.01136 |
| 925.018 | 0.01352 | 0.02245 |  | 0.03879 | 0.04154 | 0.01118 |
| 925.481 | 0.01239 | 0.02247 |  | 0.03859 | 0.04205 | 0.01103 |
| 925.947 | 0.01165 | 0.02186 |  | 0.03841 | 0.04249 | 0.01094 |
| 926.41 | 0.01173 | 0.02115 |  | 0.03825 | 0.04267 | 0.01092 |
| 926.875 | 0.01330 | 0.02071 |  | 0.03816 | 0.04267 | 0.01102 |
| 927.341 | 0.01371 | 0.02057 |  | 0.03814 | 0.04253 | 0.01116 |
| 927.804 | 0.01434 | 0.02228 |  | 0.03807 | 0.04220 | 0.01124 |
| 928.269 | 0.01445 | 0.02328 |  | 0.03803 | 0.04161 | 0.01131 |
| 928.733 | 0.01345 | 0.02284 |  | 0.03826 | 0.04095 | 0.01129 |
| 929.198 | 0.01288 | 0.02233 |  | 0.03868 | 0.04032 | 0.01128 |
| 929.661 | 0.01234 | 0.02198 |  | 0.03934 | 0.04030 | 0.01130 |
| 930.127 | 0.01210 | 0.02193 |  | 0.04008 | 0.04117 | 0.01129 |
| 930.59 | 0.01230 | 0.02183 |  | 0.04064 | 0.04219 | 0.01132 |
| 931.055 | 0.01314 | 0.02177 |  | 0.04085 | 0.04217 | 0.01132 |
| 931.518 | 0.01348 | 0.02181 |  | 0.04070 | 0.04179 | 0.01133 |
| 931.983 | 0.01255 | 0.02189 |  | 0.04011 | 0.04107 | 0.01130 |
| 932.446 | 0.01271 | 0.02207 |  | 0.03729 | 0.04034 | 0.01122 |
| 932.911 | 0.01312 | 0.02230 |  | 0.03674 | 0.03980 | 0.01118 |
| 933.374 | 0.01334 | 0.02255 |  | 0.03720 | 0.03927 | 0.01121 |
| 933.839 | 0.01229 | 0.02279 |  | 0.03779 | 0.03877 | 0.01143 |
| 934.302 | 0.01139 | 0.02095 |  | 0.03860 | 0.03845 | 0.01190 |
| 934.767 | 0.01147 | 0.02104 |  | 0.03937 | 0.03844 | 0.01058 |
| 935.23 | 0.01180 | 0.02163 |  | 0.03976 | 0.03872 | 0.00974 |
| 935.695 | 0.01211 | 0.02234 |  | 0.03923 | 0.03922 | 0.01073 |
| 936.158 | 0.01244 | 0.02364 |  | 0.03857 | 0.03988 | 0.01145 |
| 936.623 | 0.01269 | 0.02484 |  | 0.03812 | 0.04007 | 0.01193 |
| 937.086 | 0.01267 | 0.02483 |  | 0.03820 | 0.04041 | 0.01227 |
| 937.551 | 0.01268 | 0.02455 |  | 0.03859 | 0.04061 | 0.01247 |
| 938.014 | 0.01201 | 0.02401 |  | 0.03880 | 0.04073 | 0.01209 |
| 938.477 | 0.01160 | 0.02326 |  | 0.03839 | 0.04076 | 0.01154 |
| 938.941 | 0.01290 | 0.02281 |  | 0.03776 | 0.04077 | 0.01090 |
| 939.404 | 0.01464 | 0.02307 |  | 0.03749 | 0.04077 | 0.01007 |
| 939.869 | 0.01431 | 0.02203 |  | 0.03722 | 0.04075 | 0.00910 |
| 940.331 | 0.01401 | 0.02239 |  | 0.03699 | 0.04084 | 0.00677 |
| 940.794 | 0.01379 | 0.02308 |  | 0.03683 | 0.04096 | 0.01160 |
| 941.259 | 0.01354 | 0.02299 |  | 0.03679 | 0.04066 | 0.01195 |
| 941.721 | 0.01324 | 0.02286 |  | 0.03689 | 0.03987 | 0.01039 |
| 942.186 | 0.01289 | 0.02271 |  | 0.03715 | 0.03746 | 0.00901 |
| 942.649 | 0.01253 | 0.02250 |  | 0.03751 | 0.03476 | 0.00825 |
| 943.111 | 0.01214 | 0.02130 |  | 0.03797 | 0.03310 | 0.00820 |
| 943.576 | 0.01172 | 0.02203 |  | 0.03841 | 0.03424 | 0.00831 |
| 944.038 | 0.01126 | 0.02181 |  | 0.03891 | 0.03879 | 0.00873 |
| 944.501 | 0.01080 | 0.02163 |  | 0.03945 | 0.03979 | 0.00938 |
| 944.965 | 0.01181 | 0.02143 |  | 0.03990 | 0.03859 | 0.01029 |
| 945.428 | 0.01221 | 0.02143 |  | 0.03999 | 0.03793 | 0.01125 |
| 945.89 | 0.01309 | 0.02162 |  | 0.03983 | 0.03759 | 0.01146 |
| 946.354 | 0.01380 | 0.02202 |  | 0.03938 | 0.03822 | 0.01084 |
| 946.817 | 0.01416 | 0.02207 |  | 0.03876 | 0.03874 | 0.01019 |
| 947.279 | 0.01357 | 0.02149 |  | 0.03896 | 0.03926 | 0.00953 |
| 947.743 | 0.01318 | 0.02081 |  | 0.03952 | 0.03972 | 0.00942 |
| 948.206 | 0.01302 | 0.02143 |  | 0.04016 | 0.04012 | 0.00992 |
| 948.668 | 0.01204 | 0.02156 |  | 0.04077 | 0.04030 | 0.01075 |
| 949.132 | 0.01154 | 0.02179 |  | 0.04135 | 0.03947 | 0.01021 |
| 949.594 | 0.01151 | 0.02243 |  | 0.04106 | 0.03882 | 0.00956 |
| 950.057 | 0.01176 | 0.02306 |  | 0.04037 | 0.03823 | 0.00918 |
| 950.519 | 0.01225 | 0.02305 |  | 0.03949 | 0.03775 | 0.00941 |
| 950.983 | 0.01246 | 0.02283 |  | 0.03841 | 0.03750 | 0.00954 |
| 951.445 | 0.01221 | 0.02233 |  | 0.03731 | 0.03733 | 0.00974 |
| 951.907 | 0.01192 | 0.02161 |  | 0.03665 | 0.03729 | 0.01002 |
| 952.369 | 0.01162 | 0.02078 |  | 0.03617 | 0.03735 | 0.00941 |
| 952.833 | 0.01166 | 0.01981 |  | 0.03588 | 0.03755 | 0.01034 |
| 953.295 | 0.01218 | 0.02047 |  | 0.03608 | 0.03771 | 0.01138 |
| 953.758 | 0.01264 | 0.02111 |  | 0.03649 | 0.03761 | 0.01208 |
| 954.219 | 0.01217 | 0.02195 |  | 0.03691 | 0.03777 | 0.01240 |
| 954.683 | 0.01242 | 0.02231 |  | 0.03672 | 0.03833 | 0.01255 |
| 955.145 | 0.01252 | 0.02182 |  | 0.03510 | 0.03901 | 0.01255 |
| 955.607 | 0.01308 | 0.02085 |  | 0.03528 | 0.03943 | 0.01244 |
| 956.069 | 0.01366 | 0.01945 |  | 0.03532 | 0.03956 | 0.01223 |
| 956.531 | 0.01411 | 0.01876 |  | 0.03633 | 0.03952 | 0.01188 |
| 956.995 | 0.01404 | 0.01974 |  | 0.03738 | 0.03939 | 0.01151 |
| 957.457 | 0.01357 | 0.02057 |  | 0.03805 | 0.03919 | 0.01115 |
| 957.919 | 0.01328 | 0.02144 |  | 0.03862 | 0.03896 | 0.01065 |
| 958.38 | 0.01314 | 0.02213 |  | 0.03888 | 0.03872 | 0.01002 |
| 958.842 | 0.01312 | 0.02269 |  | 0.03888 | 0.03848 | 0.01025 |
| 959.306 | 0.01319 | 0.02283 |  | 0.03851 | 0.03826 | 0.01016 |
| 959.768 | 0.01331 | 0.02297 |  | 0.03803 | 0.03818 | 0.01002 |
| 960.229 | 0.01330 | 0.02277 |  | 0.03755 | 0.03817 | 0.01023 |
| 960.691 | 0.01347 | 0.02246 |  | 0.03714 | 0.03825 | 0.01082 |
| 961.153 | 0.01363 | 0.02209 |  | 0.03739 | 0.03841 | 0.01186 |
| 961.614 | 0.01374 | 0.02186 |  | 0.03761 | 0.03871 | 0.01274 |
| 962.076 | 0.01379 | 0.02202 |  | 0.03789 | 0.03910 | 0.01325 |
| 962.537 | 0.01368 | 0.02228 |  | 0.03825 | 0.03959 | 0.01276 |
| 963.001 | 0.01359 | 0.02254 |  | 0.03834 | 0.04019 | 0.01190 |
| 963.462 | 0.01354 | 0.02271 |  | 0.03769 | 0.04088 | 0.01115 |
| 963.924 | 0.01350 | 0.02286 |  | 0.03694 | 0.04159 | 0.01051 |
| 964.385 | 0.01347 | 0.02282 |  | 0.03660 | 0.04213 | 0.00998 |
| 964.847 | 0.01329 | 0.02253 |  | 0.03667 | 0.04245 | 0.00948 |
| 965.308 | 0.01284 | 0.02218 |  | 0.03717 | 0.04245 | 0.00929 |
| 965.77 | 0.01274 | 0.02180 |  | 0.03776 | 0.04220 | 0.01014 |
| 966.231 | 0.01280 | 0.02158 |  | 0.03848 | 0.04175 | 0.01063 |
| 966.692 | 0.01274 | 0.02153 |  | 0.03918 | 0.04046 | 0.01035 |
| 967.154 | 0.01272 | 0.02181 |  | 0.03945 | 0.03964 | 0.01034 |
| 967.615 | 0.01274 | 0.02206 |  | 0.03887 | 0.03989 | 0.01054 |
| 968.076 | 0.01251 | 0.02228 |  | 0.03796 | 0.04064 | 0.01092 |
| 968.538 | 0.01244 | 0.02251 |  | 0.03734 | 0.04166 | 0.01139 |
| 969.001 | 0.01306 | 0.02274 |  | 0.03703 | 0.04303 | 0.01193 |
| 969.462 | 0.01314 | 0.02302 |  | 0.03695 | 0.04418 | 0.01247 |
| 969.923 | 0.01305 | 0.02329 |  | 0.03748 | 0.04514 | 0.01280 |
| 970.384 | 0.01296 | 0.02353 |  | 0.03792 | 0.04576 | 0.01298 |
| 970.845 | 0.01285 | 0.02371 |  | 0.03821 | 0.04527 | 0.01287 |
| 971.307 | 0.01268 | 0.02357 |  | 0.03845 | 0.04483 | 0.01259 |
| 971.768 | 0.01250 | 0.02347 |  | 0.03864 | 0.04464 | 0.01250 |
| 972.229 | 0.01241 | 0.02308 |  | 0.03875 | 0.04469 | 0.01262 |
| 972.69 | 0.01246 | 0.02258 |  | 0.03888 | 0.04487 | 0.01330 |
| 973.151 | 0.01270 | 0.02215 |  | 0.03873 | 0.04518 | 0.01305 |
| 973.612 | 0.01307 | 0.02180 |  | 0.03854 | 0.04534 | 0.01198 |
| 974.073 | 0.01309 | 0.02187 |  | 0.03840 | 0.04526 | 0.01129 |
| 974.532 | 0.01329 | 0.02201 |  | 0.03835 | 0.04480 | 0.01107 |
| 974.993 | 0.01348 | 0.02245 |  | 0.03902 | 0.04398 | 0.01109 |
| 975.454 | 0.01379 | 0.02296 |  | 0.03959 | 0.04293 | 0.01145 |
| 975.914 | 0.01417 | 0.02351 |  | 0.03973 | 0.04283 | 0.01209 |
| 976.375 | 0.01439 | 0.02387 |  | 0.03969 | 0.04296 | 0.01317 |
| 976.836 | 0.01361 | 0.02365 |  | 0.03937 | 0.04311 | 0.01425 |
| 977.297 | 0.01347 | 0.02343 |  | 0.03895 | 0.04334 | 0.01502 |
| 977.758 | 0.01368 | 0.02317 |  | 0.03851 | 0.04348 | 0.01213 |
| 978.219 | 0.01402 | 0.02293 |  | 0.03829 | 0.04343 | 0.00818 |
| 978.679 | 0.01390 | 0.02267 |  | 0.03886 | 0.04317 | 0.00844 |
| 979.14 | 0.01388 | 0.02243 |  | 0.03958 | 0.04266 | 0.00903 |
| 979.601 | 0.01401 | 0.02215 |  | 0.04030 | 0.04207 | 0.01157 |
| 980.061 | 0.01424 | 0.02187 |  | 0.04093 | 0.04143 | 0.01294 |
| 980.522 | 0.01437 | 0.02156 |  | 0.04126 | 0.04095 | 0.01172 |
| 980.981 | 0.01380 | 0.02128 |  | 0.04055 | 0.04129 | 0.01139 |
| 981.441 | 0.01327 | 0.02123 |  | 0.03943 | 0.04177 | 0.01249 |
| 981.902 | 0.01285 | 0.02146 |  | 0.03824 | 0.04235 | 0.01307 |
| 982.362 | 0.01286 | 0.02231 |  | 0.03716 | 0.04295 | 0.01322 |
| 982.823 | 0.01268 | 0.02310 |  | 0.03738 | 0.04343 | 0.01288 |
| 983.283 | 0.01253 | 0.02352 |  | 0.03859 | 0.04385 | 0.01214 |
| 983.744 | 0.01237 | 0.02361 |  | 0.04066 | 0.04343 | 0.01108 |
| 984.202 | 0.01298 | 0.02327 |  | 0.04251 | 0.04301 | 0.00986 |
| 984.663 | 0.01366 | 0.02285 |  | 0.04329 | 0.04252 | 0.00880 |
| 985.123 | 0.01355 | 0.02248 |  | 0.04287 | 0.04189 | 0.00822 |
| 985.584 | 0.01313 | 0.02228 |  | 0.04147 | 0.04126 | 0.00817 |
| 986.044 | 0.01292 | 0.02219 |  | 0.03963 | 0.04072 | 0.00879 |
| 986.504 | 0.01299 | 0.02202 |  | 0.03782 | 0.04045 | 0.00994 |
| 986.963 | 0.01345 | 0.02184 |  | 0.03641 | 0.04046 | 0.01237 |
| 987.423 | 0.01364 | 0.02164 |  | 0.03521 | 0.04074 | 0.01387 |
| 987.883 | 0.01292 | 0.02148 |  | 0.03421 | 0.04142 | 0.01337 |
| 988.343 | 0.01163 | 0.02137 |  | 0.03322 | 0.04252 | 0.01312 |
| 988.802 | 0.01140 | 0.02130 |  | 0.03422 | 0.04408 | 0.01282 |
| 989.262 | 0.01278 | 0.02134 |  | 0.03579 | 0.04489 | 0.01207 |
| 989.722 | 0.01286 | 0.02141 |  | 0.03717 | 0.04434 | 0.01162 |
| 990.182 | 0.01314 | 0.02151 |  | 0.03732 | 0.04358 | 0.01124 |
| 990.642 | 0.01315 | 0.02163 |  | 0.03713 | 0.04291 | 0.01095 |
| 991.101 | 0.01137 | 0.02174 |  | 0.03678 | 0.04271 | 0.01077 |
| 991.561 | 0.01271 | 0.02185 |  | 0.03641 | 0.04196 | 0.01069 |
| 992.021 | 0.01319 | 0.02189 |  | 0.03608 | 0.04208 | 0.01074 |
| 992.481 | 0.01321 | 0.02203 |  | 0.03592 | 0.04361 | 0.01091 |
| 992.939 | 0.01325 | 0.02222 |  | 0.03565 | 0.04288 | 0.01113 |
| 993.399 | 0.01332 | 0.02252 |  | 0.03541 | 0.04111 | 0.01138 |
| 993.859 | 0.01329 | 0.02237 |  | 0.03519 | 0.03971 | 0.00993 |
| 994.317 | 0.01277 | 0.02184 |  | 0.03513 | 0.03931 | 0.00882 |
| 994.777 | 0.01189 | 0.02144 |  | 0.03626 | 0.03926 | 0.00846 |
| 995.236 | 0.01109 | 0.02113 |  | 0.03737 | 0.03959 | 0.00963 |
| 995.694 | 0.01118 | 0.02096 |  | 0.03848 | 0.03964 | 0.01039 |
| 996.154 | 0.01183 | 0.02096 |  | 0.03906 | 0.03965 | 0.01074 |
| 996.614 | 0.01172 | 0.02119 |  | 0.03946 | 0.03997 | 0.01077 |
| 997.074 | 0.01137 | 0.02145 |  | 0.03931 | 0.04026 | 0.01097 |
| 997.532 | 0.01136 | 0.02167 |  | 0.03884 | 0.04062 | 0.01142 |
| 997.991 | 0.01154 | 0.02191 |  | 0.03801 | 0.04093 | 0.01174 |
| 998.451 | 0.01219 | 0.02158 |  | 0.03758 | 0.04095 | 0.01190 |
| 998.909 | 0.01263 | 0.02116 |  | 0.03724 | 0.04041 | 0.01195 |
| 999.369 | 0.01249 | 0.02069 |  | 0.03682 | 0.03838 | 0.01190 |
| 999.826 | 0.01238 | 0.02020 |  | 0.03652 | 0.03713 | 0.01159 |
| 1000.29 | 0.01204 | 0.01972 |  | 0.03623 | 0.03653 | 0.01108 |
| 1000.75 | 0.01191 | 0.01936 |  | 0.03610 | 0.03665 | 0.01043 |
| 1001.2 | 0.01180 | 0.01975 |  | 0.03615 | 0.03807 | 0.00974 |
| 1001.66 | 0.01204 | 0.01997 |  | 0.03640 | 0.03972 | 0.00908 |
| 1002.12 | 0.01166 | 0.02058 |  | 0.03666 | 0.04150 | 0.00846 |
| 1002.58 | 0.01115 | 0.02117 |  | 0.03698 | 0.04230 | 0.00886 |
| 1003.04 | 0.01098 | 0.02172 |  | 0.03733 | 0.04316 | 0.00922 |
| 1003.5 | 0.01088 | 0.02209 |  | 0.03777 | 0.04386 | 0.00967 |
| 1003.96 | 0.01075 | 0.02194 |  | 0.03825 | 0.04394 | 0.01010 |
| 1004.41 | 0.01044 | 0.02179 |  | 0.03865 | 0.04097 | 0.01040 |
| 1004.87 | 0.00985 | 0.02151 |  | 0.03899 | 0.03907 | 0.01065 |
| 1005.33 | 0.00912 | 0.02110 |  | 0.03892 | 0.03904 | 0.01080 |
| 1005.79 | 0.00862 | 0.02061 |  | 0.03835 | 0.04021 | 0.01087 |
| 1006.25 | 0.00950 | 0.02032 |  | 0.03777 | 0.04250 | 0.01084 |
| 1006.71 | 0.00992 | 0.02064 |  | 0.03709 | 0.04214 | 0.01078 |
| 1007.17 | 0.01038 | 0.02093 |  | 0.03640 | 0.04176 | 0.01056 |
| 1007.62 | 0.01100 | 0.02101 |  | 0.03609 | 0.04143 | 0.01031 |
| 1008.08 | 0.01161 | 0.02110 |  | 0.03651 | 0.03997 | 0.01032 |
| 1008.54 | 0.01216 | 0.02109 |  | 0.03712 | 0.03830 | 0.01007 |
| 1009 | 0.01203 | 0.02083 |  | 0.03728 | 0.03697 | 0.00980 |
| 1009.46 | 0.01163 | 0.02054 |  | 0.03732 | 0.03635 | 0.00953 |
| 1009.92 | 0.01122 | 0.02028 |  | 0.03737 | 0.03646 | 0.00928 |
| 1010.37 | 0.01064 | 0.02004 |  | 0.03746 | 0.03673 | 0.00862 |
| 1010.83 | 0.01032 | 0.01990 |  | 0.03763 | 0.03807 | 0.00828 |
| 1011.29 | 0.01058 | 0.01997 |  | 0.03782 | 0.03936 | 0.00881 |
| 1011.75 | 0.01080 | 0.02003 |  | 0.03785 | 0.04046 | 0.00979 |
| 1012.2 | 0.01114 | 0.02011 |  | 0.03776 | 0.04119 | 0.01039 |
| 1012.66 | 0.01133 | 0.02014 |  | 0.03757 | 0.04116 | 0.01007 |
| 1013.12 | 0.01149 | 0.02007 |  | 0.03738 | 0.04169 | 0.00985 |
| 1013.58 | 0.01160 | 0.02003 |  | 0.03727 | 0.04220 | 0.00937 |
| 1014.04 | 0.01172 | 0.02012 |  | 0.03727 | 0.04282 | 0.00908 |
| 1014.5 | 0.01185 | 0.02034 |  | 0.03744 | 0.04013 | 0.00893 |
| 1014.95 | 0.01193 | 0.02070 |  | 0.03780 | 0.03843 | 0.00884 |
| 1015.41 | 0.01189 | 0.02087 |  | 0.03789 | 0.03787 | 0.00881 |
| 1015.87 | 0.01179 | 0.02088 |  | 0.03780 | 0.03807 | 0.00887 |
| 1016.32 | 0.01169 | 0.02091 |  | 0.03761 | 0.03828 | 0.00907 |
| 1016.78 | 0.01167 | 0.02085 |  | 0.03745 | 0.03815 | 0.00929 |
| 1017.24 | 0.01195 | 0.02087 |  | 0.03741 | 0.03843 | 0.00941 |
| 1017.7 | 0.01222 | 0.02073 |  | 0.03751 | 0.03878 | 0.00946 |
| 1018.16 | 0.01154 | 0.02047 |  | 0.03774 | 0.03903 | 0.00923 |
| 1018.61 | 0.01099 | 0.02015 |  | 0.03811 | 0.03940 | 0.00915 |
| 1019.07 | 0.01083 | 0.01989 |  | 0.03837 | 0.03998 | 0.00944 |
| 1019.53 | 0.01089 | 0.01970 |  | 0.03838 | 0.04059 | 0.00981 |
| 1019.99 | 0.01116 | 0.01955 |  | 0.03816 | 0.04105 | 0.01011 |
| 1020.44 | 0.01152 | 0.01953 |  | 0.03786 | 0.04124 | 0.01026 |
| 1020.9 | 0.01178 | 0.01994 |  | 0.03733 | 0.04100 | 0.01023 |
| 1021.36 | 0.01147 | 0.02031 |  | 0.03673 | 0.04106 | 0.01012 |
| 1021.82 | 0.01129 | 0.02063 |  | 0.03609 | 0.04098 | 0.00991 |
| 1022.27 | 0.01119 | 0.02091 |  | 0.03554 | 0.04076 | 0.00958 |
| 1022.73 | 0.01114 | 0.02106 |  | 0.03521 | 0.04050 | 0.00902 |
| 1023.19 | 0.01115 | 0.02109 |  | 0.03507 | 0.04022 | 0.00813 |
| 1023.64 | 0.01108 | 0.02079 |  | 0.03524 | 0.03995 | 0.00760 |
| 1024.1 | 0.01108 | 0.02033 |  | 0.03554 | 0.03971 | 0.00802 |
| 1024.56 | 0.01098 | 0.01984 |  | 0.03594 | 0.03953 | 0.00902 |
| 1025.01 | 0.01096 | 0.01946 |  | 0.03620 | 0.03942 | 0.01046 |
| 1025.47 | 0.01101 | 0.01915 |  | 0.03632 | 0.03942 | 0.01140 |
| 1025.93 | 0.01115 | 0.01890 |  | 0.03626 | 0.03946 | 0.01176 |
| 1026.39 | 0.01145 | 0.01888 |  | 0.03606 | 0.03929 | 0.01168 |
| 1026.84 | 0.01187 | 0.01914 |  | 0.03559 | 0.03901 | 0.01098 |
| 1027.3 | 0.01204 | 0.01956 |  | 0.03476 | 0.03874 | 0.01024 |
| 1027.76 | 0.01159 | 0.02023 |  | 0.03394 | 0.03825 | 0.00954 |
| 1028.21 | 0.01102 | 0.02050 |  | 0.03328 | 0.03753 | 0.00895 |
| 1028.67 | 0.01098 | 0.02079 |  | 0.03310 | 0.03653 | 0.00892 |
| 1029.13 | 0.01115 | 0.02112 |  | 0.03381 | 0.03588 | 0.00953 |
| 1029.58 | 0.01139 | 0.02137 |  | 0.03466 | 0.03621 | 0.01050 |
| 1030.04 | 0.01161 | 0.02156 |  | 0.03550 | 0.03699 | 0.01111 |
| 1030.5 | 0.01178 | 0.02189 |  | 0.03643 | 0.03771 | 0.01115 |
| 1030.95 | 0.01184 | 0.02222 |  | 0.03719 | 0.03791 | 0.01055 |
| 1031.41 | 0.01168 | 0.02208 |  | 0.03749 | 0.03779 | 0.01053 |
| 1031.87 | 0.01147 | 0.02161 |  | 0.03729 | 0.03754 | 0.01061 |
| 1032.32 | 0.01129 | 0.02103 |  | 0.03619 | 0.03693 | 0.01035 |
| 1032.78 | 0.01149 | 0.02052 |  | 0.03503 | 0.03661 | 0.00992 |
| 1033.24 | 0.01215 | 0.02013 |  | 0.03399 | 0.03600 | 0.01015 |
| 1033.69 | 0.01282 | 0.01989 |  | 0.03317 | 0.03553 | 0.01061 |
| 1034.15 | 0.01252 | 0.01978 |  | 0.03280 | 0.03483 | 0.01116 |
| 1034.6 | 0.01257 | 0.01979 |  | 0.03277 | 0.03449 | 0.01110 |
| 1035.06 | 0.01215 | 0.01993 |  | 0.03289 | 0.03547 | 0.01060 |
| 1035.52 | 0.01166 | 0.02021 |  | 0.03302 | 0.03615 | 0.01026 |
| 1035.97 | 0.01197 | 0.02055 |  | 0.03325 | 0.03760 | 0.01002 |
| 1036.43 | 0.01256 | 0.02064 |  | 0.03369 | 0.03861 | 0.00955 |
| 1036.88 | 0.01295 | 0.02051 |  | 0.03417 | 0.03974 | 0.00823 |
| 1037.34 | 0.01226 | 0.01980 |  | 0.03458 | 0.04091 | 0.00991 |
| 1037.8 | 0.01193 | 0.01954 |  | 0.03485 | 0.04181 | 0.01090 |
| 1038.25 | 0.01207 | 0.01957 |  | 0.03502 | 0.04132 | 0.01182 |
| 1038.71 | 0.01206 | 0.01995 |  | 0.03508 | 0.04083 | 0.01220 |
| 1039.17 | 0.01208 | 0.02073 |  | 0.03510 | 0.04014 | 0.01214 |
| 1039.62 | 0.01191 | 0.02098 |  | 0.03504 | 0.03977 | 0.01184 |
| 1040.08 | 0.01197 | 0.02095 |  | 0.03492 | 0.03904 | 0.01149 |
| 1040.53 | 0.01233 | 0.01977 |  | 0.03469 | 0.04052 | 0.01114 |
| 1040.99 | 0.01269 | 0.01951 |  | 0.03417 | 0.04074 | 0.01116 |
| 1041.44 | 0.01250 | 0.01988 |  | 0.03334 | 0.04044 | 0.01142 |
| 1041.9 | 0.01237 | 0.02043 |  | 0.03261 | 0.04059 | 0.01202 |
| 1042.36 | 0.01232 | 0.02100 |  | 0.03222 | 0.04135 | 0.01236 |
| 1042.81 | 0.01235 | 0.02137 |  | 0.03214 | 0.04212 | 0.01244 |
| 1043.27 | 0.01196 | 0.02144 |  | 0.03283 | 0.04277 | 0.01238 |
| 1043.72 | 0.01154 | 0.02100 |  | 0.03313 | 0.04245 | 0.01255 |
| 1044.18 | 0.01141 | 0.02082 |  | 0.03345 | 0.04059 | 0.01275 |
| 1044.63 | 0.01150 | 0.02076 |  | 0.03372 | 0.03830 | 0.01286 |
| 1045.09 | 0.01151 | 0.01990 |  | 0.03398 | 0.03700 | 0.01284 |
| 1045.54 | 0.01152 | 0.01933 |  | 0.03381 | 0.03951 | 0.01267 |
| 1046 | 0.01149 | 0.01947 |  | 0.03353 | 0.04104 | 0.01381 |
| 1046.45 | 0.01141 | 0.01963 |  | 0.03319 | 0.04048 | 0.01374 |
| 1046.91 | 0.01142 | 0.01982 |  | 0.03365 | 0.04032 | 0.01333 |
| 1047.37 | 0.01168 | 0.02010 |  | 0.03466 | 0.04073 | 0.01284 |
| 1047.82 | 0.01236 | 0.02039 |  | 0.03472 | 0.04146 | 0.01227 |
| 1048.28 | 0.01315 | 0.02048 |  | 0.03416 | 0.04158 | 0.01049 |
| 1048.73 | 0.01312 | 0.02028 |  | 0.03346 | 0.04028 | 0.01036 |
| 1048.8 | 0.01310 | 0.02025 |  | 0.03333 | 0.04001 | 0.01042 |
| 1049.26 | 0.01285 | 0.01979 |  | 0.03243 | 0.03857 | 0.01108 |
| 1049.72 | 0.01282 | 0.01929 |  | 0.03162 | 0.03864 | 0.01229 |
| 1050.18 | 0.01294 | 0.01953 |  | 0.03104 | 0.03876 | 0.01260 |
| 1050.64 | 0.01283 | 0.01988 |  | 0.03139 | 0.03767 | 0.01292 |
| 1051.1 | 0.01275 | 0.02027 |  | 0.03207 | 0.03728 | 0.01306 |
| 1051.57 | 0.01262 | 0.02059 |  | 0.03293 | 0.03712 | 0.01203 |
| 1052.02 | 0.01284 | 0.02078 |  | 0.03359 | 0.03738 | 0.01190 |
| 1052.49 | 0.01328 | 0.02067 |  | 0.03365 | 0.03812 | 0.01168 |
| 1052.95 | 0.01381 | 0.02026 |  | 0.03344 | 0.03927 | 0.01142 |
| 1053.41 | 0.01436 | 0.01999 |  | 0.03311 | 0.03950 | 0.01144 |
| 1053.87 | 0.01446 | 0.01971 |  | 0.03260 | 0.03910 | 0.01108 |
| 1054.33 | 0.01480 | 0.01956 |  | 0.03186 | 0.03925 | 0.01069 |
| 1054.79 | 0.01430 | 0.01956 |  | 0.03098 | 0.03987 | 0.01041 |
| 1055.25 | 0.01437 | 0.01969 |  | 0.03098 | 0.04008 | 0.01039 |
| 1055.71 | 0.01476 | 0.01998 |  | 0.03202 | 0.03686 | 0.01068 |
| 1056.17 | 0.01535 | 0.02046 |  | 0.03279 | 0.03531 | 0.01123 |
| 1056.63 | 0.01598 | 0.02072 |  | 0.03308 | 0.04398 | 0.01148 |
| 1057.09 | 0.01643 | 0.02105 |  | 0.03327 | 0.04237 | 0.01137 |
| 1057.55 | 0.01658 | 0.02134 |  | 0.03347 | 0.04175 | 0.01144 |
| 1058.01 | 0.01590 | 0.02141 |  | 0.03365 | 0.04128 | 0.01169 |
| 1058.47 | 0.01538 | 0.02117 |  | 0.03382 | 0.04092 | 0.01211 |
| 1058.93 | 0.01510 | 0.02063 |  | 0.03397 | 0.04068 | 0.01230 |
| 1059.39 | 0.01649 | 0.02081 |  | 0.03413 | 0.04058 | 0.01347 |
| 1059.85 | 0.01605 | 0.02097 |  | 0.03429 | 0.04061 | 0.01446 |
| 1060.31 | 0.01554 | 0.02115 |  | 0.03449 | 0.04083 | 0.01549 |
| 1060.77 | 0.01523 | 0.02126 |  | 0.03470 | 0.04108 | 0.01622 |
| 1061.23 | 0.01509 | 0.02114 |  | 0.03495 | 0.04087 | 0.01640 |
| 1061.69 | 0.01571 | 0.02066 |  | 0.03524 | 0.03848 | 0.01664 |
| 1062.14 | 0.01689 | 0.02015 |  | 0.03558 | 0.03430 | 0.01636 |
| 1062.6 | 0.01783 | 0.01975 |  | 0.03602 | 0.04782 | 0.01579 |
| 1063.06 | 0.01875 | 0.01951 |  | 0.03654 | 0.04885 | 0.01521 |
| 1063.52 | 0.01926 | 0.01977 |  | 0.03711 | 0.04312 | 0.01460 |
| 1063.98 | 0.01970 | 0.02302 |  | 0.03771 | 0.03665 | 0.01540 |
| 1064.44 | 0.02146 | 0.02441 |  | 0.03805 | 0.04002 | 0.01560 |
| 1064.9 | 0.02141 | 0.02448 |  | 0.03758 | 0.04067 | 0.01598 |
| 1065.36 | 0.02096 | 0.02332 |  | 0.03668 | 0.04196 | 0.01653 |
| 1065.82 | 0.02066 | 0.02285 |  | 0.03609 | 0.04326 | 0.01697 |
| 1066.28 | 0.01997 | 0.02251 |  | 0.03563 | 0.04458 | 0.01661 |
| 1066.74 | 0.01939 | 0.02240 |  | 0.03551 | 0.04587 | 0.01623 |
| 1067.2 | 0.01897 | 0.02249 |  | 0.03576 | 0.04712 | 0.01646 |
| 1067.66 | 0.01856 | 0.02265 |  | 0.03660 | 0.04829 | 0.01690 |
| 1068.12 | 0.01790 | 0.02334 |  | 0.03732 | 0.04635 | 0.01742 |
| 1068.58 | 0.01733 | 0.02406 |  | 0.03799 | 0.04553 | 0.01771 |
| 1069.04 | 0.01716 | 0.02425 |  | 0.03856 | 0.04524 | 0.01725 |
| 1069.49 | 0.01788 | 0.02408 |  | 0.03899 | 0.04476 | 0.01669 |
| 1069.95 | 0.01893 | 0.02356 |  | 0.03934 | 0.04383 | 0.01602 |
| 1070.41 | 0.01855 | 0.02263 |  | 0.03959 | 0.04272 | 0.01547 |
| 1070.87 | 0.01819 | 0.02160 |  | 0.03965 | 0.04152 | 0.01509 |
| 1071.33 | 0.01802 | 0.02121 |  | 0.03937 | 0.04061 | 0.01489 |
| 1071.79 | 0.01791 | 0.02107 |  | 0.03866 | 0.04003 | 0.01501 |
| 1072.25 | 0.01802 | 0.02121 |  | 0.03757 | 0.04431 | 0.01499 |
| 1072.71 | 0.01847 | 0.02164 |  | 0.03591 | 0.04466 | 0.01504 |
| 1073.17 | 0.01889 | 0.02241 |  | 0.03504 | 0.04379 | 0.01480 |
| 1073.62 | 0.01902 | 0.02292 |  | 0.03435 | 0.04410 | 0.01452 |
| 1074.08 | 0.01910 | 0.02318 |  | 0.03473 | 0.04421 | 0.01420 |
| 1074.54 | 0.01911 | 0.02297 |  | 0.03573 | 0.04405 | 0.01391 |
| 1075 | 0.01905 | 0.02272 |  | 0.03669 | 0.04382 | 0.01373 |
| 1075.46 | 0.01888 | 0.02243 |  | 0.03722 | 0.04368 | 0.01373 |
| 1075.92 | 0.01891 | 0.02246 |  | 0.03575 | 0.04346 | 0.01412 |
| 1076.38 | 0.02048 | 0.02257 |  | 0.03494 | 0.04323 | 0.01454 |
| 1076.84 | 0.02278 | 0.02279 |  | 0.03492 | 0.04303 | 0.01511 |
| 1077.29 | 0.02382 | 0.02311 |  | 0.03539 | 0.04290 | 0.01610 |
| 1077.75 | 0.02443 | 0.02343 |  | 0.03603 | 0.04292 | 0.01634 |
| 1078.21 | 0.02564 | 0.02373 |  | 0.03687 | 0.04286 | 0.01618 |
| 1078.67 | 0.02710 | 0.02390 |  | 0.03797 | 0.04302 | 0.01513 |
| 1079.13 | 0.02884 | 0.02372 |  | 0.03936 | 0.04335 | 0.01432 |
| 1079.59 | 0.03089 | 0.02354 |  | 0.04103 | 0.04381 | 0.01380 |
| 1080.04 | 0.03335 | 0.02326 |  | 0.04289 | 0.04381 | 0.01346 |
| 1080.5 | 0.03662 | 0.02296 |  | 0.04488 | 0.04323 | 0.01346 |
| 1080.96 | 0.04013 | 0.02265 |  | 0.04669 | 0.04327 | 0.01577 |
| 1081.42 | 0.04177 | 0.02237 |  | 0.04727 | 0.04350 | 0.01692 |
| 1081.88 | 0.04261 | 0.02212 |  | 0.04727 | 0.04385 | 0.01768 |
| 1082.33 | 0.04271 | 0.02217 |  | 0.04604 | 0.04428 | 0.01764 |
| 1082.79 | 0.04215 | 0.02248 |  | 0.04465 | 0.04479 | 0.01750 |
| 1083.25 | 0.04096 | 0.02299 |  | 0.04349 | 0.04468 | 0.01736 |
| 1083.71 | 0.03917 | 0.02339 |  | 0.04253 | 0.04436 | 0.01736 |
| 1084.17 | 0.03669 | 0.02354 |  | 0.04177 | 0.04401 | 0.01728 |
| 1084.62 | 0.03328 | 0.02348 |  | 0.04120 | 0.04359 | 0.01632 |
| 1085.08 | 0.02991 | 0.02314 |  | 0.04081 | 0.04316 | 0.01512 |
| 1085.54 | 0.02704 | 0.02251 |  | 0.04063 | 0.04278 | 0.01381 |
| 1086 | 0.02510 | 0.02236 |  | 0.04052 | 0.04258 | 0.01248 |
| 1086.46 | 0.02393 | 0.02237 |  | 0.04008 | 0.04281 | 0.01196 |
| 1086.91 | 0.02325 | 0.02252 |  | 0.03936 | 0.04330 | 0.01151 |
| 1087.37 | 0.02241 | 0.02263 |  | 0.03660 | 0.04374 | 0.01124 |
| 1087.83 | 0.02141 | 0.02233 |  | 0.03551 | 0.04420 | 0.01084 |
| 1088.29 | 0.02012 | 0.02201 |  | 0.03477 | 0.04460 | 0.01064 |
| 1088.74 | 0.01872 | 0.02170 |  | 0.03458 | 0.04445 | 0.01070 |
| 1089.2 | 0.01746 | 0.02144 |  | 0.03486 | 0.04356 | 0.01125 |
| 1089.66 | 0.01643 | 0.02141 |  | 0.03550 | 0.04368 | 0.01113 |
| 1090.12 | 0.01589 | 0.02100 |  | 0.03667 | 0.04288 | 0.01108 |
| 1090.57 | 0.01585 | 0.02076 |  | 0.03726 | 0.04193 | 0.01115 |
| 1091.03 | 0.01574 | 0.02072 |  | 0.03747 | 0.04078 | 0.01132 |
| 1091.49 | 0.01446 | 0.02041 |  | 0.03701 | 0.03954 | 0.01158 |
| 1091.95 | 0.01392 | 0.02026 |  | 0.03589 | 0.03923 | 0.01178 |
| 1092.4 | 0.01395 | 0.02024 |  | 0.03518 | 0.03891 | 0.01181 |
| 1092.86 | 0.01403 | 0.02055 |  | 0.03483 | 0.04052 | 0.01187 |
| 1093.32 | 0.01416 | 0.02088 |  | 0.03465 | 0.04315 | 0.01197 |
| 1093.78 | 0.01417 | 0.02071 |  | 0.03451 | 0.04390 | 0.01186 |
| 1094.23 | 0.01374 | 0.02054 |  | 0.03445 | 0.04395 | 0.01156 |
| 1094.69 | 0.01355 | 0.02034 |  | 0.03447 | 0.04335 | 0.01118 |
| 1095.15 | 0.01385 | 0.02013 |  | 0.03464 | 0.04274 | 0.01073 |
| 1095.6 | 0.01374 | 0.01993 |  | 0.03464 | 0.04215 | 0.01019 |
| 1096.06 | 0.01363 | 0.02010 |  | 0.03433 | 0.04130 | 0.01001 |
| 1096.52 | 0.01375 | 0.02066 |  | 0.03376 | 0.04033 | 0.01106 |
| 1096.98 | 0.01403 | 0.02096 |  | 0.03335 | 0.03892 | 0.01098 |
| 1097.43 | 0.01438 | 0.02131 |  | 0.03302 | 0.03738 | 0.01028 |
| 1097.89 | 0.01456 | 0.02092 |  | 0.03266 | 0.03579 | 0.00990 |
| 1098.35 | 0.01415 | 0.02056 |  | 0.03224 | 0.03442 | 0.00998 |
| 1098.8 | 0.01364 | 0.02029 |  | 0.03173 | 0.03478 | 0.01050 |
| 1099.26 | 0.01302 | 0.02011 |  | 0.03126 | 0.03593 | 0.01139 |
| 1099.72 | 0.01253 | 0.02002 |  | 0.03082 | 0.03762 | 0.01227 |
| 1100.17 | 0.01245 | 0.02005 |  | 0.03049 | 0.03972 | 0.01236 |
| 1100.63 | 0.01260 | 0.02021 |  | 0.03040 | 0.04210 | 0.01151 |
| 1101.09 | 0.01285 | 0.02063 |  | 0.03034 | 0.04251 | 0.01074 |
| 1101.54 | 0.01220 | 0.02055 |  | 0.03028 | 0.04251 | 0.01015 |
| 1102 | 0.01133 | 0.02046 |  | 0.03078 | 0.04211 | 0.00977 |
| 1102.46 | 0.01171 | 0.02044 |  | 0.03160 | 0.04127 | 0.00993 |
| 1102.91 | 0.01235 | 0.01965 |  | 0.03250 | 0.03995 | 0.01014 |
| 1103.37 | 0.01331 | 0.01978 |  | 0.03337 | 0.03816 | 0.01067 |
| 1103.83 | 0.01314 | 0.01971 |  | 0.03401 | 0.03580 | 0.01055 |
| 1104.28 | 0.01300 | 0.01895 |  | 0.03442 | 0.03628 | 0.01005 |
| 1104.74 | 0.01308 | 0.01824 |  | 0.03429 | 0.03736 | 0.00969 |
| 1105.2 | 0.01308 | 0.01895 |  | 0.03342 | 0.04102 | 0.00936 |
| 1105.65 | 0.01251 | 0.01787 |  | 0.03172 | 0.04313 | 0.00884 |
| 1106.11 | 0.01208 | 0.01826 |  | 0.03210 | 0.04214 | 0.00900 |
| 1106.57 | 0.01178 | 0.01916 |  | 0.03347 | 0.03619 | 0.00927 |
| 1107.02 | 0.01163 | 0.01971 |  | 0.03436 | 0.04079 | 0.00962 |
| 1107.48 | 0.01151 | 0.01996 |  | 0.03486 | 0.04025 | 0.01005 |
| 1107.93 | 0.01144 | 0.02013 |  | 0.03496 | 0.03959 | 0.01056 |
| 1108.39 | 0.01145 | 0.02001 |  | 0.03470 | 0.03883 | 0.01112 |
| 1108.85 | 0.01153 | 0.02027 |  | 0.03408 | 0.03866 | 0.01091 |
| 1109.3 | 0.01167 | 0.02017 |  | 0.03317 | 0.03854 | 0.01076 |
| 1109.76 | 0.01181 | 0.01994 |  | 0.03191 | 0.03867 | 0.01040 |
| 1110.21 | 0.01198 | 0.01962 |  | 0.03041 | 0.03882 | 0.01007 |
| 1110.67 | 0.01222 | 0.01929 |  | 0.02981 | 0.03799 | 0.00983 |
| 1111.13 | 0.01249 | 0.01897 |  | 0.03019 | 0.03760 | 0.00971 |
| 1111.58 | 0.01260 | 0.01921 |  | 0.03084 | 0.03759 | 0.00949 |
| 1112.04 | 0.01209 | 0.01850 |  | 0.03148 | 0.03648 | 0.00950 |
| 1112.49 | 0.01155 | 0.01817 |  | 0.03227 | 0.03712 | 0.00993 |
| 1112.95 | 0.01106 | 0.01847 |  | 0.03311 | 0.03801 | 0.01084 |
| 1113.41 | 0.01086 | 0.01943 |  | 0.03352 | 0.03894 | 0.01175 |
| 1113.86 | 0.01079 | 0.01936 |  | 0.03353 | 0.04008 | 0.01248 |
| 1114.32 | 0.01078 | 0.01928 |  | 0.03318 | 0.04138 | 0.01195 |
| 1114.77 | 0.01082 | 0.01926 |  | 0.03279 | 0.04036 | 0.01143 |
| 1115.23 | 0.01094 | 0.01934 |  | 0.03257 | 0.03948 | 0.01099 |
| 1115.68 | 0.01113 | 0.01947 |  | 0.03244 | 0.03889 | 0.01055 |
| 1116.14 | 0.01141 | 0.01964 |  | 0.03244 | 0.03841 | 0.01025 |
| 1116.59 | 0.01177 | 0.01976 |  | 0.03250 | 0.03804 | 0.01015 |
| 1117.05 | 0.01221 | 0.01974 |  | 0.03257 | 0.03787 | 0.01026 |
| 1117.51 | 0.01268 | 0.01950 |  | 0.03265 | 0.03785 | 0.01054 |
| 1117.96 | 0.01279 | 0.01837 |  | 0.03263 | 0.03802 | 0.01098 |
| 1118.42 | 0.01269 | 0.01729 |  | 0.03252 | 0.03832 | 0.01158 |
| 1118.87 | 0.01218 | 0.01660 |  | 0.03226 | 0.03843 | 0.01163 |
| 1119.33 | 0.01149 | 0.01643 |  | 0.03202 | 0.03834 | 0.01122 |
| 1119.78 | 0.01122 | 0.01674 |  | 0.03269 | 0.03767 | 0.01063 |
| 1120.24 | 0.01131 | 0.01676 |  | 0.03378 | 0.03709 | 0.01002 |
| 1120.69 | 0.01168 | 0.01691 |  | 0.03433 | 0.03678 | 0.00941 |
| 1121.15 | 0.01182 | 0.01751 |  | 0.03465 | 0.03632 | 0.00895 |
| 1121.6 | 0.01197 | 0.01800 |  | 0.03448 | 0.03589 | 0.00886 |
| 1122.06 | 0.01196 | 0.01821 |  | 0.03409 | 0.03534 | 0.00912 |
| 1122.51 | 0.01201 | 0.01852 |  | 0.03301 | 0.03481 | 0.00990 |
| 1122.97 | 0.01197 | 0.01884 |  | 0.03182 | 0.03431 | 0.01037 |
| 1123.42 | 0.01198 | 0.01916 |  | 0.03059 | 0.03402 | 0.01069 |
| 1123.88 | 0.01217 | 0.01944 |  | 0.02946 | 0.03503 | 0.00960 |
| 1124.33 | 0.01244 | 0.01968 |  | 0.02942 | 0.03623 | 0.00859 |
| 1124.79 | 0.01218 | 0.01988 |  | 0.02941 | 0.03743 | 0.00793 |
| 1125.24 | 0.01139 | 0.01994 |  | 0.02968 | 0.03773 | 0.00790 |
| 1125.7 | 0.01109 | 0.01971 |  | 0.03032 | 0.03772 | 0.00837 |
| 1126.15 | 0.01207 | 0.01962 |  | 0.03125 | 0.03653 | 0.00963 |
| 1126.61 | 0.01256 | 0.01954 |  | 0.03246 | 0.03637 | 0.01034 |
| 1127.06 | 0.01248 | 0.01933 |  | 0.03362 | 0.03656 | 0.01070 |
| 1127.52 | 0.01214 | 0.01887 |  | 0.03386 | 0.03690 | 0.01105 |
| 1127.97 | 0.01173 | 0.01951 |  | 0.03296 | 0.03728 | 0.01137 |
| 1128.43 | 0.01142 | 0.01949 |  | 0.03254 | 0.03763 | 0.01166 |
| 1128.88 | 0.01120 | 0.01926 |  | 0.03237 | 0.03787 | 0.01191 |
| 1129.34 | 0.01126 | 0.01841 |  | 0.03229 | 0.03733 | 0.01162 |
| 1129.79 | 0.01165 | 0.01820 |  | 0.03177 | 0.03800 | 0.01043 |
| 1130.25 | 0.01196 | 0.01732 |  | 0.03230 | 0.03922 | 0.00954 |
| 1130.7 | 0.01220 | 0.01717 |  | 0.03230 | 0.03922 | 0.00926 |
| 1131.15 | 0.00556 | 0.01721 |  | 0.03168 | 0.03915 | 0.00917 |
| 1131.61 | 0.01166 | 0.01812 |  | 0.03050 | 0.03923 | 0.00943 |
| 1132.06 | 0.01014 | 0.01771 |  | 0.02889 | 0.03943 | 0.00985 |
| 1132.52 | 0.00969 | 0.01714 |  | 0.02837 | 0.03893 | 0.00982 |
| 1132.97 | 0.00979 | 0.01666 |  | 0.02935 | 0.03789 | 0.00997 |
| 1133.43 | 0.00969 | 0.01629 |  | 0.02983 | 0.03674 | 0.01013 |
| 1133.88 | 0.01063 | 0.01608 |  | 0.03066 | 0.03533 | 0.00990 |
| 1134.33 | 0.01119 | 0.01623 |  | 0.03179 | 0.03422 | 0.00985 |
| 1134.79 | 0.01152 | 0.01649 |  | 0.03281 | 0.03346 | 0.00984 |
| 1135.24 | 0.01172 | 0.01694 |  | 0.03349 | 0.03352 | 0.00985 |
| 1135.7 | 0.01131 | 0.01756 |  | 0.03366 | 0.03399 | 0.00991 |
| 1136.15 | 0.01058 | 0.01811 |  | 0.03338 | 0.03485 | 0.00993 |
| 1136.61 | 0.00979 | 0.01752 |  | 0.03211 | 0.03475 | 0.00955 |
| 1137.06 | 0.00949 | 0.01735 |  | 0.03108 | 0.03351 | 0.01028 |
| 1137.51 | 0.00965 | 0.01735 |  | 0.03032 | 0.03190 | 0.01040 |
| 1137.97 | 0.01039 | 0.01819 |  | 0.02907 | 0.03559 | 0.01063 |
| 1138.42 | 0.01085 | 0.01909 |  | 0.02886 | 0.03653 | 0.01091 |
| 1138.88 | 0.01118 | 0.01967 |  | 0.02962 | 0.03540 | 0.01122 |
| 1139.33 | 0.01153 | 0.01923 |  | 0.02977 | 0.03500 | 0.01151 |
| 1139.78 | 0.01127 | 0.01875 |  | 0.03048 | 0.03538 | 0.01178 |
| 1140.24 | 0.01085 | 0.01833 |  | 0.03137 | 0.03635 | 0.01201 |
| 1140.69 | 0.01036 | 0.01844 |  | 0.03161 | 0.03706 | 0.01218 |
| 1141.14 | 0.01006 | 0.01868 |  | 0.03122 | 0.03780 | 0.01238 |
| 1141.6 | 0.01050 | 0.01928 |  | 0.03021 | 0.03700 | 0.01221 |
| 1142.05 | 0.01105 | 0.01943 |  | 0.02868 | 0.03629 | 0.01205 |
| 1142.5 | 0.01152 | 0.01952 |  | 0.02694 | 0.03570 | 0.01222 |
| 1142.96 | 0.01117 | 0.01958 |  | 0.02505 | 0.03507 | 0.01180 |
| 1143.41 | 0.01097 | 0.01962 |  | 0.02456 | 0.03629 | 0.01079 |
| 1143.87 | 0.01080 | 0.01966 |  | 0.02549 | 0.03755 | 0.01016 |
| 1144.32 | 0.01071 | 0.01972 |  | 0.02783 | 0.03732 | 0.01067 |
| 1144.77 | 0.01092 | 0.01979 |  | 0.03025 | 0.03652 | 0.01136 |
| 1145.23 | 0.01134 | 0.02030 |  | 0.03219 | 0.03652 | 0.01221 |
| 1145.68 | 0.01198 | 0.02083 |  | 0.03281 | 0.03681 | 0.01271 |
| 1146.13 | 0.01232 | 0.02069 |  | 0.03292 | 0.03740 | 0.01211 |
| 1146.59 | 0.01254 | 0.01992 |  | 0.03266 | 0.03828 | 0.01151 |
| 1147.04 | 0.01256 | 0.01956 |  | 0.03248 | 0.03966 | 0.01095 |
| 1147.49 | 0.01198 | 0.01939 |  | 0.03226 | 0.03858 | 0.01094 |
| 1147.95 | 0.01147 | 0.01954 |  | 0.03258 | 0.03746 | 0.01097 |
| 1148.4 | 0.01110 | 0.01981 |  | 0.03301 | 0.03664 | 0.01147 |
| 1148.85 | 0.01136 | 0.02020 |  | 0.03347 | 0.03599 | 0.01251 |
| 1149.31 | 0.01187 | 0.02063 |  | 0.03398 | 0.03554 | 0.01273 |
| 1149.76 | 0.01231 | 0.02126 |  | 0.03441 | 0.03527 | 0.01217 |
| 1150.21 | 0.01250 | 0.02127 |  | 0.03471 | 0.03517 | 0.01310 |
| 1150.66 | 0.01263 | 0.02106 |  | 0.03493 | 0.03537 | 0.01406 |
| 1151.12 | 0.01274 | 0.02127 |  | 0.03507 | 0.03579 | 0.01453 |
| 1151.57 | 0.01232 | 0.02199 |  | 0.03517 | 0.03641 | 0.01464 |
| 1152.02 | 0.01156 | 0.02318 |  | 0.03548 | 0.03721 | 0.01564 |
| 1152.48 | 0.01296 | 0.02362 |  | 0.03635 | 0.03806 | 0.01612 |
| 1152.93 | 0.01310 | 0.02435 |  | 0.03773 | 0.03895 | 0.01598 |
| 1153.38 | 0.01322 | 0.02524 |  | 0.03723 | 0.03979 | 0.01562 |
| 1153.83 | 0.01339 | 0.02617 |  | 0.03730 | 0.04062 | 0.01547 |
| 1154.29 | 0.01360 | 0.02705 |  | 0.03752 | 0.04139 | 0.01529 |
| 1154.74 | 0.01398 | 0.02790 |  | 0.03802 | 0.04214 | 0.01519 |
| 1155.19 | 0.01478 | 0.02875 |  | 0.03910 | 0.04291 | 0.01558 |
| 1155.65 | 0.01572 | 0.02964 |  | 0.04052 | 0.04378 | 0.01591 |
| 1156.1 | 0.01577 | 0.03072 |  | 0.04231 | 0.04624 | 0.01632 |
| 1156.55 | 0.01588 | 0.03220 |  | 0.04443 | 0.04806 | 0.01763 |
| 1157 | 0.01611 | 0.03308 |  | 0.04688 | 0.04541 | 0.01962 |
| 1157.46 | 0.01645 | 0.03474 |  | 0.04953 | 0.04602 | 0.02320 |
| 1157.91 | 0.01683 | 0.03674 |  | 0.05066 | 0.04840 | 0.02531 |
| 1158.36 | 0.01721 | 0.03907 |  | 0.05132 | 0.05139 | 0.02671 |
| 1158.81 | 0.01773 | 0.03984 |  | 0.05152 | 0.05390 | 0.02792 |
| 1159.27 | 0.01779 | 0.04059 |  | 0.05068 | 0.05575 | 0.02869 |
| 1159.72 | 0.01687 | 0.04173 |  | 0.04992 | 0.05588 | 0.02917 |
| 1160.17 | 0.01641 | 0.04303 |  | 0.04982 | 0.05616 | 0.02972 |
| 1160.62 | 0.01617 | 0.04456 |  | 0.04986 | 0.05651 | 0.03022 |
| 1161.07 | 0.01635 | 0.04528 |  | 0.04980 | 0.05693 | 0.03076 |
| 1161.53 | 0.01679 | 0.04524 |  | 0.04963 | 0.05758 | 0.03116 |
| 1161.98 | 0.01685 | 0.04487 |  | 0.04927 | 0.05821 | 0.03133 |
| 1162.43 | 0.01671 | 0.04411 |  | 0.04875 | 0.05851 | 0.03060 |
| 1162.88 | 0.01658 | 0.04287 |  | 0.04816 | 0.05844 | 0.02950 |
| 1163.33 | 0.01646 | 0.04117 |  | 0.04730 | 0.05809 | 0.02838 |
| 1163.79 | 0.01623 | 0.03897 |  | 0.04587 | 0.05686 | 0.02727 |
| 1164.24 | 0.01572 | 0.03665 |  | 0.04387 | 0.05473 | 0.02659 |
| 1164.69 | 0.01603 | 0.03457 |  | 0.04213 | 0.05159 | 0.02580 |
| 1165.14 | 0.01574 | 0.03300 |  | 0.04065 | 0.04933 | 0.02505 |
| 1165.59 | 0.01544 | 0.03160 |  | 0.03946 | 0.04835 | 0.02433 |
| 1166.05 | 0.01518 | 0.03043 |  | 0.03851 | 0.04694 | 0.02331 |
| 1166.5 | 0.01493 | 0.02958 |  | 0.03784 | 0.04629 | 0.02231 |
| 1166.95 | 0.01380 | 0.02895 |  | 0.03740 | 0.04578 | 0.02140 |
| 1167.4 | 0.01311 | 0.02864 |  | 0.03769 | 0.04521 | 0.02053 |
| 1167.85 | 0.01264 | 0.02857 |  | 0.03701 | 0.04458 | 0.01976 |
| 1168.3 | 0.01221 | 0.02866 |  | 0.03562 | 0.04356 | 0.01905 |
| 1168.76 | 0.01208 | 0.02845 |  | 0.03484 | 0.04268 | 0.01848 |
| 1169.21 | 0.01214 | 0.02734 |  | 0.03452 | 0.04225 | 0.01804 |
| 1169.66 | 0.01176 | 0.02546 |  | 0.03451 | 0.04218 | 0.01786 |
| 1170.11 | 0.01134 | 0.02337 |  | 0.03453 | 0.04249 | 0.01739 |
| 1170.56 | 0.01166 | 0.02196 |  | 0.03442 | 0.04316 | 0.01566 |
| 1171.01 | 0.01205 | 0.02123 |  | 0.03424 | 0.04420 | 0.01391 |
| 1171.46 | 0.01262 | 0.02122 |  | 0.03401 | 0.04483 | 0.01292 |
| 1171.92 | 0.01283 | 0.02099 |  | 0.03380 | 0.04606 | 0.01275 |
| 1172.37 | 0.01286 | 0.02076 |  | 0.03343 | 0.04667 | 0.01341 |
| 1172.82 | 0.01289 | 0.02055 |  | 0.03304 | 0.04609 | 0.01382 |
| 1173.27 | 0.01290 | 0.02038 |  | 0.03258 | 0.04535 | 0.01396 |
| 1173.72 | 0.01265 | 0.02030 |  | 0.03227 | 0.04456 | 0.01370 |
| 1174.17 | 0.01237 | 0.02028 |  | 0.03249 | 0.04322 | 0.01352 |
| 1174.63 | 0.01200 | 0.02064 |  | 0.03286 | 0.04029 | 0.01319 |
| 1175.08 | 0.01199 | 0.02064 |  | 0.03310 | 0.03856 | 0.01284 |
| 1175.53 | 0.01192 | 0.02085 |  | 0.03307 | 0.04084 | 0.01264 |
| 1175.98 | 0.01190 | 0.02104 |  | 0.03241 | 0.04179 | 0.01253 |
| 1176.43 | 0.01189 | 0.02115 |  | 0.03190 | 0.04261 | 0.01249 |
| 1176.88 | 0.01193 | 0.02116 |  | 0.03089 | 0.04437 | 0.01238 |
| 1177.33 | 0.01176 | 0.02104 |  | 0.03009 | 0.04461 | 0.01219 |
| 1177.78 | 0.01137 | 0.02081 |  | 0.02954 | 0.04268 | 0.01194 |
| 1178.23 | 0.01115 | 0.02030 |  | 0.02932 | 0.04262 | 0.01164 |
| 1178.68 | 0.01126 | 0.01991 |  | 0.02978 | 0.04236 | 0.01125 |
| 1179.13 | 0.01129 | 0.01956 |  | 0.03012 | 0.04190 | 0.01077 |
| 1179.58 | 0.01149 | 0.01930 |  | 0.03018 | 0.04125 | 0.00987 |
| 1180.04 | 0.01133 | 0.01908 |  | 0.03017 | 0.04053 | 0.00994 |
| 1180.49 | 0.01083 | 0.01896 |  | 0.03018 | 0.03986 | 0.01096 |
| 1180.94 | 0.01050 | 0.01891 |  | 0.03020 | 0.03935 | 0.01115 |
| 1181.39 | 0.01011 | 0.01897 |  | 0.03019 | 0.03898 | 0.01096 |
| 1181.84 | 0.00947 | 0.01911 |  | 0.03017 | 0.03831 | 0.01097 |
| 1182.29 | 0.00996 | 0.01909 |  | 0.03003 | 0.03800 | 0.01097 |
| 1182.74 | 0.01048 | 0.01897 |  | 0.03160 | 0.03808 | 0.01105 |
| 1183.19 | 0.01076 | 0.01883 |  | 0.03363 | 0.03852 | 0.01098 |
| 1183.64 | 0.01095 | 0.01858 |  | 0.03296 | 0.03890 | 0.01097 |
| 1184.09 | 0.01113 | 0.01803 |  | 0.03224 | 0.03909 | 0.01032 |
| 1184.54 | 0.01120 | 0.01720 |  | 0.03261 | 0.03903 | 0.01034 |
| 1184.99 | 0.01120 | 0.01771 |  | 0.03310 | 0.03862 | 0.01061 |
| 1185.44 | 0.01112 | 0.01872 |  | 0.03360 | 0.03840 | 0.01132 |
| 1185.89 | 0.01099 | 0.01929 |  | 0.03376 | 0.03818 | 0.01223 |
| 1186.34 | 0.01080 | 0.01944 |  | 0.03366 | 0.03748 | 0.01275 |
| 1186.79 | 0.01101 | 0.01818 |  | 0.03331 | 0.03659 | 0.01267 |
| 1187.24 | 0.01102 | 0.01769 |  | 0.03278 | 0.03581 | 0.01166 |
| 1187.69 | 0.01099 | 0.01702 |  | 0.03195 | 0.03526 | 0.01059 |
| 1188.14 | 0.01094 | 0.01675 |  | 0.03137 | 0.03488 | 0.00980 |
| 1188.6 | 0.01088 | 0.01673 |  | 0.03114 | 0.03481 | 0.00921 |
| 1189.05 | 0.01060 | 0.01686 |  | 0.03120 | 0.03499 | 0.00923 |
| 1189.49 | 0.01028 | 0.01734 |  | 0.03166 | 0.03553 | 0.00937 |
| 1189.94 | 0.01004 | 0.01784 |  | 0.03238 | 0.03647 | 0.00955 |
| 1190.39 | 0.00993 | 0.01791 |  | 0.03240 | 0.03775 | 0.00990 |
| 1190.84 | 0.00992 | 0.01790 |  | 0.03046 | 0.03885 | 0.00926 |
| 1191.29 | 0.00996 | 0.01786 |  | 0.02906 | 0.03917 | 0.01019 |
| 1191.74 | 0.00998 | 0.01801 |  | 0.02813 | 0.03936 | 0.01198 |
| 1192.19 | 0.00997 | 0.01816 |  | 0.02753 | 0.03959 | 0.01257 |
| 1192.64 | 0.00995 | 0.01835 |  | 0.02797 | 0.03963 | 0.01133 |
| 1193.09 | 0.01021 | 0.01856 |  | 0.02871 | 0.03898 | 0.01029 |
| 1193.54 | 0.00974 | 0.01877 |  | 0.02943 | 0.03822 | 0.00860 |
| 1193.99 | 0.00917 | 0.01871 |  | 0.03029 | 0.03826 | 0.00837 |
| 1194.44 | 0.00892 | 0.01807 |  | 0.03104 | 0.03821 | 0.00904 |
| 1194.89 | 0.00901 | 0.01731 |  | 0.03162 | 0.03789 | 0.00980 |
| 1195.34 | 0.00944 | 0.01713 |  | 0.03201 | 0.03658 | 0.01043 |
| 1195.79 | 0.01020 | 0.01741 |  | 0.03223 | 0.03560 | 0.01049 |
| 1196.24 | 0.01131 | 0.01814 |  | 0.03229 | 0.03489 | 0.01057 |
| 1196.69 | 0.01088 | 0.01833 |  | 0.03215 | 0.03451 | 0.00924 |
| 1197.14 | 0.01015 | 0.01864 |  | 0.03180 | 0.03519 | 0.01005 |
| 1197.59 | 0.01009 | 0.01881 |  | 0.03127 | 0.03578 | 0.01014 |
| 1198.04 | 0.01105 | 0.01879 |  | 0.03063 | 0.03646 | 0.00953 |
| 1198.49 | 0.01148 | 0.01862 |  | 0.03025 | 0.03712 | 0.00966 |
| 1198.94 | 0.01088 | 0.01841 |  | 0.03046 | 0.03773 | 0.01024 |
| 1199.39 | 0.01079 | 0.01832 |  | 0.03078 | 0.03818 | 0.01109 |
| 1199.84 | 0.01068 | 0.01870 |  | 0.03116 | 0.03858 | 0.01110 |
| 1200.29 | 0.01068 | 0.01905 |  | 0.03145 | 0.03889 | 0.01007 |
| 1200.73 | 0.01079 | 0.01915 |  | 0.03010 | 0.03910 | 0.00901 |
| 1201.18 | 0.01096 | 0.01911 |  | 0.03344 | 0.03891 | 0.00898 |
| 1201.63 | 0.01112 | 0.01892 |  | 0.03608 | 0.03826 | 0.00955 |
| 1202.08 | 0.01132 | 0.01845 |  | 0.03683 | 0.03793 | 0.00996 |
| 1202.53 | 0.01072 | 0.01771 |  | 0.03410 | 0.03801 | 0.00964 |
| 1202.98 | 0.01039 | 0.01749 |  | 0.03269 | 0.03822 | 0.00945 |
| 1203.43 | 0.01069 | 0.01826 |  | 0.03142 | 0.03724 | 0.00933 |
| 1203.88 | 0.01095 | 0.01897 |  | 0.03177 | 0.03576 | 0.00929 |
| 1204.33 | 0.01045 | 0.01909 |  | 0.03088 | 0.03461 | 0.00937 |
| 1204.78 | 0.00979 | 0.01931 |  | 0.03015 | 0.03417 | 0.00960 |
| 1205.23 | 0.00999 | 0.01966 |  | 0.02971 | 0.03446 | 0.01006 |
| 1205.68 | 0.01046 | 0.02002 |  | 0.02943 | 0.03530 | 0.01036 |
| 1206.12 | 0.01145 | 0.02009 |  | 0.02967 | 0.03648 | 0.01062 |
| 1206.57 | 0.01184 | 0.01960 |  | 0.03020 | 0.03766 | 0.01032 |
| 1207.02 | 0.01164 | 0.01931 |  | 0.03109 | 0.03850 | 0.01013 |
| 1207.47 | 0.01132 | 0.01912 |  | 0.03192 | 0.03857 | 0.00979 |
| 1207.92 | 0.01092 | 0.01893 |  | 0.03217 | 0.03818 | 0.00754 |
| 1208.37 | 0.01050 | 0.01900 |  | 0.03195 | 0.03730 | 0.00816 |
| 1208.82 | 0.01010 | 0.01928 |  | 0.03181 | 0.03600 | 0.00920 |
| 1209.26 | 0.01006 | 0.01864 |  | 0.03216 | 0.03605 | 0.00987 |
| 1209.71 | 0.00990 | 0.01801 |  | 0.03166 | 0.03668 | 0.01054 |
| 1210.16 | 0.00985 | 0.01773 |  | 0.03104 | 0.03816 | 0.01094 |
| 1210.61 | 0.00984 | 0.01786 |  | 0.03062 | 0.03994 | 0.01046 |
| 1211.06 | 0.00994 | 0.01643 |  | 0.03081 | 0.04105 | 0.01027 |
| 1211.51 | 0.01017 | 0.01638 |  | 0.03089 | 0.04104 | 0.01051 |
| 1211.95 | 0.01055 | 0.01608 |  | 0.03129 | 0.04095 | 0.01066 |
| 1212.4 | 0.01075 | 0.01778 |  | 0.03204 | 0.04082 | 0.01093 |
| 1212.85 | 0.01054 | 0.01786 |  | 0.03304 | 0.04064 | 0.01082 |
| 1213.3 | 0.01135 | 0.01756 |  | 0.03426 | 0.04043 | 0.01022 |
| 1213.75 | 0.01194 | 0.01739 |  | 0.03561 | 0.04040 | 0.00970 |
| 1214.2 | 0.01230 | 0.01738 |  | 0.03424 | 0.03971 | 0.00938 |
| 1214.64 | 0.01104 | 0.01759 |  | 0.03146 | 0.03910 | 0.00902 |
| 1215.09 | 0.01142 | 0.01790 |  | 0.03107 | 0.03846 | 0.00878 |
| 1215.54 | 0.01155 | 0.01835 |  | 0.03178 | 0.03768 | 0.00863 |
| 1215.99 | 0.01103 | 0.01880 |  | 0.03196 | 0.03682 | 0.00859 |
| 1216.44 | 0.01053 | 0.01907 |  | 0.03184 | 0.03614 | 0.00868 |
| 1216.89 | 0.01020 | 0.01858 |  | 0.03131 | 0.03568 | 0.00894 |
| 1217.33 | 0.00987 | 0.01765 |  | 0.03083 | 0.03597 | 0.00945 |
| 1217.78 | 0.00964 | 0.01701 |  | 0.03081 | 0.03574 | 0.01027 |
| 1218.23 | 0.00946 | 0.01762 |  | 0.03216 | 0.03562 | 0.01000 |
| 1218.68 | 0.00938 | 0.01897 |  | 0.03322 | 0.03574 | 0.01015 |
| 1219.13 | 0.00949 | 0.01904 |  | 0.03316 | 0.03608 | 0.01049 |
| 1219.57 | 0.00988 | 0.01916 |  | 0.03273 | 0.03663 | 0.01086 |
| 1220.02 | 0.01022 | 0.01931 |  | 0.03127 | 0.03738 | 0.01124 |
| 1220.47 | 0.00980 | 0.01950 |  | 0.03131 | 0.03834 | 0.01160 |
| 1220.92 | 0.00949 | 0.01975 |  | 0.03142 | 0.03800 | 0.01166 |
| 1221.36 | 0.00930 | 0.02003 |  | 0.03149 | 0.03723 | 0.01175 |
| 1221.81 | 0.00944 | 0.01998 |  | 0.03150 | 0.03782 | 0.01171 |
| 1222.26 | 0.00994 | 0.01987 |  | 0.03141 | 0.03918 | 0.01136 |
| 1222.71 | 0.00970 | 0.01976 |  | 0.03125 | 0.04026 | 0.01081 |
| 1223.15 | 0.00998 | 0.01967 |  | 0.03043 | 0.04054 | 0.01081 |
| 1223.6 | 0.01056 | 0.01957 |  | 0.02958 | 0.04067 | 0.01161 |
| 1224.05 | 0.01110 | 0.01949 |  | 0.02871 | 0.03977 | 0.01215 |
| 1224.5 | 0.01109 | 0.01944 |  | 0.02798 | 0.03882 | 0.01109 |
| 1224.95 | 0.01060 | 0.01943 |  | 0.03041 | 0.03826 | 0.01002 |
| 1225.39 | 0.01200 | 0.01940 |  | 0.03323 | 0.03754 | 0.00962 |
| 1225.84 | 0.01212 | 0.01991 |  | 0.03459 | 0.03702 | 0.00990 |
| 1226.29 | 0.01216 | 0.02065 |  | 0.03508 | 0.03639 | 0.01092 |
| 1226.73 | 0.01166 | 0.02121 |  | 0.03531 | 0.03640 | 0.01270 |
| 1227.18 | 0.01079 | 0.02181 |  | 0.03541 | 0.03700 | 0.01345 |
| 1227.63 | 0.01014 | 0.02206 |  | 0.03539 | 0.03792 | 0.01397 |
| 1228.08 | 0.01001 | 0.02161 |  | 0.03525 | 0.03857 | 0.01425 |
| 1228.52 | 0.00996 | 0.02127 |  | 0.03501 | 0.03901 | 0.01397 |
| 1228.97 | 0.01006 | 0.02095 |  | 0.03464 | 0.03928 | 0.01263 |
| 1229.42 | 0.01027 | 0.02074 |  | 0.03418 | 0.03955 | 0.01258 |
| 1229.86 | 0.01049 | 0.02023 |  | 0.03318 | 0.03958 | 0.01267 |
| 1230.31 | 0.00998 | 0.01753 |  | 0.03233 | 0.03954 | 0.01403 |
| 1230.76 | 0.00951 | 0.02108 |  | 0.03208 | 0.03942 | 0.01359 |
| 1231.2 | 0.00918 | 0.02191 |  | 0.03118 | 0.03875 | 0.01298 |
| 1231.65 | 0.00934 | 0.02200 |  | 0.03061 | 0.03843 | 0.01115 |
| 1232.1 | 0.00996 | 0.02192 |  | 0.03053 | 0.03871 | 0.01131 |
| 1232.55 | 0.01101 | 0.02147 |  | 0.03061 | 0.03956 | 0.01152 |
| 1232.99 | 0.01192 | 0.02142 |  | 0.03082 | 0.03947 | 0.01124 |
| 1233.44 | 0.01211 | 0.02110 |  | 0.03075 | 0.03932 | 0.01176 |
| 1233.89 | 0.01136 | 0.02092 |  | 0.03100 | 0.03912 | 0.01206 |
| 1234.33 | 0.01116 | 0.02117 |  | 0.03122 | 0.03880 | 0.01214 |
| 1234.78 | 0.01112 | 0.02171 |  | 0.03152 | 0.03845 | 0.01209 |
| 1235.23 | 0.01119 | 0.02196 |  | 0.03177 | 0.03807 | 0.01211 |
| 1235.67 | 0.01117 | 0.02196 |  | 0.03206 | 0.03729 | 0.01230 |
| 1236.12 | 0.01049 | 0.02193 |  | 0.03222 | 0.03699 | 0.01161 |
| 1236.56 | 0.01003 | 0.02179 |  | 0.03298 | 0.03723 | 0.01142 |
| 1237.01 | 0.00973 | 0.02152 |  | 0.03349 | 0.03778 | 0.01151 |
| 1237.46 | 0.00898 | 0.02080 |  | 0.03382 | 0.03825 | 0.01170 |
| 1237.91 | 0.00954 | 0.01990 |  | 0.03364 | 0.03939 | 0.01223 |
| 1238.35 | 0.01015 | 0.01912 |  | 0.02973 | 0.03960 | 0.01197 |
| 1238.8 | 0.01047 | 0.01862 |  | 0.02742 | 0.03773 | 0.01175 |
| 1239.24 | 0.01086 | 0.01820 |  | 0.02547 | 0.03733 | 0.01156 |
| 1239.69 | 0.01132 | 0.01789 |  | 0.02784 | 0.03787 | 0.01180 |
| 1240.14 | 0.00976 | 0.01773 |  | 0.02874 | 0.03881 | 0.01219 |
| 1240.58 | 0.00941 | 0.01771 |  | 0.02967 | 0.03856 | 0.01266 |
| 1241.03 | 0.00979 | 0.01785 |  | 0.03055 | 0.03840 | 0.01251 |
| 1241.47 | 0.01010 | 0.01813 |  | 0.03124 | 0.03844 | 0.01208 |
| 1241.92 | 0.01054 | 0.01857 |  | 0.03137 | 0.03950 | 0.01190 |
| 1242.37 | 0.01102 | 0.01892 |  | 0.03127 | 0.04046 | 0.01176 |
| 1242.81 | 0.01112 | 0.01860 |  | 0.03090 | 0.04108 | 0.01101 |
| 1243.26 | 0.01071 | 0.01822 |  | 0.03036 | 0.04068 | 0.01017 |
| 1243.7 | 0.01045 | 0.01778 |  | 0.02974 | 0.03931 | 0.00989 |
| 1244.15 | 0.01062 | 0.01732 |  | 0.02899 | 0.03819 | 0.01022 |
| 1244.6 | 0.01056 | 0.01690 |  | 0.02850 | 0.03674 | 0.01110 |
| 1245.04 | 0.00994 | 0.01649 |  | 0.02839 | 0.03513 | 0.01199 |
| 1245.49 | 0.01000 | 0.01613 |  | 0.02887 | 0.03398 | 0.01196 |
| 1245.93 | 0.01012 | 0.01576 |  | 0.02985 | 0.03403 | 0.01177 |
| 1246.38 | 0.01007 | 0.01561 |  | 0.03129 | 0.03520 | 0.01154 |
| 1246.83 | 0.01007 | 0.01619 |  | 0.03315 | 0.03649 | 0.01112 |
| 1247.27 | 0.01015 | 0.01716 |  | 0.03281 | 0.03603 | 0.01043 |
| 1247.72 | 0.01028 | 0.01791 |  | 0.03057 | 0.03431 | 0.00954 |
| 1248.16 | 0.01041 | 0.01807 |  | 0.02954 | 0.03070 | 0.00869 |
| 1248.61 | 0.01056 | 0.01794 |  | 0.02880 | 0.03050 | 0.00886 |
| 1249.06 | 0.01071 | 0.01745 |  | 0.03093 | 0.03292 | 0.00947 |
| 1249.5 | 0.01006 | 0.01686 |  | 0.03178 | 0.03392 | 0.00931 |
| 1249.95 | 0.00969 | 0.01647 |  | 0.03123 | 0.03499 | 0.00882 |
| 1250.39 | 0.00959 | 0.01611 |  | 0.03072 | 0.03567 | 0.00820 |
| 1250.84 | 0.00959 | 0.01597 |  | 0.03052 | 0.03621 | 0.00744 |
| 1251.28 | 0.00997 | 0.01578 |  | 0.03059 | 0.03640 | 0.00659 |
| 1251.73 | 0.01050 | 0.01520 |  | 0.03095 | 0.03638 | 0.00590 |
| 1252.17 | 0.01067 | 0.01592 |  | 0.03140 | 0.03614 | 0.00603 |
| 1252.62 | 0.01054 | 0.01676 |  | 0.03176 | 0.03561 | 0.00680 |
| 1253.06 | 0.01067 | 0.01739 |  | 0.03101 | 0.03484 | 0.00782 |
| 1253.51 | 0.01111 | 0.01775 |  | 0.03158 | 0.03380 | 0.00862 |
| 1253.95 | 0.01108 | 0.01796 |  | 0.03134 | 0.03261 | 0.00922 |
| 1254.4 | 0.01094 | 0.01785 |  | 0.03063 | 0.03194 | 0.00931 |
| 1254.85 | 0.01068 | 0.01738 |  | 0.02945 | 0.03208 | 0.00884 |
| 1255.29 | 0.01036 | 0.01698 |  | 0.02794 | 0.03203 | 0.00884 |
| 1255.73 | 0.01000 | 0.01678 |  | 0.02660 | 0.03211 | 0.00865 |
| 1256.18 | 0.00960 | 0.01676 |  | 0.02565 | 0.03228 | 0.00844 |
| 1256.63 | 0.00930 | 0.01725 |  | 0.02512 | 0.03249 | 0.00840 |
| 1257.07 | 0.00902 | 0.01771 |  | 0.02493 | 0.03271 | 0.00855 |
| 1257.52 | 0.00894 | 0.01749 |  | 0.02507 | 0.03291 | 0.00886 |
| 1257.96 | 0.01019 | 0.01724 |  | 0.02558 | 0.03319 | 0.00906 |
| 1258.4 | 0.00893 | 0.01705 |  | 0.02642 | 0.03347 | 0.00865 |
| 1258.85 | 0.00917 | 0.01701 |  | 0.02756 | 0.03383 | 0.00850 |
| 1259.29 | 0.00985 | 0.01706 |  | 0.02877 | 0.03418 | 0.00831 |
| 1259.74 | 0.01029 | 0.01679 |  | 0.02954 | 0.03460 | 0.00818 |
| 1260.18 | 0.01067 | 0.01636 |  | 0.03039 | 0.03474 | 0.00818 |
| 1260.63 | 0.01026 | 0.01647 |  | 0.03109 | 0.03495 | 0.00811 |
| 1261.07 | 0.00973 | 0.01645 |  | 0.02990 | 0.03522 | 0.00773 |
| 1261.52 | 0.00929 | 0.01619 |  | 0.02813 | 0.03432 | 0.00740 |
| 1261.96 | 0.00895 | 0.01544 |  | 0.02585 | 0.03274 | 0.00722 |
| 1262.41 | 0.00875 | 0.01513 |  | 0.02465 | 0.03147 | 0.00718 |
| 1262.85 | 0.00867 | 0.01515 |  | 0.02472 | 0.03058 | 0.00724 |
| 1263.3 | 0.00871 | 0.01545 |  | 0.02737 | 0.03006 | 0.00728 |
| 1263.74 | 0.00888 | 0.01609 |  | 0.02921 | 0.02995 | 0.00733 |
| 1264.19 | 0.00918 | 0.01530 |  | 0.03122 | 0.03026 | 0.00736 |
| 1264.63 | 0.00958 | 0.01481 |  | 0.02950 | 0.03093 | 0.00730 |
| 1265.07 | 0.00988 | 0.01523 |  | 0.02919 | 0.03181 | 0.00722 |
| 1265.52 | 0.00974 | 0.01592 |  | 0.02884 | 0.03117 | 0.00712 |
| 1265.96 | 0.00965 | 0.01645 |  | 0.02849 | 0.03060 | 0.00697 |
| 1266.41 | 0.00958 | 0.01675 |  | 0.02815 | 0.03101 | 0.00680 |
| 1266.85 | 0.00964 | 0.01615 |  | 0.02792 | 0.03270 | 0.00653 |
| 1267.29 | 0.00991 | 0.01566 |  | 0.02831 | 0.03367 | 0.00600 |
| 1267.74 | 0.01012 | 0.01541 |  | 0.02849 | 0.03373 | 0.00592 |
| 1268.18 | 0.01034 | 0.01540 |  | 0.02767 | 0.03345 | 0.00616 |
| 1268.63 | 0.00976 | 0.01553 |  | 0.02686 | 0.03271 | 0.00705 |
| 1269.07 | 0.00879 | 0.01582 |  | 0.02632 | 0.03202 | 0.00750 |
| 1269.52 | 0.00854 | 0.01619 |  | 0.02592 | 0.03190 | 0.00805 |
| 1269.96 | 0.00798 | 0.01658 |  | 0.02553 | 0.03208 | 0.00765 |
| 1270.4 | 0.00787 | 0.01618 |  | 0.02531 | 0.03246 | 0.00712 |
| 1270.85 | 0.00967 | 0.01578 |  | 0.02519 | 0.03301 | 0.00651 |
| 1271.29 | 0.01044 | 0.01534 |  | 0.02516 | 0.03375 | 0.00591 |
| 1271.74 | 0.01082 | 0.01506 |  | 0.02522 | 0.03406 | 0.00581 |
| 1272.18 | 0.01068 | 0.01511 |  | 0.02535 | 0.03226 | 0.00674 |
| 1272.62 | 0.01073 | 0.01563 |  | 0.02491 | 0.03158 | 0.00685 |
| 1273.07 | 0.01047 | 0.01589 |  | 0.02769 | 0.03121 | 0.00629 |
| 1273.51 | 0.00997 | 0.01595 |  | 0.02944 | 0.03147 | 0.00732 |
| 1273.95 | 0.00918 | 0.01596 |  | 0.02902 | 0.03137 | 0.00748 |
| 1274.4 | 0.00901 | 0.01594 |  | 0.02581 | 0.03281 | 0.00755 |
| 1274.84 | 0.00945 | 0.01585 |  | 0.02592 | 0.03291 | 0.00751 |
| 1275.29 | 0.00913 | 0.01547 |  | 0.02552 | 0.03232 | 0.00746 |
| 1275.73 | 0.00926 | 0.01486 |  | 0.02563 | 0.03203 | 0.00745 |
| 1276.17 | 0.00937 | 0.01423 |  | 0.02563 | 0.03009 | 0.00751 |
| 1276.62 | 0.00943 | 0.01385 |  | 0.02559 | 0.02910 | 0.00786 |
| 1277.06 | 0.00951 | 0.01419 |  | 0.02580 | 0.02888 | 0.00821 |
| 1277.5 | 0.00963 | 0.01458 |  | 0.02626 | 0.02970 | 0.00856 |
| 1277.95 | 0.00983 | 0.01480 |  | 0.02691 | 0.03060 | 0.00872 |
| 1278.39 | 0.00962 | 0.01522 |  | 0.02785 | 0.02964 | 0.00875 |
| 1278.83 | 0.00951 | 0.01561 |  | 0.02888 | 0.02842 | 0.00872 |
| 1279.28 | 0.00964 | 0.01598 |  | 0.02933 | 0.02857 | 0.00847 |
| 1279.72 | 0.00975 | 0.01636 |  | 0.02955 | 0.02912 | 0.00822 |
| 1280.16 | 0.01010 | 0.01673 |  | 0.02955 | 0.02981 | 0.00779 |
| 1280.61 | 0.01050 | 0.01705 |  | 0.02936 | 0.03079 | 0.00755 |
| 1281.05 | 0.01002 | 0.01719 |  | 0.02899 | 0.02998 | 0.00749 |
| 1281.49 | 0.00946 | 0.01642 |  | 0.02843 | 0.02901 | 0.00750 |
| 1281.94 | 0.00902 | 0.01549 |  | 0.02771 | 0.02797 | 0.00709 |
| 1282.38 | 0.00867 | 0.01578 |  | 0.02685 | 0.02798 | 0.00664 |
| 1282.82 | 0.00846 | 0.01639 |  | 0.02599 | 0.02788 | 0.00624 |
| 1283.27 | 0.00822 | 0.01592 |  | 0.02556 | 0.02805 | 0.00622 |
| 1283.71 | 0.00797 | 0.01552 |  | 0.02553 | 0.02859 | 0.00661 |
| 1284.15 | 0.00778 | 0.01526 |  | 0.02593 | 0.02925 | 0.00827 |
| 1284.6 | 0.00766 | 0.01516 |  | 0.02630 | 0.02980 | 0.00974 |
| 1285.04 | 0.00772 | 0.01521 |  | 0.02637 | 0.02986 | 0.00966 |
| 1285.48 | 0.00853 | 0.01513 |  | 0.02637 | 0.03024 | 0.00951 |
| 1285.92 | 0.00936 | 0.01507 |  | 0.02635 | 0.03074 | 0.00905 |
| 1286.37 | 0.00965 | 0.01476 |  | 0.02636 | 0.03144 | 0.00859 |
| 1286.81 | 0.00989 | 0.01435 |  | 0.02641 | 0.03212 | 0.00814 |
| 1287.25 | 0.01007 | 0.01426 |  | 0.02651 | 0.03289 | 0.00773 |
| 1287.7 | 0.00903 | 0.01430 |  | 0.02659 | 0.03369 | 0.00750 |
| 1288.14 | 0.00842 | 0.01501 |  | 0.02676 | 0.03459 | 0.00766 |
| 1288.58 | 0.00796 | 0.01568 |  | 0.02705 | 0.03552 | 0.00784 |
| 1289.02 | 0.00769 | 0.01600 |  | 0.02765 | 0.03554 | 0.00821 |
| 1289.47 | 0.00749 | 0.01604 |  | 0.02839 | 0.03540 | 0.00883 |
| 1289.91 | 0.00742 | 0.01547 |  | 0.02944 | 0.03528 | 0.00947 |
| 1290.35 | 0.00744 | 0.01473 |  | 0.03191 | 0.03520 | 0.00904 |
| 1290.79 | 0.00757 | 0.01400 |  | 0.03219 | 0.03509 | 0.00868 |
| 1291.23 | 0.00781 | 0.01336 |  | 0.02430 | 0.03369 | 0.00847 |
| 1291.68 | 0.00813 | 0.01318 |  | 0.02531 | 0.03195 | 0.00843 |
| 1292.12 | 0.00719 | 0.01311 |  | 0.02460 | 0.03057 | 0.00851 |
| 1292.56 | 0.00583 | 0.01332 |  | 0.02685 | 0.03119 | 0.00827 |
| 1293 | 0.00591 | 0.01453 |  | 0.02790 | 0.03178 | 0.00754 |
| 1293.45 | 0.00673 | 0.01584 |  | 0.02800 | 0.03269 | 0.00672 |
| 1293.89 | 0.00804 | 0.01596 |  | 0.02752 | 0.03399 | 0.00745 |
| 1294.33 | 0.00901 | 0.01576 |  | 0.02696 | 0.03436 | 0.00812 |
| 1294.77 | 0.00945 | 0.01633 |  | 0.02630 | 0.03438 | 0.00811 |
| 1295.22 | 0.01012 | 0.01704 |  | 0.02413 | 0.03442 | 0.00778 |
| 1295.66 | 0.01020 | 0.01695 |  | 0.02326 | 0.03442 | 0.00749 |
| 1296.1 | 0.00976 | 0.01625 |  | 0.02326 | 0.03442 | 0.00723 |
| 1296.54 | 0.00914 | 0.01556 |  | 0.02406 | 0.03398 | 0.00732 |
| 1296.98 | 0.00834 | 0.01493 |  | 0.02470 | 0.03282 | 0.00759 |
| 1297.42 | 0.00818 | 0.01435 |  | 0.02508 | 0.03206 | 0.00787 |
| 1297.87 | 0.00857 | 0.01426 |  | 0.02544 | 0.03244 | 0.00816 |
| 1298.31 | 0.00853 | 0.01442 |  | 0.02573 | 0.03196 | 0.00838 |
| 1298.75 | 0.00823 | 0.01490 |  | 0.02617 | 0.03261 | 0.00863 |
| 1299.19 | 0.00856 | 0.01528 |  | 0.02649 | 0.03275 | 0.00888 |
| 1299.64 | 0.00895 | 0.01556 |  | 0.02673 | 0.03310 | 0.00914 |
| 1300.08 | 0.00921 | 0.01561 |  | 0.02660 | 0.03349 | 0.00944 |
| 1300.52 | 0.00872 | 0.01514 |  | 0.02611 | 0.03393 | 0.00977 |
| 1300.96 | 0.00814 | 0.01434 |  | 0.02589 | 0.03375 | 0.01010 |
| 1301.4 | 0.00767 | 0.01383 |  | 0.02576 | 0.03319 | 0.01007 |
| 1301.84 | 0.00738 | 0.01453 |  | 0.02564 | 0.03328 | 0.00920 |
| 1302.28 | 0.00779 | 0.01447 |  | 0.02566 | 0.03387 | 0.00777 |
| 1302.73 | 0.00847 | 0.01527 |  | 0.02581 | 0.03546 | 0.00603 |
| 1303.17 | 0.00914 | 0.01601 |  | 0.02608 | 0.03432 | 0.00425 |
| 1303.61 | 0.00960 | 0.01654 |  | 0.02650 | 0.03435 | 0.00715 |
| 1304.05 | 0.00938 | 0.01684 |  | 0.02705 | 0.03441 | 0.00700 |
| 1304.49 | 0.00906 | 0.01650 |  | 0.02781 | 0.03442 | 0.00697 |
| 1304.93 | 0.00875 | 0.01608 |  | 0.02863 | 0.03397 | 0.00709 |
| 1305.38 | 0.00847 | 0.01568 |  | 0.02814 | 0.03340 | 0.00727 |
| 1305.82 | 0.00823 | 0.01530 |  | 0.02704 | 0.03275 | 0.00754 |
| 1306.26 | 0.00804 | 0.01500 |  | 0.02863 | 0.03204 | 0.00787 |
| 1306.7 | 0.00791 | 0.01505 |  | 0.03023 | 0.03122 | 0.00831 |
| 1307.14 | 0.00787 | 0.01516 |  | 0.03100 | 0.03029 | 0.00880 |
| 1307.58 | 0.00787 | 0.01549 |  | 0.03065 | 0.02943 | 0.00927 |
| 1308.02 | 0.00798 | 0.01522 |  | 0.02864 | 0.02909 | 0.00903 |
| 1308.46 | 0.00831 | 0.01539 |  | 0.02719 | 0.02936 | 0.00861 |
| 1308.9 | 0.00890 | 0.01547 |  | 0.02618 | 0.03011 | 0.00850 |
| 1309.35 | 0.00867 | 0.01617 |  | 0.02560 | 0.03107 | 0.00867 |
| 1309.79 | 0.00855 | 0.01547 |  | 0.02525 | 0.03179 | 0.00820 |
| 1310.23 | 0.00813 | 0.01470 |  | 0.02634 | 0.03293 | 0.00783 |
| 1310.67 | 0.00783 | 0.01414 |  | 0.02750 | 0.03425 | 0.00783 |
| 1311.11 | 0.00817 | 0.01377 |  | 0.02837 | 0.03549 | 0.00795 |
| 1311.55 | 0.00868 | 0.01356 |  | 0.02887 | 0.03643 | 0.00813 |
| 1311.99 | 0.00891 | 0.01354 |  | 0.02916 | 0.03642 | 0.00834 |
| 1312.43 | 0.00915 | 0.01367 |  | 0.02931 | 0.03597 | 0.00856 |
| 1312.87 | 0.00923 | 0.01398 |  | 0.02930 | 0.03388 | 0.00870 |
| 1313.31 | 0.00912 | 0.01415 |  | 0.02918 | 0.03110 | 0.00843 |
| 1313.75 | 0.00893 | 0.01396 |  | 0.02903 | 0.03491 | 0.00780 |
| 1314.19 | 0.00887 | 0.01392 |  | 0.02897 | 0.03576 | 0.00675 |
| 1314.64 | 0.00887 | 0.01409 |  | 0.02895 | 0.03585 | 0.00656 |
| 1315.08 | 0.00873 | 0.01439 |  | 0.02902 | 0.03552 | 0.00655 |
| 1315.52 | 0.00859 | 0.01479 |  | 0.02910 | 0.03213 | 0.00677 |
| 1315.96 | 0.00856 | 0.01523 |  | 0.02923 | 0.03229 | 0.00718 |
| 1316.4 | 0.00855 | 0.01580 |  | 0.02936 | 0.03391 | 0.00802 |
| 1316.84 | 0.00853 | 0.01489 |  | 0.02958 | 0.03400 | 0.00909 |
| 1317.28 | 0.00859 | 0.01493 |  | 0.02984 | 0.03388 | 0.00929 |
| 1317.72 | 0.00871 | 0.01522 |  | 0.02998 | 0.03389 | 0.00891 |
| 1318.16 | 0.00888 | 0.01563 |  | 0.02998 | 0.03401 | 0.00861 |
| 1318.6 | 0.00901 | 0.01564 |  | 0.02958 | 0.03423 | 0.00850 |
| 1319.04 | 0.00849 | 0.01570 |  | 0.02922 | 0.03459 | 0.00874 |
| 1319.48 | 0.00798 | 0.01583 |  | 0.02892 | 0.03404 | 0.00896 |
| 1319.92 | 0.00816 | 0.01601 |  | 0.02875 | 0.03324 | 0.00800 |
| 1320.36 | 0.00861 | 0.01621 |  | 0.02872 | 0.03393 | 0.00766 |
| 1320.8 | 0.00918 | 0.01637 |  | 0.02881 | 0.03499 | 0.00775 |
| 1321.24 | 0.00953 | 0.01649 |  | 0.02904 | 0.03499 | 0.00829 |
| 1321.68 | 0.00967 | 0.01651 |  | 0.02941 | 0.03395 | 0.00924 |
| 1322.12 | 0.00965 | 0.01632 |  | 0.02998 | 0.03278 | 0.00956 |
| 1322.56 | 0.00949 | 0.01705 |  | 0.03057 | 0.03163 | 0.00858 |
| 1323 | 0.00920 | 0.01598 |  | 0.03077 | 0.03082 | 0.00798 |
| 1323.44 | 0.00859 | 0.01521 |  | 0.02978 | 0.03151 | 0.00773 |
| 1323.88 | 0.00839 | 0.01471 |  | 0.02863 | 0.03275 | 0.00793 |
| 1324.32 | 0.00878 | 0.01374 |  | 0.02770 | 0.03240 | 0.00827 |
| 1324.76 | 0.00929 | 0.01547 |  | 0.02745 | 0.03164 | 0.00745 |
| 1325.2 | 0.00909 | 0.01495 |  | 0.02676 | 0.03250 | 0.00673 |
| 1325.64 | 0.00840 | 0.01492 |  | 0.02836 | 0.03379 | 0.00646 |
| 1326.08 | 0.00826 | 0.01503 |  | 0.02800 | 0.03417 | 0.00645 |
| 1326.52 | 0.00833 | 0.01485 |  | 0.02809 | 0.03324 | 0.00835 |
| 1326.96 | 0.00855 | 0.01485 |  | 0.02823 | 0.03259 | 0.00829 |
| 1327.4 | 0.00892 | 0.01495 |  | 0.02846 | 0.03253 | 0.00806 |
| 1327.84 | 0.00870 | 0.01443 |  | 0.02868 | 0.03347 | 0.00796 |
| 1328.28 | 0.00903 | 0.01485 |  | 0.02884 | 0.03434 | 0.00796 |
| 1328.72 | 0.00902 | 0.01578 |  | 0.02890 | 0.03505 | 0.00712 |
| 1329.16 | 0.00869 | 0.01639 |  | 0.02887 | 0.03491 | 0.00776 |
| 1329.6 | 0.00808 | 0.01697 |  | 0.02879 | 0.03449 | 0.00813 |
| 1330.04 | 0.00766 | 0.01760 |  | 0.02854 | 0.03404 | 0.00856 |
| 1330.48 | 0.00744 | 0.01805 |  | 0.02790 | 0.03366 | 0.00890 |
| 1330.92 | 0.00704 | 0.01797 |  | 0.02711 | 0.03352 | 0.00916 |
| 1331.36 | 0.00661 | 0.01724 |  | 0.02634 | 0.03334 | 0.00934 |
| 1331.79 | 0.00684 | 0.01641 |  | 0.02581 | 0.03361 | 0.00940 |
| 1332.24 | 0.00734 | 0.01655 |  | 0.02586 | 0.03392 | 0.00938 |
| 1332.67 | 0.00843 | 0.01663 |  | 0.02584 | 0.03425 | 0.00906 |
| 1333.11 | 0.00891 | 0.01658 |  | 0.02630 | 0.03456 | 0.00844 |
| 1333.55 | 0.00832 | 0.01613 |  | 0.02699 | 0.03444 | 0.00785 |
| 1333.99 | 0.00752 | 0.01564 |  | 0.02774 | 0.03463 | 0.00757 |
| 1334.43 | 0.00769 | 0.01539 |  | 0.02845 | 0.03467 | 0.00803 |
| 1334.87 | 0.00758 | 0.01459 |  | 0.02904 | 0.03448 | 0.00831 |
| 1335.31 | 0.00760 | 0.01399 |  | 0.02838 | 0.03420 | 0.00768 |
| 1335.75 | 0.00775 | 0.01449 |  | 0.02805 | 0.03395 | 0.00727 |
| 1336.19 | 0.00803 | 0.01492 |  | 0.02819 | 0.03385 | 0.00696 |
| 1336.63 | 0.00846 | 0.01526 |  | 0.02860 | 0.03395 | 0.00707 |
| 1337.07 | 0.00905 | 0.01544 |  | 0.02909 | 0.03436 | 0.00729 |
| 1337.5 | 0.00959 | 0.01560 |  | 0.02957 | 0.03471 | 0.00764 |
| 1337.94 | 0.00975 | 0.01574 |  | 0.02905 | 0.03513 | 0.00802 |
| 1338.38 | 0.00954 | 0.01577 |  | 0.02833 | 0.03567 | 0.00850 |
| 1338.82 | 0.00898 | 0.01573 |  | 0.02776 | 0.03628 | 0.00896 |
| 1339.26 | 0.00839 | 0.01563 |  | 0.02722 | 0.03691 | 0.00932 |
| 1339.7 | 0.00789 | 0.01550 |  | 0.02681 | 0.03751 | 0.00959 |
| 1340.14 | 0.00755 | 0.01530 |  | 0.02655 | 0.03804 | 0.00977 |
| 1340.58 | 0.00733 | 0.01507 |  | 0.02647 | 0.03811 | 0.00988 |
| 1341.01 | 0.00725 | 0.01481 |  | 0.02670 | 0.03698 | 0.00927 |
| 1341.45 | 0.00726 | 0.01459 |  | 0.02701 | 0.03593 | 0.00804 |
| 1341.89 | 0.00743 | 0.01464 |  | 0.02737 | 0.03376 | 0.00750 |
| 1342.33 | 0.00780 | 0.01478 |  | 0.02778 | 0.03119 | 0.00718 |
| 1342.77 | 0.00835 | 0.01524 |  | 0.02817 | 0.03079 | 0.00891 |
| 1343.21 | 0.00788 | 0.01546 |  | 0.02866 | 0.03373 | 0.00437 |
| 1343.64 | 0.00868 | 0.01544 |  | 0.02912 | 0.03470 | 0.00509 |
| 1344.08 | 0.00829 | 0.01541 |  | 0.02937 | 0.03486 | 0.00573 |
| 1344.52 | 0.00795 | 0.01531 |  | 0.02938 | 0.03555 | 0.00720 |
| 1344.96 | 0.00816 | 0.01523 |  | 0.02910 | 0.03599 | 0.00842 |
| 1345.4 | 0.00815 | 0.01516 |  | 0.02859 | 0.03551 | 0.00851 |
| 1345.84 | 0.00776 | 0.01517 |  | 0.02790 | 0.03420 | 0.00864 |
| 1346.28 | 0.00792 | 0.01515 |  | 0.02709 | 0.03312 | 0.00838 |
| 1346.71 | 0.00799 | 0.01481 |  | 0.02591 | 0.03216 | 0.00861 |
| 1347.15 | 0.00812 | 0.01443 |  | 0.02615 | 0.03193 | 0.00880 |
| 1347.59 | 0.00833 | 0.01417 |  | 0.02813 | 0.03197 | 0.00881 |
| 1348.03 | 0.00829 | 0.01405 |  | 0.02904 | 0.03214 | 0.00875 |
| 1348.47 | 0.00794 | 0.01405 |  | 0.02960 | 0.03155 | 0.00880 |
| 1348.9 | 0.00806 | 0.01418 |  | 0.02759 | 0.03177 | 0.00883 |
| 1349.34 | 0.00851 | 0.01440 |  | 0.02610 | 0.03190 | 0.00884 |
| 1349.78 | 0.00914 | 0.01465 |  | 0.02438 | 0.03176 | 0.00870 |
| 1350.22 | 0.00947 | 0.01491 |  | 0.02391 | 0.03170 | 0.00835 |
| 1350.66 | 0.00970 | 0.01420 |  | 0.02549 | 0.03285 | 0.00803 |
| 1351.09 | 0.00992 | 0.01392 |  | 0.02600 | 0.03395 | 0.00800 |
| 1351.53 | 0.01000 | 0.01385 |  | 0.02632 | 0.03497 | 0.00781 |
| 1351.97 | 0.00953 | 0.01413 |  | 0.02646 | 0.03510 | 0.00735 |
| 1352.41 | 0.00948 | 0.01463 |  | 0.02643 | 0.03423 | 0.00677 |
| 1352.85 | 0.00945 | 0.01392 |  | 0.02636 | 0.03297 | 0.00762 |
| 1353.28 | 0.00932 | 0.01352 |  | 0.02616 | 0.03203 | 0.00811 |
| 1353.72 | 0.00894 | 0.01334 |  | 0.02588 | 0.03184 | 0.00848 |
| 1354.16 | 0.00830 | 0.01357 |  | 0.02547 | 0.03358 | 0.00871 |
| 1354.6 | 0.00743 | 0.01395 |  | 0.02500 | 0.03269 | 0.00885 |
| 1355.03 | 0.00663 | 0.01458 |  | 0.02453 | 0.03138 | 0.00888 |
| 1355.47 | 0.00627 | 0.01512 |  | 0.02419 | 0.03122 | 0.00866 |
| 1355.91 | 0.00645 | 0.01506 |  | 0.02394 | 0.03192 | 0.00858 |
| 1356.35 | 0.00709 | 0.01510 |  | 0.02382 | 0.03319 | 0.00827 |
| 1356.78 | 0.00801 | 0.01521 |  | 0.02385 | 0.03375 | 0.00882 |
| 1357.22 | 0.00871 | 0.01541 |  | 0.02396 | 0.03301 | 0.00918 |
| 1357.66 | 0.00893 | 0.01570 |  | 0.02422 | 0.03229 | 0.00910 |
| 1358.1 | 0.00915 | 0.01617 |  | 0.02462 | 0.03198 | 0.00897 |
| 1358.53 | 0.00906 | 0.01639 |  | 0.02532 | 0.03318 | 0.00889 |
| 1358.97 | 0.00878 | 0.01596 |  | 0.02654 | 0.03358 | 0.00882 |
| 1359.41 | 0.00780 | 0.01550 |  | 0.02750 | 0.03403 | 0.00876 |
| 1359.85 | 0.00741 | 0.01506 |  | 0.02787 | 0.03457 | 0.00832 |
| 1360.28 | 0.00768 | 0.01467 |  | 0.02771 | 0.03485 | 0.00824 |
| 1360.72 | 0.00858 | 0.01432 |  | 0.02746 | 0.03465 | 0.00826 |
| 1361.16 | 0.00844 | 0.01404 |  | 0.02724 | 0.03415 | 0.00851 |
| 1361.59 | 0.00847 | 0.01282 |  | 0.02720 | 0.03362 | 0.00817 |
| 1362.03 | 0.00859 | 0.01135 |  | 0.02686 | 0.03362 | 0.00781 |
| 1362.47 | 0.00789 | 0.01086 |  | 0.02696 | 0.03274 | 0.00751 |
| 1362.91 | 0.00599 | 0.01149 |  | 0.02728 | 0.03158 | 0.00724 |
| 1363.34 | 0.00591 | 0.01296 |  | 0.02773 | 0.03008 | 0.00718 |
| 1363.78 | 0.00612 | 0.01482 |  | 0.02761 | 0.03039 | 0.00668 |
| 1364.22 | 0.00627 | 0.01422 |  | 0.02645 | 0.03187 | 0.00630 |
| 1364.65 | 0.00638 | 0.01413 |  | 0.02616 | 0.03288 | 0.00757 |
| 1365.09 | 0.00635 | 0.01408 |  | 0.02611 | 0.03375 | 0.00838 |
| 1365.53 | 0.00626 | 0.01408 |  | 0.02589 | 0.03451 | 0.00851 |
| 1365.96 | 0.00643 | 0.01419 |  | 0.02541 | 0.03458 | 0.00812 |
| 1366.4 | 0.00750 | 0.01434 |  | 0.02488 | 0.03403 | 0.00780 |
| 1366.84 | 0.00801 | 0.01454 |  | 0.02417 | 0.03248 | 0.00741 |
| 1367.27 | 0.00821 | 0.01307 |  | 0.02373 | 0.03116 | 0.00685 |
| 1367.71 | 0.00841 | 0.01328 |  | 0.02442 | 0.03016 | 0.00621 |
| 1368.15 | 0.00846 | 0.01381 |  | 0.02550 | 0.03069 | 0.00572 |
| 1368.58 | 0.00829 | 0.01418 |  | 0.02634 | 0.03123 | 0.00530 |
| 1369.02 | 0.00826 | 0.01463 |  | 0.02715 | 0.03076 | 0.00499 |
| 1369.46 | 0.00837 | 0.01478 |  | 0.02782 | 0.03031 | 0.00497 |
| 1369.89 | 0.00862 | 0.01421 |  | 0.02837 | 0.02994 | 0.00556 |
| 1370.33 | 0.00891 | 0.01389 |  | 0.02877 | 0.02951 | 0.00611 |
| 1370.77 | 0.00918 | 0.01376 |  | 0.02904 | 0.02949 | 0.00681 |
| 1371.2 | 0.00930 | 0.01377 |  | 0.02906 | 0.02984 | 0.00756 |
| 1371.64 | 0.00932 | 0.01384 |  | 0.02893 | 0.03068 | 0.00819 |
| 1372.07 | 0.00924 | 0.01384 |  | 0.02870 | 0.03061 | 0.00842 |
| 1372.51 | 0.00908 | 0.01395 |  | 0.02856 | 0.03019 | 0.00794 |
| 1372.95 | 0.00878 | 0.01404 |  | 0.02849 | 0.03009 | 0.00702 |
| 1373.38 | 0.00840 | 0.01397 |  | 0.02849 | 0.03027 | 0.00668 |
| 1373.82 | 0.00790 | 0.01354 |  | 0.02767 | 0.02970 | 0.00732 |
| 1374.26 | 0.00740 | 0.01311 |  | 0.02653 | 0.02814 | 0.00833 |
| 1374.69 | 0.00740 | 0.01290 |  | 0.02550 | 0.02966 | 0.00916 |
| 1375.13 | 0.00760 | 0.01292 |  | 0.02483 | 0.03163 | 0.00979 |
| 1375.57 | 0.00783 | 0.01308 |  | 0.02441 | 0.03251 | 0.01016 |
| 1376 | 0.00800 | 0.01337 |  | 0.02424 | 0.03301 | 0.00810 |
| 1376.44 | 0.00802 | 0.01368 |  | 0.02427 | 0.03176 | 0.00597 |
| 1376.87 | 0.00794 | 0.01397 |  | 0.02447 | 0.02969 | 0.00560 |
| 1377.31 | 0.00782 | 0.01455 |  | 0.02474 | 0.02738 | 0.00535 |
| 1377.74 | 0.00771 | 0.01521 |  | 0.02504 | 0.02508 | 0.00530 |
| 1378.18 | 0.00758 | 0.01499 |  | 0.02528 | 0.02416 | 0.00577 |
| 1378.62 | 0.00743 | 0.01482 |  | 0.02541 | 0.02890 | 0.00663 |
| 1379.05 | 0.00733 | 0.01476 |  | 0.02553 | 0.03065 | 0.00752 |
| 1379.49 | 0.00729 | 0.01428 |  | 0.02559 | 0.03075 | 0.00831 |
| 1379.92 | 0.00737 | 0.01397 |  | 0.02534 | 0.03134 | 0.00787 |
| 1380.36 | 0.00756 | 0.01378 |  | 0.02488 | 0.03205 | 0.00718 |
| 1380.79 | 0.00785 | 0.01359 |  | 0.02413 | 0.03216 | 0.00653 |
| 1381.23 | 0.00809 | 0.01369 |  | 0.02417 | 0.03227 | 0.00628 |
| 1381.67 | 0.00785 | 0.01391 |  | 0.02682 | 0.03242 | 0.00667 |
| 1382.1 | 0.00765 | 0.01425 |  | 0.02837 | 0.03261 | 0.00672 |
| 1382.54 | 0.00751 | 0.01470 |  | 0.02747 | 0.03168 | 0.00722 |
| 1382.97 | 0.00747 | 0.01503 |  | 0.02698 | 0.02928 | 0.00753 |
| 1383.41 | 0.00757 | 0.01553 |  | 0.02675 | 0.02754 | 0.00811 |
| 1383.84 | 0.00780 | 0.01582 |  | 0.02678 | 0.02627 | 0.00866 |
| 1384.28 | 0.00810 | 0.01606 |  | 0.02700 | 0.02614 | 0.00899 |
| 1384.71 | 0.00810 | 0.01627 |  | 0.02708 | 0.02760 | 0.00860 |
| 1385.15 | 0.00816 | 0.01636 |  | 0.02578 | 0.02934 | 0.00822 |
| 1385.59 | 0.00822 | 0.01628 |  | 0.02450 | 0.03113 | 0.00803 |
| 1386.02 | 0.00827 | 0.01511 |  | 0.02472 | 0.03255 | 0.00784 |
| 1386.46 | 0.00827 | 0.01449 |  | 0.02497 | 0.03324 | 0.00762 |
| 1386.89 | 0.00823 | 0.01422 |  | 0.02518 | 0.03162 | 0.00741 |
| 1387.33 | 0.00811 | 0.01427 |  | 0.02533 | 0.03021 | 0.00682 |
| 1387.76 | 0.00788 | 0.01406 |  | 0.02541 | 0.02899 | 0.00636 |
| 1388.2 | 0.00741 | 0.01314 |  | 0.02540 | 0.02807 | 0.00612 |
| 1388.63 | 0.00698 | 0.01206 |  | 0.02521 | 0.02769 | 0.00609 |
| 1389.07 | 0.00692 | 0.01400 |  | 0.02495 | 0.02793 | 0.00631 |
| 1389.5 | 0.00707 | 0.01388 |  | 0.02470 | 0.02858 | 0.00639 |
| 1389.94 | 0.00740 | 0.01379 |  | 0.02458 | 0.02974 | 0.00625 |
| 1390.37 | 0.00731 | 0.01376 |  | 0.02466 | 0.03059 | 0.00633 |
| 1390.81 | 0.00674 | 0.01381 |  | 0.02484 | 0.03108 | 0.00662 |
| 1391.24 | 0.00624 | 0.01405 |  | 0.02520 | 0.03120 | 0.00718 |
| 1391.68 | 0.00667 | 0.01421 |  | 0.02561 | 0.03062 | 0.00473 |
| 1392.11 | 0.00732 | 0.01435 |  | 0.02605 | 0.02983 | 0.00044 |
| 1392.55 | 0.00786 | 0.01443 |  | 0.02644 | 0.02855 | 0.00797 |
| 1392.98 | 0.00704 | 0.01447 |  | 0.02663 | 0.02731 | 0.00815 |
| 1393.42 | 0.00663 | 0.01443 |  | 0.02665 | 0.02637 | 0.00801 |
| 1393.85 | 0.00661 | 0.01452 |  | 0.02653 | 0.02556 | 0.00776 |
| 1394.28 | 0.00704 | 0.01467 |  | 0.02608 | 0.02600 | 0.00487 |
| 1394.72 | 0.00695 | 0.01464 |  | 0.02526 | 0.02667 | 0.00331 |
| 1395.15 | 0.00673 | 0.01518 |  | 0.02443 | 0.02771 | 0.00615 |
| 1395.59 | 0.00666 | 0.01524 |  | 0.02429 | 0.02923 | 0.00538 |
| 1396.02 | 0.00669 | 0.01525 |  | 0.02525 | 0.03078 | 0.00465 |
| 1396.46 | 0.00649 | 0.01283 |  | 0.02570 | 0.03154 | 0.00441 |
| 1396.89 | 0.00611 | 0.01169 |  | 0.02637 | 0.03234 | 0.00228 |
| 1397.33 | 0.00584 | 0.01195 |  | 0.02661 | 0.03258 | 0.00223 |
| 1397.76 | 0.00561 | 0.01251 |  | 0.02633 | 0.03205 | 0.00336 |
| 1398.2 | 0.00582 | 0.01308 |  | 0.02582 | 0.03136 | 0.00796 |
| 1398.63 | 0.00600 | 0.01364 |  | 0.02516 | 0.03059 | 0.00713 |
| 1399.06 | 0.00629 | 0.01421 |  | 0.02445 | 0.03031 | 0.00621 |
| 1399.5 | 0.00665 | 0.01455 |  | 0.02390 | 0.03051 | 0.00685 |
| 1399.93 | 0.00712 | 0.01474 |  | 0.02345 | 0.03108 | 0.00748 |
| 1400.37 | 0.00663 | 0.01472 |  | 0.02317 | 0.03172 | 0.00682 |
| 1400.8 | 0.00634 | 0.01455 |  | 0.02336 | 0.03005 | 0.00618 |
| 1401.23 | 0.00628 | 0.01428 |  | 0.02409 | 0.02854 | 0.00572 |
| 1401.67 | 0.00622 | 0.01392 |  | 0.02380 | 0.02793 | 0.00556 |
| 1402.1 | 0.00627 | 0.01359 |  | 0.02261 | 0.02763 | 0.00557 |
| 1402.54 | 0.00638 | 0.01384 |  | 0.02253 | 0.02796 | 0.00568 |
| 1402.97 | 0.00655 | 0.01390 |  | 0.02334 | 0.02852 | 0.00591 |
| 1403.4 | 0.00674 | 0.01339 |  | 0.02429 | 0.02931 | 0.00618 |
| 1403.84 | 0.00694 | 0.01334 |  | 0.02496 | 0.03031 | 0.00641 |
| 1404.27 | 0.00716 | 0.01410 |  | 0.02547 | 0.03159 | 0.00652 |
| 1404.71 | 0.00629 | 0.01496 |  | 0.02594 | 0.03301 | 0.00662 |
| 1405.14 | 0.00477 | 0.01564 |  | 0.02637 | 0.03288 | 0.00669 |
| 1405.57 | 0.00475 | 0.01552 |  | 0.02675 | 0.03235 | 0.00669 |
| 1406.01 | 0.00594 | 0.01521 |  | 0.02703 | 0.03185 | 0.00666 |
| 1406.44 | 0.00680 | 0.01473 |  | 0.02728 | 0.03134 | 0.00656 |
| 1406.87 | 0.00749 | 0.01398 |  | 0.02746 | 0.03169 | 0.00642 |
| 1407.31 | 0.00782 | 0.01313 |  | 0.02761 | 0.03015 | 0.00631 |
| 1407.74 | 0.00747 | 0.01248 |  | 0.02760 | 0.02839 | 0.00633 |
| 1408.17 | 0.00689 | 0.01255 |  | 0.02719 | 0.02851 | 0.00748 |
| 1408.61 | 0.00692 | 0.01292 |  | 0.02612 | 0.02945 | 0.00851 |
| 1409.04 | 0.00700 | 0.01327 |  | 0.02534 | 0.03092 | 0.00916 |
| 1409.48 | 0.00715 | 0.01397 |  | 0.02590 | 0.03186 | 0.00952 |
| 1409.91 | 0.00726 | 0.01451 |  | 0.02644 | 0.03166 | 0.00956 |
| 1410.34 | 0.00733 | 0.01402 |  | 0.02675 | 0.03179 | 0.00925 |
| 1410.78 | 0.00718 | 0.01333 |  | 0.02683 | 0.03227 | 0.00860 |
| 1411.21 | 0.00703 | 0.01304 |  | 0.02676 | 0.03289 | 0.00798 |
| 1411.64 | 0.00737 | 0.01292 |  | 0.02671 | 0.03346 | 0.00696 |
| 1412.08 | 0.00805 | 0.01292 |  | 0.02627 | 0.03348 | 0.00619 |
| 1412.51 | 0.00811 | 0.01303 |  | 0.02569 | 0.03247 | 0.00584 |
| 1412.94 | 0.00823 | 0.01321 |  | 0.02491 | 0.03109 | 0.00651 |
| 1413.38 | 0.00826 | 0.01350 |  | 0.02422 | 0.02965 | 0.00668 |
| 1413.81 | 0.00839 | 0.01277 |  | 0.02496 | 0.02880 | 0.00694 |
| 1414.24 | 0.00796 | 0.01227 |  | 0.02590 | 0.02836 | 0.00737 |
| 1414.67 | 0.00784 | 0.01217 |  | 0.02693 | 0.02833 | 0.00723 |
| 1415.11 | 0.00792 | 0.01257 |  | 0.02759 | 0.02931 | 0.00582 |
| 1415.54 | 0.00804 | 0.01303 |  | 0.02740 | 0.03117 | 0.00550 |
| 1415.97 | 0.00841 | 0.01357 |  | 0.02620 | 0.03288 | 0.00546 |
| 1416.41 | 0.00886 | 0.01418 |  | 0.02428 | 0.03395 | 0.00686 |
| 1416.84 | 0.00915 | 0.01471 |  | 0.02319 | 0.03427 | 0.00750 |
| 1417.27 | 0.00915 | 0.01434 |  | 0.02337 | 0.03415 | 0.00813 |
| 1417.71 | 0.00899 | 0.01411 |  | 0.02431 | 0.03414 | 0.00842 |
| 1418.14 | 0.00866 | 0.01430 |  | 0.02523 | 0.03373 | 0.00790 |
| 1418.57 | 0.00822 | 0.01474 |  | 0.02663 | 0.03312 | 0.00698 |
| 1419 | 0.00811 | 0.01477 |  | 0.02760 | 0.03240 | 0.00619 |
| 1419.44 | 0.00802 | 0.01490 |  | 0.02765 | 0.03136 | 0.00556 |
| 1419.87 | 0.00798 | 0.01498 |  | 0.02708 | 0.03095 | 0.00587 |
| 1420.3 | 0.00792 | 0.01471 |  | 0.02591 | 0.03052 | 0.00628 |
| 1420.73 | 0.00776 | 0.01424 |  | 0.02468 | 0.03061 | 0.00729 |
| 1421.17 | 0.00710 | 0.01396 |  | 0.02408 | 0.03083 | 0.00780 |
| 1421.6 | 0.00686 | 0.01386 |  | 0.02358 | 0.03120 | 0.00814 |
| 1422.03 | 0.00682 | 0.01431 |  | 0.02353 | 0.03191 | 0.00795 |
| 1422.47 | 0.00692 | 0.01441 |  | 0.02467 | 0.03307 | 0.00785 |
| 1422.9 | 0.00719 | 0.01440 |  | 0.02555 | 0.03486 | 0.00744 |
| 1423.33 | 0.00770 | 0.01436 |  | 0.02664 | 0.03417 | 0.00723 |
| 1423.76 | 0.00834 | 0.01428 |  | 0.02747 | 0.03461 | 0.00722 |
| 1424.2 | 0.00869 | 0.01438 |  | 0.02781 | 0.03470 | 0.00738 |
| 1424.63 | 0.00898 | 0.01446 |  | 0.02728 | 0.03340 | 0.00771 |
| 1425.06 | 0.00926 | 0.01454 |  | 0.02693 | 0.03380 | 0.00801 |
| 1425.49 | 0.00912 | 0.01380 |  | 0.02663 | 0.03316 | 0.00766 |
| 1425.92 | 0.00887 | 0.01333 |  | 0.02628 | 0.03292 | 0.00746 |
| 1426.36 | 0.00857 | 0.01394 |  | 0.02615 | 0.03205 | 0.00737 |
| 1426.79 | 0.00827 | 0.01469 |  | 0.02615 | 0.03163 | 0.00735 |
| 1427.22 | 0.00797 | 0.01534 |  | 0.02592 | 0.03048 | 0.00741 |
| 1427.65 | 0.00775 | 0.01493 |  | 0.02584 | 0.03194 | 0.00758 |
| 1428.08 | 0.00769 | 0.01442 |  | 0.02584 | 0.03372 | 0.00782 |
| 1428.52 | 0.00766 | 0.01375 |  | 0.02582 | 0.03349 | 0.00793 |
| 1428.95 | 0.00785 | 0.01317 |  | 0.02543 | 0.03356 | 0.00743 |
| 1429.38 | 0.00814 | 0.01316 |  | 0.02508 | 0.03352 | 0.00736 |
| 1429.81 | 0.00843 | 0.01350 |  | 0.02471 | 0.03347 | 0.00800 |
| 1430.25 | 0.00826 | 0.01406 |  | 0.02444 | 0.03375 | 0.00889 |
| 1430.68 | 0.00844 | 0.01413 |  | 0.02417 | 0.03411 | 0.00973 |
| 1431.11 | 0.00845 | 0.01412 |  | 0.02406 | 0.03466 | 0.00977 |
| 1431.54 | 0.00832 | 0.01409 |  | 0.02411 | 0.03531 | 0.00945 |
| 1431.97 | 0.00810 | 0.01413 |  | 0.02427 | 0.03606 | 0.00909 |
| 1432.4 | 0.00790 | 0.01415 |  | 0.02459 | 0.03677 | 0.00892 |
| 1432.84 | 0.00756 | 0.01390 |  | 0.02507 | 0.03645 | 0.00880 |
| 1433.27 | 0.00735 | 0.01362 |  | 0.02578 | 0.03608 | 0.00881 |
| 1433.7 | 0.00751 | 0.01330 |  | 0.02623 | 0.03581 | 0.00906 |
| 1434.13 | 0.00773 | 0.01343 |  | 0.02632 | 0.03557 | 0.00983 |
| 1434.56 | 0.00802 | 0.01355 |  | 0.02642 | 0.03484 | 0.01054 |
| 1434.99 | 0.00845 | 0.01390 |  | 0.02646 | 0.03383 | 0.01067 |
| 1435.43 | 0.00877 | 0.01427 |  | 0.02617 | 0.03222 | 0.01046 |
| 1435.86 | 0.00903 | 0.01472 |  | 0.02577 | 0.03218 | 0.01002 |
| 1436.29 | 0.00936 | 0.01530 |  | 0.02551 | 0.03343 | 0.00979 |
| 1436.72 | 0.00971 | 0.01585 |  | 0.02566 | 0.03512 | 0.00961 |
| 1437.15 | 0.00946 | 0.01624 |  | 0.02590 | 0.03591 | 0.00925 |
| 1437.58 | 0.00938 | 0.01612 |  | 0.02611 | 0.03565 | 0.00869 |
| 1438.01 | 0.00932 | 0.01577 |  | 0.02639 | 0.03495 | 0.00872 |
| 1438.44 | 0.00914 | 0.01517 |  | 0.02678 | 0.03447 | 0.00926 |
| 1438.88 | 0.00817 | 0.01461 |  | 0.02765 | 0.03455 | 0.00987 |
| 1439.31 | 0.00775 | 0.01482 |  | 0.02822 | 0.03474 | 0.01014 |
| 1439.74 | 0.00801 | 0.01471 |  | 0.02818 | 0.03453 | 0.00948 |
| 1440.17 | 0.00824 | 0.01444 |  | 0.02772 | 0.03445 | 0.00910 |
| 1440.6 | 0.00811 | 0.01422 |  | 0.02674 | 0.03438 | 0.00920 |
| 1441.03 | 0.00815 | 0.01410 |  | 0.02556 | 0.03432 | 0.00981 |
| 1441.46 | 0.00800 | 0.01431 |  | 0.02499 | 0.03426 | 0.01016 |
| 1441.89 | 0.00803 | 0.01387 |  | 0.02448 | 0.03426 | 0.01019 |
| 1442.33 | 0.00798 | 0.01320 |  | 0.02376 | 0.03442 | 0.01060 |
| 1442.76 | 0.00820 | 0.01233 |  | 0.02373 | 0.03455 | 0.01058 |
| 1443.19 | 0.00889 | 0.01220 |  | 0.02429 | 0.03445 | 0.00988 |
| 1443.62 | 0.00916 | 0.01488 |  | 0.02566 | 0.03453 | 0.00884 |
| 1444.05 | 0.00905 | 0.01508 |  | 0.02528 | 0.03502 | 0.00788 |
| 1444.48 | 0.00897 | 0.01471 |  | 0.02492 | 0.03571 | 0.00796 |
| 1444.91 | 0.00896 | 0.01396 |  | 0.02480 | 0.03618 | 0.00834 |
| 1445.34 | 0.00903 | 0.01304 |  | 0.02503 | 0.03660 | 0.00878 |
| 1445.77 | 0.00919 | 0.01299 |  | 0.02540 | 0.03684 | 0.00940 |
| 1446.2 | 0.00948 | 0.01317 |  | 0.02572 | 0.03709 | 0.00996 |
| 1446.63 | 0.00977 | 0.01363 |  | 0.02587 | 0.03673 | 0.01022 |
| 1447.06 | 0.00987 | 0.01427 |  | 0.02570 | 0.03614 | 0.01034 |
| 1447.5 | 0.00951 | 0.01465 |  | 0.02520 | 0.03580 | 0.01030 |
| 1447.93 | 0.00916 | 0.01472 |  | 0.02448 | 0.03571 | 0.00996 |
| 1448.36 | 0.00920 | 0.01458 |  | 0.02369 | 0.03526 | 0.00939 |
| 1448.79 | 0.00934 | 0.01449 |  | 0.02290 | 0.03523 | 0.00840 |
| 1449.22 | 0.00923 | 0.01413 |  | 0.02263 | 0.03572 | 0.00775 |
| 1449.65 | 0.00911 | 0.01385 |  | 0.02295 | 0.03629 | 0.00736 |
| 1450.08 | 0.00909 | 0.01375 |  | 0.02382 | 0.03684 | 0.00754 |
| 1450.51 | 0.00914 | 0.01385 |  | 0.02527 | 0.03729 | 0.00805 |
| 1450.94 | 0.00934 | 0.01395 |  | 0.02630 | 0.03772 | 0.00902 |
| 1451.37 | 0.00948 | 0.01431 |  | 0.02651 | 0.03819 | 0.00970 |
| 1451.8 | 0.00924 | 0.01452 |  | 0.02657 | 0.03865 | 0.01015 |
| 1452.23 | 0.00894 | 0.01440 |  | 0.02625 | 0.03898 | 0.00962 |
| 1452.66 | 0.00876 | 0.01414 |  | 0.02500 | 0.03913 | 0.00987 |
| 1453.09 | 0.00880 | 0.01394 |  | 0.02377 | 0.03898 | 0.01011 |
| 1453.52 | 0.00838 | 0.01381 |  | 0.02304 | 0.03828 | 0.00983 |
| 1453.95 | 0.00835 | 0.01371 |  | 0.02275 | 0.03710 | 0.00970 |
| 1454.38 | 0.00885 | 0.01364 |  | 0.02285 | 0.03578 | 0.00973 |
| 1454.81 | 0.00922 | 0.01373 |  | 0.02331 | 0.03556 | 0.00988 |
| 1455.24 | 0.00958 | 0.01391 |  | 0.02415 | 0.03580 | 0.01009 |
| 1455.67 | 0.00989 | 0.01415 |  | 0.02490 | 0.03553 | 0.01039 |
| 1456.1 | 0.01013 | 0.01439 |  | 0.02551 | 0.03507 | 0.01063 |
| 1456.53 | 0.01011 | 0.01455 |  | 0.02601 | 0.03460 | 0.01077 |
| 1456.96 | 0.01004 | 0.01444 |  | 0.02647 | 0.03422 | 0.01051 |
| 1457.39 | 0.01025 | 0.01397 |  | 0.02644 | 0.03406 | 0.01015 |
| 1457.82 | 0.01073 | 0.01337 |  | 0.02546 | 0.03402 | 0.00988 |
| 1458.25 | 0.01131 | 0.01302 |  | 0.02423 | 0.03414 | 0.00976 |
| 1458.68 | 0.01153 | 0.01298 |  | 0.02359 | 0.03457 | 0.00993 |
| 1459.11 | 0.01133 | 0.01313 |  | 0.02381 | 0.03514 | 0.01000 |
| 1459.54 | 0.01107 | 0.01341 |  | 0.02446 | 0.03589 | 0.00980 |
| 1459.79 | 0.01094 | 0.01361 |  | 0.02489 | 0.03623 | 0.00946 |
| 1460.23 | 0.01056 | 0.01369 |  | 0.02378 | 0.03649 | 0.00894 |
| 1460.66 | 0.01022 | 0.01381 |  | 0.02317 | 0.03664 | 0.00952 |
| 1461.1 | 0.00992 | 0.01393 |  | 0.02310 | 0.03669 | 0.00989 |
| 1461.53 | 0.00980 | 0.01401 |  | 0.02332 | 0.03665 | 0.00944 |
| 1461.97 | 0.00980 | 0.01409 |  | 0.02354 | 0.03643 | 0.00889 |
| 1462.4 | 0.00988 | 0.01414 |  | 0.02359 | 0.03614 | 0.00810 |
| 1462.84 | 0.00999 | 0.01418 |  | 0.02346 | 0.03559 | 0.00943 |
| 1463.27 | 0.00997 | 0.01420 |  | 0.02321 | 0.03491 | 0.00931 |
| 1463.71 | 0.01000 | 0.01418 |  | 0.02280 | 0.03427 | 0.00868 |
| 1464.14 | 0.00985 | 0.01409 |  | 0.02265 | 0.03398 | 0.00791 |
| 1464.58 | 0.00969 | 0.01382 |  | 0.02295 | 0.03424 | 0.00766 |
| 1465.01 | 0.00951 | 0.01382 |  | 0.02336 | 0.03462 | 0.00834 |
| 1465.45 | 0.00935 | 0.01364 |  | 0.02381 | 0.03558 | 0.00892 |
| 1465.88 | 0.00931 | 0.01338 |  | 0.02428 | 0.03648 | 0.00971 |
| 1466.32 | 0.00948 | 0.01312 |  | 0.02485 | 0.03731 | 0.01062 |
| 1466.75 | 0.00932 | 0.01309 |  | 0.02497 | 0.03696 | 0.01091 |
| 1467.18 | 0.00920 | 0.01355 |  | 0.02419 | 0.03638 | 0.01163 |
| 1467.62 | 0.00914 | 0.01526 |  | 0.02370 | 0.03641 | 0.01152 |
| 1468.05 | 0.00896 | 0.01601 |  | 0.02349 | 0.03591 | 0.01106 |
| 1468.49 | 0.00865 | 0.01484 |  | 0.02367 | 0.03478 | 0.00969 |
| 1468.92 | 0.00853 | 0.01486 |  | 0.02418 | 0.03372 | 0.00877 |
| 1469.36 | 0.00854 | 0.01277 |  | 0.02481 | 0.03321 | 0.00848 |
| 1469.79 | 0.00868 | 0.01156 |  | 0.02522 | 0.03291 | 0.00873 |
| 1470.22 | 0.00899 | 0.01049 |  | 0.02529 | 0.03281 | 0.00919 |
| 1470.66 | 0.00945 | 0.01152 |  | 0.02516 | 0.03285 | 0.00887 |
| 1471.09 | 0.00968 | 0.01056 |  | 0.02153 | 0.03321 | 0.00844 |
| 1471.53 | 0.01001 | 0.01268 |  | 0.02557 | 0.03372 | 0.00818 |
| 1471.96 | 0.01039 | 0.01323 |  | 0.02484 | 0.03415 | 0.00798 |
| 1472.4 | 0.01066 | 0.01373 |  | 0.02489 | 0.03447 | 0.00813 |
| 1472.83 | 0.01068 | 0.01401 |  | 0.02440 | 0.03463 | 0.00868 |
| 1473.26 | 0.00991 | 0.01423 |  | 0.02407 | 0.03470 | 0.00939 |
| 1473.7 | 0.00878 | 0.01433 |  | 0.02386 | 0.03459 | 0.01018 |
| 1474.13 | 0.00880 | 0.01434 |  | 0.02381 | 0.03434 | 0.01089 |
| 1474.57 | 0.00964 | 0.01424 |  | 0.02382 | 0.03392 | 0.01090 |
| 1475 | 0.00939 | 0.01401 |  | 0.02387 | 0.03335 | 0.01084 |
| 1475.43 | 0.00861 | 0.01388 |  | 0.02396 | 0.03262 | 0.01074 |
| 1475.87 | 0.00820 | 0.01384 |  | 0.02405 | 0.03174 | 0.01062 |
| 1476.3 | 0.00797 | 0.01395 |  | 0.02409 | 0.03066 | 0.01049 |
| 1476.73 | 0.00802 | 0.01414 |  | 0.02413 | 0.02950 | 0.01030 |
| 1477.17 | 0.00829 | 0.01439 |  | 0.02421 | 0.02906 | 0.01008 |
| 1477.6 | 0.00875 | 0.01469 |  | 0.02426 | 0.03053 | 0.00984 |
| 1478.04 | 0.00899 | 0.01492 |  | 0.02425 | 0.03381 | 0.00966 |
| 1478.47 | 0.00871 | 0.01456 |  | 0.02390 | 0.03452 | 0.00984 |
| 1478.9 | 0.00884 | 0.01395 |  | 0.02219 | 0.03360 | 0.01009 |
| 1479.34 | 0.00894 | 0.01355 |  | 0.02018 | 0.03298 | 0.01000 |
| 1479.77 | 0.00915 | 0.01416 |  | 0.02217 | 0.03216 | 0.00957 |
| 1480.2 | 0.00939 | 0.01453 |  | 0.02205 | 0.03122 | 0.00935 |
| 1480.64 | 0.00968 | 0.01461 |  | 0.02194 | 0.02976 | 0.00988 |
| 1481.07 | 0.00996 | 0.01450 |  | 0.02179 | 0.02859 | 0.01009 |
| 1481.5 | 0.01018 | 0.01422 |  | 0.02165 | 0.03012 | 0.01018 |
| 1481.94 | 0.01021 | 0.01393 |  | 0.02154 | 0.03077 | 0.01021 |
| 1482.37 | 0.00908 | 0.01336 |  | 0.02144 | 0.03144 | 0.01022 |
| 1482.8 | 0.00817 | 0.01274 |  | 0.02139 | 0.03201 | 0.01013 |
| 1483.23 | 0.00756 | 0.01215 |  | 0.02142 | 0.03251 | 0.00995 |
| 1483.67 | 0.00737 | 0.01156 |  | 0.02168 | 0.03299 | 0.00969 |
| 1484.1 | 0.00772 | 0.01138 |  | 0.02244 | 0.03343 | 0.00934 |
| 1484.53 | 0.00862 | 0.01149 |  | 0.02319 | 0.03379 | 0.00909 |
| 1484.97 | 0.00841 | 0.01172 |  | 0.02418 | 0.03413 | 0.00895 |
| 1485.4 | 0.00371 | 0.01208 |  | 0.02345 | 0.03443 | 0.00882 |
| 1485.83 | 0.00922 | 0.01256 |  | 0.02256 | 0.03471 | 0.00881 |
| 1486.27 | 0.00933 | 0.01295 |  | 0.02195 | 0.03500 | 0.00877 |
| 1486.7 | 0.00934 | 0.01273 |  | 0.02153 | 0.03528 | 0.00880 |
| 1487.13 | 0.00925 | 0.01268 |  | 0.02133 | 0.03557 | 0.00785 |
| 1487.57 | 0.00906 | 0.01258 |  | 0.02140 | 0.03566 | 0.00776 |
| 1488 | 0.00860 | 0.01249 |  | 0.02158 | 0.03475 | 0.00779 |
| 1488.43 | 0.00800 | 0.01245 |  | 0.02199 | 0.03417 | 0.00813 |
| 1488.86 | 0.00734 | 0.01258 |  | 0.02247 | 0.03396 | 0.00799 |
| 1489.3 | 0.00680 | 0.01293 |  | 0.02291 | 0.03396 | 0.00854 |
| 1489.73 | 0.00675 | 0.01353 |  | 0.02325 | 0.03396 | 0.01000 |
| 1490.16 | 0.00694 | 0.01430 |  | 0.02348 | 0.03360 | 0.01096 |
| 1490.6 | 0.00727 | 0.01417 |  | 0.02356 | 0.03266 | 0.01082 |
| 1491.03 | 0.00787 | 0.01362 |  | 0.02359 | 0.03181 | 0.01020 |
| 1491.46 | 0.00863 | 0.01284 |  | 0.02353 | 0.03113 | 0.00979 |
| 1491.89 | 0.00953 | 0.01281 |  | 0.02340 | 0.03151 | 0.00905 |
| 1492.33 | 0.01020 | 0.01276 |  | 0.02316 | 0.03184 | 0.00911 |
| 1492.76 | 0.01024 | 0.01319 |  | 0.02294 | 0.03242 | 0.00881 |
| 1493.19 | 0.01023 | 0.01379 |  | 0.02279 | 0.03322 | 0.00934 |
| 1493.62 | 0.00985 | 0.01441 |  | 0.02272 | 0.03310 | 0.00941 |
| 1494.06 | 0.00951 | 0.01481 |  | 0.02274 | 0.03164 | 0.00938 |
| 1494.49 | 0.00940 | 0.01481 |  | 0.02291 | 0.03067 | 0.00939 |
| 1494.92 | 0.00955 | 0.01447 |  | 0.02293 | 0.03002 | 0.00969 |
| 1495.35 | 0.01001 | 0.01388 |  | 0.02251 | 0.02975 | 0.00979 |
| 1495.79 | 0.00929 | 0.01302 |  | 0.02280 | 0.02962 | 0.00973 |
| 1496.22 | 0.00839 | 0.01196 |  | 0.02327 | 0.03067 | 0.00966 |
| 1496.65 | 0.00775 | 0.01061 |  | 0.02378 | 0.03169 | 0.00961 |
| 1497.08 | 0.00781 | 0.01030 |  | 0.02265 | 0.03118 | 0.00962 |
| 1497.51 | 0.00825 | 0.01039 |  | 0.02120 | 0.03127 | 0.00961 |
| 1497.95 | 0.00861 | 0.01076 |  | 0.02085 | 0.03244 | 0.00971 |
| 1498.38 | 0.00849 | 0.01159 |  | 0.02163 | 0.03296 | 0.00989 |
| 1498.81 | 0.00843 | 0.01211 |  | 0.02231 | 0.03287 | 0.01000 |
| 1499.24 | 0.00846 | 0.01217 |  | 0.02222 | 0.03287 | 0.00973 |
| 1499.68 | 0.00859 | 0.01262 |  | 0.02178 | 0.03296 | 0.00916 |
| 1500.11 | 0.00884 | 0.01224 |  | 0.02104 | 0.03301 | 0.00779 |
| 1500.54 | 0.00870 | 0.01149 |  | 0.02062 | 0.03306 | 0.00713 |
| 1500.97 | 0.00936 | 0.01116 |  | 0.02066 | 0.03296 | 0.00752 |
| 1501.4 | 0.00924 | 0.01112 |  | 0.02045 | 0.03310 | 0.00764 |
| 1501.84 | 0.00856 | 0.01177 |  | 0.02043 | 0.03393 | 0.00726 |
| 1502.27 | 0.00802 | 0.01211 |  | 0.02040 | 0.03476 | 0.00689 |
| 1502.7 | 0.00769 | 0.01233 |  | 0.02050 | 0.03353 | 0.00681 |
| 1503.13 | 0.00757 | 0.01239 |  | 0.02098 | 0.03281 | 0.00699 |
| 1503.56 | 0.00752 | 0.01228 |  | 0.02186 | 0.03234 | 0.00732 |
| 1503.99 | 0.00823 | 0.01195 |  | 0.02243 | 0.03212 | 0.00779 |
| 1504.42 | 0.00813 | 0.01161 |  | 0.02255 | 0.03215 | 0.00822 |
| 1504.86 | 0.00742 | 0.01173 |  | 0.02265 | 0.03179 | 0.00869 |
| 1505.29 | 0.00738 | 0.01244 |  | 0.02281 | 0.03142 | 0.00903 |
| 1505.72 | 0.00743 | 0.01278 |  | 0.02295 | 0.03142 | 0.00877 |
| 1506.15 | 0.00770 | 0.01266 |  | 0.02307 | 0.03161 | 0.00822 |
| 1506.58 | 0.00903 | 0.01262 |  | 0.02323 | 0.03144 | 0.00633 |
| 1507.01 | 0.00906 | 0.01245 |  | 0.02336 | 0.03047 | 0.00616 |
| 1507.45 | 0.00914 | 0.01225 |  | 0.02343 | 0.02985 | 0.00643 |
| 1507.88 | 0.00921 | 0.01248 |  | 0.02315 | 0.02946 | 0.00678 |
| 1508.31 | 0.00849 | 0.01287 |  | 0.02300 | 0.02927 | 0.00721 |
| 1508.74 | 0.00820 | 0.01339 |  | 0.02296 | 0.02920 | 0.00771 |
| 1509.17 | 0.00836 | 0.01405 |  | 0.02259 | 0.02913 | 0.00826 |
| 1509.6 | 0.00842 | 0.01351 |  | 0.02160 | 0.02872 | 0.00901 |
| 1510.03 | 0.00845 | 0.01330 |  | 0.02092 | 0.02769 | 0.00974 |
| 1510.46 | 0.00847 | 0.01338 |  | 0.02050 | 0.02770 | 0.00902 |
| 1510.9 | 0.00853 | 0.01373 |  | 0.02022 | 0.02888 | 0.00835 |
| 1511.33 | 0.00858 | 0.01440 |  | 0.02073 | 0.02930 | 0.00819 |
| 1511.76 | 0.00878 | 0.01433 |  | 0.02141 | 0.02810 | 0.00810 |
| 1512.19 | 0.00903 | 0.01448 |  | 0.02189 | 0.02350 | 0.00819 |
| 1512.62 | 0.00924 | 0.01476 |  | 0.02239 | 0.02261 | 0.00850 |
| 1513.05 | 0.00933 | 0.01479 |  | 0.02268 | 0.02433 | 0.00870 |
| 1513.48 | 0.00919 | 0.01469 |  | 0.02288 | 0.02993 | 0.00845 |
| 1513.91 | 0.00888 | 0.01434 |  | 0.02315 | 0.03083 | 0.00988 |
| 1514.34 | 0.00839 | 0.01387 |  | 0.02347 | 0.03155 | 0.01029 |
| 1514.78 | 0.00780 | 0.01301 |  | 0.02366 | 0.03215 | 0.01008 |
| 1515.21 | 0.00706 | 0.01221 |  | 0.02367 | 0.03226 | 0.00945 |
| 1515.64 | 0.00695 | 0.01167 |  | 0.02354 | 0.03107 | 0.00863 |
| 1516.07 | 0.00771 | 0.01229 |  | 0.02333 | 0.02884 | 0.00796 |
| 1516.5 | 0.00839 | 0.01210 |  | 0.02320 | 0.02596 | 0.00743 |
| 1516.93 | 0.00938 | 0.01199 |  | 0.02304 | 0.02625 | 0.00720 |
| 1517.36 | 0.01013 | 0.01174 |  | 0.02245 | 0.02956 | 0.00719 |
| 1517.79 | 0.01078 | 0.01137 |  | 0.02186 | 0.02903 | 0.00739 |
| 1518.22 | 0.01086 | 0.01074 |  | 0.02143 | 0.02880 | 0.00737 |
| 1518.65 | 0.01018 | 0.01013 |  | 0.02118 | 0.02886 | 0.00780 |
| 1519.08 | 0.00945 | 0.00986 |  | 0.02116 | 0.02981 | 0.00828 |
| 1519.51 | 0.00917 | 0.01036 |  | 0.02135 | 0.03050 | 0.00861 |
| 1519.94 | 0.00886 | 0.01092 |  | 0.02119 | 0.03119 | 0.00883 |
| 1520.37 | 0.00860 | 0.01146 |  | 0.02111 | 0.03179 | 0.00894 |
| 1520.81 | 0.00846 | 0.01143 |  | 0.02111 | 0.03209 | 0.00872 |
| 1521.24 | 0.00838 | 0.01158 |  | 0.02122 | 0.03165 | 0.00832 |
| 1521.67 | 0.00854 | 0.01165 |  | 0.02139 | 0.03117 | 0.00785 |
| 1522.1 | 0.00853 | 0.01158 |  | 0.02171 | 0.02840 | 0.00738 |
| 1522.53 | 0.00841 | 0.01149 |  | 0.02040 | 0.02680 | 0.00693 |
| 1522.96 | 0.00847 | 0.01142 |  | 0.02061 | 0.02726 | 0.00657 |
| 1523.39 | 0.00885 | 0.01139 |  | 0.02041 | 0.02686 | 0.00667 |
| 1523.82 | 0.00871 | 0.01144 |  | 0.02036 | 0.02588 | 0.00674 |
| 1524.25 | 0.00848 | 0.01162 |  | 0.01659 | 0.02705 | 0.00694 |
| 1524.68 | 0.00816 | 0.01196 |  | 0.01676 | 0.02860 | 0.00727 |
| 1525.11 | 0.00778 | 0.01215 |  | 0.01869 | 0.02971 | 0.00761 |
| 1525.54 | 0.00731 | 0.01241 |  | 0.01845 | 0.03103 | 0.00753 |
| 1525.97 | 0.00703 | 0.01244 |  | 0.01836 | 0.03180 | 0.00797 |
| 1526.4 | 0.00724 | 0.01198 |  | 0.01832 | 0.03151 | 0.00834 |
| 1526.83 | 0.00780 | 0.01164 |  | 0.01885 | 0.03052 | 0.00872 |
| 1527.26 | 0.00892 | 0.01118 |  | 0.01978 | 0.02915 | 0.00917 |
| 1527.69 | 0.01014 | 0.01101 |  | 0.02121 | 0.02790 | 0.00937 |
| 1528.12 | 0.00996 | 0.01099 |  | 0.02209 | 0.02695 | 0.00900 |
| 1528.55 | 0.00968 | 0.01156 |  | 0.02265 | 0.02613 | 0.00886 |
| 1528.98 | 0.00944 | 0.01196 |  | 0.02255 | 0.02633 | 0.00867 |
| 1529.41 | 0.00895 | 0.01198 |  | 0.02156 | 0.02686 | 0.00870 |
| 1529.84 | 0.00855 | 0.01201 |  | 0.02081 | 0.02976 | 0.00934 |
| 1530.27 | 0.00832 | 0.01199 |  | 0.02046 | 0.03305 | 0.00979 |
| 1530.7 | 0.00830 | 0.01195 |  | 0.01992 | 0.03173 | 0.00989 |
| 1531.13 | 0.00833 | 0.01184 |  | 0.01973 | 0.02989 | 0.00984 |
| 1531.56 | 0.00832 | 0.01172 |  | 0.01994 | 0.02871 | 0.00960 |
| 1531.99 | 0.00892 | 0.01141 |  | 0.02061 | 0.02850 | 0.00933 |
| 1532.42 | 0.00938 | 0.01094 |  | 0.02161 | 0.02857 | 0.00915 |
| 1532.85 | 0.00958 | 0.01058 |  | 0.02253 | 0.02865 | 0.00834 |
| 1533.27 | 0.00965 | 0.01128 |  | 0.02316 | 0.02877 | 0.00871 |
| 1533.7 | 0.00919 | 0.01238 |  | 0.02348 | 0.02918 | 0.00858 |
| 1534.13 | 0.00782 | 0.01313 |  | 0.02350 | 0.02979 | 0.00844 |
| 1534.56 | 0.00709 | 0.01350 |  | 0.02318 | 0.03048 | 0.00833 |
| 1534.99 | 0.00662 | 0.01391 |  | 0.02244 | 0.02977 | 0.00825 |
| 1535.42 | 0.00658 | 0.01406 |  | 0.02117 | 0.02764 | 0.00820 |
| 1535.85 | 0.00679 | 0.01391 |  | 0.01921 | 0.02748 | 0.00824 |
| 1536.28 | 0.00690 | 0.01327 |  | 0.01807 | 0.02926 | 0.00871 |
| 1536.71 | 0.00725 | 0.01248 |  | 0.01905 | 0.03070 | 0.00914 |
| 1537.14 | 0.00770 | 0.01133 |  | 0.01937 | 0.03132 | 0.00892 |
| 1537.57 | 0.00836 | 0.01056 |  | 0.01992 | 0.03067 | 0.00845 |
| 1538 | 0.00909 | 0.00981 |  | 0.02025 | 0.02985 | 0.00770 |
| 1538.43 | 0.00998 | 0.01024 |  | 0.02004 | 0.02945 | 0.00688 |
| 1538.86 | 0.01053 | 0.01202 |  | 0.01969 | 0.02934 | 0.00668 |
| 1539.29 | 0.00985 | 0.01205 |  | 0.01932 | 0.02942 | 0.00656 |
| 1539.71 | 0.00925 | 0.01203 |  | 0.01912 | 0.02923 | 0.00687 |
| 1540.14 | 0.00864 | 0.01190 |  | 0.01908 | 0.02707 | 0.00758 |
| 1540.57 | 0.00812 | 0.01185 |  | 0.01930 | 0.02551 | 0.00682 |
| 1541 | 0.00774 | 0.01186 |  | 0.01954 | 0.02776 | 0.00629 |
| 1541.43 | 0.00750 | 0.01192 |  | 0.01985 | 0.03119 | 0.00644 |
| 1541.86 | 0.00748 | 0.01198 |  | 0.02021 | 0.03053 | 0.00672 |
| 1542.29 | 0.00774 | 0.01213 |  | 0.02059 | 0.02958 | 0.00730 |
| 1542.72 | 0.00877 | 0.01232 |  | 0.02075 | 0.02836 | 0.00805 |
| 1543.15 | 0.00889 | 0.01267 |  | 0.02028 | 0.02982 | 0.00903 |
| 1543.57 | 0.00860 | 0.01324 |  | 0.01995 | 0.02901 | 0.00848 |
| 1544 | 0.00829 | 0.01344 |  | 0.02033 | 0.02799 | 0.00802 |
| 1544.43 | 0.00790 | 0.01293 |  | 0.02046 | 0.02797 | 0.00789 |
| 1544.86 | 0.00749 | 0.01222 |  | 0.01972 | 0.02944 | 0.00779 |
| 1545.29 | 0.00705 | 0.01139 |  | 0.01910 | 0.03023 | 0.00772 |
| 1545.72 | 0.00677 | 0.01200 |  | 0.01871 | 0.03112 | 0.00763 |
| 1546.15 | 0.00712 | 0.01246 |  | 0.01884 | 0.03108 | 0.00752 |
| 1546.57 | 0.00727 | 0.01275 |  | 0.01900 | 0.03089 | 0.00729 |
| 1547 | 0.00742 | 0.01283 |  | 0.01928 | 0.03060 | 0.00747 |
| 1547.43 | 0.00748 | 0.01268 |  | 0.01964 | 0.03014 | 0.00801 |
| 1547.86 | 0.00741 | 0.01226 |  | 0.02010 | 0.02965 | 0.00824 |
| 1548.29 | 0.00733 | 0.01161 |  | 0.02053 | 0.02935 | 0.00816 |
| 1548.72 | 0.00753 | 0.01082 |  | 0.02090 | 0.02925 | 0.00801 |
| 1549.15 | 0.00742 | 0.00987 |  | 0.02122 | 0.02906 | 0.00796 |
| 1549.57 | 0.00746 | 0.00958 |  | 0.02143 | 0.03091 | 0.00797 |
| 1550 | 0.00741 | 0.00997 |  | 0.02155 | 0.03231 | 0.00809 |
| 1550.43 | 0.00754 | 0.01053 |  | 0.02156 | 0.03247 | 0.00828 |
| 1550.86 | 0.00779 | 0.01137 |  | 0.02150 | 0.03218 | 0.00840 |
| 1551.29 | 0.00817 | 0.01108 |  | 0.02143 | 0.03150 | 0.00759 |
| 1551.71 | 0.00852 | 0.01115 |  | 0.02140 | 0.03047 | 0.00777 |
| 1552.14 | 0.00877 | 0.01223 |  | 0.02153 | 0.02999 | 0.00829 |
| 1552.57 | 0.00895 | 0.01347 |  | 0.02180 | 0.02970 | 0.00910 |
| 1553 | 0.00906 | 0.01367 |  | 0.02219 | 0.02964 | 0.00944 |
| 1553.43 | 0.00911 | 0.01380 |  | 0.02272 | 0.02893 | 0.00941 |
| 1553.86 | 0.00911 | 0.01384 |  | 0.02338 | 0.02951 | 0.00926 |
| 1554.28 | 0.00904 | 0.01379 |  | 0.02404 | 0.03044 | 0.00890 |
| 1554.71 | 0.00885 | 0.01367 |  | 0.02373 | 0.03159 | 0.00784 |
| 1555.14 | 0.00865 | 0.01348 |  | 0.02340 | 0.03192 | 0.00688 |
| 1555.57 | 0.00843 | 0.01322 |  | 0.02305 | 0.03186 | 0.00687 |
| 1555.99 | 0.00834 | 0.01292 |  | 0.02269 | 0.03137 | 0.00753 |
| 1556.42 | 0.00834 | 0.01261 |  | 0.02255 | 0.03030 | 0.00808 |
| 1556.85 | 0.00853 | 0.01238 |  | 0.02235 | 0.02938 | 0.00847 |
| 1557.28 | 0.00896 | 0.01219 |  | 0.02217 | 0.02800 | 0.00883 |
| 1557.71 | 0.00902 | 0.01212 |  | 0.02168 | 0.02770 | 0.00905 |
| 1558.13 | 0.00828 | 0.01214 |  | 0.02113 | 0.02801 | 0.00908 |
| 1558.56 | 0.00752 | 0.01251 |  | 0.02053 | 0.02844 | 0.00840 |
| 1558.99 | 0.00733 | 0.01294 |  | 0.01993 | 0.02915 | 0.00890 |
| 1559.42 | 0.00780 | 0.01327 |  | 0.01952 | 0.02980 | 0.00961 |
| 1559.85 | 0.00826 | 0.01342 |  | 0.02199 | 0.03030 | 0.01064 |
| 1560.27 | 0.00860 | 0.01320 |  | 0.02212 | 0.03069 | 0.00993 |
| 1560.7 | 0.00878 | 0.01257 |  | 0.02207 | 0.03095 | 0.00857 |
| 1561.13 | 0.00874 | 0.01274 |  | 0.02192 | 0.03105 | 0.00749 |
| 1561.56 | 0.00838 | 0.01283 |  | 0.02171 | 0.03101 | 0.00670 |
| 1561.98 | 0.00796 | 0.01281 |  | 0.02156 | 0.03083 | 0.00696 |
| 1562.41 | 0.00777 | 0.01261 |  | 0.02149 | 0.03054 | 0.00744 |
| 1562.84 | 0.00781 | 0.01222 |  | 0.02177 | 0.03017 | 0.00801 |
| 1563.26 | 0.00737 | 0.01165 |  | 0.02227 | 0.02979 | 0.00845 |
| 1563.69 | 0.00704 | 0.01105 |  | 0.02229 | 0.02933 | 0.00885 |
| 1564.12 | 0.00695 | 0.01091 |  | 0.02200 | 0.02893 | 0.00913 |
| 1564.55 | 0.00706 | 0.01159 |  | 0.02118 | 0.02874 | 0.00895 |
| 1564.97 | 0.00737 | 0.01115 |  | 0.02113 | 0.02873 | 0.00915 |
| 1565.4 | 0.00792 | 0.01120 |  | 0.02105 | 0.02885 | 0.00949 |
| 1565.83 | 0.00852 | 0.01195 |  | 0.02184 | 0.02916 | 0.00930 |
| 1566.25 | 0.00911 | 0.01229 |  | 0.02134 | 0.02907 | 0.00900 |
| 1566.68 | 0.00910 | 0.01254 |  | 0.02054 | 0.02903 | 0.00836 |
| 1567.11 | 0.00894 | 0.01265 |  | 0.02021 | 0.02944 | 0.00783 |
| 1567.54 | 0.00865 | 0.01260 |  | 0.02013 | 0.02995 | 0.00866 |
| 1567.96 | 0.00830 | 0.01237 |  | 0.02031 | 0.03048 | 0.00951 |
| 1568.39 | 0.00760 | 0.01215 |  | 0.02073 | 0.03102 | 0.00988 |
| 1568.82 | 0.00807 | 0.01196 |  | 0.02148 | 0.03160 | 0.01004 |
| 1569.24 | 0.00859 | 0.01179 |  | 0.02250 | 0.03206 | 0.00988 |
| 1569.67 | 0.00903 | 0.01132 |  | 0.02376 | 0.03186 | 0.00911 |
| 1570.1 | 0.00935 | 0.01078 |  | 0.02518 | 0.03163 | 0.00837 |
| 1570.52 | 0.00933 | 0.01036 |  | 0.02548 | 0.03137 | 0.00805 |
| 1570.95 | 0.00924 | 0.01081 |  | 0.02514 | 0.03103 | 0.00765 |
| 1571.38 | 0.00904 | 0.01167 |  | 0.02473 | 0.03171 | 0.00738 |
| 1571.8 | 0.00831 | 0.01217 |  | 0.02435 | 0.03338 | 0.00724 |
| 1572.23 | 0.00757 | 0.01257 |  | 0.02404 | 0.03347 | 0.00726 |
| 1572.66 | 0.00723 | 0.01282 |  | 0.02340 | 0.03336 | 0.00741 |
| 1573.08 | 0.00710 | 0.01289 |  | 0.02250 | 0.03322 | 0.00769 |
| 1573.51 | 0.00668 | 0.01280 |  | 0.02169 | 0.03227 | 0.00810 |
| 1573.94 | 0.00678 | 0.01241 |  | 0.02118 | 0.03132 | 0.00874 |
| 1574.36 | 0.00691 | 0.01200 |  | 0.02108 | 0.03093 | 0.00960 |
| 1574.79 | 0.00735 | 0.01159 |  | 0.02098 | 0.03096 | 0.01025 |
| 1575.22 | 0.00791 | 0.01129 |  | 0.02051 | 0.03141 | 0.00984 |
| 1575.64 | 0.00881 | 0.01110 |  | 0.02017 | 0.03235 | 0.00955 |
| 1576.07 | 0.00972 | 0.01100 |  | 0.01991 | 0.03300 | 0.00893 |
| 1576.5 | 0.00981 | 0.01101 |  | 0.01975 | 0.03307 | 0.00854 |
| 1576.92 | 0.00949 | 0.01114 |  | 0.01967 | 0.03272 | 0.00832 |
| 1577.35 | 0.00886 | 0.01138 |  | 0.01970 | 0.03232 | 0.00827 |
| 1577.78 | 0.00804 | 0.01175 |  | 0.02017 | 0.03187 | 0.00861 |
| 1578.2 | 0.00736 | 0.01152 |  | 0.02075 | 0.03143 | 0.00890 |
| 1578.63 | 0.00729 | 0.01144 |  | 0.02155 | 0.03108 | 0.00882 |
| 1579.05 | 0.00783 | 0.01157 |  | 0.02238 | 0.02973 | 0.00856 |
| 1579.48 | 0.00831 | 0.01255 |  | 0.02196 | 0.02878 | 0.00807 |
| 1579.9 | 0.00828 | 0.01379 |  | 0.02100 | 0.02799 | 0.00680 |
| 1580.33 | 0.00820 | 0.01347 |  | 0.02027 | 0.02739 | 0.00780 |
| 1580.76 | 0.00804 | 0.01322 |  | 0.02014 | 0.02711 | 0.00834 |
| 1581.18 | 0.00785 | 0.01296 |  | 0.02041 | 0.02717 | 0.00864 |
| 1581.61 | 0.00775 | 0.01024 |  | 0.02121 | 0.02727 | 0.00906 |
| 1582.04 | 0.00780 | 0.00827 |  | 0.02182 | 0.02710 | 0.00958 |
| 1582.46 | 0.00800 | 0.01035 |  | 0.02228 | 0.02573 | 0.00841 |
| 1582.89 | 0.00835 | 0.01214 |  | 0.02252 | 0.02533 | 0.00751 |
| 1583.31 | 0.00887 | 0.01191 |  | 0.02267 | 0.02519 | 0.00700 |
| 1583.74 | 0.00910 | 0.01180 |  | 0.02247 | 0.02537 | 0.00864 |
| 1584.17 | 0.00875 | 0.01154 |  | 0.02203 | 0.02599 | 0.00946 |
| 1584.59 | 0.00819 | 0.01020 |  | 0.02133 | 0.02647 | 0.00979 |
| 1585.02 | 0.00772 | 0.01119 |  | 0.02070 | 0.02709 | 0.00969 |
| 1585.44 | 0.00802 | 0.01269 |  | 0.02023 | 0.02789 | 0.00881 |
| 1585.87 | 0.00858 | 0.01368 |  | 0.01992 | 0.02874 | 0.00824 |
| 1586.29 | 0.00900 | 0.01420 |  | 0.01978 | 0.02953 | 0.00780 |
| 1586.72 | 0.00914 | 0.01213 |  | 0.01976 | 0.03017 | 0.00840 |
| 1587.14 | 0.00884 | 0.01082 |  | 0.01988 | 0.03073 | 0.00901 |
| 1587.57 | 0.00877 | 0.01072 |  | 0.02015 | 0.03107 | 0.00957 |
| 1588 | 0.00868 | 0.01072 |  | 0.02055 | 0.03112 | 0.01010 |
| 1588.42 | 0.00858 | 0.01038 |  | 0.02109 | 0.03075 | 0.01054 |
| 1588.85 | 0.00841 | 0.01047 |  | 0.02172 | 0.03004 | 0.01071 |
| 1589.27 | 0.00818 | 0.01059 |  | 0.02273 | 0.02893 | 0.01023 |
| 1589.7 | 0.00784 | 0.01078 |  | 0.02307 | 0.02866 | 0.00975 |
| 1590.12 | 0.00744 | 0.01101 |  | 0.02200 | 0.02965 | 0.00941 |
| 1590.55 | 0.00697 | 0.01126 |  | 0.02196 | 0.03049 | 0.00912 |
| 1590.97 | 0.00657 | 0.01150 |  | 0.02201 | 0.03116 | 0.00905 |
| 1591.4 | 0.00683 | 0.01175 |  | 0.02220 | 0.02916 | 0.00915 |
| 1591.82 | 0.00763 | 0.01202 |  | 0.02162 | 0.03003 | 0.00937 |
| 1592.25 | 0.00830 | 0.01208 |  | 0.02102 | 0.03199 | 0.00930 |
| 1592.67 | 0.00829 | 0.01199 |  | 0.02063 | 0.03292 | 0.00989 |
| 1593.1 | 0.00799 | 0.01122 |  | 0.02062 | 0.03318 | 0.01025 |
| 1593.52 | 0.00758 | 0.01015 |  | 0.02077 | 0.03354 | 0.01059 |
| 1593.95 | 0.00727 | 0.00851 |  | 0.02089 | 0.03286 | 0.01033 |
| 1594.37 | 0.00698 | 0.01076 |  | 0.02047 | 0.03256 | 0.00984 |
| 1594.8 | 0.00677 | 0.01099 |  | 0.01956 | 0.03232 | 0.00901 |
| 1595.22 | 0.00656 | 0.00997 |  | 0.01868 | 0.03203 | 0.00853 |
| 1595.65 | 0.00653 | 0.00961 |  | 0.01869 | 0.03212 | 0.00865 |
| 1596.07 | 0.00678 | 0.01005 |  | 0.01907 | 0.03195 | 0.00901 |
| 1596.5 | 0.00735 | 0.01116 |  | 0.01942 | 0.03034 | 0.00946 |
| 1596.92 | 0.00795 | 0.01215 |  | 0.02006 | 0.02976 | 0.00881 |
| 1597.35 | 0.00784 | 0.01303 |  | 0.02051 | 0.02955 | 0.00783 |
| 1597.77 | 0.00783 | 0.01336 |  | 0.02062 | 0.02849 | 0.00779 |
| 1598.2 | 0.00812 | 0.01366 |  | 0.02055 | 0.02797 | 0.00803 |
| 1598.62 | 0.00848 | 0.01418 |  | 0.01987 | 0.02738 | 0.00751 |
| 1599.05 | 0.00892 | 0.01389 |  | 0.01950 | 0.02759 | 0.00655 |
| 1599.47 | 0.00913 | 0.01381 |  | 0.02002 | 0.02836 | 0.00530 |
| 1599.9 | 0.00931 | 0.01313 |  | 0.02101 | 0.02920 | 0.00590 |
| 1600.32 | 0.00944 | 0.01259 |  | 0.02195 | 0.02833 | 0.00709 |
| 1600.74 | 0.00953 | 0.01213 |  | 0.02266 | 0.02727 | 0.00824 |
| 1601.17 | 0.00870 | 0.01182 |  | 0.02281 | 0.02706 | 0.00925 |
| 1601.59 | 0.00790 | 0.01191 |  | 0.02275 | 0.02802 | 0.00956 |
| 1602.02 | 0.00749 | 0.01214 |  | 0.02256 | 0.02839 | 0.00986 |
| 1602.44 | 0.00729 | 0.01269 |  | 0.02231 | 0.02865 | 0.01028 |
| 1602.87 | 0.00730 | 0.01261 |  | 0.02161 | 0.02942 | 0.01049 |
| 1603.29 | 0.00751 | 0.01245 |  | 0.02104 | 0.03057 | 0.01020 |
| 1603.72 | 0.00798 | 0.01244 |  | 0.02103 | 0.02955 | 0.00965 |
| 1604.14 | 0.00865 | 0.01256 |  | 0.02102 | 0.02924 | 0.00885 |
| 1604.56 | 0.00890 | 0.01268 |  | 0.02116 | 0.02948 | 0.00797 |
| 1604.99 | 0.00864 | 0.01274 |  | 0.02142 | 0.02974 | 0.00740 |
| 1605.41 | 0.00831 | 0.01276 |  | 0.02181 | 0.02984 | 0.00738 |
| 1605.84 | 0.00797 | 0.01275 |  | 0.02135 | 0.02973 | 0.00766 |
| 1606.26 | 0.00786 | 0.01269 |  | 0.01880 | 0.02940 | 0.00798 |
| 1606.69 | 0.00752 | 0.01260 |  | 0.01767 | 0.02809 | 0.00835 |
| 1607.11 | 0.00740 | 0.01246 |  | 0.01699 | 0.02796 | 0.00883 |
| 1607.53 | 0.00750 | 0.01226 |  | 0.01683 | 0.02673 | 0.00940 |
| 1607.96 | 0.00751 | 0.01197 |  | 0.01682 | 0.02626 | 0.00985 |
| 1608.38 | 0.00753 | 0.01165 |  | 0.01709 | 0.02606 | 0.00963 |
| 1608.81 | 0.00757 | 0.01143 |  | 0.01769 | 0.02620 | 0.00933 |
| 1609.23 | 0.00768 | 0.01158 |  | 0.01872 | 0.02648 | 0.00901 |
| 1609.65 | 0.00786 | 0.01211 |  | 0.01991 | 0.02716 | 0.00859 |
| 1610.08 | 0.00816 | 0.01256 |  | 0.02106 | 0.02783 | 0.00821 |
| 1610.5 | 0.00860 | 0.01267 |  | 0.02203 | 0.02753 | 0.00794 |
| 1610.93 | 0.00910 | 0.01223 |  | 0.02288 | 0.02798 | 0.00777 |
| 1611.35 | 0.00955 | 0.01162 |  | 0.02310 | 0.02856 | 0.00767 |
| 1611.77 | 0.00903 | 0.01159 |  | 0.02321 | 0.02864 | 0.00756 |
| 1612.2 | 0.00802 | 0.01170 |  | 0.02296 | 0.02890 | 0.00742 |
| 1612.62 | 0.00828 | 0.01192 |  | 0.02240 | 0.02854 | 0.00732 |
| 1613.04 | 0.00862 | 0.01210 |  | 0.02165 | 0.02819 | 0.00728 |
| 1613.47 | 0.00777 | 0.01213 |  | 0.02109 | 0.02845 | 0.00725 |
| 1613.89 | 0.00703 | 0.01204 |  | 0.02071 | 0.02740 | 0.00726 |
| 1614.31 | 0.00636 | 0.01172 |  | 0.02042 | 0.02582 | 0.00738 |
| 1614.74 | 0.00598 | 0.01111 |  | 0.02020 | 0.02415 | 0.00760 |
| 1615.16 | 0.00601 | 0.01024 |  | 0.02022 | 0.02313 | 0.00789 |
| 1615.58 | 0.00624 | 0.00977 |  | 0.02034 | 0.02286 | 0.00768 |
| 1616.01 | 0.00664 | 0.01012 |  | 0.02025 | 0.02321 | 0.00683 |
| 1616.43 | 0.00717 | 0.01059 |  | 0.02035 | 0.02418 | 0.00636 |
| 1616.86 | 0.00769 | 0.01104 |  | 0.02084 | 0.02587 | 0.00665 |
| 1617.28 | 0.00801 | 0.01135 |  | 0.02036 | 0.02687 | 0.00687 |
| 1617.7 | 0.00846 | 0.01107 |  | 0.01980 | 0.02632 | 0.00755 |
| 1618.13 | 0.00868 | 0.01099 |  | 0.01984 | 0.02575 | 0.00849 |
| 1618.55 | 0.00875 | 0.01061 |  | 0.01984 | 0.02525 | 0.00900 |
| 1618.97 | 0.00887 | 0.01146 |  | 0.01986 | 0.02535 | 0.00866 |
| 1619.39 | 0.00899 | 0.01226 |  | 0.01987 | 0.02592 | 0.00812 |
| 1619.82 | 0.00907 | 0.01278 |  | 0.01992 | 0.02686 | 0.00747 |
| 1620.24 | 0.00909 | 0.01308 |  | 0.01924 | 0.02775 | 0.00689 |
| 1620.66 | 0.00899 | 0.01252 |  | 0.01873 | 0.02657 | 0.00648 |
| 1621.09 | 0.00880 | 0.01214 |  | 0.01844 | 0.02568 | 0.00651 |
| 1621.51 | 0.00852 | 0.01161 |  | 0.01837 | 0.02503 | 0.00699 |
| 1621.93 | 0.00820 | 0.01113 |  | 0.01899 | 0.02474 | 0.00751 |
| 1622.36 | 0.00788 | 0.01077 |  | 0.01931 | 0.02471 | 0.00791 |
| 1622.78 | 0.00761 | 0.01053 |  | 0.01938 | 0.02494 | 0.00822 |
| 1623.2 | 0.00740 | 0.01042 |  | 0.01912 | 0.02516 | 0.00847 |
| 1623.62 | 0.00728 | 0.01045 |  | 0.01851 | 0.02455 | 0.00866 |
| 1624.05 | 0.00730 | 0.01057 |  | 0.01790 | 0.02434 | 0.00883 |
| 1624.47 | 0.00732 | 0.01049 |  | 0.01749 | 0.02443 | 0.00892 |
| 1624.89 | 0.00731 | 0.01058 |  | 0.01733 | 0.02504 | 0.00877 |
| 1625.32 | 0.00707 | 0.01086 |  | 0.01762 | 0.02563 | 0.00851 |
| 1625.74 | 0.00679 | 0.01137 |  | 0.01804 | 0.02612 | 0.00816 |
| 1626.16 | 0.00648 | 0.01084 |  | 0.01853 | 0.02650 | 0.00774 |
| 1626.58 | 0.00621 | 0.01056 |  | 0.01880 | 0.02652 | 0.00723 |
| 1627.01 | 0.00608 | 0.01215 |  | 0.01905 | 0.02574 | 0.00745 |
| 1627.43 | 0.00646 | 0.01375 |  | 0.01914 | 0.02499 | 0.00763 |
| 1627.85 | 0.00677 | 0.01336 |  | 0.01852 | 0.02454 | 0.00807 |
| 1628.27 | 0.00648 | 0.01269 |  | 0.01797 | 0.02441 | 0.00844 |
| 1628.7 | 0.00619 | 0.01192 |  | 0.01748 | 0.02455 | 0.00816 |
| 1629.12 | 0.00589 | 0.01126 |  | 0.01714 | 0.02488 | 0.00774 |
| 1629.54 | 0.00554 | 0.01080 |  | 0.01697 | 0.02553 | 0.00729 |
| 1629.96 | 0.00555 | 0.01072 |  | 0.01752 | 0.02642 | 0.00695 |
| 1630.39 | 0.00576 | 0.01086 |  | 0.01802 | 0.02673 | 0.00673 |
| 1630.81 | 0.00615 | 0.01105 |  | 0.01866 | 0.02591 | 0.00663 |
| 1631.23 | 0.00666 | 0.01131 |  | 0.01890 | 0.02525 | 0.00679 |
| 1631.65 | 0.00735 | 0.01160 |  | 0.01847 | 0.02479 | 0.00655 |
| 1632.08 | 0.00686 | 0.01139 |  | 0.01826 | 0.02444 | 0.00724 |
| 1632.5 | 0.00578 | 0.01108 |  | 0.01954 | 0.02425 | 0.00705 |
| 1632.92 | 0.00590 | 0.01074 |  | 0.01930 | 0.02411 | 0.00667 |
| 1633.34 | 0.00664 | 0.01038 |  | 0.01902 | 0.02221 | 0.00693 |
| 1633.76 | 0.00798 | 0.01001 |  | 0.01881 | 0.02179 | 0.00724 |
| 1634.19 | 0.00841 | 0.00970 |  | 0.01857 | 0.02179 | 0.00754 |
| 1634.61 | 0.00815 | 0.00948 |  | 0.01836 | 0.02233 | 0.00778 |
| 1635.03 | 0.00779 | 0.00949 |  | 0.01817 | 0.02341 | 0.00792 |
| 1635.45 | 0.00734 | 0.00959 |  | 0.01808 | 0.02485 | 0.00774 |
| 1635.87 | 0.00689 | 0.00978 |  | 0.01809 | 0.02462 | 0.00718 |
| 1636.3 | 0.00696 | 0.01002 |  | 0.01820 | 0.02357 | 0.00662 |
| 1636.72 | 0.00760 | 0.01021 |  | 0.01771 | 0.02240 | 0.00622 |
| 1637.14 | 0.00726 | 0.01040 |  | 0.01826 | 0.02204 | 0.00599 |
| 1637.56 | 0.00701 | 0.01039 |  | 0.01930 | 0.02167 | 0.00607 |
| 1637.98 | 0.00684 | 0.01041 |  | 0.01930 | 0.02274 | 0.00617 |
| 1638.41 | 0.00671 | 0.01041 |  | 0.01911 | 0.02388 | 0.00690 |
| 1638.83 | 0.00660 | 0.01025 |  | 0.01878 | 0.02311 | 0.00644 |
| 1639.25 | 0.00651 | 0.01053 |  | 0.01830 | 0.02290 | 0.00610 |
| 1639.67 | 0.00632 | 0.01063 |  | 0.01767 | 0.02323 | 0.00578 |
| 1640.09 | 0.00615 | 0.01079 |  | 0.01704 | 0.02372 | 0.00641 |
| 1640.51 | 0.00594 | 0.01098 |  | 0.01644 | 0.02411 | 0.00478 |
| 1640.93 | 0.00573 | 0.01122 |  | 0.01625 | 0.02455 | 0.00494 |
| 1641.36 | 0.00557 | 0.01152 |  | 0.01653 | 0.02518 | 0.00547 |
| 1641.78 | 0.00545 | 0.01174 |  | 0.01703 | 0.02581 | 0.00605 |
| 1642.2 | 0.00569 | 0.01127 |  | 0.01744 | 0.02639 | 0.00673 |
| 1642.62 | 0.00608 | 0.01044 |  | 0.01772 | 0.02677 | 0.00744 |
| 1643.04 | 0.00653 | 0.00987 |  | 0.01796 | 0.02692 | 0.00838 |
| 1643.46 | 0.00680 | 0.01010 |  | 0.01820 | 0.02684 | 0.00901 |
| 1643.88 | 0.00682 | 0.01034 |  | 0.01836 | 0.02624 | 0.00873 |
| 1644.31 | 0.00684 | 0.01050 |  | 0.01849 | 0.02512 | 0.00788 |
| 1644.73 | 0.00685 | 0.01045 |  | 0.01858 | 0.02311 | 0.00714 |
| 1645.15 | 0.00683 | 0.01049 |  | 0.01863 | 0.02295 | 0.00701 |
| 1645.57 | 0.00678 | 0.01050 |  | 0.01866 | 0.02421 | 0.00692 |
| 1645.99 | 0.00647 | 0.01047 |  | 0.01868 | 0.02431 | 0.00708 |
| 1646.41 | 0.00592 | 0.01030 |  | 0.01876 | 0.02443 | 0.00745 |
| 1646.83 | 0.00575 | 0.01025 |  | 0.01906 | 0.02456 | 0.00762 |
| 1647.25 | 0.00570 | 0.01035 |  | 0.01938 | 0.02466 | 0.00775 |
| 1647.68 | 0.00589 | 0.01128 |  | 0.01953 | 0.02508 | 0.00790 |
| 1648.1 | 0.00643 | 0.01179 |  | 0.01946 | 0.02526 | 0.00802 |
| 1648.52 | 0.00686 | 0.01084 |  | 0.01901 | 0.02480 | 0.00778 |
| 1648.94 | 0.00703 | 0.00985 |  | 0.01842 | 0.02406 | 0.00785 |
| 1649.36 | 0.00731 | 0.00955 |  | 0.01782 | 0.02325 | 0.00800 |
| 1649.78 | 0.00765 | 0.00960 |  | 0.01712 | 0.02316 | 0.00805 |
| 1650.2 | 0.00794 | 0.01006 |  | 0.01646 | 0.02396 | 0.00796 |
| 1650.62 | 0.00806 | 0.01027 |  | 0.01610 | 0.02513 | 0.00712 |
| 1651.04 | 0.00787 | 0.01039 |  | 0.01583 | 0.02603 | 0.00601 |
| 1651.46 | 0.00806 | 0.01044 |  | 0.01550 | 0.02572 | 0.00606 |
| 1651.88 | 0.00826 | 0.01051 |  | 0.01534 | 0.02618 | 0.00666 |
| 1652.31 | 0.00768 | 0.01051 |  | 0.01570 | 0.02653 | 0.00689 |
| 1652.72 | 0.00730 | 0.01068 |  | 0.01696 | 0.02682 | 0.00657 |
| 1653.15 | 0.00698 | 0.01076 |  | 0.01901 | 0.02699 | 0.00597 |
| 1653.57 | 0.00670 | 0.01087 |  | 0.01909 | 0.02663 | 0.00528 |
| 1653.99 | 0.00655 | 0.01073 |  | 0.01634 | 0.02656 | 0.00465 |
| 1654.41 | 0.00643 | 0.01048 |  | 0.01574 | 0.02676 | 0.00377 |
| 1654.83 | 0.00646 | 0.01042 |  | 0.01590 | 0.02657 | 0.00406 |
| 1655.25 | 0.00595 | 0.01048 |  | 0.01629 | 0.02608 | 0.00500 |
| 1655.67 | 0.00595 | 0.01028 |  | 0.01679 | 0.02518 | 0.00607 |
| 1656.09 | 0.00641 | 0.01036 |  | 0.01739 | 0.02436 | 0.00647 |
| 1656.51 | 0.00721 | 0.01024 |  | 0.01818 | 0.02394 | 0.00645 |
| 1656.93 | 0.00803 | 0.01101 |  | 0.01890 | 0.02368 | 0.00629 |
| 1657.35 | 0.00833 | 0.01145 |  | 0.01947 | 0.02355 | 0.00616 |
| 1657.77 | 0.00813 | 0.01163 |  | 0.01990 | 0.02363 | 0.00593 |
| 1658.19 | 0.00756 | 0.01133 |  | 0.01971 | 0.02387 | 0.00571 |
| 1658.61 | 0.00686 | 0.01085 |  | 0.01856 | 0.02429 | 0.00556 |
| 1659.03 | 0.00639 | 0.01024 |  | 0.01672 | 0.02495 | 0.00588 |
| 1659.45 | 0.00616 | 0.00956 |  | 0.01504 | 0.02569 | 0.00590 |
| 1659.87 | 0.00568 | 0.00856 |  | 0.01433 | 0.02638 | 0.00554 |
| 1660.29 | 0.00450 | 0.00885 |  | 0.01417 | 0.02673 | 0.00516 |
| 1660.71 | 0.00519 | 0.00936 |  | 0.01424 | 0.02694 | 0.00507 |
| 1661.13 | 0.00660 | 0.00966 |  | 0.01487 | 0.02694 | 0.00542 |
| 1661.55 | 0.00668 | 0.00993 |  | 0.01616 | 0.02672 | 0.00581 |
| 1661.97 | 0.00594 | 0.01020 |  | 0.01762 | 0.02636 | 0.00597 |
| 1662.39 | 0.00606 | 0.01028 |  | 0.01893 | 0.02543 | 0.00598 |
| 1662.81 | 0.00662 | 0.01032 |  | 0.01933 | 0.02430 | 0.00601 |
| 1663.23 | 0.00698 | 0.01024 |  | 0.01955 | 0.02510 | 0.00596 |
| 1663.65 | 0.00680 | 0.01010 |  | 0.01933 | 0.02488 | 0.00590 |
| 1664.07 | 0.00678 | 0.00988 |  | 0.01866 | 0.02487 | 0.00573 |
| 1664.49 | 0.00690 | 0.00976 |  | 0.01732 | 0.02499 | 0.00550 |
| 1664.91 | 0.00715 | 0.01023 |  | 0.01588 | 0.02647 | 0.00563 |
| 1665.33 | 0.00763 | 0.00970 |  | 0.01448 | 0.02641 | 0.00588 |
| 1665.75 | 0.00733 | 0.00927 |  | 0.01412 | 0.02602 | 0.00631 |
| 1666.17 | 0.00712 | 0.00902 |  | 0.01448 | 0.02536 | 0.00673 |
| 1666.59 | 0.00700 | 0.00896 |  | 0.01523 | 0.02681 | 0.00684 |
| 1667.01 | 0.00674 | 0.00909 |  | 0.01561 | 0.02663 | 0.00685 |
| 1667.43 | 0.00654 | 0.00937 |  | 0.01553 | 0.02621 | 0.00686 |
| 1667.85 | 0.00648 | 0.00980 |  | 0.01490 | 0.02506 | 0.00674 |
| 1668.27 | 0.00650 | 0.01002 |  | 0.01447 | 0.02440 | 0.00661 |
| 1668.69 | 0.00629 | 0.00995 |  | 0.01443 | 0.02443 | 0.00653 |
| 1669.11 | 0.00605 | 0.00991 |  | 0.01471 | 0.02452 | 0.00649 |
| 1669.53 | 0.00590 | 0.00984 |  | 0.01547 | 0.02474 | 0.00648 |
| 1669.95 | 0.00579 | 0.00969 |  | 0.01658 | 0.02520 | 0.00621 |
| 1670.37 | 0.00585 | 0.00959 |  | 0.01804 | 0.02593 | 0.00588 |
| 1670.79 | 0.00537 | 0.00511 |  | 0.01760 | 0.02536 | 0.00497 |
| 1671.2 | 0.00541 | 0.00778 |  | 0.01684 | 0.02533 | 0.00433 |
| 1671.62 | 0.00556 | 0.00938 |  | 0.01643 | 0.02279 | 0.00381 |
| 1672.04 | 0.00483 | 0.00999 |  | 0.01612 | 0.02115 | 0.00354 |
| 1672.46 | 0.00479 | 0.00926 |  | 0.01616 | 0.02046 | 0.00366 |
| 1672.88 | 0.00519 | 0.00976 |  | 0.01757 | 0.02087 | 0.00415 |
| 1673.3 | 0.00682 | 0.01037 |  | 0.01848 | 0.02358 | 0.00461 |
| 1673.72 | 0.00680 | 0.01083 |  | 0.01836 | 0.02306 | 0.00506 |
| 1674.14 | 0.00662 | 0.01054 |  | 0.01813 | 0.02165 | 0.00549 |
| 1674.56 | 0.00644 | 0.01025 |  | 0.01778 | 0.02220 | 0.00574 |
| 1674.98 | 0.00633 | 0.01004 |  | 0.01737 | 0.02341 | 0.00557 |
| 1675.4 | 0.00672 | 0.00988 |  | 0.01689 | 0.02491 | 0.00600 |
| 1675.81 | 0.00708 | 0.00977 |  | 0.01643 | 0.02457 | 0.00652 |
| 1676.23 | 0.00726 | 0.00945 |  | 0.01699 | 0.02377 | 0.00724 |
| 1676.65 | 0.00707 | 0.00895 |  | 0.01706 | 0.02279 | 0.00801 |
| 1677.07 | 0.00680 | 0.00891 |  | 0.01623 | 0.02195 | 0.00866 |
| 1677.49 | 0.00638 | 0.00887 |  | 0.01546 | 0.02106 | 0.00844 |
| 1677.91 | 0.00575 | 0.00883 |  | 0.01454 | 0.02027 | 0.00829 |
| 1678.33 | 0.00496 | 0.00881 |  | 0.01484 | 0.01963 | 0.00829 |
| 1678.75 | 0.00427 | 0.00882 |  | 0.01603 | 0.01950 | 0.00833 |
| 1679.17 | 0.00391 | 0.00883 |  | 0.01717 | 0.02140 | 0.00858 |
| 1679.59 | 0.00465 | 0.00883 |  | 0.01803 | 0.02238 | 0.00876 |
| 1680 | 0.00628 | 0.00883 |  | 0.01869 | 0.02173 | 0.00879 |
| 1680.42 | 0.00724 | 0.00881 |  | 0.01905 | 0.02198 | 0.00809 |
| 1680.84 | 0.00609 | 0.00879 |  | 0.01920 | 0.02241 | 0.00739 |
| 1681.26 | 0.00677 | 0.00864 |  | 0.01902 | 0.02314 | 0.00677 |
| 1681.68 | 0.00694 | 0.00837 |  | 0.01876 | 0.02415 | 0.00628 |
| 1682.1 | 0.00718 | 0.00798 |  | 0.01735 | 0.02469 | 0.00626 |
| 1682.51 | 0.00698 | 0.00812 |  | 0.01377 | 0.02464 | 0.00603 |
| 1682.93 | 0.00676 | 0.00852 |  | 0.01288 | 0.02450 | 0.00582 |
| 1683.35 | 0.00656 | 0.00931 |  | 0.01320 | 0.02389 | 0.00560 |
| 1683.77 | 0.00637 | 0.01049 |  | 0.01365 | 0.02110 | 0.00533 |
| 1684.19 | 0.00619 | 0.01039 |  | 0.01430 | 0.02174 | 0.00510 |
| 1684.61 | 0.00603 | 0.01055 |  | 0.01585 | 0.02317 | 0.00483 |
| 1685.02 | 0.00588 | 0.01090 |  | 0.01757 | 0.02320 | 0.00467 |
| 1685.44 | 0.00577 | 0.01112 |  | 0.01883 | 0.02302 | 0.00468 |
| 1685.86 | 0.00571 | 0.01040 |  | 0.01956 | 0.02285 | 0.00499 |
| 1686.28 | 0.00575 | 0.00980 |  | 0.01950 | 0.02332 | 0.00537 |
| 1686.7 | 0.00586 | 0.00947 |  | 0.01886 | 0.02447 | 0.00581 |
| 1687.12 | 0.00608 | 0.00951 |  | 0.01775 | 0.02688 | 0.00538 |
| 1687.53 | 0.00574 | 0.00932 |  | 0.01633 | 0.02818 | 0.00503 |
| 1687.95 | 0.00671 | 0.00907 |  | 0.01510 | 0.02878 | 0.00530 |
| 1688.37 | 0.00686 | 0.00924 |  | 0.01417 | 0.02788 | 0.00568 |
| 1688.79 | 0.00706 | 0.00994 |  | 0.01359 | 0.02651 | 0.00581 |
| 1689.21 | 0.00733 | 0.01086 |  | 0.01341 | 0.02480 | 0.00562 |
| 1689.62 | 0.00761 | 0.01085 |  | 0.01353 | 0.02311 | 0.00561 |
| 1690.04 | 0.00667 | 0.01074 |  | 0.01399 | 0.02178 | 0.00560 |
| 1690.46 | 0.00707 | 0.01081 |  | 0.01480 | 0.02214 | 0.00570 |
| 1690.88 | 0.00738 | 0.01084 |  | 0.01573 | 0.02370 | 0.00595 |
| 1691.3 | 0.00759 | 0.01082 |  | 0.01636 | 0.02324 | 0.00614 |
| 1691.71 | 0.00765 | 0.00896 |  | 0.01651 | 0.02359 | 0.00612 |
| 1692.13 | 0.00751 | 0.00814 |  | 0.01651 | 0.02303 | 0.00615 |
| 1692.55 | 0.00704 | 0.00790 |  | 0.01636 | 0.02212 | 0.00624 |
| 1692.97 | 0.00666 | 0.00853 |  | 0.01620 | 0.01890 | 0.00525 |
| 1693.38 | 0.00635 | 0.00978 |  | 0.01599 | 0.01746 | 0.00462 |
| 1693.8 | 0.00604 | 0.00905 |  | 0.01583 | 0.01833 | 0.00454 |
| 1694.22 | 0.00579 | 0.00838 |  | 0.01577 | 0.02020 | 0.00459 |
| 1694.64 | 0.00557 | 0.00786 |  | 0.01577 | 0.02182 | 0.00484 |
| 1695.05 | 0.00540 | 0.00766 |  | 0.01583 | 0.02326 | 0.00502 |
| 1695.47 | 0.00527 | 0.00840 |  | 0.01592 | 0.02348 | 0.00531 |
| 1695.89 | 0.00518 | 0.00818 |  | 0.01603 | 0.02251 | 0.00547 |
| 1696.31 | 0.00516 | 0.00789 |  | 0.01620 | 0.02213 | 0.00530 |
| 1696.72 | 0.00523 | 0.00769 |  | 0.01601 | 0.02177 | 0.00555 |
| 1697.14 | 0.00570 | 0.00748 |  | 0.01570 | 0.02146 | 0.00575 |
| 1697.56 | 0.00650 | 0.00766 |  | 0.01526 | 0.02144 | 0.00568 |
| 1697.98 | 0.00617 | 0.00874 |  | 0.01488 | 0.02168 | 0.00553 |
| 1698.39 | 0.00573 | 0.00945 |  | 0.01471 | 0.02205 | 0.00532 |
| 1698.81 | 0.00507 | 0.00942 |  | 0.01471 | 0.02252 | 0.00507 |
| 1699.23 | 0.00496 | 0.00937 |  | 0.01540 | 0.02304 | 0.00481 |
| 1699.65 | 0.00513 | 0.00932 |  | 0.01613 | 0.02341 | 0.00470 |
| 1700.06 | 0.00490 | 0.00926 |  | 0.01711 | 0.02255 | 0.00410 |
| 1700.48 | 0.00484 | 0.00917 |  | 0.01777 | 0.02296 | 0.00402 |
| 1700.9 | 0.00500 | 0.00832 |  | 0.01793 | 0.02345 | 0.00353 |
| 1701.31 | 0.00533 | 0.00787 |  | 0.01775 | 0.02370 | 0.00400 |
| 1701.73 | 0.00582 | 0.00821 |  | 0.01732 | 0.02388 | 0.00457 |
| 1702.15 | 0.00633 | 0.00879 |  | 0.01672 | 0.02352 | 0.00498 |
| 1702.57 | 0.00583 | 0.00862 |  | 0.01614 | 0.02280 | 0.00519 |
| 1702.98 | 0.00554 | 0.00840 |  | 0.01574 | 0.02185 | 0.00543 |
| 1703.4 | 0.00521 | 0.00802 |  | 0.01558 | 0.02080 | 0.00574 |
| 1703.82 | 0.00500 | 0.00758 |  | 0.01565 | 0.02189 | 0.00607 |
| 1704.23 | 0.00657 | 0.00770 |  | 0.01604 | 0.02135 | 0.00642 |
| 1704.65 | 0.00637 | 0.00765 |  | 0.01638 | 0.02065 | 0.00670 |
| 1705.07 | 0.00609 | 0.00800 |  | 0.01653 | 0.02062 | 0.00703 |
| 1705.48 | 0.00572 | 0.00903 |  | 0.01649 | 0.02063 | 0.00722 |
| 1705.9 | 0.00515 | 0.00908 |  | 0.01596 | 0.02075 | 0.00721 |
| 1706.32 | 0.00421 | 0.00921 |  | 0.01542 | 0.01913 | 0.00660 |
| 1706.73 | 0.00369 | 0.00948 |  | 0.01490 | 0.01985 | 0.00645 |
| 1707.15 | 0.00392 | 0.00940 |  | 0.01454 | 0.02065 | 0.00594 |
| 1707.57 | 0.00489 | 0.00921 |  | 0.01434 | 0.02141 | 0.00554 |
| 1707.98 | 0.00656 | 0.00906 |  | 0.01450 | 0.02143 | 0.00514 |
| 1708.4 | 0.00715 | 0.00895 |  | 0.01529 | 0.02143 | 0.00477 |
| 1708.82 | 0.00682 | 0.00920 |  | 0.01609 | 0.02134 | 0.00451 |
| 1709.23 | 0.00590 | 0.00936 |  | 0.01683 | 0.02122 | 0.00430 |
| 1709.65 | 0.00521 | 0.00938 |  | 0.01735 | 0.02098 | 0.00417 |
| 1710.07 | 0.00473 | 0.00935 |  | 0.01691 | 0.02086 | 0.00409 |
| 1710.48 | 0.00432 | 0.00930 |  | 0.01662 | 0.02124 | 0.00407 |
| 1710.9 | 0.00480 | 0.00922 |  | 0.01638 | 0.02244 | 0.00401 |
| 1711.31 | 0.00476 | 0.00923 |  | 0.01618 | 0.02283 | 0.00374 |
| 1711.73 | 0.00510 | 0.00934 |  | 0.01614 | 0.02328 | 0.00369 |
| 1712.15 | 0.00628 | 0.00971 |  | 0.01624 | 0.02361 | 0.00368 |
| 1712.56 | 0.00679 | 0.01094 |  | 0.01670 | 0.02380 | 0.00378 |
| 1712.98 | 0.00647 | 0.01146 |  | 0.01637 | 0.02390 | 0.00394 |
| 1713.4 | 0.00613 | 0.01124 |  | 0.01607 | 0.02394 | 0.00428 |
| 1713.81 | 0.00608 | 0.00995 |  | 0.01557 | 0.02389 | 0.00468 |
| 1714.23 | 0.00605 | 0.00728 |  | 0.01510 | 0.02320 | 0.00511 |
| 1714.64 | 0.00602 | 0.00421 |  | 0.01466 | 0.02251 | 0.00552 |
| 1715.06 | 0.00599 | 0.00597 |  | 0.01447 | 0.02181 | 0.00560 |
| 1715.48 | 0.00595 | 0.00756 |  | 0.01521 | 0.02114 | 0.00555 |
| 1715.89 | 0.00592 | 0.00856 |  | 0.01561 | 0.02054 | 0.00517 |
| 1716.31 | 0.00586 | 0.00896 |  | 0.01605 | 0.02005 | 0.00507 |
| 1716.72 | 0.00580 | 0.00962 |  | 0.01619 | 0.01970 | 0.00543 |
| 1717.14 | 0.00577 | 0.00907 |  | 0.01627 | 0.02006 | 0.00576 |
| 1717.56 | 0.00583 | 0.00789 |  | 0.01627 | 0.02057 | 0.00608 |
| 1717.97 | 0.00624 | 0.00824 |  | 0.01641 | 0.02131 | 0.00568 |
| 1718.39 | 0.00654 | 0.00877 |  | 0.01712 | 0.02205 | 0.00516 |
| 1718.8 | 0.00654 | 0.00873 |  | 0.01755 | 0.02250 | 0.00473 |
| 1719.22 | 0.00641 | 0.00914 |  | 0.01772 | 0.02236 | 0.00454 |
| 1719.63 | 0.00624 | 0.00857 |  | 0.01711 | 0.02192 | 0.00443 |
| 1720.05 | 0.00608 | 0.00795 |  | 0.01607 | 0.02122 | 0.00444 |
| 1720.47 | 0.00600 | 0.00788 |  | 0.01483 | 0.02034 | 0.00458 |
| 1720.88 | 0.00586 | 0.00774 |  | 0.01361 | 0.01909 | 0.00483 |
| 1721.3 | 0.00593 | 0.00787 |  | 0.01342 | 0.01893 | 0.00519 |
| 1721.71 | 0.00577 | 0.00822 |  | 0.01369 | 0.01929 | 0.00552 |
| 1722.13 | 0.00552 | 0.00871 |  | 0.01415 | 0.01976 | 0.00573 |
| 1722.54 | 0.00537 | 0.00922 |  | 0.01467 | 0.01997 | 0.00547 |
| 1722.96 | 0.00530 | 0.00959 |  | 0.01499 | 0.02035 | 0.00551 |
| 1723.37 | 0.00536 | 0.00912 |  | 0.01528 | 0.02068 | 0.00587 |
| 1723.79 | 0.00381 | 0.00909 |  | 0.01549 | 0.02107 | 0.00595 |
| 1724.21 | 0.00066 | 0.00890 |  | 0.01561 | 0.02138 | 0.00616 |
| 1724.62 | 0.00483 | 0.00862 |  | 0.01532 | 0.02159 | 0.00598 |
| 1725.04 | 0.00671 | 0.00853 |  | 0.01503 | 0.02145 | 0.00462 |
| 1725.45 | 0.00681 | 0.00853 |  | 0.01608 | 0.01912 | 0.00375 |
| 1725.87 | 0.00660 | 0.00880 |  | 0.01730 | 0.01946 | 0.00342 |
| 1726.28 | 0.00644 | 0.00927 |  | 0.01708 | 0.02131 | 0.00363 |
| 1726.7 | 0.00362 | 0.00954 |  | 0.01695 | 0.02172 | 0.00429 |
| 1727.11 | 0.00077 | 0.00964 |  | 0.01550 | 0.02193 | 0.00464 |
| 1727.53 | 0.00568 | 0.00966 |  | 0.01642 | 0.02222 | 0.00514 |
| 1727.94 | 0.00739 | 0.00965 |  | 0.01715 | 0.02243 | 0.00561 |
| 1728.36 | 0.00519 | 0.00954 |  | 0.01717 | 0.02083 | 0.00552 |
| 1728.77 | 0.00493 | 0.00937 |  | 0.01701 | 0.02091 | 0.00512 |
| 1729.19 | 0.00284 | 0.00907 |  | 0.01662 | 0.02116 | 0.00484 |
| 1729.6 | 0.00504 | 0.00869 |  | 0.01599 | 0.02469 | 0.00443 |
| 1730.02 | 0.00613 | 0.00822 |  | 0.01531 | 0.02422 | 0.00323 |
| 1730.43 | 0.00685 | 0.00780 |  | 0.01485 | 0.02338 | 0.00238 |
| 1730.85 | 0.00711 | 0.00810 |  | 0.01446 | 0.02218 | 0.00177 |
| 1731.26 | 0.00687 | 0.00830 |  | 0.01466 | 0.02040 | 0.00390 |
| 1731.68 | 0.00636 | 0.00727 |  | 0.01567 | 0.01818 | 0.00520 |
| 1732.09 | 0.00569 | 0.00683 |  | 0.01666 | 0.01666 | 0.00593 |
| 1732.51 | 0.00483 | 0.00832 |  | 0.01684 | 0.01625 | 0.00622 |
| 1732.92 | 0.00394 | 0.00881 |  | 0.01660 | 0.01701 | 0.00485 |
| 1733.34 | 0.00375 | 0.00822 |  | 0.01627 | 0.01861 | 0.00401 |
| 1733.75 | 0.00373 | 0.00776 |  | 0.01567 | 0.01998 | 0.00330 |
| 1734.16 | 0.00391 | 0.00752 |  | 0.01505 | 0.02178 | 0.00294 |
| 1734.58 | 0.00437 | 0.00757 |  | 0.01435 | 0.02232 | 0.00436 |
| 1734.99 | 0.00516 | 0.00811 |  | 0.01438 | 0.02202 | 0.00522 |
| 1735.41 | 0.00692 | 0.00892 |  | 0.01441 | 0.02150 | 0.00511 |
| 1735.82 | 0.00784 | 0.00872 |  | 0.01475 | 0.02006 | 0.00524 |
| 1736.24 | 0.00778 | 0.00820 |  | 0.01507 | 0.01892 | 0.00558 |
| 1736.65 | 0.00694 | 0.00726 |  | 0.01470 | 0.01898 | 0.00619 |
| 1737.07 | 0.00663 | 0.00675 |  | 0.01545 | 0.01904 | 0.00554 |
| 1737.48 | 0.00642 | 0.00662 |  | 0.01595 | 0.01909 | 0.00454 |
| 1737.9 | 0.00576 | 0.00694 |  | 0.01614 | 0.01914 | 0.00327 |
| 1738.31 | 0.00521 | 0.00772 |  | 0.01624 | 0.01930 | 0.00218 |
| 1738.72 | 0.00489 | 0.00827 |  | 0.01626 | 0.01862 | 0.00456 |
| 1739.14 | 0.00475 | 0.00791 |  | 0.01615 | 0.01831 | 0.00341 |
| 1739.55 | 0.00483 | 0.00742 |  | 0.01587 | 0.01833 | 0.00351 |
| 1739.97 | 0.00509 | 0.00740 |  | 0.01536 | 0.01869 | 0.00430 |
| 1740.38 | 0.00557 | 0.00798 |  | 0.01413 | 0.01925 | 0.00505 |
| 1740.8 | 0.00623 | 0.00910 |  | 0.01345 | 0.02042 | 0.00568 |
| 1741.21 | 0.00657 | 0.01013 |  | 0.01385 | 0.02166 | 0.00603 |
| 1741.62 | 0.00679 | 0.00945 |  | 0.01419 | 0.02308 | 0.00552 |
| 1742.04 | 0.00699 | 0.00881 |  | 0.01448 | 0.02432 | 0.00514 |
| 1742.45 | 0.00715 | 0.00846 |  | 0.01470 | 0.02440 | 0.00476 |
| 1742.87 | 0.00704 | 0.00831 |  | 0.01507 | 0.02376 | 0.00430 |
| 1743.28 | 0.00617 | 0.00812 |  | 0.01494 | 0.02255 | 0.00383 |
| 1743.69 | 0.00587 | 0.00721 |  | 0.01459 | 0.02086 | 0.00345 |
| 1744.11 | 0.00565 | 0.00663 |  | 0.01413 | 0.01876 | 0.00320 |
| 1744.52 | 0.00553 | 0.00628 |  | 0.01326 | 0.01734 | 0.00326 |
| 1744.93 | 0.00545 | 0.00627 |  | 0.01347 | 0.01737 | 0.00346 |
| 1745.35 | 0.00540 | 0.00665 |  | 0.01403 | 0.01798 | 0.00393 |
| 1745.76 | 0.00532 | 0.00725 |  | 0.01472 | 0.01880 | 0.00465 |
| 1746.18 | 0.00524 | 0.00715 |  | 0.01598 | 0.01972 | 0.00553 |
| 1746.59 | 0.00476 | 0.00721 |  | 0.01696 | 0.02072 | 0.00635 |
| 1747 | 0.00453 | 0.00781 |  | 0.01740 | 0.02059 | 0.00650 |
| 1747.42 | 0.00457 | 0.00875 |  | 0.01654 | 0.01977 | 0.00667 |
| 1747.83 | 0.00493 | 0.00940 |  | 0.01579 | 0.01904 | 0.00687 |
| 1748.24 | 0.00521 | 0.00987 |  | 0.01544 | 0.01693 | 0.00669 |
| 1748.66 | 0.00502 | 0.00911 |  | 0.01536 | 0.01484 | 0.00581 |
| 1749.07 | 0.00480 | 0.00783 |  | 0.01511 | 0.01520 | 0.00529 |
| 1749.48 | 0.00437 | 0.00667 |  | 0.01485 | 0.01627 | 0.00509 |
| 1749.9 | 0.00392 | 0.00625 |  | 0.01458 | 0.01757 | 0.00411 |
| 1750.31 | 0.00356 | 0.00640 |  | 0.01433 | 0.01927 | 0.00370 |
| 1750.72 | 0.00393 | 0.00750 |  | 0.01407 | 0.02048 | 0.00378 |
| 1751.14 | 0.00553 | 0.00812 |  | 0.01385 | 0.02006 | 0.00401 |
| 1751.55 | 0.00580 | 0.00838 |  | 0.01453 | 0.01885 | 0.00342 |
| 1751.96 | 0.00613 | 0.00808 |  | 0.01568 | 0.01871 | 0.00310 |
| 1752.38 | 0.00635 | 0.00782 |  | 0.01629 | 0.01912 | 0.00297 |
| 1752.79 | 0.00599 | 0.00757 |  | 0.01660 | 0.02014 | 0.00307 |
| 1753.2 | 0.00545 | 0.00749 |  | 0.01622 | 0.02182 | 0.00335 |
| 1753.62 | 0.00472 | 0.00788 |  | 0.01561 | 0.02243 | 0.00381 |
| 1754.03 | 0.00454 | 0.00841 |  | 0.01504 | 0.02192 | 0.00443 |
| 1754.44 | 0.00477 | 0.00854 |  | 0.01485 | 0.02068 | 0.00521 |
| 1754.86 | 0.00516 | 0.00899 |  | 0.01493 | 0.02012 | 0.00589 |
| 1755.27 | 0.00495 | 0.00898 |  | 0.01599 | 0.01947 | 0.00535 |
| 1755.68 | 0.00444 | 0.00807 |  | 0.01633 | 0.01910 | 0.00484 |
| 1756.1 | 0.00401 | 0.00756 |  | 0.01642 | 0.01919 | 0.00442 |
| 1756.51 | 0.00363 | 0.00741 |  | 0.01554 | 0.01937 | 0.00462 |
| 1756.92 | 0.00326 | 0.00732 |  | 0.01430 | 0.01666 | 0.00512 |
| 1757.34 | 0.00366 | 0.00721 |  | 0.01389 | 0.01550 | 0.00575 |
| 1757.75 | 0.00407 | 0.00715 |  | 0.01406 | 0.01706 | 0.00628 |
| 1758.16 | 0.00437 | 0.00715 |  | 0.01412 | 0.01757 | 0.00710 |
| 1758.57 | 0.00423 | 0.00715 |  | 0.01424 | 0.01868 | 0.00702 |
| 1758.99 | 0.00402 | 0.00719 |  | 0.01440 | 0.02021 | 0.00650 |
| 1759.4 | 0.00406 | 0.00726 |  | 0.01463 | 0.01941 | 0.00548 |
| 1759.81 | 0.00433 | 0.00737 |  | 0.01491 | 0.01852 | 0.00466 |
| 1760.22 | 0.00486 | 0.00754 |  | 0.01525 | 0.01730 | 0.00474 |
| 1760.64 | 0.00536 | 0.00776 |  | 0.01560 | 0.01690 | 0.00515 |
| 1761.05 | 0.00531 | 0.00808 |  | 0.01593 | 0.01642 | 0.00532 |
| 1761.46 | 0.00496 | 0.00836 |  | 0.01619 | 0.01615 | 0.00542 |
| 1761.87 | 0.00452 | 0.00866 |  | 0.01616 | 0.01577 | 0.00545 |
| 1762.29 | 0.00400 | 0.00896 |  | 0.01605 | 0.01536 | 0.00524 |
| 1762.7 | 0.00340 | 0.00925 |  | 0.01524 | 0.01483 | 0.00497 |
| 1763.11 | 0.00279 | 0.00913 |  | 0.01563 | 0.01441 | 0.00466 |
| 1763.53 | 0.00254 | 0.00815 |  | 0.01591 | 0.01420 | 0.00432 |
| 1763.94 | 0.00326 | 0.00786 |  | 0.01564 | 0.01411 | 0.00385 |
| 1764.35 | 0.00252 | 0.00758 |  | 0.01533 | 0.01417 | 0.00387 |
| 1764.76 | 0.00247 | 0.00798 |  | 0.01496 | 0.01453 | 0.00439 |
[truncated: 69,093 more chars]
